# Circulating mitochondrial DNA promotes M2 polarization of tumor associated macrophages and HCC resistance to sorafenib

**DOI:** 10.1038/s41419-025-07473-8

**Published:** 2025-03-04

**Authors:** Qi Yang, Mengmeng Cui, Jiaxin Wang, Yuan Zhao, Weitao Yin, Ziqian Liao, Yixuan Liang, Zhixiong Jiang, Yujia Li, Jinrong Guo, Lixia Qi, Jiaxing Chen, Jing Zhao, Dengke Bao, Zhi-Xiang Xu

**Affiliations:** 1 School of Life Sciences, School of Pharmacy, Henan University, Kaifeng, Henan 475004 China; 2 The Zhongzhou Laboratory for Integrative Biology, Henan University, Zhengzhou, Henan 450000 China; 3 Laboratory of Cancer Biomarkers and Liquid Biopsy, School of Pharmacy, Henan University, Kaifeng, Henan 475004 China; 4 Department of Thoracic Surgery, The First Affiliated Hospital of Henan University, Kaifeng, Henan 475000 China; 5 State key Laboratory of Antiviral Drugs, School of Pharmacy, Henan University, Kaifeng, Henan 475004 China

**Keywords:** Cancer microenvironment, Cancer therapeutic resistance

## Abstract

Mitochondrial damage-associated molecular patterns (DAMPs) including mitochondrial DNA (mtDNA), TFAM (transcription factor A, mitochondrial), and ATP, which play crucial roles in the regulation of inflammatory environment in human diseases. However, the role of mitochondrial DAMPs in regulating tumor microenvironment (TME) remains unclear. Herein, we demonstrate that infiltration of M2-type tumor-associated macrophages (TAMs) was correlated with the resistance of hepatocellular carcinoma (HCC) to sorafenib. We found that cell-free mtDNA in the plasma was significantly increased in sorafenib-resistant HCC mice. Sorafenib induced mitochondrial dysfunction and promoted the release of mtDNA into extracellular matrix of HCC. Macrophages retook the mtDNA in the TME of HCC, activated TLR9 signaling, and promoted the activation of NF-κB and the polarization of TAMs into M2. Application of DNase I to digest mtDNA or depletion of macrophages with clodronate liposomes reduced M2 macrophage infiltration, decreased the growth of HCC, and sensitized the tumors to sorafenib. Furthermore, we showed that blocking the activation of TLR9 enhanced the therapeutic effect of sorafenib in HCC. Together, we demonstrate that sorafenib treatment leads to the release of mtDNA into TME in HCC, which in turn facilitates the polarization of TAMs into M2 macrophages through TLR9 activation and aggravates the resistance of HCC to sorafenib. Our study reveals a novel mechanism underlying circulating mtDAMPs in remodeling the HCC microenvironment by reprograming the TAMs and provides a new strategy for improving the therapeutic effect of sorafenib and overcoming its resistance in HCC.

## Background

Hepatocellular carcinoma (HCC) is one of the most common cancers in patients with chronic hepatitis and the fourth leading cause of cancer-related deaths in China and worldwide [1–3]. Accumulated evidence shows that 50% HCC patients are diagnosed with an advanced stage which means patients are unable to undergo radical resection [1–3]. Targeted therapy, percutaneous ablation, radiotherapy, chemotherapy, and immunotherapy, or a combination of these treatments are usually administrated for advanced HCC patients [4, 5]. Sorafenib is a multiple-target tyrosine kinase inhibitor (TKI), which is the first molecular-targeted therapy to show efficacy for advanced HCC [5, 6]. Unfortunately, clinical trials showed that only ~30% of HCC patients benefit from sorafenib and almost inevitably develop acquired resistance within 6 months [6, 7]. Previous researches demonstrated that tumor immune microenvironment, epigenetic modification, tumor metabolic switch, and regulated cell death were implicated in initial or acquired sorafenib resistance of HCC [6]. However, the accurate mechanism by which advanced HCC acquires resistance to sorafenib remains largely unknown. Thus, it is urgent to illustrate the underlying mechanisms of acquired sorafenib resistance in advanced HCC patients for effective targeted therapy of the disease.

Tumor immune microenvironment plays an important role in tumourigenesis, progression, metastasis and drug resistance of HCC [8]. Tumor-associated macrophages (TAMs), which are identified as alternatively activated (M2) macrophages, are the most abundant component residing in tumor microenvironment (TME) to build the immunosuppressive environment [9]. TAMs restrain antitumor immunity and facilitate angiogenesis, metastasis and tumor progression through expressing cytokines, chemokines, growth factors and matrix metalloproteases [10]. We previously reported that TAMs recruitment and M2 polarization promoted HCC progression and metastasis through mitochondrial fission-induced mtDNA stress [11, 12], supporting that mtDNA of cancer cells may positively affect the polarization of TAMs. Chen et al. reported that increased TAM infiltration is associated with sorafenib resistance and poor prognosis of HCC patients [13]. Fan and Zhou et al. found that TAMs promote cancer stem cell (CSC)-like properties and epithelial-mesenchymal transition (EMT) in HCC cells to facilitate sorafenib resistance [14, 15]. TAMs trigger mucosal-associated invariant T (MAIT) cell dysfunction through interaction at the tumor-to-liver interface and dampen immune checkpoint blockade (ICB) therapies in HCC [16]. Blocking the recruitment of TAMs and reprogramming these cells into anti-tumoural phenotype have consistently been verified as the potential strategy for overcoming sorafenib resistance and enhancing HCC therapeutic effect [17–19]. However, the underlying mechanism for M2 polarization of TAMs in sorafenib resistance is not well known.

Mitochondria are vital mediators in innate and adaptive immunity, which play multi-functional roles in malignant tumor initiation and progression [20, 21]. Accumulative evidence reveals that various mitochondrial molecules, known as mitochondrial damage-associated molecular patterns (DAMPs), are released to the cytoplasm or extracellular space under stresses and trigger innate and adaptive immune responses by interaction with receptors on cell surface or intracellular [22, 23]. Mitochondrial DAMPs include mitochondrial DNA (mtDNA), TFAM (transcription factor A, mitochondrial), ATP, N-formyl peptide (NFP), etc. [22, 23]. Excessive release of mitochondrial DAMPs is implicated in multiple inflammatory diseases and contributes to cancer and other human diseases [22, 23].

Mitochondrial DNA is known as the only extra-nuclear genetic material in mammalian cells and comprises unmethylated DNA as CpG islands [24, 25]. MtDNA is released into the cytoplasm or extracellular under pathological conditions and recognized by cGAS, NLRP3 inflammasome, and TLR9 to trigger downstream cascade and cytokine secretion and regulate innate immunity [24, 25]. Our previous studies have demonstrated that abnormal mitochondrial dynamics and biogenesis induce cytosolic mtDNA stress and promote the proliferation of HCC and esophageal squamous cell carcinoma (ESCC) cells by activating the TLR9 and/or cGAS-STING pathway [11, 26, 27]. Previous studies have shown that free mtDNA from circulating cells is significantly elevated and acts as a risk biomarker for HCC patients receiving transarterial chemoembolization (TACE) and Traditional Chinese Medicine (TCM) treatment [28]. However, whether circulating mtDNA is involved in M2 polarization of TAMs and sorafenib resistance of HCC remains uncharacterized.

In this study, we demonstrated that infiltration of M2-TAMs was associated with HCC resistance to sorafenib. Sorafenib promoted mtDNA release into extracellular matrix, which in turn facilitates M2 polarization of TAMs through TLR9 activation and aggravates the resistance of HCC to sorafenib. Our findings provide a new insight into the molecular mechanisms underlying M2 polarization of TAMs during sorafenib resistance.

## Methods and Materials

### Cell culture and reagents

Human monocytic THP-1 cells (Procell Life Science and Technology, cat. CL-0233) and HCC cell lines SNU739 (Cobioer Biosciences, cat. CBP60219) were routinely cultured in RPMI-1640 (Corning, cat. 10-040-CVR) medium supplemented with 10% fetal bovine serum (Biological Industries, cat. 04-001-1ACS). Murine HCC cell lines Hepa1-6 (Procell Life Science and Technology, cat. CL-0105) and monocyte/macrophage cell line RAW264.7 (Procell Life Science and Technology, cat. CL-0190) were cultured in Dulbecco’s Modified Eagle Medium (Corning, cat. 10-013-CVR) supplemented with 10% fetal bovine serum. THP-1 monocytic cells were incubated with 150 nM phorbol 12-myristate 13-acetate (PMA, Sigma, cat. 79346) for 24 h to differentiate into THP-1-derived macrophages. All cell lines were verified based on cell morphology and authenticated using short tandem repeat (STR) DNA testing and incubated at 37 °C with a 5% CO_2_ incubator. The antibodies and reagents are listed in Supplementary Table S3.

To prepare conditioned medium (CM) from M2 tumor associated macrophages, 5 × 10^6^/mL THP-1-derived macrophages and RAW264.7 were first incubated with 20 ng/mL IL-13 (MedChemExpress, cat. HY-P70568) and 20 ng/mL IL-4 (MedChemExpress, cat. HY-P70445) for 24 h to polarize into the M2 phenotype. The supernatant was collected and filtered using 0.22 μm filters to prepare M2-CM for further studies.

To prepare CM from HCC, 5×10^6^/mL SNU739 and Hepa1-6 HCC cell lines were treated with 50% inhibitory concentration (IC_50_) of sorafenib indicated condition (SNU739, 16 μM for 24 h or 7 μM for 48 h; Hepa1-6, 30 μM for 24 h or 16 μM for 48 h), especially (Fig. S4). The supernatant was collected and filtered using 0.22 μm filters to prepare HCC-CM for further studies.

### Animals

Four-week-old male C57BL/6 J mice (SCXK2020-0005) were purchased from Beijing Vital River Laboratory Animal Technology. To establish a mouse orthotopic implantation HCC model resistance to sorafenib (*n* = 6), 3 × 10^6^ Hepa1-6 cells that stably expressed luciferase were resuspended in sterile PBS buffer containing 50% Matrigel (Corning, cat. 354234) and orthotopically intrahepatic injected into the left hepatic lobe. Two weeks after the orthotopic implantation, the mice were treated by intragastric administration of sorafenib (Solarbio, cat. S5080, 30 mg/kg/day). The tumor size was evaluated using an IVIS® Lumina LT Series III in vivo imaging system (PerkinElmer, USA). Tumors without reduction in size following 1 week treatment of sorafenib were defined as resistance to sorafenib in the HCC orthotopic mouse models. HCC orthotopic mouse models, which persistent decreased during sorafenib treatment was considered as sensitive group. After the treatment, mice were euthanized to evaluate the hepatic orthotopic tumor [15, 29].

To establish subcutaneous xenograft models, 4-week-old male BALB/c nude mice (SCXK 2019-0008) were purchased from Beijing Vital River Laboratory Animal Technology and bred in an individually ventilated cage system under pathogen-free conditions for 1 week before experiments. 3 × 10^6^ SNU739 cells with or without 1 × 10^6^ THP-1-derived M2-TAMs were injected into the right flank of the nude mice (*n* = 6). After 7 days, the mice were treated by intragastric administration (on gavage) of sorafenib with 30 mg/kg/day. Tumor volume was measured weekly and calculated according to 0.5 × Width^2^× Length. After 6 weeks, all mice were euthanized and the tumors were harvested to photograph and weight.

Diethylnitrosamine (DEN)-induced HCC mouse model was used to evaluate the combined therapeutic effect of blocking the TLR9 pathway and sorafenib. Two-week-old male *Tlr9* wild type (*Tlr9*^+/+^) or knockout (*Tlr9*^-/-^, which was established by our group in the previous study) C57BL/6 J mice were injected intraperitoneal (i.p.) with DEN (25 mg/kg) (Sigma cat. N0756) and fed with regular chow diet for 30 weeks. Then the mice were treated by intragastric administration of sorafenib (30 mg/kg/day) with or without E6446 (20 mg/kg/day) (TargetMol, cat. T4206) for 6 weeks. The vehicle group was treated by intragastric administration of PBS. All mice were euthanized and the body weight and tumor area ratio with tumors were measured and photographed.

The tumor tissue samples were evaluated by H&E staining, flow cytometry, immunofluorescence and immunohistochemistry. Animal study was approved by the Institutional Animal Care and Use Committee of Henan University (Permission number: HUSOM2020-087; Date issued: 2020-03-25).

### Human tissue samples

A total of 55 paired samples of HCC and precancerous tissues were collected from Nanyang Central Hospital of Henan University (Henan, China.). The demographic factors, clinical data, and follow-up information for each patient were summarized in Supplementary Table S1. This study was approved by the Ethics Committee of Henan University (Permission number: HUSOM2020-087; Date issued: 2020-03-25).

### Quantitative reverse transcription PCR (qRT-PCR) and western blot analysis

Total RNA from HCC cells, macrophage cells, patients or mice tumor and peritumor tissues were extracted using TRIzol LS Reagent (Invitrogen, cat. 15596018) and then was reverse transcribed into cDNA using EvoM-MLV RT Premix (Accurate Biology, cat. AG11706). The quantitative real-time PCR was performed using SYBR Green PCR Master Mix (Accurate Biology, China) with a real-time quantitative PCR system (SLAN-96S, China). Primers were synthesized by Sangon Biotech Co., Ltd. (Shanghai, China) and all primer sequences for qRT-PCR involved in this article were provided in Supplementary Table S2.

For western blot analysis, proteins from HCC cells, macrophage cells, patients or mice tumor and peritumor tissues were lysed with radioimmunoprecipitation lysis buffer (RAPI) (SolarBio, cat. R0010) containing 1% phosphatase inhibitor cocktail II (Roche, cat. 04906837001) and 1% protease inhibitor cocktail (Roche, cat. 04693132001). Protein concentration was detected by an enhanced BCA protein assay kit (Solarbio, cat. PC0020). 20 μg of protein lysates were separated by SDS-PAGE electrophoresis and transferred onto PVDF membrane (Millipore, USA) by electroblotting. Then the membranes were subjected to blocking for 1 h at room temperature in 5% skim milk and incubated with specific primary antibodies overnight at 4 °C. After incubating with appropriate HRP-conjugated secondary antibody, membranes were visualized using the ECL Plus chemiluminescence detection system (Solarbio, cat. PE0010) and visualized using a FluorChem Multifluor system (ProteinSimple, USA). The information for all primary and secondary antibodies used in this study was presented in Supplementary Table S3.

### Detection of mtDNA oxidative damage

To detect whether the circulating mtDNA is oxidized, we employed Fpg-sensitive real-time PCR analysis as previously described [30]. This method utilized primers that encompass sequences in the D-loop region and the ND4 gene, which are listed in Supplementary Table S1. The assay is based on the treatment of mtDNA with Fpg, which removes oxidized purines from DNA, thereby creating single-strand breaks and inhibiting PCR amplification at these sites. The differences in qPCR cycles between Fpg-treated and untreated DNA serve as a specific indicator for the presence of oxidative base damage. One aliquot of purified ccf-DNA (250 ng) was incubated with 8 units of Fpg in 1 × NEBuffer 1 and 100 μg/mL BSA in a total volume of 50 μL at 37 °C for 1 h. Fpg was then inactivated by heating at 60 °C for 5 min. Subsequently, 10 ng of DNA was used for the qPCR assay to detect Fpg-sensitive cleavage sites.

### Immunohistochemistry (IHC) and H&E staining

IHC and H&E staining were processed on tissue from patients and mice tumor and peritumor tissues as previously described using Instant Immunohistochemistry Kit (Sangon Biotech, cat. E607318) [27]. Olympus IX53 microscope (Olympus Corporation, Japan) was used to review and photograph the IHC and H&E staining. The result was evaluated independently by two pathologists who were blinded of to the specimens and clinicopathological features of patients. The percentage of positively stained macrophages was calculated. The information for all primary and secondary antibodies utilized for IHC staining was presented in Supplementary Table S3.

### Flow cytometry analysis

To detect macrophage M2 polarization, THP-1-derived macrophages or RAW264.7 were collected after incubation with indicated CM. The cells were washed three times with cold PBS containing 10% human serum and then incubated with the indicated antibody for 1 h. Then cells were washed with PBS buffer and incubated with secondary antibody for 15 min. Immediately, cells were analyzed by Cytomics FC500 (CytoFLEX, China). Single cell suspensions were prepared from fresh mouse tumors by using the mouse tissue dissociation kit (Miltenyi, cat. 130-096-730) following the manufacturer’s instructions. After red blood cell lysis, the cells were centrifuged for 5 min at 500 × g before being suspended in flow cytometry buffer and then incubated with primary antibody at room temperature for 1 h in the dark, washed twice with PBS, suspended in 300 μL PBS and analyzed with a Cytomics FC500 (CytoFLEX, China). The information for all primary and secondary antibodies utilized for flow cytometry was presented in Supplementary Table S3.

Flow cytometry analysis was performed to detect the intracellular ROS using 2’,7’-dichlorofluorescein diacetate (DCFH-DA) fluorescent probe (Beyotime, cat. S0033S) and cell apoptosis using Annexin V-FITC apoptosis detection kit (Beyotime,cat. C1062M) as previously described. Briefly, HCC cells suspension was incubated with 10 μM DCFH-DA or 5 μL Annexin V-FITC and 10 μL PI, which was diluted with serum-free medium, at 37 °C for 20 min. After centrifugation at 1,000 rpm for 5 min, the cells were washed three times, re-suspended in 300 µL PBS and assessed by flow cytometry.

### Immunofluorescence staining analysis

Immunofluorescence staining was used to investigate the CD163 or CD206 positive cell percentage and colocalization of TLR9 and mtDNA within M2 macrophage in tissue and cell samples. For cell immunofluorescence staining, 2 × 10^3^ THP-1-derived macrophages and RAW264.7 were seeded in 24-well plate and treated as indicated. Then, the cells were first fixed with 4% paraformaldehyde in phosphate-buffered saline (PBS; pH 7.4) and permeabilized with 0.1% Triton X-100, blocked with 3% bovine serum albumin (BSA) at room temperature for 1 h. After incubation with specific primary antibodies and appropriate fluorescence-conjugated secondary antibody, 4’,6-diamidino-2-phenylindole (DAPI) (BestBio, cat. BB-4401), the immunolabeled cells were immediately observed with Olympus BX60 fluorescence microscope (Olympus Corporation, Japan) or confocal microscope (Olympus Corporation, Japan).

Paraffin-embedded human and mouse HCC tissue sections were deparaffinized with xylene, rehydrated, unmasked in sodium citrate buffer (10 mmol/L, pH 6.0), and quenched autofluorescence signals using a glycine solution (2 mg/mL), blocked with 3% BSA at room temperature for 1 h. Then, tissue slides were incubated with specific mouse and rabbit primary antibodies overnight at 4 °C and appropriate fluorescence-conjugated secondary antibodies. After nuclear counterstaining with DAPI, the immunolabeled slides were immediately observed with Olympus BX60 fluorescence microscope (Olympus Corporation, Japan) or confocal microscope (Olympus Corporation, Japan). The information for all primary and secondary antibodies utilized for immunofluorescence staining was presented in Supplementary Table S3.

### Cell viability, proliferation and caspase 3/7 activity assays

SNU739 and Hepa1-6 HCC cells were seeded into 96-well culture plate at an appropriate density, which was treated with sorafenib (SNU739, 16 μM; Hepa1-6, 30 μM, especially) in combination with or without M2-CM. Cell viability was determined by 3-(4,5-dimethylthiazol-2-yl)-2,5-diphenyltetrazolium bromide (MTT; 1 mg/mL, Solarbio, cat. M8180) assay. Cell proliferation was analyzed by ethynyl deoxyuridine (EdU) incorporation assay kit (Ribbio, cat. C10310-1) incorporation assay. Caspase 3/7 activity was evaluated using the Caspase-Glo 3/7 assay system (Promega, cat. G8091). All assays were performed according to the manufacturer’s instructions and repeated at least in triplicate.

### Migration assays

Migration potential of macrophages was conducted by a Transwell assay in transwell chambers (Corning, cat. 3401) without Matrigel coating. The 4 × 10^4^ THP-1-derived macrophages or RAW264.7 in 200 μL serum-free medium with 0.1% BSA were seeded into the apical side of the chamber. The 500 μL indicated CM from HCC cells or RPMI-1640 medium containing 20% FBS was filled basolateral side. After incubating for 48 h, the outer membrane with crossed cells was fixed with 4% paraformaldehyde and stained with 0.1% crystal violet solution. Migrated cells were counted and imaged using an inverted microscope (Olympus Corporation, Japan) by five randomly selected fields.

### Detection of cell free mtDNA content, mitochondrial membrane potential (Δψm) and ATP measurement

The mtDNA content in mouse plasma and cell supernatant was measured by qRT-PCR or droplet digital PCR (ddPCR) system as previously described [11, 26]. The primer sequences of the MT-ND1 and 36B4 were provided in Supplementary Table S2.

JC-1 dye was used to detect the mitochondrial membrane potential. HCC cells were adjusted to a density of 1 × 10^5^ /mL and treated with indicated sorafenib and stained with 5 mg/L JC-1 dye (Beyotime, cat. C2006) for 20 min at 37 °C. After washing with JC-1 dye buffer for 3 times, cells were observed under a fluorescence microscope and the fluorescence intensity ratio of JC-1 monomers to aggregates was detected (ratio of 529:590 nm emission intensity).

The luciferase-based ATP assay kit (Beyotime, cat. S0026) was used to detect the cellular ATP levels. Briefly, the standard curve was first established by serially diluted standards according to the instructions. Total protein of HCC cells treated with indicated sorafenib was collected and the RLU value was measured with a Luminometer according to the manufacture of ATP assay kit. Finally, the ATP concentration of the samples was calculated using the standard curve.

### Construction of THP-1 mtDNA-depleted cell lines and DNase I treatment

The parental mtDNA of THP-1 cells and RAW264.7 were specifically depleted through duration treatment with 50 ng/mL ethidium bromide (EtBr) (Amresco, cat. 0492) for 4 weeks.

To digest the cell free mtDNA in CM from HCC cells, 3 μg DNase I was added to CM for 4 h at 37 °C according to the manufacturer’s instructions.

### RNA sequencing analysis

Total RNA of THP-1-derived macrophages treated as indication were extracted as previously described. RNA integrity was determined by Bioanalyzer 2100 (Agilent, Santa Clara, CA) and sequencing sample was constructed. The library was sequenced on Illumina novaseq by Genewiz Suzhou, In. Differentially expressed mRNAs, GO analysis, and KEGG pathway annotation as previously reported [27].

### Statistical analysis

GraphPad Prism 8.3.0 was utilized to conduct the Student’s *t*-test to compare two groups and the one-way analysis of variance (ANOVA) for comparisons among multiple groups. The data were presented as the standard error of the mean (SEM). The *p*-value obtained from the *t*-tests was denoted as follows: *P < 0.05; **P < 0.01; ***P < 0.001. The investigator was blinded to patients’ clinical data and no blinding was done in animal studies.

## Results

### M2-TAMs infiltration is correlated with HCC resistance to sorafenib

**Fig. 1:**
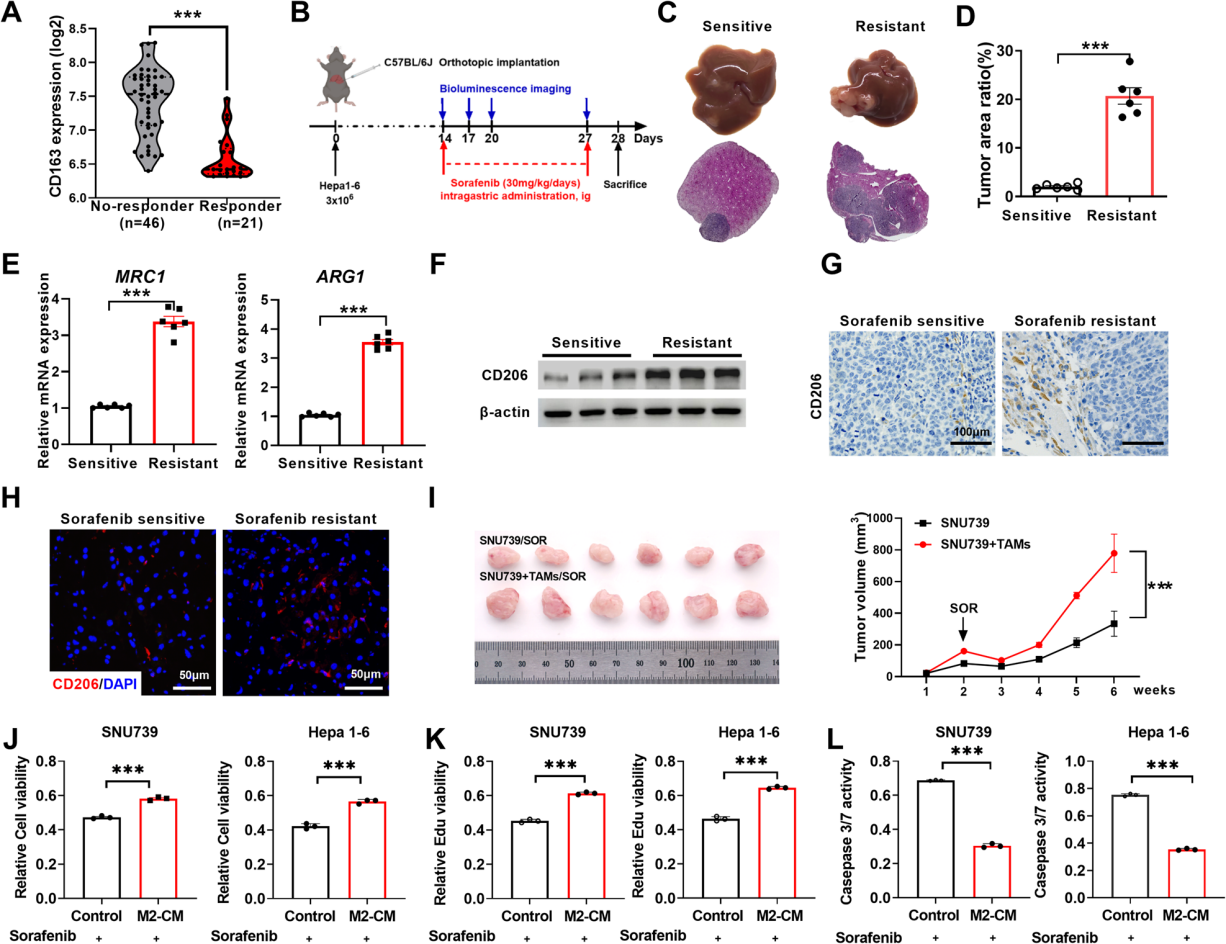
M2-TAMs infiltration is correlated with HCC resistance to sorafenib. **A** CD163 expression in patients which responder or non-responder to sorafenib from the GSE109211 datasets. **B** Schematic description of sorafenib treatment in the mouse orthotopic HCC model (*n* = 6). **C** Representative images of orthotopic tumors and H&E staining in representative group (*n* = 6). **D** The ratio of mouse liver tumors to liver area in indicated groups (*n* = 6). **E** qRT-PCR analysis for the mRNA expression levels of MRC1 and ARG1 in indicated groups. **F** Western blotting analysis for the expression levels of CD206 in indicated groups. **G**, **H** Immunohistochemical (IHC) (**G**) and immunofluorescence (IF) (**H**) analysis for the expression levels of CD206 in representative groups. **I** Representative images and tumor growth curves of subcutaneous xenograft tumor model developed from HCC cells treatment with indication (*n* = 6). **J–L** The sensitivity of HCC cells (SNU739 and Hepa1-6 cells) incubated with M2-CM to sorafenib was determined by cell viability (**J**), cell proliferation (K), and caspase 3/7 activity assays (**L**). M2-CM, Conditioned medium from IL-4 and IL-13 induced M2 macrophages. ***P < 0.001.

TAMs are the most abundant component residing in the TME, which plays a crucial role in HCC progression and chemo-resistance. We first evaluated the abundance of tumor-infiltrating immune cells (TIICs) in HCC tissue using CIBERSORT analysis. We found that M2-TAM infiltration was significantly higher than M1 macrophages in HCC tissues from TCGA (*N* = 371), GSE36376 (*N* = 240), and GSE25097 (*N* = 268) dataset (Fig. S1A). Macrophage infiltration was positively correlated with the expression of CD163, an M2 polarization marker of TAMs (Fig. S1B). Survival analysis showed that HCC patients with high M2-TAM infiltration exhibited a significantly lower overall survival rate in TCGA dataset (Fig. S1C). Subsequently, we explored the M2-TAM infiltration in HCC tissues using the GSE109211 dataset, which contains 67 HCC patients treated with sorafenib and 73 HCC patients treated with placebo. M2-TAM infiltration was also higher than that of M1 macrophages (Fig. S2A). More importantly, M2-TAM infiltration and CD163 expression were markedly higher in tumors from HCC patients resistant to sorafenib as compared with those from patients sensitive to the treatment (Fig. S2B and Fig. 1A). Consistent with the data from TCGA and GEO datasets and our previous findings, the percentage of CD163^+^ cells was significantly up-regulated in peritumor tissues from HCC patients (Fig. S3A- S3E).

To validate the association of M2-TAMs infiltration with the sensitivity of HCC to sorafenib, we established a mouse orthotopic implantation HCC model using Hepa1-6 cells that stably expressed luciferase. Two weeks after the orthotopic implantation, mice received intragastrical administrated of sorafenib (30 mg/kg/day) and visualized using in vivo imaging system (Fig. 1B). Tumor did not further decrease following 1-week exposure to sorafenib, suggesting that the HCC orthotopic mouse models are resistant to sorafenib. HCC orthotopic mouse models, which persistent decreased during sorafenib treatment was considered as sensitive group (Fig. 1C, D). Reverse transcription polymerase chain reaction (RT-PCR) and western blotting (WB) results demonstrated that the expression of M2-TAM functional markers (CD206 and arginine-1 (Arg-1)) were significantly up-regulated in the resistant group (Fig. 1E, F). Immunohistochemical (IHC) and immunofluorescence (IF) staining showed that CD206^+^ TAMs remarkably increased in tumor tissue of the resistant group (Fig. 1G, H). Flow cytometry (FCM) analysis confirmed the higher percentage of CD206^+^/F4/80^+^ macrophage population in sorafenib-resistant mouse HCC tissue (Fig. S3F). To directly determine the impact of M2-TAMs on tumor growth of HCC subjected to sorafenib treatment, we established a xenograft mouse model with human HCC cell SNU739 in the presence or absence of human THP-1-derived M2-TAMs and treated the mice with sorafenib. The tumor volumes derived from SNU739 with THP-1-derived M2-TAMs were significantly larger than those generated from SNU739 alone after sorafenib treatment (Fig. 1I). To further determine the effect of M2-TAMs on the growth of HCC cells upon sorafenib treatment, we measured the IC_50_ of SNU739 and Hepa1-6 exposed to sorafenib (Fig. S4A–D). Conditioned medium (CM) from M2-TAMs was applied to the cell culture to determine the influence of M2 on the cell growth of HCC. Cell viability, cell proliferation, and caspase 3/7 activity assays demonstrated that HCC cells incubated with conditioned medium (CM) from M2-TAMs bore a significantly lower sensitivity to sorafenib (Fig. 1J–L). Taken together, these data indicate that M2-TAM infiltration is correlated with HCC resistance to sorafenib.

### Conditioned medium from Sorafenib-treated HCC cells promotes M2 polarization of TAMs

**Fig. 2:**
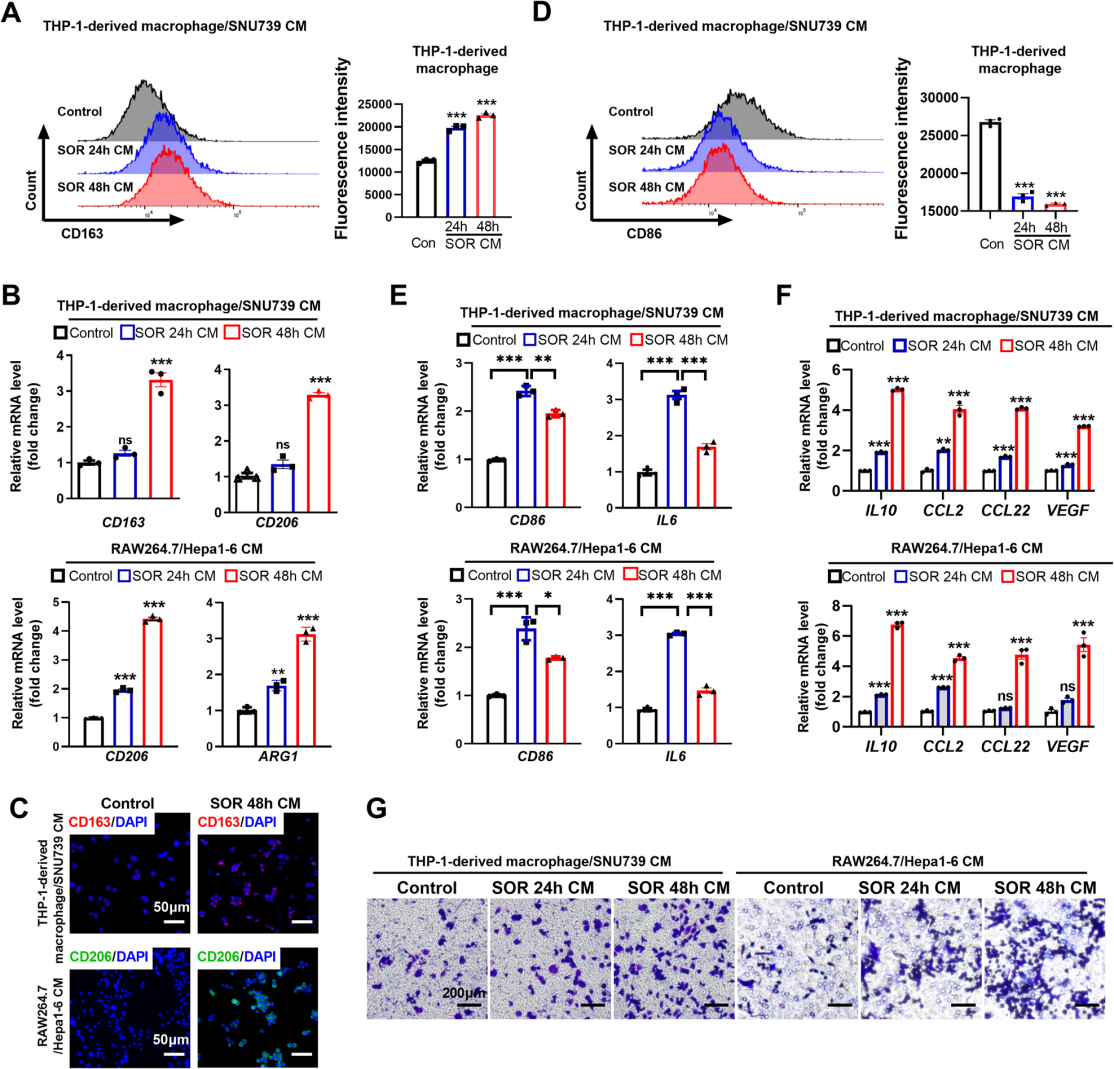
Conditioned medium from Sorafenib-treated HCC cells promotes M2 polarization of TAMs. **A** Flow cytometry analysis of M2-TAM percentage in THP-1-derived macrophages treated with CM from HCC cells. **B** mRNA content of M2-TAM-associated cytokines in THP-1-derived macrophages and RAW264.7 cells treated with CM from HCC cells. **C** Immunofluorescence (IF) analysis of CD163 and CD206 in THP-1-derived macrophages and RAW264.7 cells treated with CM from HCC cells. **D** Flow cytometry analysis of M1-TAM contents in THP-1-derived macrophages treated with CM from HCC cells. **E** qRT-PCR analysis of CD86 and IL6 in THP-1-derived macrophages and RAW264.7 cells treated with CM from HCC cells. **F** qRT-PCR analysis for the mRNA expression of *IL10, CCL2, CCL22* and *VEGF* in THP-1-derived macrophages and RAW264.7 which were treated with CM from HCC cells as indicated. **G** Transwell migration assay of THP-1-derived macrophages and RAW264.7 by CM from HCC cells as indicated. CM, Conditioned medium; SOR, Sorafenib. **P < 0.01; ***P < 0.001.

It is widely accepted that macrophage polarization is regulated by TME. Thus, we hypothesized that sorafenib treatment may increase the release of DAMPs, cytokine and chemokine from HCC cells, leading to the polarization of macrophage in TME. To address the hypothesis, we used a conditioned medium (CM) from human or mouse HCC cells pre-treated with sorafenib to culture human THP-1-derived macrophages or murine RAW264.7 macrophages. CM from human HCC SNU739 cells or murine Hepa1-6 pre-treated with sorafenib strikingly increased the expression of CD163, CD206, or ARG1, which were the typical markers labeling M2-TAMs as compared with the corresponding control group (Fig. 2A–C, Fig. S5A, B). Additionally, CM from HCC cells pre-treated with sorafenib significantly reduced the percentage of M1-TAM (Fig. 2D, E). As shown in Fig. 2F, the mRNA expression of TAM characteristic cytokines *(IL-10, CCL2, CCL22* and *VEGF*) were also substantially increased in macrophages incubated with CM from HCC cells pre-treated with sorafenib. Furthermore, we found that CM from HCC cells pre-treated with sorafenib increased macrophage infiltration in the in vitro migration assay (Fig. 2G).

### Sorafenib promotes extracellular release of mitochondrial DNA from HCC cells by induction of apoptosis

**Fig. 3:**
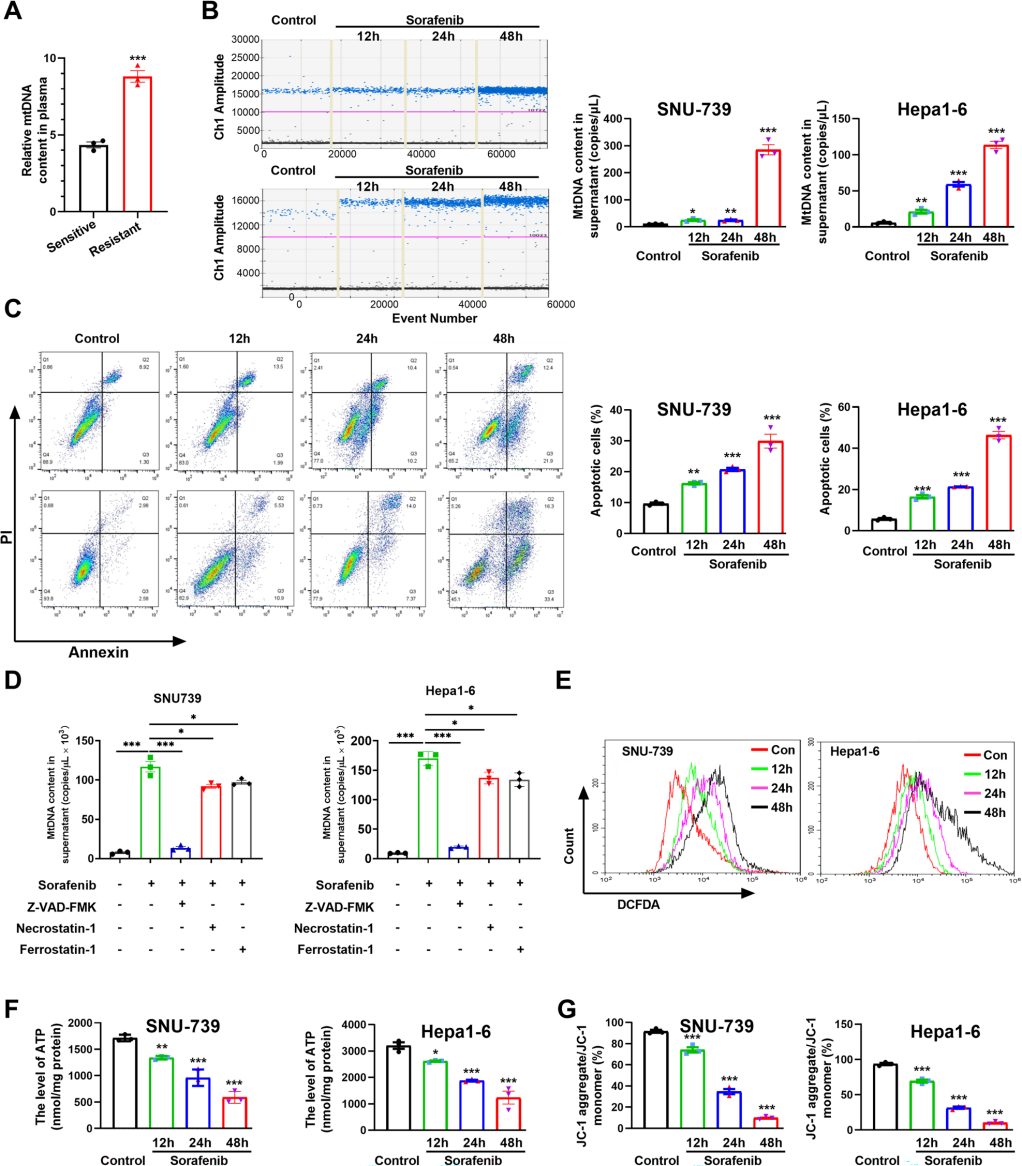
Sorafenib promotes extracellular release of mitochondrial DNA from HCC cells by induction of apoptosis. **A** Relative mtDNA content in plasma from HCC orthotopic mouse model was detected by qPCR. **B** Cell free mtDNA content in supernatant of HCC cells treatment with sorafenib was quantitated via ddPCR. **C** The apoptosis level of HCC cells treated as indicated were detected by flow cytometry. **D** Relative mtDNA content in the supernatant of HCC cells treated with inhibitors of apoptosis, necroptosis and ferroptosis. **E** ROS in HCC cells were detected by flow cytometry. **F**, **G** The ATP levels (**F**) and mitochondrial membrane potential (**G**) of HCC cells treated as indicated were determined using a commercial kit. *P < 0.05; **P < 0.01; ***P < 0.001.

Circulating cell-free mtDNA content is significantly elevated and is identified as a biomarker for HCC and other cancers [28, 31]. We detected the circulating mtDNA level in the plasma from orthotopic HCC mice. An elevated level of circulating mtDNA in sorafenib-resistant mice was observed when compared to that in sorafenib-sensitive mice (Fig. 3A). The cell-free mtDNA content was also investigated in the supernatant from human and murine HCC cells treated with sorafenib by droplet digital PCR (ddPCR). A significantly increased content of mtDNA in a time-dependent manner was identified in cells exposed to sorafenib (Fig. 3B). Interestingly, we found that the circulating mtDNA was oxidized after treatment with sorafenib in HCC cells (Fig. S6A). To ascertain the origin of cell-free mtDNA detected in the supernatant of sorafenib-treated cells, we explored the mitochondrial function and apoptosis of HCC cells. A marked apoptosis occurred in human and murine HCC cells after sorafenib treatment (Fig. 3C and Fig. S6B). Furthermore, we used the inhibitor for apoptosis, necroptosis and ferroptosis to investigate the type(s) of cell death involved in circulating mtDNA release. Our results indicated that the circulating mtDNA content induced by sorafenib was significantly inhibited after treatment with Z-VAD-FMK, an apoptosis inhibitor. Furthermore, the necroptosis inhibitor Necrostatin-1 and the ferroptosis inhibitor Ferrostatin-1 partially mitigated the release of circulating mtDNA in the supernatant of HCC cells with sorafenib treatment. These results demonstrated that the circulating mtDNA was predominantly mediated by sorafenib-induced apoptosis of HCC cells (Fig. 3D). Increased ROS, decreased ATP, and loss of mitochondrial membrane potential were observed in human and murine HCC cells exposed to sorafenib (Figs. 3E–G and S6C). These data suggest that sorafenib induces extracellular release of mitochondrial DNA by inducing mitochondrial dysfunction and apoptosis.

### Mitochondrial DNA promotes M2 polarization of TAMs in vitro

**Fig. 4:**
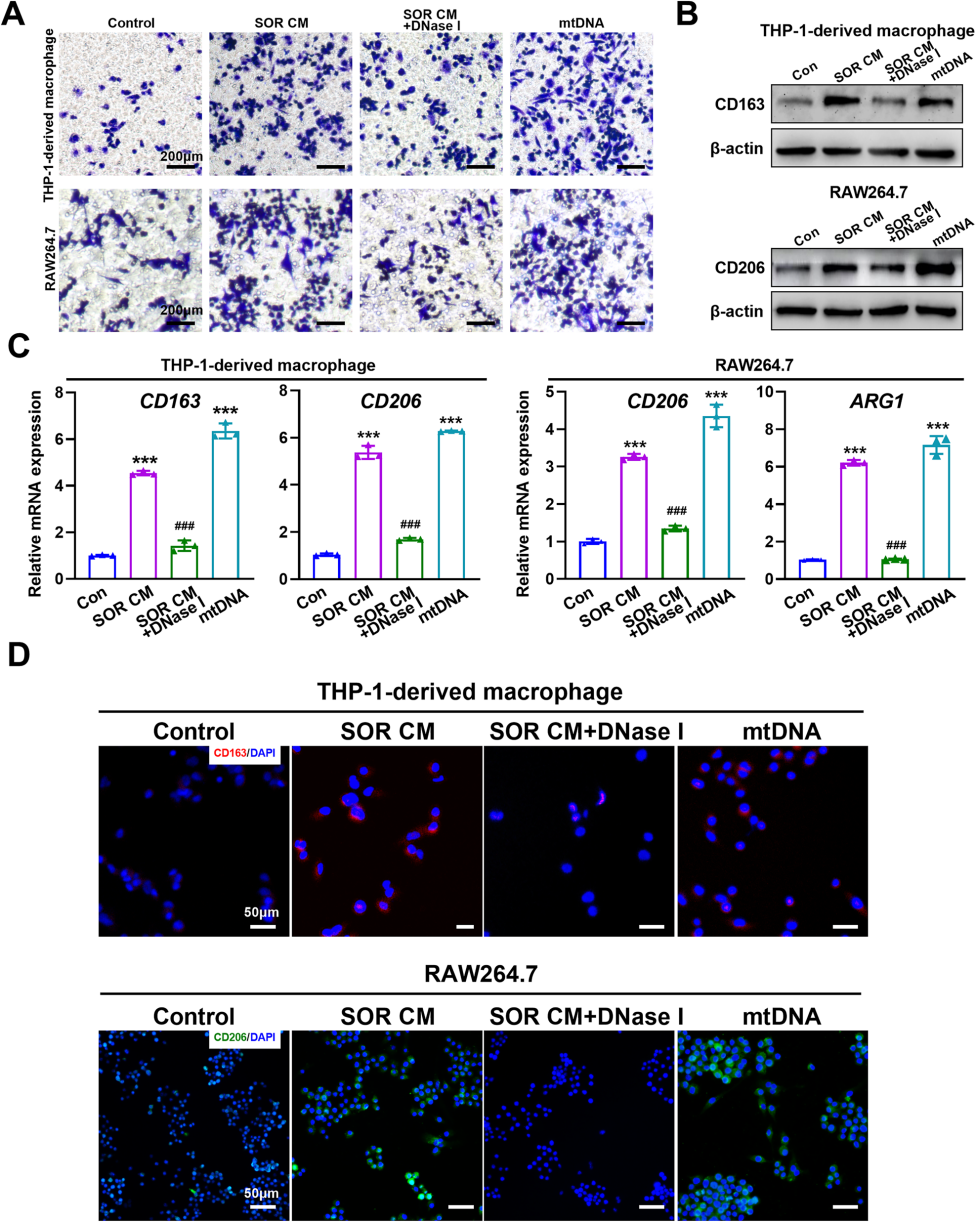
Mitochondrial DNA promotes M2 polarization of TAMs. **A** Transwell migration assay of THP-1-derived macrophages and RAW264.7 treated as indicated. **B**, **C** Western blot (**B**) and qRT-PCR (**C**) analysis the expression levels of M2-TAM marker in THP-1-derived macrophages and RAW264.7 treated as indicated. **D** Immunofluorescence (IF) analysis the expression levels of CD163 and CD206 in THP-1-derived macrophages and RAW264.7 treated as indicated. SOR CM, Conditioned medium from HCC cells treated with sorafenib. ***P < 0.001 versus control group; ^##^*P* < 0.01; ^###^*P* < 0.001 versus SOR CM treated group.

To determine whether cell-free mtDNA is implicated in the M2 polarization of macrophages, THP-1-derived macrophages were incubated with nuclear DNA (nDNA) and mtDNA from human HCC cells. As shown in Fig. S7A, no cellular morphology change in THP-1-derived macrophages was observed after incubation with nDNA. In contrast, the morphology of THP-1-derived macrophages incubated with mtDNA displayed an irregular shape, such as a long spindle-shape and protrusions around the cell membrane, which is similar to positive control (Fig. S7A and Video 1). Furthermore, cell migration assay showed that mtDNA isolated from HCC cells indeed induced macrophage infiltration. Similarly, CM from sorafenib-treated cells enhanced the migration of macrophages, which was abrogated with the treatment of DNase I which digests DNA in the CM (Fig. 4A). Consistently, both CM from sorafenib-treated cells and mtDNA alone were able to up-regulate the expression of CD163 and CD206 in macrophages (Fig. 4B, C and Fig. S8B-E). Application of DNase I markedly inhibited the expression of CM-induced M2-TAM markers, such as CD163, CD206 and Arg-1 in the WB, RT-qPCR, and FCM assays (Figs. 4B, C and S8B–E). Cytokines, such as IL-10, CCL2, CCL22, and VEGF, typically secreted by M2-TAMs, were significantly up-regulated in THP-1-derived macrophages incubated with mtDNA from human HCC cells (Fig. S8F). In addition, immunofluorescent staining demonstrated that digestion of DNAs in CM from sorafenib-treated cells with DNase I reversed CD163 and CD206 expression in THP-1-derived macrophages and RAW264.7 macrophages, respectively (Fig. 4D). These results demonstrate that mtDNA from HCC cells induces macrophage polarization toward a M2-like phenotype.

### Mitochondrial DNA promotes M2 polarization of TAMs and HCC proliferation in mice

**Fig. 5:**
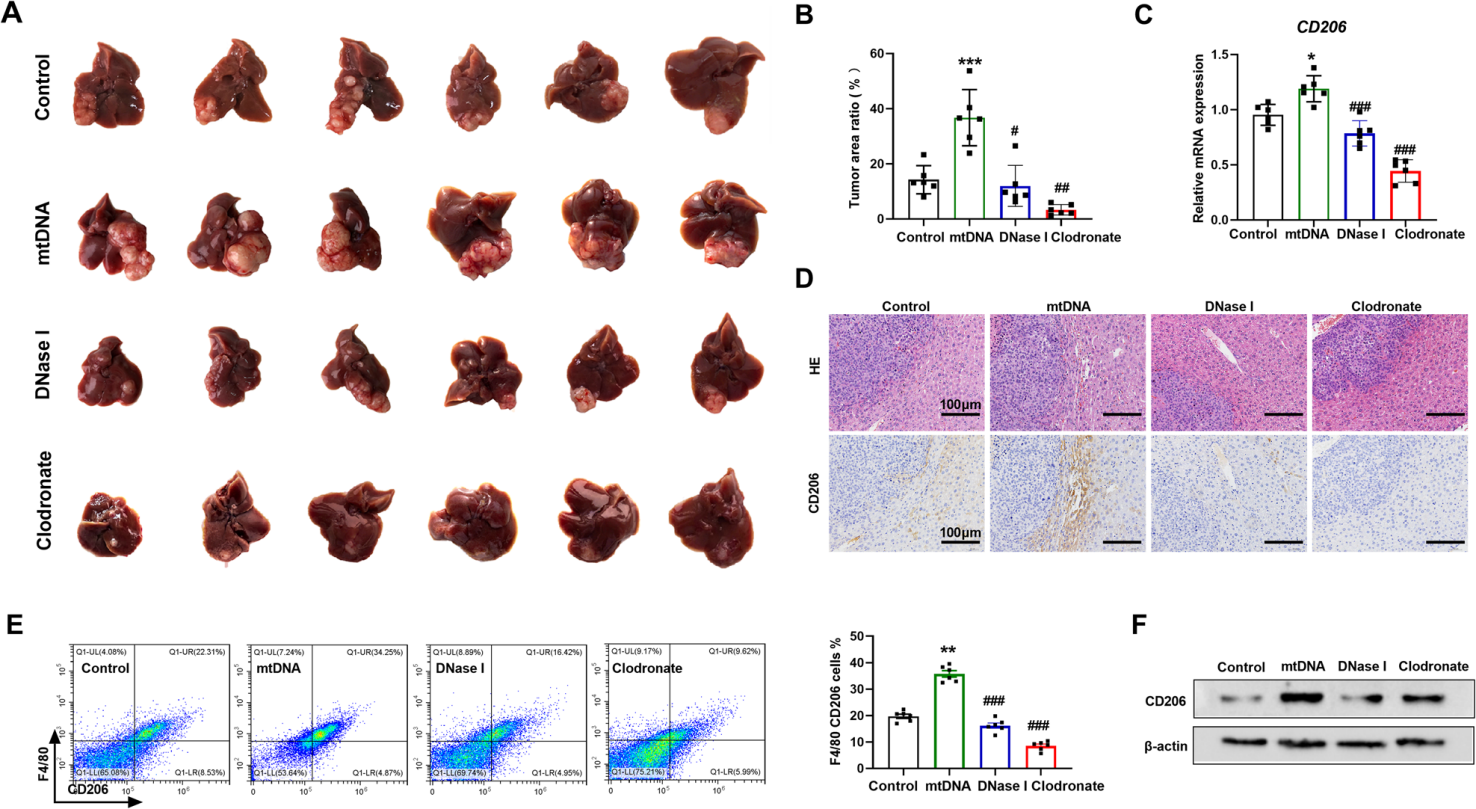
Mitochondrial DNA promotes M2 polarization of TAMs and HCC proliferation in mice. **A** Representative images of orthotopic HCC tissues treated as indicated (*n* = 6). **B** The ratio of mouse liver tumors to liver area was detected in individuals of A (*n* = 6). **C** qRT-PCR analysis of the expression levels of CD206 in orthotopic HCC tissues treated as indicated (*n* = 6). **D** Representative H&E staining (upper) and CD206 immunohistochemistry (under) images of orthotopic HCC tissues treated as indicated. Scale bar: 100 μm. **E** The percentage of CD206 positive macrophage in orthotopic HCC tissues was investigated by flow cytometry (*n* = 6). mtDNA, mice were injected with mtDNA. DNase I, mice were injected with mtDNA and DNase I. Clodronate, mice were injected mtDNA and with clodronate. **F** Western blot analysis of CD206 in orthotopic HCC tissues in mice treated as indicated. *P < 0.05; **P < 0.01 versus control group; ^##^*P* < 0.01; ^###^*P* < 0.001 versus mtDNA treated group.

To further determine the role of mtDNA and macrophage in liver cancer progression, the mouse tumor model was established by orthotopic implantation of Hepa1-6 cells. We found that application of mtDNA promoted tumor growth and increased tumor area ratio (Fig. 5A, B). Treatment with DNase I or depletion of macrophages using clodronate liposomes obviously inhibited live orthotopic tumor growth and reduced tumor area ratio (Fig. 5A, B). MtDNA up-regulated the expression of CD206 in tumor and adjacent tissues, which was ameliorated following the treatment of mice with DNase I or clodronate liposomes (Fig. 5C and F). IHC and FCM indicated an elevated level of CD206^+^ macrophage infiltration in tumor-adjacent tissues after mtDNA treatment (Fig. 5D, E). However, DNase I or clodronate liposomes reduced the CD206^+^ macrophage ratio in tumor-adjacent tissues (Fig. 5D, E). Together, these data suggested that mtDNA promotes M2 polarization of TAMs and HCC proliferation in vivo.

### Mitochondrial DNA promotes M2 polarization of TAMs through activating TLR9

**Fig. 6:**
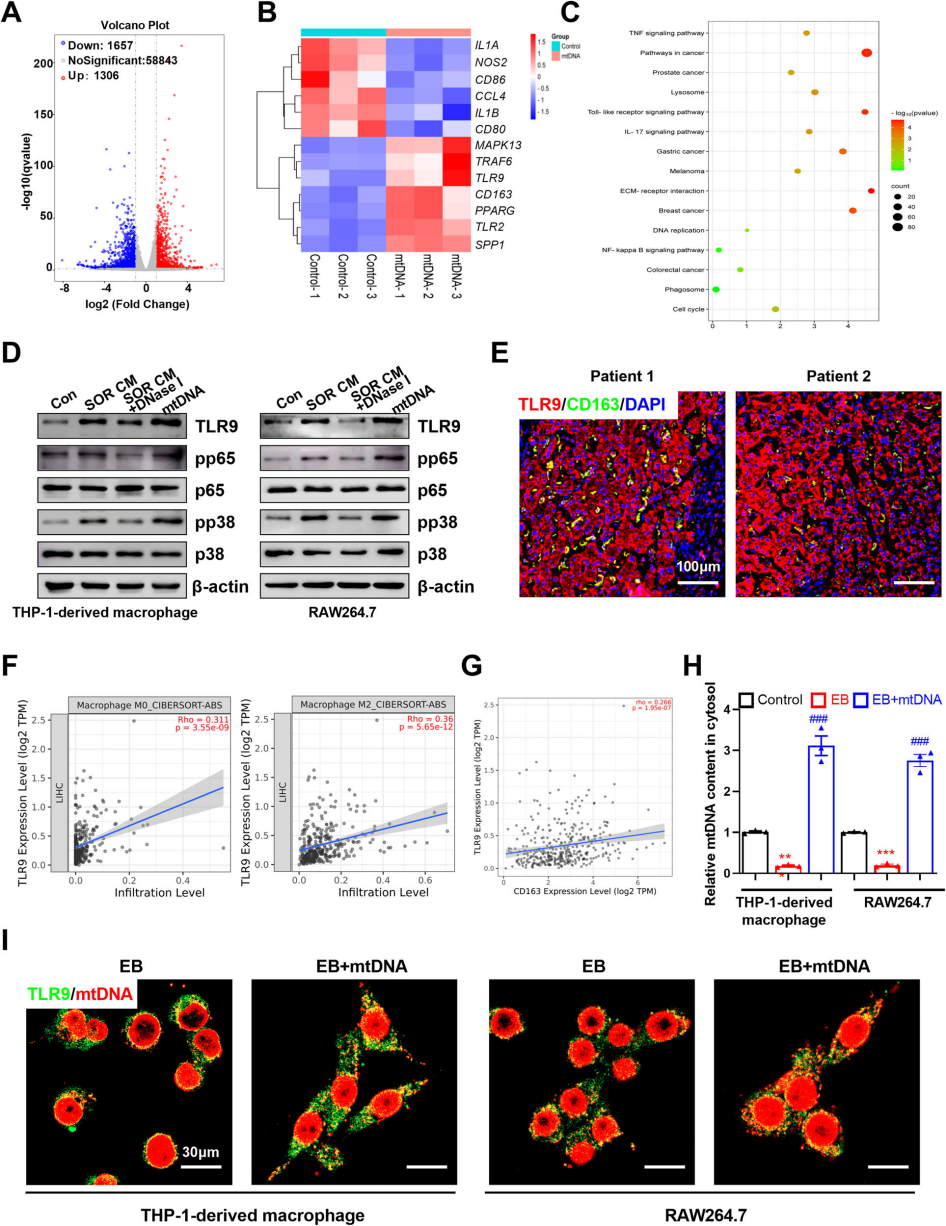
Mitochondrial DNA promotes M2 polarization of TAMs through activating TLR9. **A** Differential mRNA expression in RNA sequencing data of mtDNA-induced M2 macrophages. **B** Heatmap analysis the expression of marker for M1/M2 macrophages in mtDNA induced M2 macrophages. **C** KEGG pathway enrichment analyses of differentially expressed genes were performed. **D** Western blot analysis the related protein expression levels of TLR9 pathway in THP-1-derived macrophages and RAW264.7 treated as indicated. **E** Immunofluorescence staining of TLR9 and CD163 in tumor tissues from patients with HCC. Scale bar: 100 μm. **F**, **G** CIBERSORT analysis the correlated between TLR9 expression and M2 macrophage infiltration level (**F**) or CD163 expression level (**G**). **H** Relative mtDNA content in THP-1-derived macrophages and RAW264.7 treated as indicated was detected by qPCR. **I** Co-localization analyses between mtDNA and TLR9 in indicated THP-1-derived macrophages and RAW264.7 cells by confocal microscopy. Scale bar: 30 μm.

To explore the molecular mechanisms underlying mtDNA-mediated M2 polarization, we performed an RNA sequencing using THP-1-derived macrophages incubated with mtDNA. Transcriptome profiling identified 1306 up-regulated and 1657 downregulated genes after mtDNA treatment (Supplementary Table S4). *IL-1B*, *CD86*, and *NOS2*, markers of M1 macrophages, were notably downregulated after mtDNA treatment (Fig. 6A, B). Whereas, the M2 macrophage markers were significantly up-regulated after mtDNA treatment (Fig. 6A, B). It has been demonstrated that mtDNA contains unmethylated CpG motifs and is a potent activator of toll-like receptor 9 (TLR9). TLR signaling pathway, NF-κB signaling pathway, and pathways in cancer were enriched in the KEGG pathway analysis of differentially expressed genes (Fig. 6C). Due to the critical role that TLR9 plays in the response to mtDNA [32], we speculated that the TLR9 pathway may be implicated in mtDNA-mediated M2 polarization of macrophages. Indeed, the expression of TLR9 and TRAF6, downstream targets of the TLR9 signaling, were increased after CM from HCC cells pre-treated with sorafenib and mtDNA treatment (Fig. 6B and D). Our data showed that CM from HCC cells pre-treated with sorafenib and mtDNA significantly activated the TLR9 pathway in THP-1-derived macrophage or RAW264.7, respectively (Fig. 6D). Digestion mtDNA using DNase I reversed TLR9 pathway activation (Fig. 6D). Further, we found that the expression of TLR9 was markedly up-regulated in tumor tissues from HCC patients (Fig. S3A, S3D and S3E). Immunofluorescence co-localization analysis showed that TLR9 was co-localized with CD163^+^ macrophages in tumor tissues from HCC patients (Fig. 6E). CIBERSORT analysis indicated a moderate positive correlation between the mRNA expression levels of TLR9 and the infiltration levels of M2 macrophages (r = 0.36, *P* < 0.001), as well as between the mRNA expression levels of TLR9 and CD163 (r = 0.266, *P* < 0.001) (Fig. 6F, G). To determine the consequences of mtDNA from HCC cells on THP-1 cells and RAW264.7, endogenous mtDNAs of THP-1 cells and RAW264.7 were depleted using ethidium bromide (EtBr) as previous description [33, 34]. As shown in Fig. 6H, the mtDNA contents of THP-1-derived macrophages and RAW264.7 were obviously decreased after treatment with 50 ng/mL EtBr for 4 weeks. Immunofluorescent staining and qRT-PCR assay showed that exogenous mtDNA from HCC cells increased cytosolic mtDNA content and colocalized with TLR9 in THP-1-derived macrophages and RAW264.7 (Fig. 6H, I).

### Inhibition of TLR9 pathway reverses mtDNA-mediated M2 polarization of TAMs

**Fig. 7:**
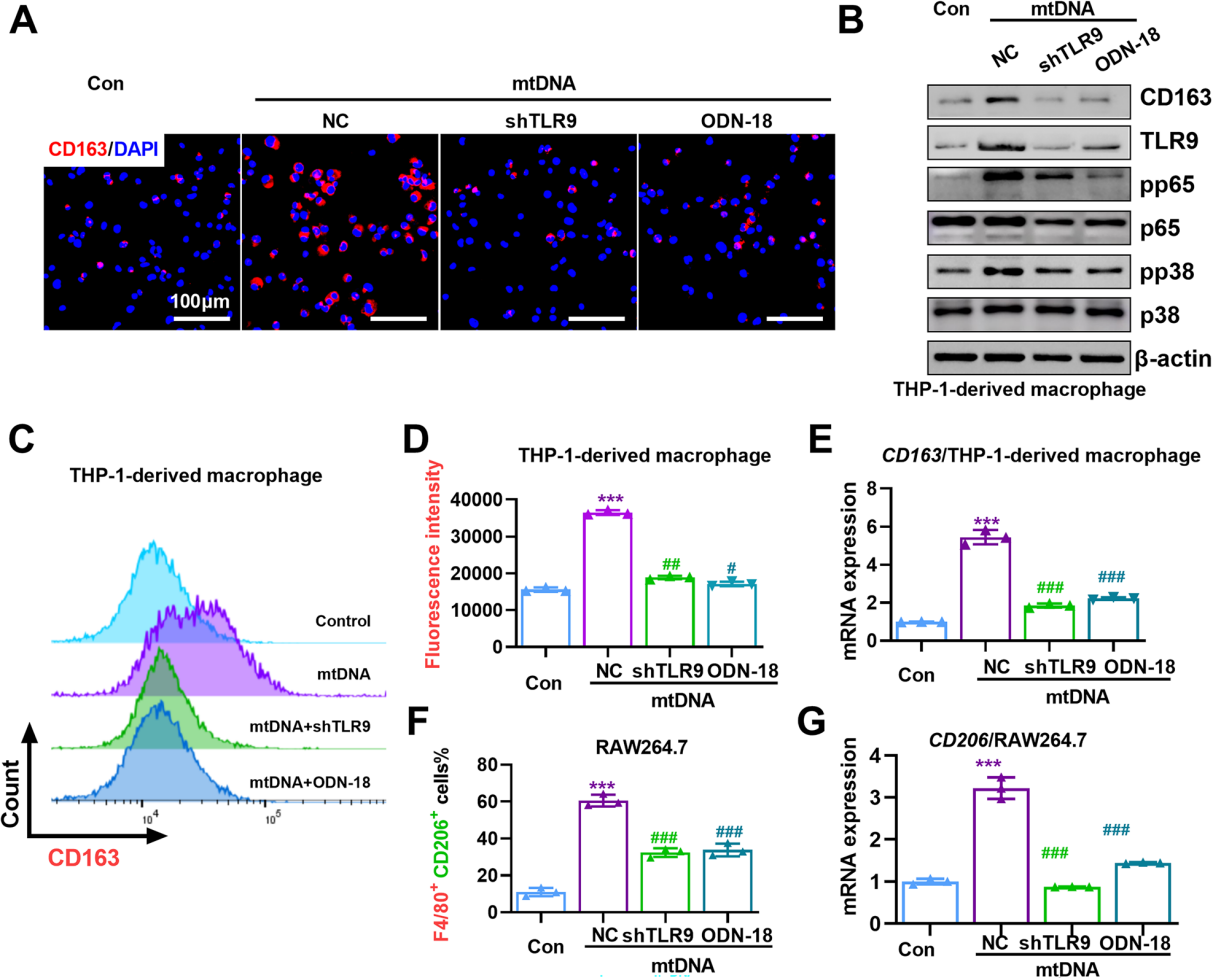
Inhibition of TLR9 pathway reverses mtDNA-mediated M2 polarization of TAMs. **A** Immunofluorescence (IF) analysis the expression levels of CD163 in THP-1-derived macrophages treated as indicated. **B** Western blot analysis the expression levels of CD163 and TLR9 pathway-related protein in THP-1-derived macrophages treated as indicated. **C** Flow cytometry analysis the percentage of CD163^+^ cells in THP-1-derived macrophages. **D** The percentage of CD163^+^ macrophage in C was statistical analysis, (*n* = 3). **E** qRT-PCR analysis of CD163^+^ in THP-1-derived macrophages treated as indicated. **F** The percentage of F4/80^+^/CD206^+^ macrophage in Fig. S9B was statistical analysis. (*n* = 3). **G** qRT-PCR analysis the expression levels of M2-TAM marker in THP-1-derived macrophages and RAW264.7 treated as indicated. ***P < 0.001 versus control group; ^###^*P* < 0.001 versus mtDNA treated group. Con, M0 macrophage; NC, M0 macrophage treated with mtDNA; shTLR9, M0 macrophage treated with mtDNA and shTLR9; ODN-18, M0 macrophage treated with mtDNA and ODN INH-18.

Next, we sought to explore whether activation of the TLR9 pathway was involved in mtDNA-mediated M2 polarization of TAMs. CIBERSORT analysis indicated that TLR9 expression level was positively correlated with *CCL2, CCL17, CCL18, CCL20, CCL22, IL6, TGFB1, VEGFA, IL-10*, which are vital cytokines for M2 macrophage function (Fig. S9A). Immunofluorescent staining showed that TLR9 antagonist (ODN INH-18) treatment or depletion of TLR9 by shRNA abolished mtDNA-induced CD163 expression in THP-1-derived macrophages (Fig. 7A, B). Similarly, FCM assay and RT-qPCR verified that ODN INH-18 and TLR9 shRNA inhibited mtDNA-induced expression of M2-TAM markers (CD163 and CD206) in THP-1-derived macrophages and RAW264.7 (Figs. 7C–G and S9B). Collectively, these data suggest that mtDNA activates TLR9 signaling and promotes macrophage polarization toward M2-like phenotype.

### Blocking TLR9 pathway enhances the therapeutic effect of sorafenib in HCC

**Fig. 8:**
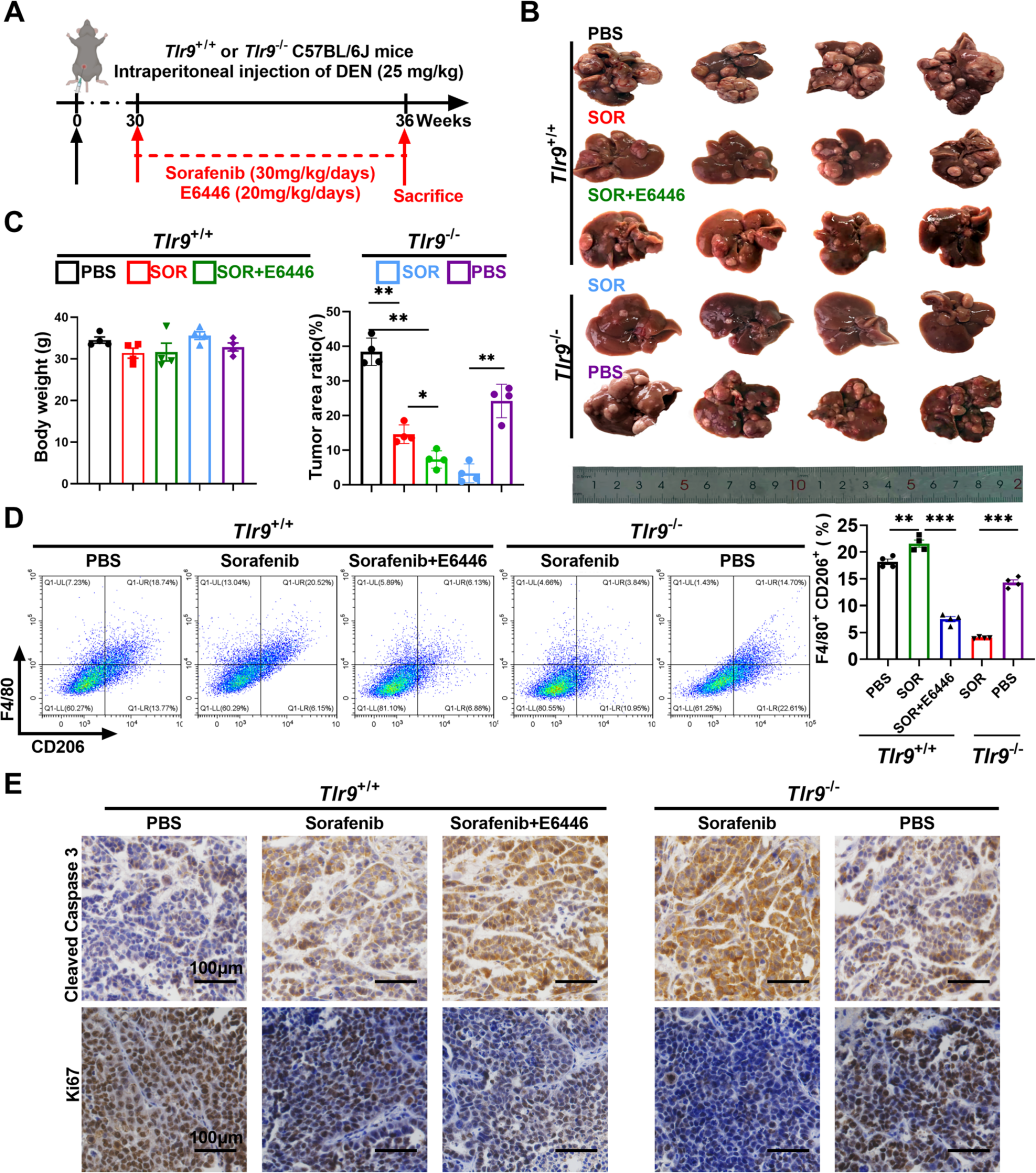
Blocking TLR9 pathway enhances the therapeutic effect of sorafenib in HCC. **A** Schematic description of sorafenib and E6446 treatment in the DEN-induced HCC model (*n* = 4). **B** Representative micrographs of DEN-induced HCC treated as indicated (*n* = 4). **C** Body weight and the ratio of mouse liver tumors to liver area were detected in individuals of B (*n* = 4). **D** Flow cytometry analysis the macrophage infiltration in the DEN-induced HCC tissues treated as indicated (*n* = 4). **E** Representative immunohistochemical image of cleaved caspase-3 and Ki-67 in the DEN-induced HCC tissues treated as indicated, Scale bar: 100 μm.

To determine the combined therapeutic effect of blocking TLR9 pathway and sorafenib, we established a DEN-induced HCC model in wild-type (*Tlr9*^+/+^) and *Tlr9* knockout (*Tlr9*^-/-^) mice and treated the mice with or without sorafenib (Fig. 8A). Sorafenib alone inhibited tumor progression in wild-type (*Tlr9*^+/+^) mice and E6446 (TLR9 inhibitor) significantly enhanced the therapeutic effect of sorafenib. Combination treatment with sorafenib and E6446 exhibited a more marked regression compared to the sorafenib alone group (Fig. 8B, C). Furthermore, the analysis of M2 macrophage percentage in the tumor tissue from DEN-induced HCC models showed that E6446 combined with sorafenib treatment significantly reduced M2 macrophage infiltration in the tumor tissues compared to sorafenib alone group (Fig. 8D). IHC analysis was performed to observe the Ki67 and cleaved caspase-3 expression in isolated tumor tissues. The levels of Ki67 expression was significantly lower and the level of cleaved caspase-3 expression was higher in the combination group (Fig. 8E). These data suggest that sorafenib and E6446 combination therapy effectively suppresses the proliferation and promotes apoptosis of tumor cells. As expected, TLR9 knockout exhibited a similar effect with E6446 (Fig. 8B–E). Furthermore, we found that there was no significant difference in circulating mtDNA content between WT and TLR9^-/-^ mice treated with sorafenib (Fig. S10). These results indicate that blocking TLR9 pathway is an ideal strategy to enhance the therapeutic effect of sorafenib in HCC.

## Discussion

**Fig. 9:**
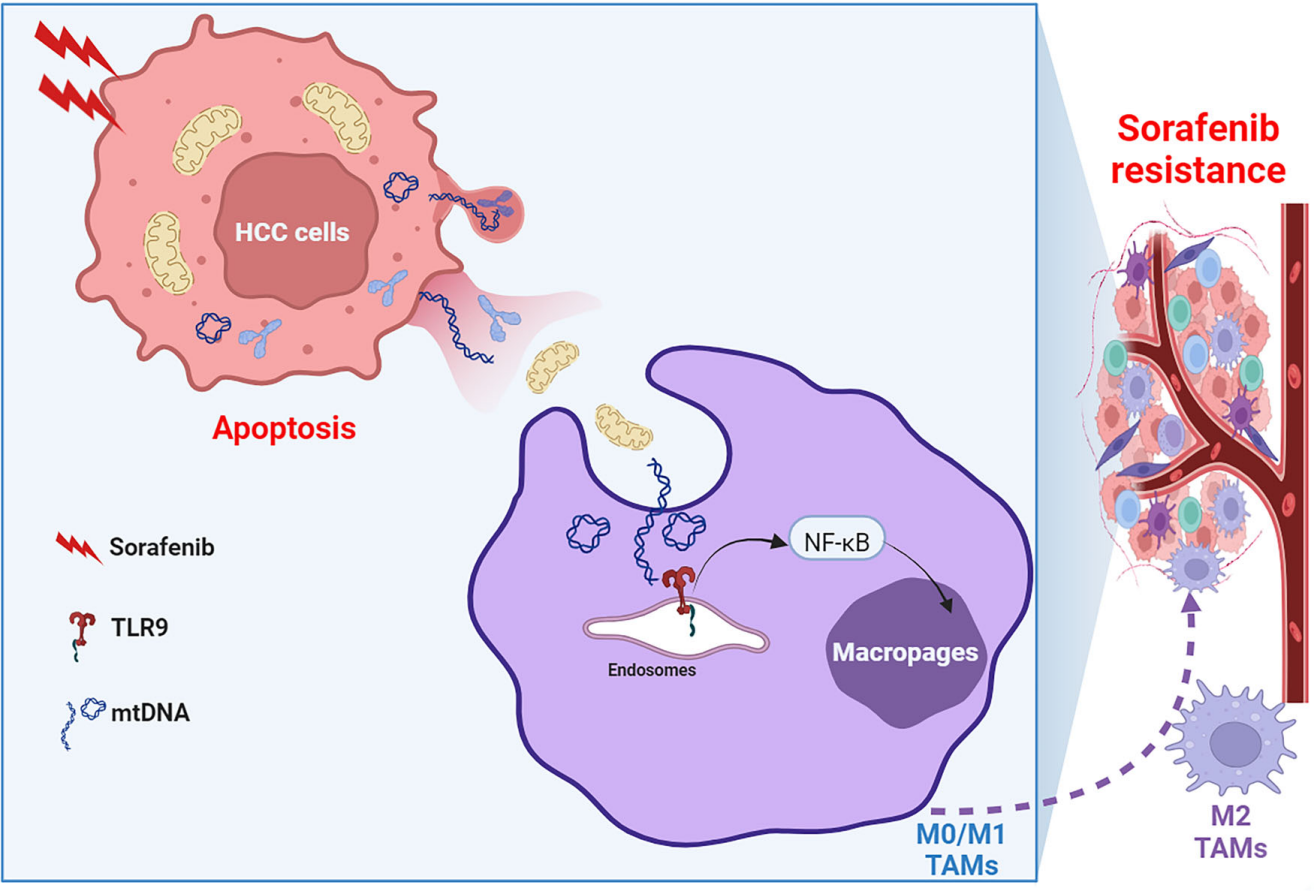
Schematic depicting the mitochondrial DNA promotes M2 polarization of tumor associated macrophages and HCC resistance to sorafenib through TLR9 pathway.

Resistance is a major hurdle to the therapeutic effect of advanced HCC populations after sorafenib treatment [6]. Previous researches have determined that M2-TAM infiltration played a vital role in sorafenib resistance and explored the mechanism underlying macrophage recruitment and M2 polarization to develop novel therapeutic strategies for overcoming resistance [8–10]. In this study, we determined that extracellular cell-free mtDNA induced by sorafenib played a key role in macrophage M2 polarization and mediating sorafenib resistance in HCC. We found that sorafenib induced extracellular release of mtDNA by inducing apoptosis of HCC cells, which subsequently promoted M2 polarization of macrophages through TLR9 pathway. Furthermore, TLR9 knockout or inhibition reversed mtDNA-mediated M2 polarization of macrophages and enhanced the therapeutic effect of sorafenib in HCC (Fig. 9). Our data indicate that blocking mtDNA-induced M2 polarization of TAMs could be a promising therapeutic strategy to overcome sorafenib resistance.

M2-TAMs have been reported to play an important role in tumorigenesis, progression, metastasis and drug resistance of HCC by building the immunosuppressive environment [9]. Consistent with our and other group’s previous studies, we found the infiltration of M2-TAMs was significantly increased in HCC tissues and correlated with poor prognosis and sorafenib resistance using CIBERSORT analysis. Orthotopic HCC mouse model indicated a significantly higher M2-TAM infiltration in sorafenib resistant tumors. The sensitivity of HCC cells incubated with M2-TAMs to sorafenib was significantly lower than control. Furthermore, depletion of macrophages using clodronate liposomes obviously inhibited HCC growth in vivo. Together, our current data demonstrate that M2-TAM infiltration elevates after sorafenib treatment and is correlated with sorafenib resistance.

Circulating cell-free mtDNA content in plasma, serum, or whole blood is significantly elevated and is a biomarker in various cancer types [31]. Our and other group studies have shown that circulating cell-free mtDNA is a risk biomarker in HCC patients receiving TACE and TCM treatment [28, 35]. In the present study, we found that circulating cell-free mtDNA content was significantly elevated in plasma of orthotopic HCC mouse models from sorafenib resistant group. Sorafenib promoted extracellular mitochondrial DNA release of HCC cells by inducing mitochondrial dysfunction and apoptosis. However, the underlying pathophysiological impact on cancer progression of mtDNA is less well known, especially in tumor microenvironment regulation. Our results showed that individual mtDNA from HCC cells induced macrophage recruitment and M2 polarization in vitro. Moreover, results from orthotopic HCC mouse model revealed that mtDNA promoted the infiltration of M2-TAMs and proliferation of HCC. Digest mtDNA with DNase I treatment reduced M2-TAMs ratio in tumor-adjacent tissues and inhibited HCC cell proliferation.

As a specific mitochondrial DAMP, mtDNA is recognized via pattern recognition receptors [24, 25]. RNA sequencing analyses showed that TLR9 pathway was implicated in mtDNA-mediated M2 polarization of macrophages. Various researches identified that TLR9 pathway is frequently activated in solid malignancies, including breast cancer, colorectal cancer and HCC [36]. Our previous study found that TLR9 was up-regulated in HCC tissues and HCC patients with a high TLR9 expression had a significantly poorer overall survival and recurrence-free survival [11]. Interestingly, we found that TLR9 expression was colocalized with CD163^+^ macrophages in tumor tissues from HCC patients. The CIBERSORT analysis showed a positive relationship between TLR9 expression and M2 macrophage infiltration and CD163 expression. Using a parental mtDNA deletion THP-1 cells model, we characterized the role of HCC cell-derived mtDNA in M2 polarization of macrophage and explored the underlying mechanism. Our data showed that mtDNA induced macrophage recruitment and M2 polarization through TLR9 pathway, and inhibition of this pathway reversed mtDNA-mediated M2 polarization of macrophages. TLR9 pathway plays a vital role in macrophage polarization and tumor progression. However, previous studies focused on the role of TLR9 pathway in cancer cells. In this study, we found the TLR9 pathway in tumor microenvironment was also implicated in tumor progression. Blocking TLR9 pathway enhanced the therapeutic effect of sorafenib in HCC by suppressing M2 polarization of macrophages. Therefore, targeting TLR9 might be an excellent strategy to enhance the therapeutic effect of sorafenib for advanced HCC patients.

## Conclusions

In present study, we established the functional role of mtDNA in regulating tumor microenvironment and sorafenib resistance of HCC. Mechanistically, we showed that sorafenib induced mtDNA release into extracelluar matrix and HCC-derived circulating mtDNA protumoral microenvironment remodeling by mediating M2 polarization of macrophages through TLR9 pathway. Specifically, inhibition of mtDNA-induced TLR9 pathway enhanced the therapeutic effect of sorafenib in HCC, and this would be particularly important to allow targeting of the mtDNA-TLR9 pathway as a therapeutic intervention strategy.

## Supplementary information

Supplementary Material

Supplementary for WB

Video

## Data Availability

All data are available in the main text or the Supplementary Materials. Sequencing data have been deposited in GEO under accession No. GSE270323.
