# Supplementary material for: Circulating mitochondrial DNA promotes M2 polarization of tumor associated macrophages and HCC resistance to sorafenib
Source: Cell Death Dis. 2025 Mar 4;16(1):153. doi: 10.1038/s41419-025-07473-8 (PMC11880550; doi:10.1038/s41419-025-07473-8)
Supplement: Supplementary file 1 — Supplementary Material [file 41419_2025_7473_MOESM1_ESM.docx]

**Supplementary Material**

**Circulating mitochondrial DNA promotes M2 polarization of tumor associated macrophages and HCC resistance to sorafenib**

**Supplementary Figure legends**

**Fig. S1 Up-regulated** **M2 tumor associated macrophage infiltration in tumor tissue of HCC patients.**


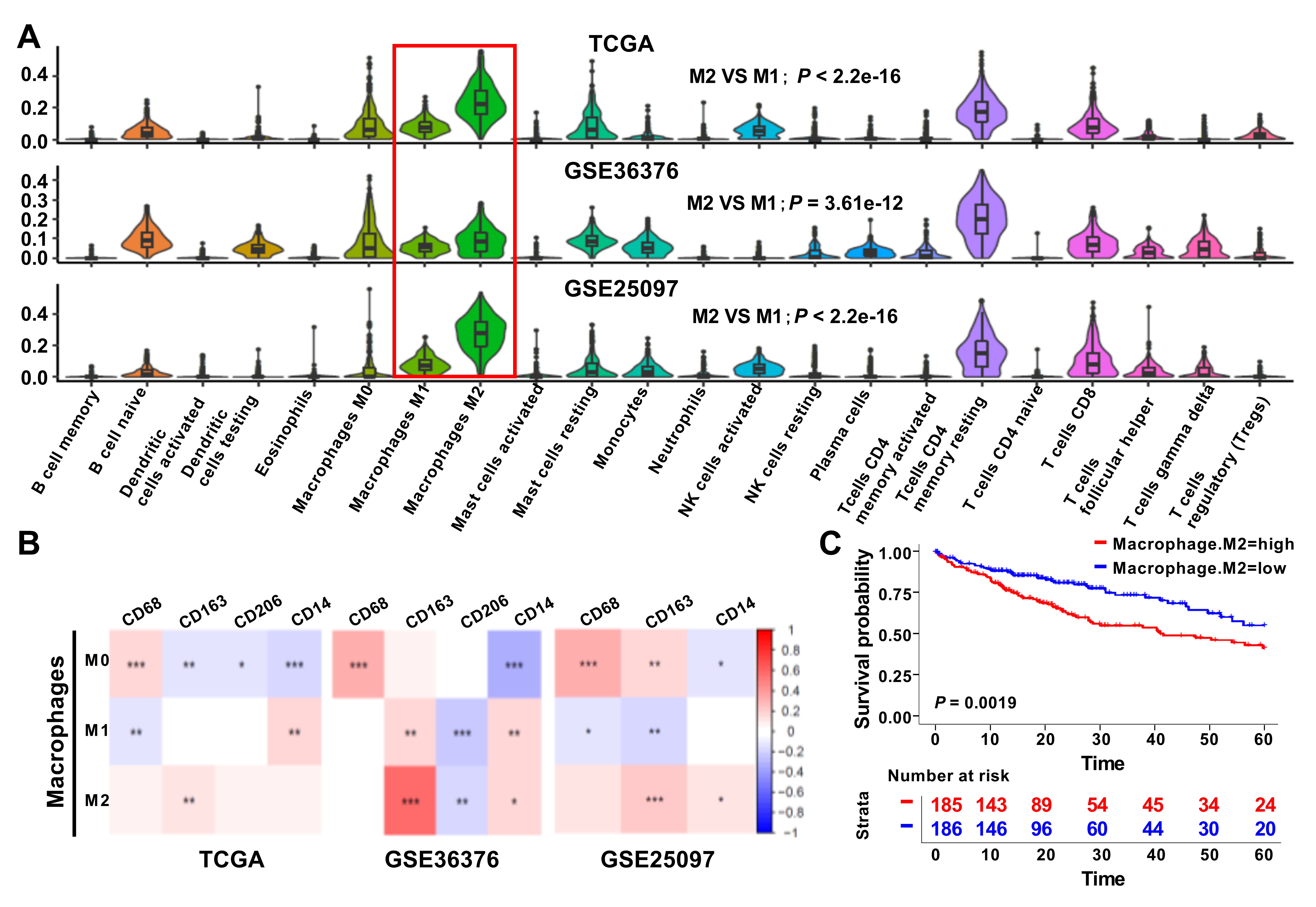


**(A)** CIBERSORT analysis the abundance of tumor-infiltrating immune cells (TIICs) in HCC tissue from TCGA, GSE36376 and GSE25097 dataset. **(B)** The correlated between macrophage abundance and expression levels of M0/M1/M2 markers were evaluated using TCGA, GSE36376 and GSE25097 dataset. **(C)** Kaplan-Meier curve analysis of survival probability based on M2 macrophage abundance in TCGA dataset.

**Fig. S2 M2 tumor associated macrophage infiltration in tumor tissue of HCC patients treatment with sorafenib.**

**
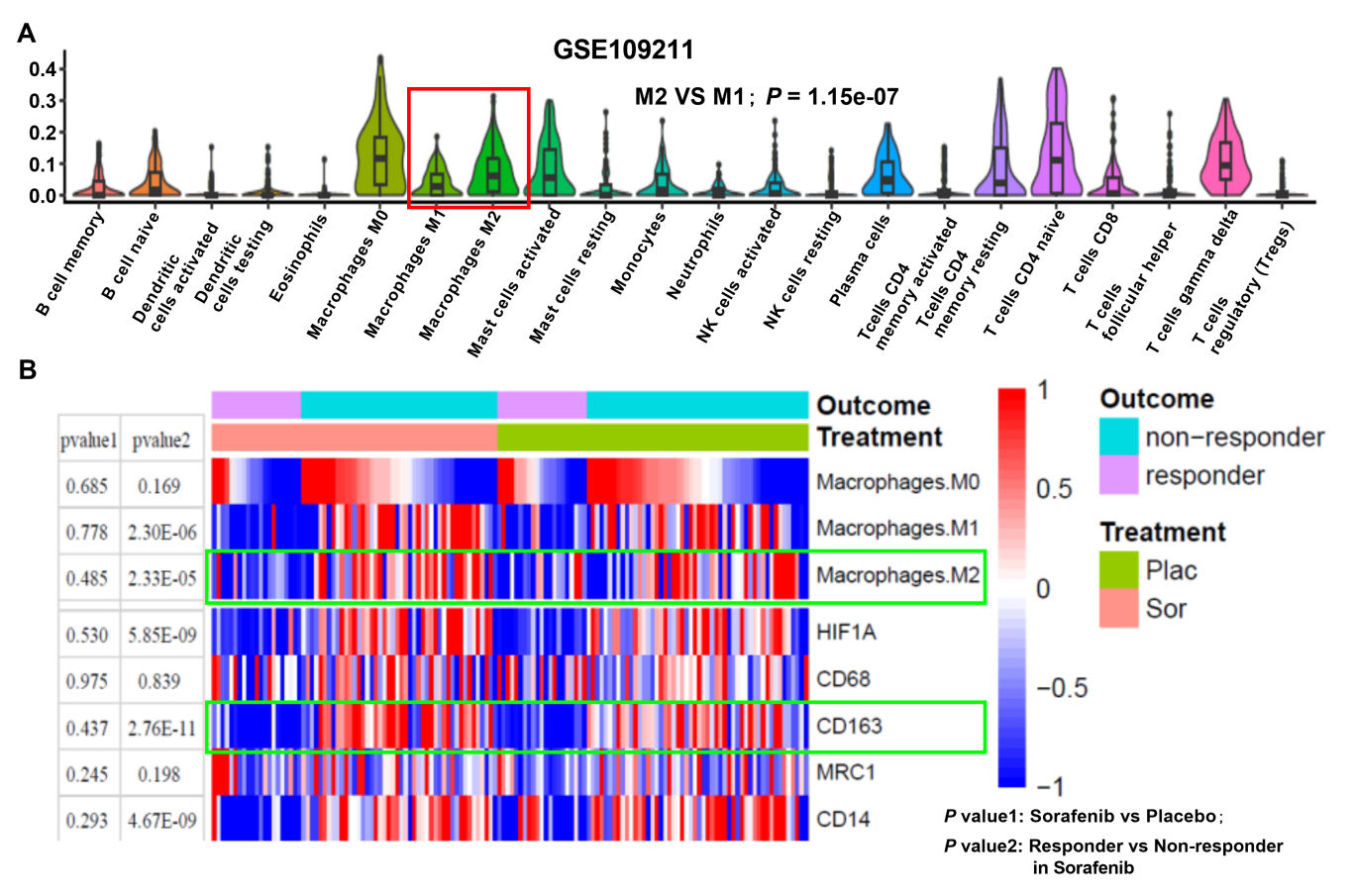
**

**(A)** CIBERSORT analysis the abundance of tumor-infiltrating immune cells (TIICs) in HCC tissue from GSE109211 dataset. **(B)** Comparison of macrophage abundance and M0/M1/M2 marker expression levels between sorafenib responders and non-responders in the GSE109211.

**Fig. S3 Expression of CD163 and TLR9 in human HCC tissues.**

**
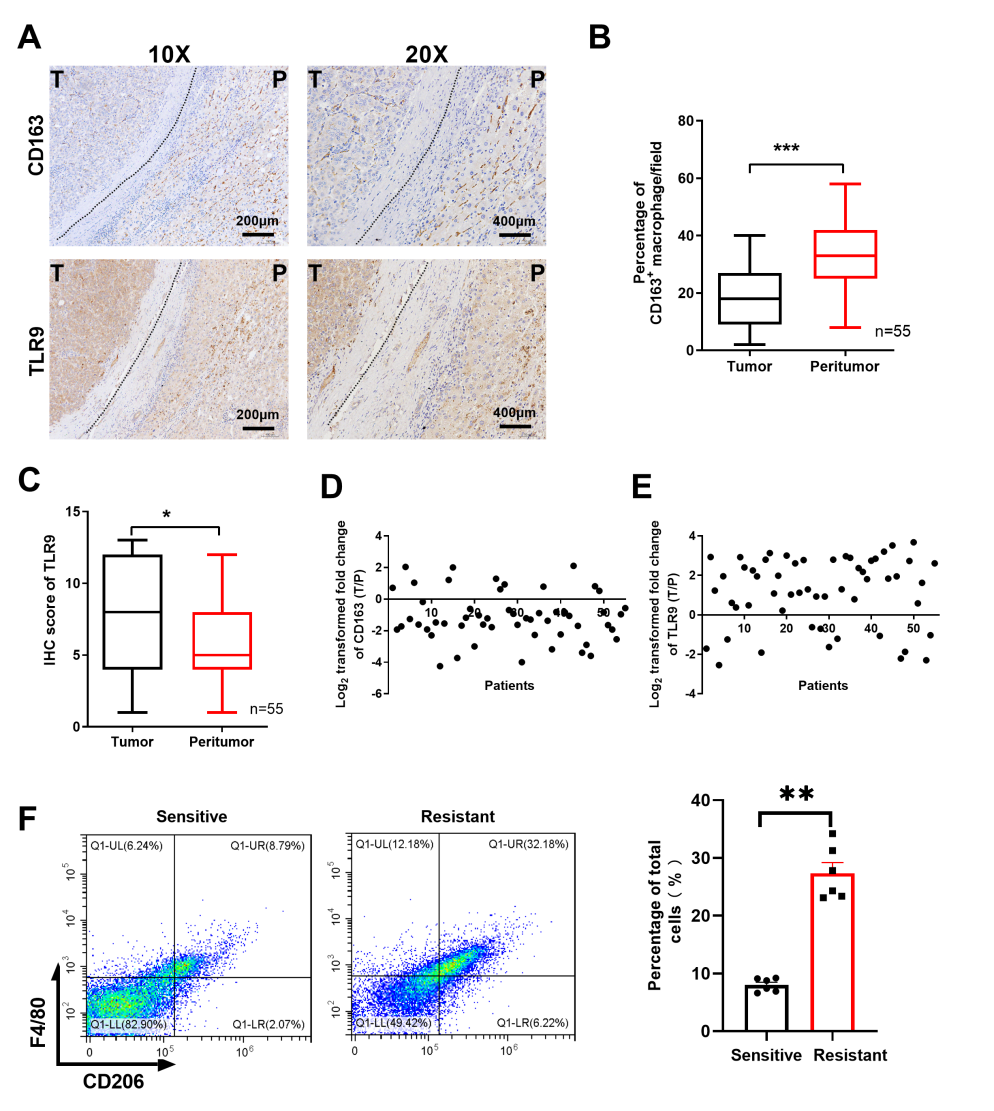
**

**(A)** Representative immunohistochemical (IHC) staining images of CD163 and TLR9 in HCC tissues (n = 55).

**(B)** The percentage of CD163 positive cells in the tissue of HCC patients was statistically analyzed.

**(C)** IHC score of TLR9 in tissue of HCC patients was statistically analyzed.

**(D)** qRT-PCR analysis for the expression of CD163 in 55 paired tissues from HCC patients. The relative expression ratio of tumor to peritumor was log_2_-transformed.

**(E)** qRT-PCR analysis for the expression of TLR9 in 55 paired tissues from HCC patients. The relative expression ratio of tumor to peritumor was log_2_-transformed. T, tumor; P, peritumor.

**(F)** Flow cytometry analysis of F4/80^+^/CD206^+^ macrophages in orthotopic tumor tissue as indicated. *, *P* < 0.05; ***, *P* < 0.001.

**Fig. S4 The 50% inhibitory concentration (IC50) of sorafenib in HCC cells.**

**
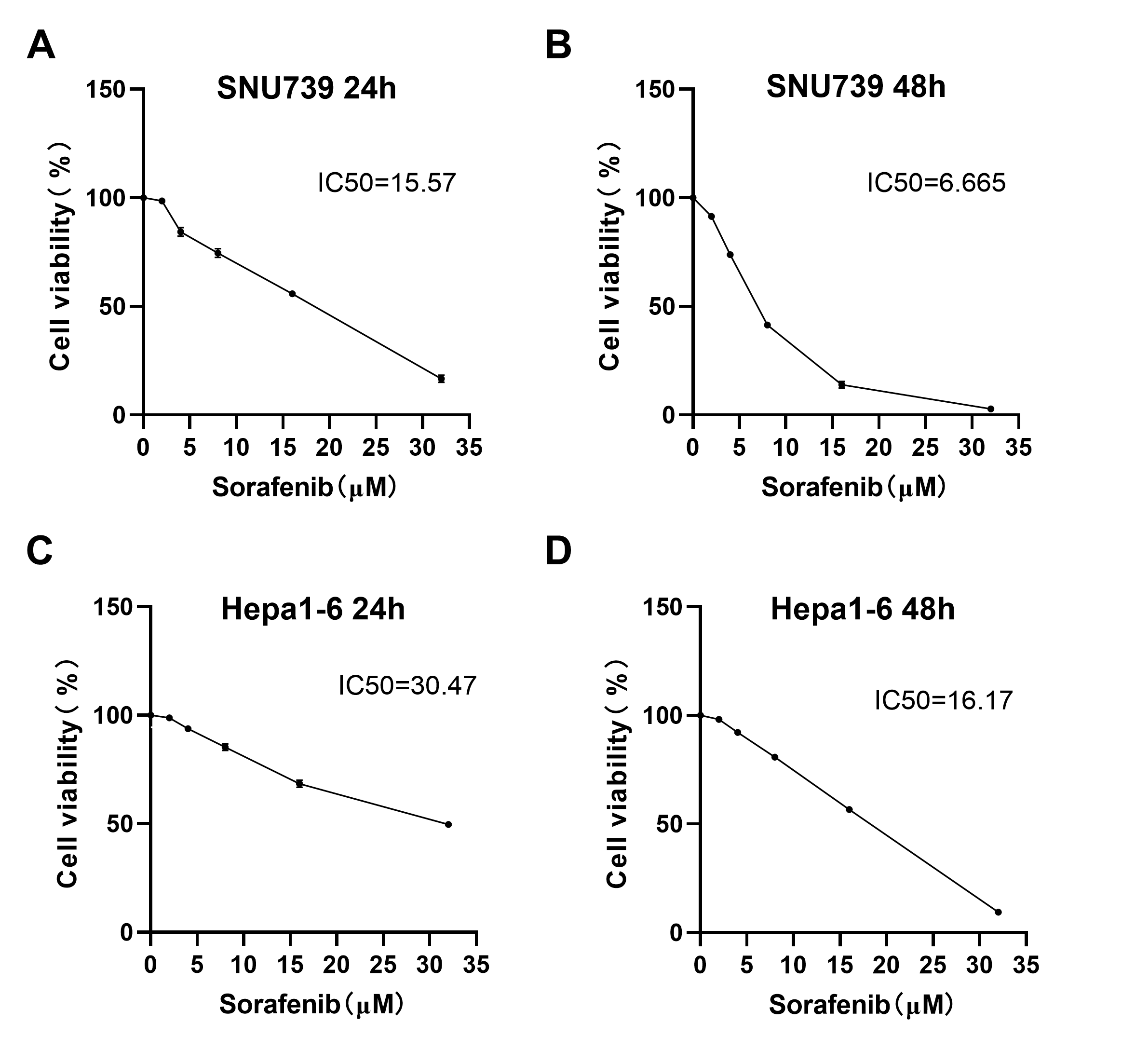
**

**(A and B)** SNU739 cells were treated with the indicated concentrations of sorafenib for 24 h (A) or 48 h (B), viability of cells was assessed by MTT assay. **(C and D)** Hepa1-6 cells were treated with the indicated concentrations of sorafenib for 24 h (C) or 48 h (D), viability of cells was assessed by MTT assay. The 50% inhibitory concentration (IC50) of sorafenib in HCC cells was calculated using a nonlinear regression with normalized dose-response fitting using Prism software (GraphPad Software, Prism 8).

**Fig. S5 Sorafenib induces the polarization of M2 macrophages.**


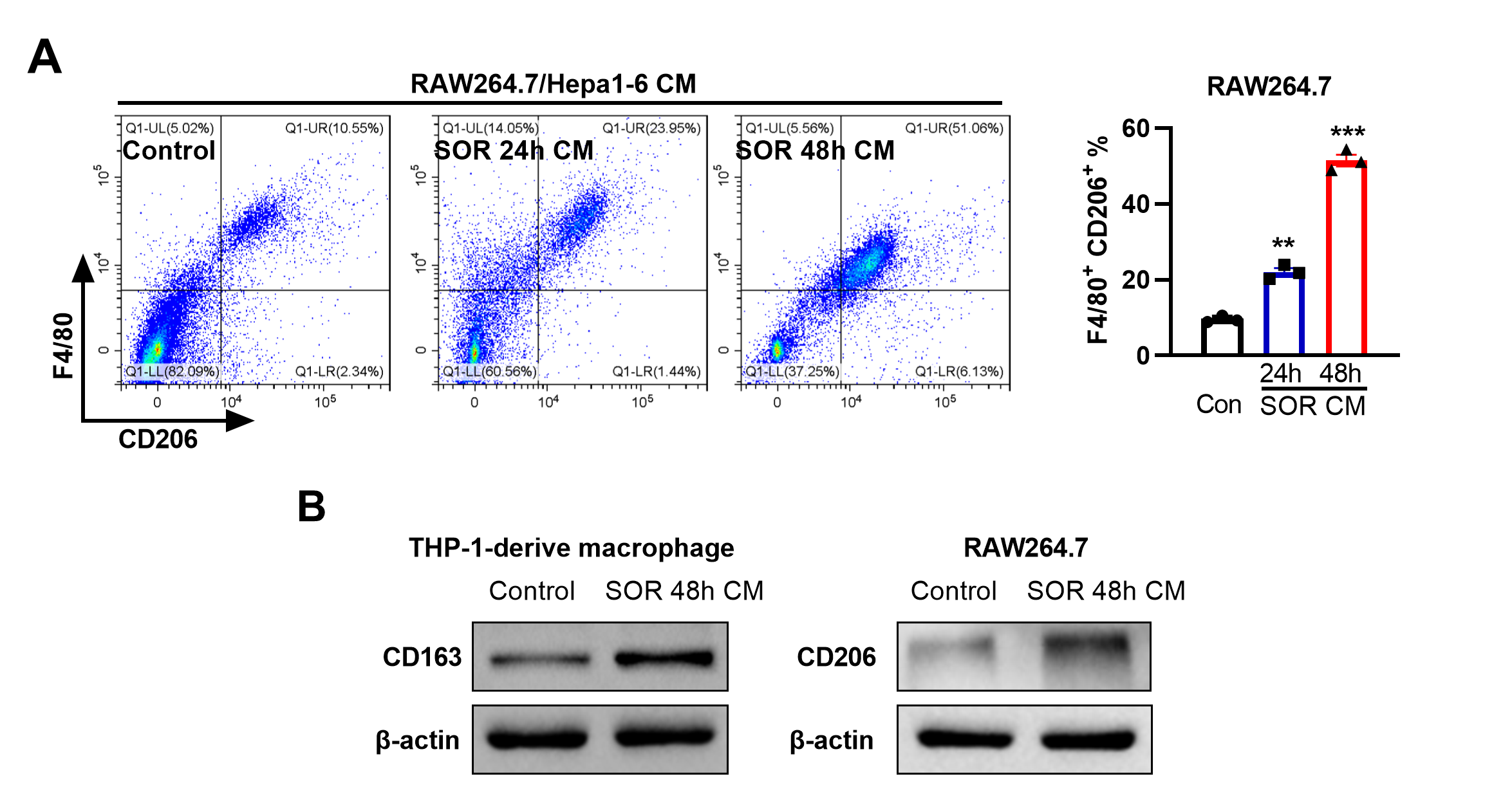


**(A)** Flow cytometry analysis of F4/80^+^/CD206^+^ cell percentage in RAW264.7 cells treated with CM from HCC cells.

**(B)** Western blot analysis of CD163 and CD206 in THP-1-derived macrophages and RAW264.7 cells treated with CM from HCC cells. *, *P* < 0.05; ***, *P* < 0.001.

**Fig. S6 Sorafenib induces apoptosis of HCC cells.**


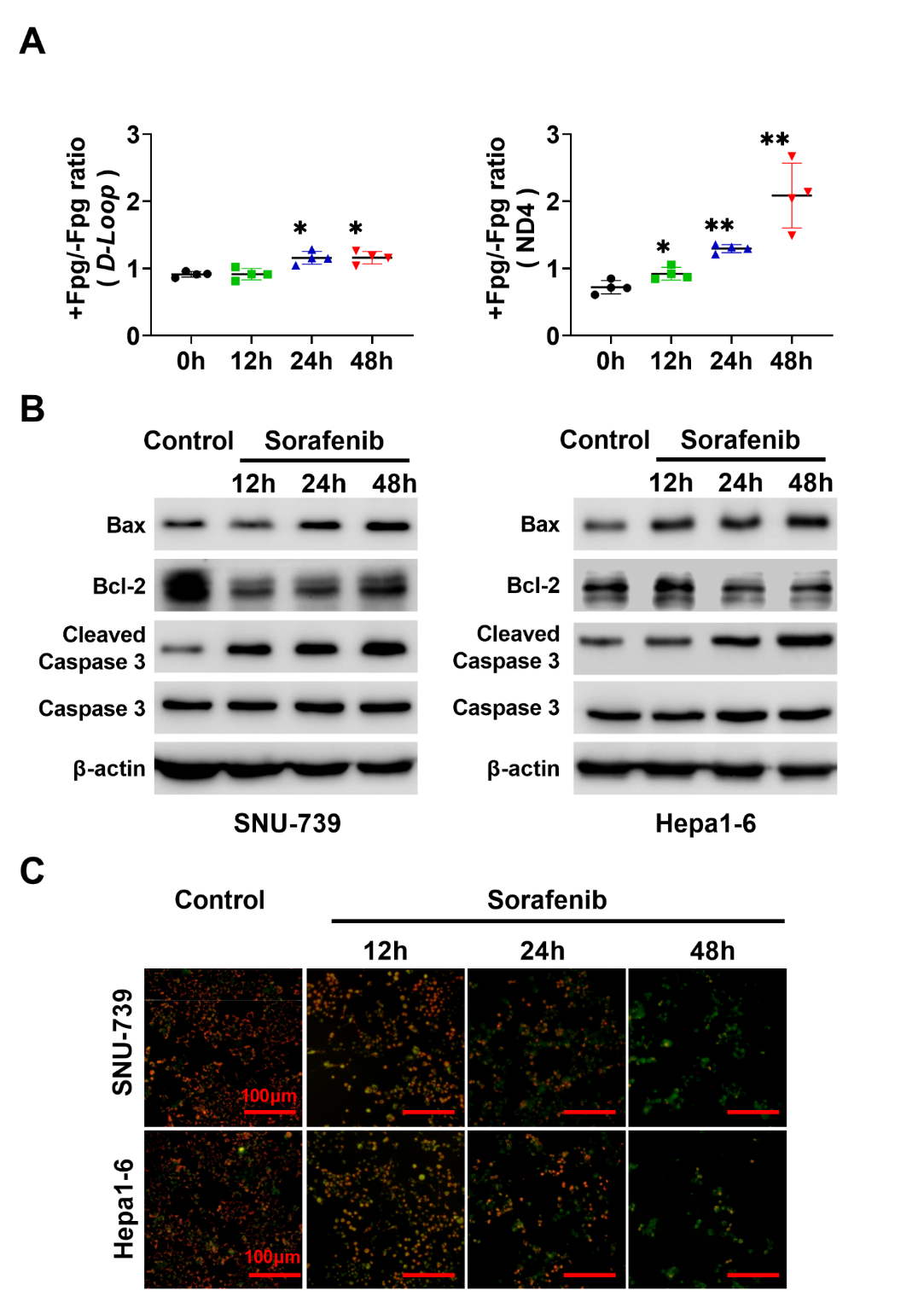


**(A)** qRT-PCR analyses of oxidized mtDNA in HCC cells treated with sorafenib for various time periods.

**(B)** Western blot analyses for apoptosis-relate protein expression were performed in human and murine HCC cells treated as indicated.

(**C)** Mitochondrial membrane potential was analyzed by JC-1 staining in human and murine HCC cells treated as indicated. Scale bars: 100 μm. *, *P* < 0.05; **, *P* < 0.01.

**Fig. S7 Mitochondrial DNA promotes M2 polarization of TAMs *in vitro*.**

**
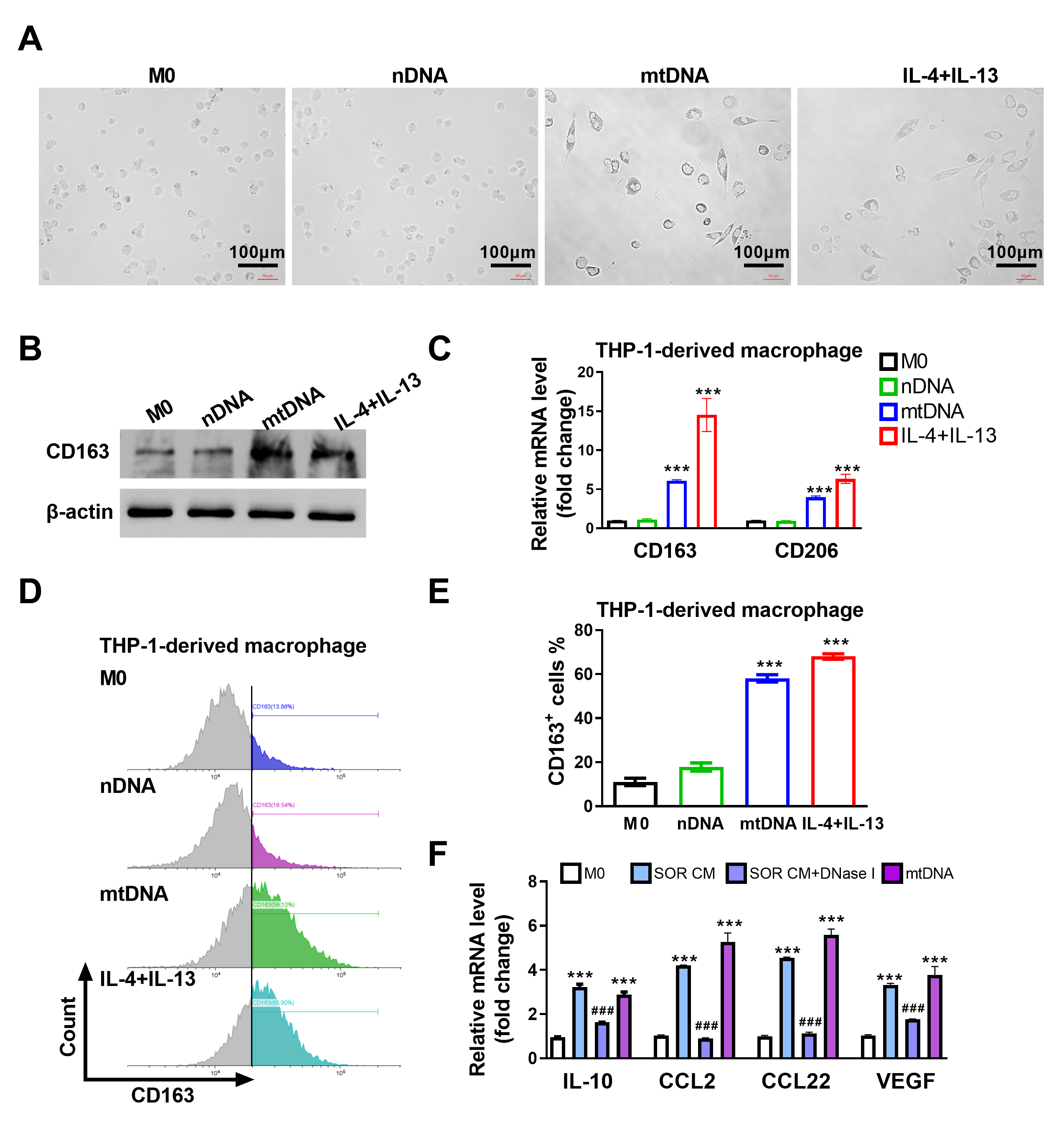
**

**(A)** Morphology of THP-1 derived macrophage treated as indicated. Scale bars: 100 μm. **(B and C)** Western blot (B) and qRT-PCR (C) analysis the expression levels of M2-TAM marker in THP-1 derived macrophages treated as indicated. **(D)** Flow cytometry analysis the percentage of CD163^+^ cells in THP-1 derived macrophages treated as indicated. **(E)** The percentage of CD163^+^ macrophage in C was statistical analysis. **(F)** qRT-PCR analysis the mRNA expression levels of M2 macrophage characteristic cytokines in derived macrophages treated as indicated. ***, *P* < 0.001 versus M0 group; ^###^, *P* < 0.001 versus SOR CM treated group. CM, conditioned medium; SOR, sorafenib.

**Fig. S8 Sorafenib promotes M2 polarization of TAMs *in vitro*.**


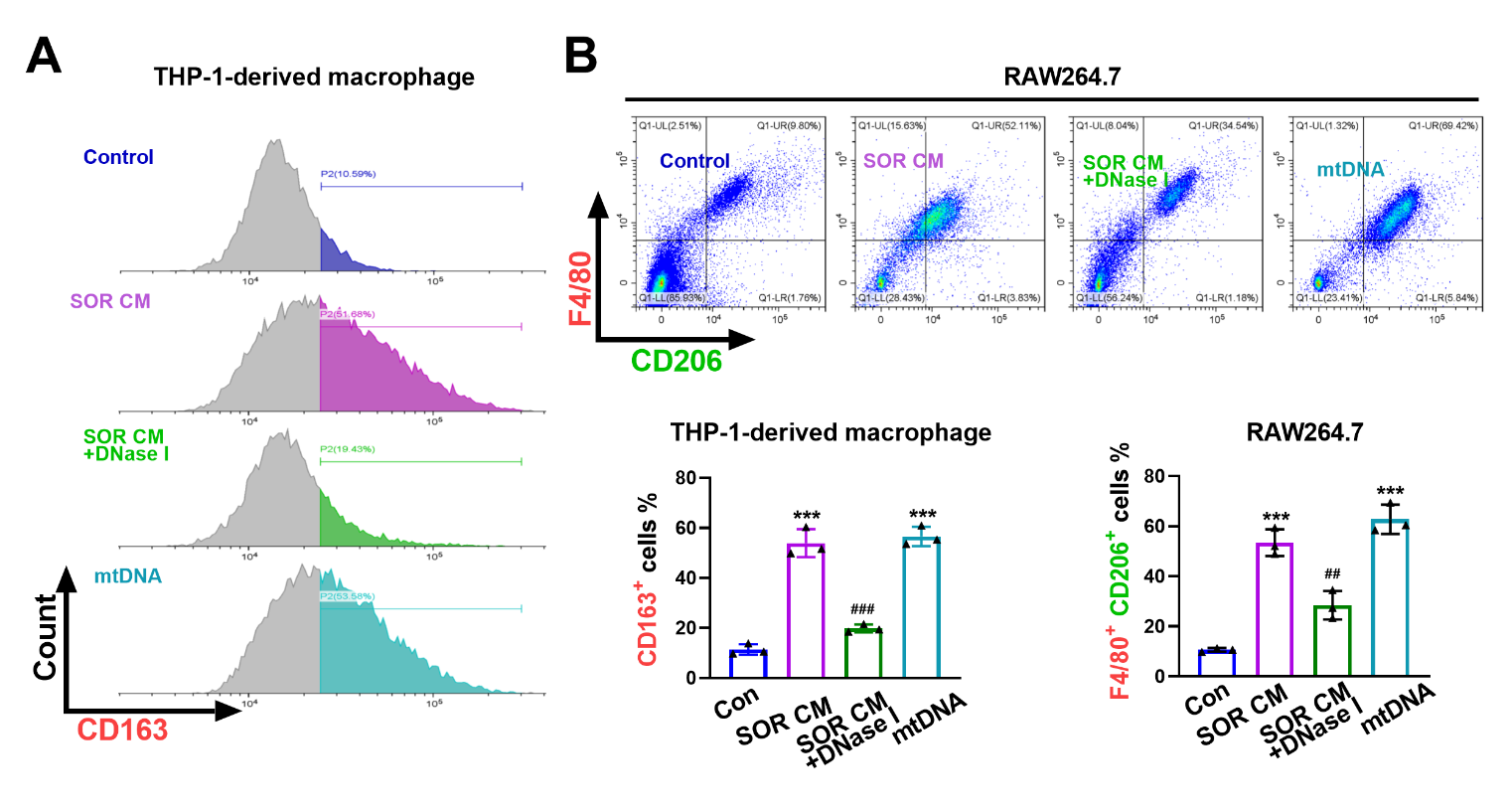


**(A)** Flow cytometry analysis of CD163^+^ cells in THP-1-derived macrophages treated with CM from HCC cells.

**(B)** Flow cytometry analysis of F4/80^+^/CD206^+^ cell percentage in RAW264.7 cells treated with CM from HCC cells. ***, *P* < 0.001. ^##^, *P* < 0.01; ^###^, *P* < 0.001

**Fig. S9 CIBERSORT analysis the correlated between TLR9 expression and M2 macrophage characteristic cytokines expression level.**


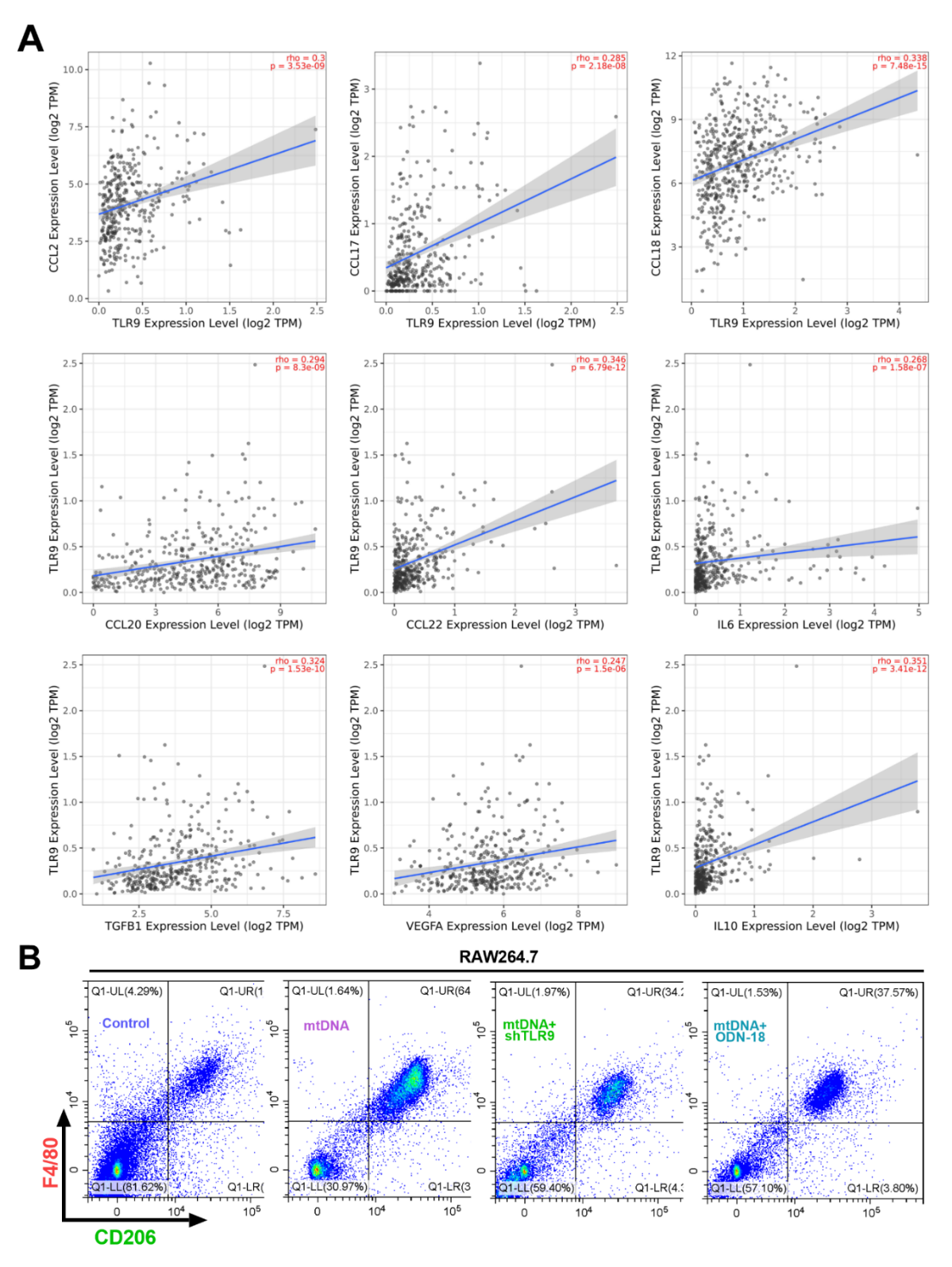


**(A)** CIBERSORT analysis the correlated between TLR9 expression and M2 macrophage characteristic cytokines expression level.

**(B)** Flow cytometry analysis of F4/80+/CD206+ cell percentage in RAW264.7 cells with treatments indicated.

**Fig. S10 The content of mtDNA in plasma of WT and** ***Tlr*9^-/-^ mice.**


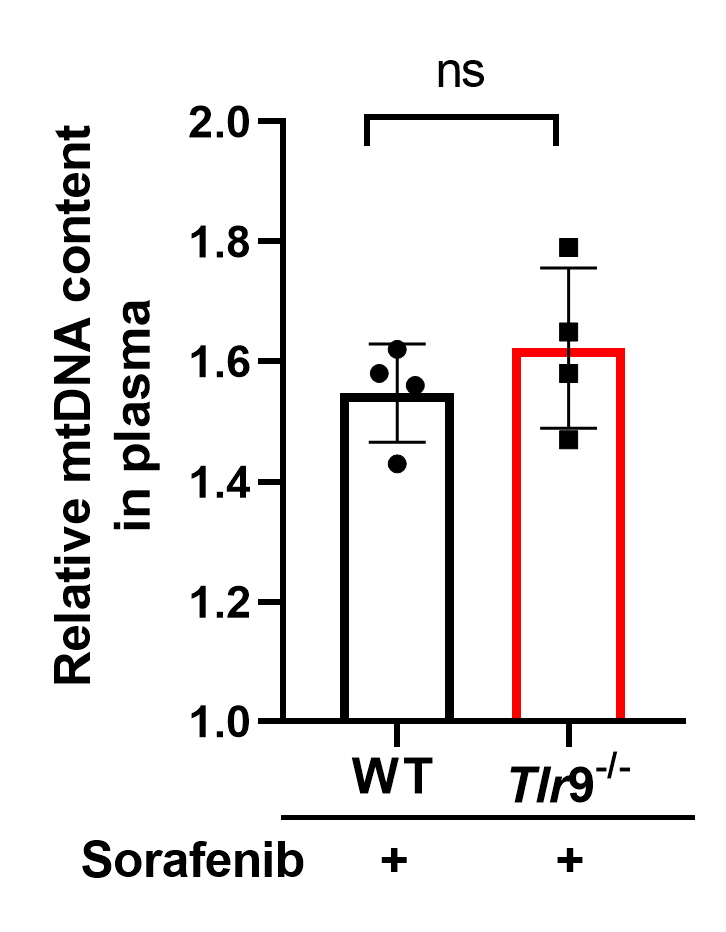


Relative mtDNA content in plasma from WT and *Tlr*9^-/-^ mice was detected by qPCR.

**Table S1. Distribution of HCC patients’ characteristics.**

| **Variable** | **All patients, n (%), n = 55** |
| --- | --- |
| Sex |  |
| Female | 7 (12.7%) |
| Male | 48 (87.3%) |
| Age, years |  |
| <50 | 30 (54.5%) |
| ≥50 | 25 (45.5%) |
| Serum HBsAg |  |
| Negative | 2 (3.6%) |
| Positive | 53 (96.4%) |
| Serum AFP, ng/ml |  |
| <200 | 32 (58.2%) |
| ≥200 | 23 (41.8%) |
| Size of tumor, cm |  |
| <5cm | 28 (50.9%) |
| ≥5cm | 27 (49.1%) |
| Number of tumor |  |
| =1 | 45 (81.8%) |
| ≥2 | 10 (18.2%) |
| PVT |  |
| No | 50 (90.9%) |
| Yes | 5 (9.1%) |
| TNM stage |  |
| I+II | 45 (81.8%) |
| III+IV | 10 (18.2%) |
| Survival |  |
| Death | 34 (61.8%) |
| Alive | 21 (38.2%) |

PVT, portal vein thrombus; TNM, tumor node metastasis; AFP, alphafetoprotein.

**Table S2. Sequence of primers for qRT-PCR and shRNA.**

| **Primers for qRT-PCR** | | |
| --- | --- | --- |
| **Target** | **Forward primer (5’-3’)** | **Reverse primer (5’-3’)** |
| Human |  |  |
| CD206 | CTCAACCCAAGGGCTCTTCTAA | AGGTGGCCTCTTGAGGTATGTG |
| ARG1 | CTCCAAGCCAAAGTCCTTAGAG | GGAGCTGTCATTAGGGACATCA |
| IL-10 | TCCCTGTCAAAACAAGAGCA | ATAGAGTCGCCACCCTGATG |
| CCL2 | CAGCCAGATGCAATCAATGCC | TGGAATCCTGAACCCACTTCT |
| CCL22 | TGATTACGTCCGTTACCGTCT | CCTGAAGGTTAGCAACACCAC |
| VEGF | TGGGTGCATTGGAGCCTTGCCTTGC | GGCAGTAGCTGCGCTGATAGACATCC |
| CD163 | TTTTGTCACCAGTTCTCTTGGA | AGCCATTATTACACACGTTCC |
| ND1 | CCCTAAAACCCGCCACATCT | GAGCGATGGTGAGAGCTAAGGT |
| 36B-4 | CAGCAAGTGGGAAGGTGTAATCC | CCCATTCTATCATCAACGGGTACAA |
| CD86 | CTTTGCTTCTCTGCTGCTGT | GGCCATCACAAAGAGAATGTTAC |
| IL-6 | AAGCCAGAGCTGTGCAGATGAGTA | TGTCCTGCAGCCACTGGTTC |
| GAPDH | AACGGATTTGGTCGTATTGG | TTGATTTTGGAGGGATCTCG |
| Mouse |  |  |
| CD206 | CTCAACCCAAGGGCTCTTCTAA | AGGTGGCCTCTTGAGGTATGTG |
| IL-10 | CTGAAGACCCTCAGGATGCG | GACACCTTGGTCTTGGAGCTTA |
| CCL2 | CTGCTGTTCACAGTTGCCG | GCACAGACCTCTCTCTTGAGC |
| CCL22 | CAAGCCTGGCGTTGTTTTGAT | GCAAGGCTCTTGCTGGAATG |
| VEGF | CTGACGATGGCCTGGAATGT | GAGGATCCTGGGGCTGTCT |
| ND1 | GCTGCGAAGTGGAAACCATC | CCTCCTTCTGCACACATTTGAA |
| 36B-4 | GAAACTGCTGCCTCACATCCG | GCTGGCACAGTGACCTCACACG |
| CD86 | TCAATGGGACTGCATATCTGCC | GCCAAAATACTACCAGCTCACT |
| IL-6 | GAGTGGCTAAGGACCAAGACC | AACGCACTAGGTTTGCCGA |
| GAPDH | TCACCATCTTCCAGGAGCGAGAC | AGACACCAGTAGACTCCACGACATAC |
| **Sequence of shRNA** | | |
| **Target** | **Sense (5’-3’)** | **Anti-sense (5’-3’)** |
| TLR9 | GCTGCCCAAATCCCTCATATC | GATATGAGGGATTTGGGCAGC |
| Control | GTTCTCCGAACGTGTCACGT | ACGTGACACGTTCGGAGAAC |

**Table S3. Reagents and antibodies used in this study.**

| **Reagent or Resouce**  **REAGENT or RESOURCE** | **Source** | **Identifier** |
| --- | --- | --- |
| **Antibodies for Western Blot** | | |
| Rabbit Anti-TLR9 monoclonal antibody | Cell Signaling | Cat#13674;RRID:AB_2798290 |
| Rabbit Anti-CD206 monoclonal antibody | Cell Signaling | Cat#24595;RRID:AB_2892682 |
| Rabbit Anti-CD163 monoclonal antibody | Cell Signaling | Cat#93498;RRID:AB_2800204 |
| Rabbit Anti-NF-κB p65 monoclonal antibody | Cell Signaling | Cat#8242;RRID:AB_10859369 |
| Rabbit Anti-phospho-NF-κB p65 monoclonal antibody | Cell Signaling | Cat#3033;RRID:AB_331284 |
| p38 MAPK Antibody | Cell Signaling | Cat#9212;RRID:AB_330713 |
| Phospho-p38 MAPK (Thr180/Tyr182) Antibody | Cell Signaling | Cat#9211;RRID:AB_331641 |
| Mouse Anti-beta Actin monoclonal Antibody | Proteintech | Cat#67243-1-Ig;RRID:AB_2341188 |
| Cleaved Caspase-3 | Cell Signaling | Cat#9661;RRID:AB_2341188 |
| Caspase-3 | Cell Signaling | Cat#9662;RRID:AB_2827489 |
| BAX polyclonal Antibody | Proteintech | Cat#50599-2-Ig;RRID:AB_2061561 |
| BLC2 polyclonal Antibody | Proteintech | Cat#ab194583;RRID:AB_2783814 |
| **Antibodies for Immunohistochemistry (IHC)** | | |
| Rabbit Anti-Ki-67 (D3B5) monoclonal antibody | Cell Signaling | Cat#9129;RRID:AB_11177044 |
| Rabbit Anti-CD206 monoclonal antibody | Cell Signaling | Cat#24595;RRID:AB_2892682 |
| Rabbit Anti-CD163 monoclonal antibody | Cell Signaling | Cat#93498;RRID:AB_2800204 |
| TLR9 | Cell Signaling | Cat#13674;RRID:AB_2798290 |
| Cleaved Caspase-3 | Cell Signaling | Cat#9661;RRID:AB_2341188 |
| **Antibodies for Immunohistochemistry (IF)** | | |
| F4/80 Monoclonal Antibody (BM8), APC | Invitrogen | Cat#41-4801-80 |
| dsDNA | Santa cruz | Cat#HYB331-01 |
| CD206 | Cell Signaling | Cat#24595;RRID:AB_2892682 |
| CD163 | Proteintech | Cat#16646-1-AP; RRID:AB_2756528 |
| Antibodies for Immunohistochemistry (FCM) | | |
| CD206 | Proteintech | Cat#18704-1-APRRID:AB_10597232 |
| CD163 | Proteintech | Cat#16646-1-AP；RRID:AB_2756528 |
| F4/80 Monoclonal Antibody (BM8), APC | Invitrogen | Cat#41-4801-80 |
| **Secondary Antibodies** | | |
| Anti-mouse IgG, HRP-linked Antibody | Cell Signaling | Cat#7076; RRID: AB_330924 |
| Anti-rabbit IgG, HRP-linked Antibody | Cell Signaling | Cat#7074; RRID: AB_2099233 |
| Goat Anti-Rabbit IgG H&L (Alexa Fluor® 488) | Abcam | Cat#ab150077;RRID:AB_2630356 |
| Goat Anti-Rabbit IgG (H+L) Alexa Fluor 594 | Abways | Cat#AB0151; |
| Biological Samples |  |  |
| Human HCC samples | This paper | N/A |
| **Chemicals, Peptides, and Recombinant Proteins** | | |
| Lipofectamine 2000 | Invitrogen | Cat# 11668019 |
| RIPA buffer | Beyotime, | Cat# P0013B |
| TRIzol LS Reagent | Invitrogen | Cat# 10296010 |
| Phosphatase Inhibitor Cocktail | Roche | Cat# 04906845001 |
| Protease Inhibitor Cocktail | Roche | Cat# 04693159001 |
| DNase I (free RNase ) | NEB | Cat# M0303S |
| Phorbol-12-myristate-13-acetate | Sigma | Cat#79346 |
| MTT | Solarbio | Cat#M8180 |
| DAPI Staining Solution | Bestibio | Cat#BB-4401 |
| ds DNA Marker(HYB331-01) | santacruz | Cat#sc-58749 |
| Sorafenib | Solarbio | Cat#S5080 |
| ODN-18 | MedChemExpress | Cat#HY-153840A |
| DEN | Sigma | Cat#N0756 |
| Caspas 3/7 Assay Kit | Bioquest | Cat#AAT-A13504 |
| Matrigel | Biosciences | Cat#354234 |
| E6446 | TargetMol | Cat#T4206 |
| IL-4 | MedChemExpress | Cat#HY-P70445 |
| IL-13 | MedChemExpress | Cat#HY-P70568 |
| **Critical Commercial Assays** | | |
| Mitochondria Isolation Kit | Beyotime | Cat# C3601 |
| Reactive Oxygen Species Assay Kit | Beyotime | Cat# S0033S |
| Mitochondrial membrane potential assay kit with JC-1 | Beyotime | Cat# C2006 |
| Cell Titer-Glo Luminescent assay (ATP) | Promega | Cat# G7570 |
| Instant Immunohistochemistry Kit | Sangon | Cat# C516337 |
| Annexin V-FITC/PI Apoptosis Kit | Yeasen | 40302ES20/50/60 |
| BCA Protein Assay Kit | Solarbio | Cat# PC0020 |
| CellAmp™ Direct SYBR®RT-qPCR Kit | TaKaRa | Cat# 3735A |
| PrimeScript™RT Master Mix(Perfect Real Time) | TaKaRa | Cat# RR036Q |
| (EdU) incorporation assay kit | Ribbio | Cat# C10310-1 |
| **Experimental Models: Cell Lines** | | |
| THP-1 | Procell Life Science and Technology | Cat# CL-0233 |
| SNU-739 | Cobioer Biosciences | Cat# CBP60219 |
| Hepa1-6 | Procell Life Science and Technology | Cat# CL-0105 |
| RAW264.7 | Procell Life Science and Technology | Cat#CL-0190 |
| **Mouse** | | |
| TLR9 knockout C57BL/6J mouse | This paper | N/A |
| **Software and Algorithms** | | |
| QX200 droplet reader software | Software Bio-Rad | QX200 droplet reader software |
| ImageJ | NIH | https://imagej.nih.gov/ij |
| Prism8 | Graphpad Software | https://www.graphpad.com/ scientiﬁc-software/prism |
| SPSS 20 | IBM | https://www.ibm.com/analytics/spss-statistics-software |

| **Table S4. Differentially expressed mRNAs in RNA sequencing data of mtDNA induced M2 polarization (log2fold change>1, p<0.05)** | | | | | |
| --- | --- | --- | --- | --- | --- |
| **Gene ID** | **baseMean** | **log2Foldchange** | **p value** | **padj** | **Gene Symbol** |
| ENSG00000181045 | 1270.039919 | 3.445787177 | 1.93E-221 | 4.75E-217 | SLC26A11 |
| ENSG00000104763 | 8811.195659 | 2.162212353 | 4.09E-207 | 5.04E-203 | ASAH1 |
| ENSG00000087086 | 278742.3303 | 2.747540384 | 2.52E-173 | 2.07E-169 | FTL |
| ENSG00000117016 | 3241.327761 | 2.232340747 | 2.15E-150 | 1.32E-146 | RIMS3 |
| ENSG00000164742 | 2109.939046 | 1.823621675 | 6.35E-130 | 3.13E-126 | ADCY1 |
| ENSG00000168329 | 410.9439162 | -3.81684461 | 7.43E-121 | 3.05E-117 | CX3CR1 |
| ENSG00000118785 | 8075.413459 | 2.094763728 | 3.62E-120 | 1.27E-116 | SPP1 |
| ENSG00000111912 | 3481.47786 | -1.635755474 | 4.35E-117 | 1.34E-113 | NCOA7 |
| ENSG00000135047 | 47002.31355 | 1.629762441 | 1.64E-111 | 4.48E-108 | CTSL |
| ENSG00000108828 | 4937.777469 | 1.769843662 | 5.12E-111 | 1.26E-107 | VAT1 |
| ENSG00000119899 | 6328.774216 | 1.460407868 | 2.26E-104 | 5.07E-101 | SLC17A5 |
| ENSG00000100427 | 897.2081461 | -2.074808817 | 3.32E-104 | 6.81E-101 | MLC1 |
| ENSG00000164733 | 25861.33177 | 1.806718517 | 2.52E-102 | 4.78E-99 | CTSB |
| ENSG00000171617 | 339.9925135 | -3.445577434 | 5.20E-101 | 9.16E-98 | ENC1 |
| ENSG00000160213 | 4715.916755 | 1.628035987 | 1.78E-97 | 2.92E-94 | CSTB |
| ENSG00000165029 | 9802.430164 | 1.48488461 | 3.88E-94 | 5.98E-91 | ABCA1 |
| ENSG00000025434 | 1409.38808 | 2.870683162 | 5.58E-94 | 8.08E-91 | NR1H3 |
| ENSG00000196950 | 2764.368617 | 1.612804876 | 7.48E-94 | 1.02E-90 | SLC39A10 |
| ENSG00000116679 | 4184.803098 | 1.430162265 | 2.79E-93 | 3.61E-90 | IVNS1ABP |
| ENSG00000104081 | 1103.346784 | 1.784605514 | 2.69E-91 | 3.32E-88 | BMF |
| ENSG00000153071 | 6449.233733 | 1.51911008 | 5.25E-88 | 6.16E-85 | DAB2 |
| ENSG00000071242 | 6773.85799 | 1.39855294 | 2.16E-82 | 2.42E-79 | RPS6KA2 |
| ENSG00000090013 | 414.3251124 | 2.940831956 | 3.45E-81 | 3.69E-78 | BLVRB |
| ENSG00000130208 | 2992.249362 | 1.864898047 | 4.98E-78 | 5.11E-75 | APOC1 |
| ENSG00000123384 | 4143.887848 | 1.471218574 | 4.06E-77 | 4.00E-74 | LRP1 |
| ENSG00000025039 | 2226.93367 | 1.843364608 | 1.62E-76 | 1.54E-73 | RRAGD |
| ENSG00000226608 | 608.3139895 | 2.732335816 | 1.67E-74 | 1.52E-71 | FTLP3 |
| ENSG00000152518 | 6682.298249 | -1.550346979 | 1.27E-73 | 1.12E-70 | ZFP36L2 |
| ENSG00000148516 | 2544.24602 | 1.626866976 | 3.27E-73 | 2.77E-70 | ZEB1 |
| ENSG00000267519 | 1677.529189 | -2.097172856 | 9.83E-72 | 8.07E-69 | MIR23AHG |
| ENSG00000184465 | 2433.803942 | 1.354718597 | 4.68E-71 | 3.72E-68 | WDR27 |
| ENSG00000121966 | 1112.59468 | 2.135598532 | 4.63E-69 | 3.56E-66 | CXCR4 |
| ENSG00000182197 | 262.2914618 | -3.168523547 | 1.34E-68 | 1.00E-65 | EXT1 |
| ENSG00000163870 | 1023.52396 | 1.730304339 | 2.88E-68 | 2.09E-65 | TPRA1 |
| ENSG00000125772 | 2256.132591 | 2.420731935 | 1.23E-67 | 8.65E-65 | GPCPD1 |
| ENSG00000206190 | 1376.712869 | -2.314846595 | 2.11E-67 | 1.44E-64 | ATP10A |
| ENSG00000071246 | 1913.534458 | -2.112719126 | 2.02E-66 | 1.34E-63 | VASH1 |
| ENSG00000139289 | 17221.61976 | 1.158439657 | 2.80E-66 | 1.82E-63 | PHLDA1 |
| ENSG00000085117 | 1635.606999 | -1.319332944 | 3.43E-66 | 2.16E-63 | CD82 |
| ENSG00000185885 | 336.7023295 | -2.714568729 | 1.41E-65 | 8.67E-63 | IFITM1 |
| ENSG00000105968 | 1097.51843 | -1.76159575 | 2.37E-65 | 1.41E-62 | H2AZ2 |
| ENSG00000140479 | 2515.436654 | 1.260056327 | 2.40E-65 | 1.41E-62 | PCSK6 |
| ENSG00000149260 | 372.5315613 | 3.604910033 | 4.16E-65 | 2.39E-62 | CAPN5 |
| ENSG00000071553 | 5629.426319 | 1.131334491 | 1.33E-64 | 7.44E-62 | ATP6AP1 |
| ENSG00000166750 | 910.556886 | 1.736130206 | 1.63E-64 | 8.93E-62 | SLFN5 |
| ENSG00000183019 | 690.7150505 | -2.792242794 | 8.36E-64 | 4.48E-61 | MCEMP1 |
| ENSG00000109158 | 185.3642304 | -4.767493632 | 2.10E-63 | 1.10E-60 | GABRA4 |
| ENSG00000028137 | 3945.5638 | 1.129643911 | 2.16E-63 | 1.11E-60 | TNFRSF1B |
| ENSG00000185504 | 2351.809458 | 1.48316232 | 3.06E-63 | 1.54E-60 | FAAP100 |
| ENSG00000127124 | 3975.579683 | -1.685564112 | 5.52E-63 | 2.72E-60 | HIVEP3 |
| ENSG00000109046 | 6818.200339 | 1.35041898 | 2.59E-62 | 1.25E-59 | WSB1 |
| ENSG00000087589 | 1205.700351 | -1.675951893 | 4.06E-62 | 1.92E-59 | CASS4 |
| ENSG00000164713 | 1426.821764 | 1.431592041 | 1.62E-61 | 7.54E-59 | BRI3 |
| ENSG00000204387 | 2256.551847 | 1.317070596 | 1.67E-60 | 7.62E-58 | SNHG32 |
| ENSG00000095383 | 792.9417626 | 1.844512813 | 2.90E-60 | 1.30E-57 | TBC1D2 |
| ENSG00000130066 | 9556.578643 | 1.240505852 | 4.26E-60 | 1.87E-57 | SAT1 |
| ENSG00000112977 | 4274.39176 | -1.417246314 | 5.58E-60 | 2.41E-57 | DAP |
| ENSG00000151726 | 5500.333251 | 1.3996825 | 1.56E-59 | 6.63E-57 | ACSL1 |
| ENSG00000101335 | 919.796385 | -1.750775971 | 4.92E-59 | 2.06E-56 | MYL9 |
| ENSG00000123908 | 19703.36701 | 1.452407248 | 6.22E-59 | 2.55E-56 | AGO2 |
| ENSG00000184584 | 560.8351963 | -1.958649065 | 7.76E-59 | 3.13E-56 | STING1 |
| ENSG00000196628 | 683.7401189 | -1.738389633 | 7.25E-58 | 2.84E-55 | TCF4 |
| ENSG00000095209 | 926.5809736 | 1.625584581 | 7.27E-58 | 2.84E-55 | TMEM38B |
| ENSG00000167996 | 48197.34694 | 2.114161981 | 7.38E-58 | 2.84E-55 | FTH1 |
| ENSG00000117984 | 7358.769267 | 1.820168082 | 1.01E-57 | 3.83E-55 | CTSD |
| ENSG00000013561 | 1071.019052 | 1.507430419 | 1.63E-56 | 6.10E-54 | RNF14 |
| ENSG00000102531 | 14121.34783 | 1.47862431 | 2.63E-56 | 9.67E-54 | FNDC3A |
| ENSG00000131504 | 5933.79654 | -1.185012286 | 2.91E-56 | 1.05E-53 | DIAPH1 |
| ENSG00000182372 | 2081.032294 | 1.831494298 | 1.65E-55 | 5.88E-53 | CLN8 |
| ENSG00000115963 | 4232.79694 | 1.200715707 | 2.23E-55 | 7.83E-53 | RND3 |
| ENSG00000113719 | 2411.468356 | 1.627321129 | 7.11E-55 | 2.47E-52 | ERGIC1 |
| ENSG00000182985 | 639.1740991 | 2.05567591 | 1.74E-54 | 5.95E-52 | CADM1 |
| ENSG00000100448 | 820.896199 | -2.808186439 | 1.93E-54 | 6.50E-52 | CTSG |
| ENSG00000145780 | 2340.603275 | 1.616422685 | 3.79E-54 | 1.26E-51 | FEM1C |
| ENSG00000113552 | 1922.781376 | 1.237242205 | 9.04E-53 | 2.97E-50 | GNPDA1 |
| ENSG00000159388 | 1293.314619 | -1.785998716 | 2.28E-52 | 7.39E-50 | BTG2 |
| ENSG00000185745 | 565.9139353 | -2.511111169 | 4.02E-52 | 1.29E-49 | IFIT1 |
| ENSG00000130203 | 5188.336646 | 2.037381032 | 7.63E-52 | 2.41E-49 | APOE |
| ENSG00000111335 | 3438.142582 | -1.173258566 | 1.08E-51 | 3.37E-49 | OAS2 |
| ENSG00000177426 | 2398.604981 | 1.634107808 | 1.14E-51 | 3.52E-49 | TGIF1 |
| ENSG00000118513 | 1245.805866 | -1.690097782 | 1.45E-51 | 4.42E-49 | MYB |
| ENSG00000127507 | 2936.946276 | -1.380447158 | 1.86E-51 | 5.59E-49 | ADGRE2 |
| ENSG00000134046 | 790.0824687 | -1.456650829 | 3.04E-51 | 9.01E-49 | MBD2 |
| ENSG00000064687 | 492.4099181 | 1.821104462 | 3.51E-51 | 1.03E-48 | ABCA7 |
| ENSG00000218336 | 591.5316215 | -1.725695078 | 5.17E-51 | 1.50E-48 | TENM3 |
| ENSG00000070540 | 536.0015129 | 2.050922682 | 5.86E-51 | 1.68E-48 | WIPI1 |
| ENSG00000105939 | 3842.332995 | 1.139717018 | 1.77E-50 | 5.00E-48 | ZC3HAV1 |
| ENSG00000052795 | 3207.350244 | 1.915986646 | 2.37E-50 | 6.63E-48 | FNIP2 |
| ENSG00000049130 | 912.2582094 | 1.405076005 | 1.13E-49 | 3.10E-47 | KITLG |
| ENSG00000163219 | 1255.31297 | 1.469541001 | 2.50E-49 | 6.76E-47 | ARHGAP25 |
| ENSG00000228956 | 362.6153189 | -2.078080738 | 2.89E-49 | 7.74E-47 | SATB1-AS1 |
| ENSG00000135124 | 2427.915024 | 1.187130349 | 5.07E-49 | 1.34E-46 | P2RX4 |
| ENSG00000136235 | 6252.078028 | 1.205209186 | 2.12E-48 | 5.55E-46 | GPNMB |
| ENSG00000084731 | 315.237249 | -2.178646896 | 7.87E-48 | 2.04E-45 | KIF3C |
| ENSG00000147036 | 945.2385378 | -1.33315998 | 8.28E-48 | 2.12E-45 | LANCL3 |
| ENSG00000135414 | 674.2399362 | -1.677677641 | 9.08E-48 | 2.31E-45 | GDF11 |
| ENSG00000205765 | 2231.380137 | 1.305002761 | 1.04E-47 | 2.62E-45 | RIMOC1 |
| ENSG00000196214 | 435.5107155 | 1.773666807 | 1.26E-47 | 3.13E-45 | ZNF766 |
| ENSG00000114796 | 1340.393089 | 2.023657554 | 2.68E-47 | 6.61E-45 | KLHL24 |
| ENSG00000047644 | 257.6201418 | -2.309846034 | 6.46E-47 | 1.56E-44 | WWC3 |
| ENSG00000205927 | 1008.88757 | 1.32922586 | 1.49E-46 | 3.57E-44 | OLIG2 |
| ENSG00000164604 | 252.7032898 | -2.462517806 | 2.88E-46 | 6.81E-44 | GPR85 |
| ENSG00000105223 | 2082.718037 | 1.147623767 | 4.39E-46 | 1.03E-43 | PLD3 |
| ENSG00000058335 | 1093.078619 | -1.465016119 | 5.87E-46 | 1.36E-43 | RASGRF1 |
| ENSG00000166340 | 8412.901398 | 1.088858917 | 6.23E-46 | 1.43E-43 | TPP1 |
| ENSG00000089685 | 506.9478251 | -1.958610313 | 9.02E-46 | 2.06E-43 | BIRC5 |
| ENSG00000134986 | 1632.679993 | -1.988115568 | 1.22E-45 | 2.76E-43 | NREP |
| ENSG00000117724 | 1187.747019 | -1.906060328 | 1.36E-45 | 3.05E-43 | CENPF |
| ENSG00000163053 | 150.4706237 | 3.490778749 | 1.80E-45 | 4.00E-43 | SLC16A14 |
| ENSG00000231346 | 448.7902028 | -1.866038651 | 2.32E-45 | 5.10E-43 | LINC01160 |
| ENSG00000151743 | 203.7450513 | 2.813793175 | 2.37E-45 | 5.16E-43 | AMN1 |
| ENSG00000116985 | 7127.574796 | -1.177607244 | 5.88E-45 | 1.27E-42 | BMP8B |
| ENSG00000143622 | 2195.061372 | 1.140222081 | 9.14E-45 | 1.96E-42 | RIT1 |
| ENSG00000148773 | 3059.084964 | -1.193474656 | 9.56E-45 | 2.03E-42 | MKI67 |
| ENSG00000186141 | 2084.341731 | -1.157433085 | 1.69E-44 | 3.57E-42 | POLR3C |
| ENSG00000167210 | 523.121342 | -1.819719681 | 1.87E-44 | 3.91E-42 | LOXHD1 |
| ENSG00000196371 | 2623.809241 | -1.066587301 | 2.60E-44 | 5.34E-42 | FUT4 |
| ENSG00000185420 | 613.9965467 | -1.544321833 | 3.36E-44 | 6.84E-42 | SMYD3 |
| ENSG00000101160 | 3430.439865 | 1.126712573 | 3.55E-44 | 7.16E-42 | CTSZ |
| ENSG00000114423 | 621.2961368 | 1.573121376 | 5.39E-44 | 1.07E-41 | CBLB |
| ENSG00000181523 | 362.6288843 | 1.92511833 | 9.79E-44 | 1.93E-41 | SGSH |
| ENSG00000172817 | 492.4216436 | -2.390284448 | 1.33E-43 | 2.61E-41 | CYP7B1 |
| ENSG00000135077 | 3918.77478 | 1.011466437 | 1.48E-43 | 2.87E-41 | HAVCR2 |
| ENSG00000171509 | 988.6641521 | -1.930401908 | 1.60E-43 | 3.08E-41 | RXFP1 |
| ENSG00000197746 | 30210.87993 | 1.000584186 | 1.80E-43 | 3.44E-41 | PSAP |
| ENSG00000160179 | 951.1156222 | 2.038053474 | 2.08E-43 | 3.94E-41 | ABCG1 |
| ENSG00000159176 | 1158.351748 | -1.291686987 | 2.28E-43 | 4.28E-41 | CSRP1 |
| ENSG00000213614 | 1896.088677 | 1.129255477 | 2.68E-43 | 5.00E-41 | HEXA |
| ENSG00000130511 | 1318.898255 | -1.227145167 | 3.48E-43 | 6.44E-41 | SSBP4 |
| ENSG00000155158 | 1131.230174 | 1.675369399 | 3.62E-43 | 6.65E-41 | TTC39B |
| ENSG00000197961 | 3221.620929 | 1.119931187 | 5.41E-43 | 9.88E-41 | ZNF121 |
| ENSG00000136098 | 191.8831068 | -2.417384585 | 6.09E-43 | 1.10E-40 | NEK3 |
| ENSG00000153012 | 362.4838048 | 2.321372536 | 6.62E-43 | 1.19E-40 | LGI2 |
| ENSG00000164830 | 3830.281084 | 1.370660192 | 7.66E-43 | 1.37E-40 | OXR1 |
| ENSG00000137575 | 8735.173553 | 1.067687436 | 9.66E-43 | 1.70E-40 | SDCBP |
| ENSG00000143554 | 235.14307 | -2.19313017 | 2.58E-42 | 4.50E-40 | SLC27A3 |
| ENSG00000164823 | 1517.81964 | 1.376301903 | 6.28E-42 | 1.08E-39 | OSGIN2 |
| ENSG00000103249 | 3730.842323 | 1.163545781 | 9.02E-42 | 1.54E-39 | CLCN7 |
| ENSG00000125733 | 1151.955774 | 1.192319118 | 1.88E-41 | 3.19E-39 | TRIP10 |
| ENSG00000198203 | 118.7269368 | 4.318013511 | 2.23E-41 | 3.77E-39 | SULT1C2 |
| ENSG00000265972 | 5315.963594 | 1.910610375 | 2.53E-41 | 4.25E-39 | TXNIP |
| ENSG00000117632 | 937.3490111 | -1.463546342 | 3.11E-41 | 5.18E-39 | STMN1 |
| ENSG00000107798 | 13027.06989 | 1.663660051 | 6.39E-41 | 1.06E-38 | LIPA |
| ENSG00000104921 | 773.8576665 | -1.477032138 | 7.18E-41 | 1.18E-38 | FCER2 |
| ENSG00000166478 | 696.7321332 | 1.515661045 | 9.62E-41 | 1.57E-38 | ZNF143 |
| ENSG00000166508 | 1029.974077 | -1.333677556 | 1.23E-40 | 1.99E-38 | MCM7 |
| ENSG00000144711 | 1975.038081 | 1.559817363 | 1.86E-40 | 2.99E-38 | IQSEC1 |
| ENSG00000149136 | 1999.628761 | -1.184622926 | 2.15E-40 | 3.43E-38 | SSRP1 |
| ENSG00000139679 | 3073.508161 | 1.032478185 | 2.83E-40 | 4.50E-38 | LPAR6 |
| ENSG00000188647 | 2291.212037 | 1.026214263 | 4.87E-40 | 7.69E-38 | PTAR1 |
| ENSG00000125968 | 229.4223985 | -2.234411681 | 1.33E-39 | 2.09E-37 | ID1 |
| ENSG00000001167 | 2907.56903 | 1.222261836 | 1.60E-39 | 2.49E-37 | NFYA |
| ENSG00000103381 | 378.7124053 | -1.760709299 | 2.28E-39 | 3.51E-37 | CPPED1 |
| ENSG00000185811 | 1304.907636 | -1.09017398 | 2.54E-39 | 3.88E-37 | IKZF1 |
| ENSG00000139970 | 142.2054196 | -2.834212936 | 2.80E-39 | 4.25E-37 | RTN1 |
| ENSG00000196776 | 960.4460747 | 1.278778169 | 3.18E-39 | 4.81E-37 | CD47 |
| ENSG00000165424 | 630.5226738 | -1.494660442 | 3.24E-39 | 4.87E-37 | ZCCHC24 |
| ENSG00000269893 | 912.1519786 | 1.228521782 | 3.93E-39 | 5.86E-37 | SNHG8 |
| ENSG00000112394 | 326.946179 | 1.78470315 | 6.01E-39 | 8.92E-37 | SLC16A10 |
| ENSG00000139832 | 394.6869579 | 1.823466255 | 1.56E-38 | 2.31E-36 | RAB20 |
| ENSG00000134072 | 1211.286868 | -1.264984113 | 1.79E-38 | 2.62E-36 | CAMK1 |
| ENSG00000171488 | 2023.331942 | -1.398974845 | 2.69E-38 | 3.93E-36 | LRRC8C |
| ENSG00000131747 | 2755.521145 | -1.564290458 | 3.10E-38 | 4.50E-36 | TOP2A |
| ENSG00000100644 | 4379.199218 | 1.4739323 | 3.25E-38 | 4.68E-36 | HIF1A |
| ENSG00000138185 | 919.1416209 | -1.183732424 | 3.42E-38 | 4.90E-36 | ENTPD1 |
| ENSG00000196935 | 248.4152567 | 2.195089602 | 4.80E-38 | 6.83E-36 | SRGAP1 |
| ENSG00000169607 | 413.99836 | -1.700696075 | 4.82E-38 | 6.83E-36 | CKAP2L |
| ENSG00000134326 | 789.261782 | -2.089746246 | 7.13E-38 | 9.98E-36 | CMPK2 |
| ENSG00000118985 | 3158.714652 | 1.490103813 | 8.39E-38 | 1.17E-35 | ELL2 |
| ENSG00000184985 | 524.2477376 | -1.936941812 | 9.35E-38 | 1.29E-35 | SORCS2 |
| ENSG00000118432 | 201.4953495 | 2.889085693 | 1.54E-37 | 2.11E-35 | CNR1 |
| ENSG00000204386 | 3004.294104 | 1.248534825 | 1.54E-37 | 2.11E-35 | NEU1 |
| ENSG00000101188 | 343.5088778 | -1.724515675 | 1.64E-37 | 2.23E-35 | NTSR1 |
| ENSG00000166562 | 499.4163489 | -1.458629119 | 1.72E-37 | 2.32E-35 | SEC11C |
| ENSG00000123983 | 4225.742144 | 1.033951199 | 2.21E-37 | 2.98E-35 | ACSL3 |
| ENSG00000113758 | 610.2356168 | -1.77611055 | 2.30E-37 | 3.09E-35 | DBN1 |
| ENSG00000106799 | 3125.453318 | -1.503251489 | 3.39E-37 | 4.52E-35 | TGFBR1 |
| ENSG00000081237 | 2251.77567 | -1.199035334 | 4.09E-37 | 5.42E-35 | PTPRC |
| ENSG00000189060 | 6714.607863 | 1.014598648 | 6.34E-37 | 8.30E-35 | H1-0 |
| ENSG00000197405 | 7574.460152 | 1.323400383 | 7.06E-37 | 9.20E-35 | C5AR1 |
| ENSG00000171163 | 692.2046023 | 1.47298906 | 1.33E-36 | 1.73E-34 | ZNF692 |
| ENSG00000134324 | 1930.459148 | 1.445190258 | 1.46E-36 | 1.88E-34 | LPIN1 |
| ENSG00000166598 | 13256.43372 | -1.010474482 | 1.58E-36 | 2.02E-34 | HSP90B1 |
| ENSG00000138131 | 473.8959534 | 2.085389237 | 1.65E-36 | 2.11E-34 | LOXL4 |
| ENSG00000113810 | 3233.361093 | -1.087134278 | 2.19E-36 | 2.79E-34 | SMC4 |
| ENSG00000196187 | 2699.572691 | 1.182568634 | 2.69E-36 | 3.39E-34 | TMEM63A |
| ENSG00000121152 | 475.7248195 | -1.839035124 | 2.94E-36 | 3.69E-34 | NCAPH |
| ENSG00000074416 | 1425.972315 | 1.916989107 | 3.88E-36 | 4.85E-34 | MGLL |
| ENSG00000260804 | 241.8232769 | -2.023599144 | 5.13E-36 | 6.38E-34 | LINC01963 |
| ENSG00000113163 | 1935.407215 | 1.1389219 | 1.80E-35 | 2.19E-33 | CERT1 |
| ENSG00000167703 | 925.5727441 | 1.913956855 | 1.84E-35 | 2.23E-33 | SLC43A2 |
| ENSG00000143774 | 1926.267166 | 1.111007769 | 2.19E-35 | 2.65E-33 | GUK1 |
| ENSG00000127418 | 1053.210292 | 1.16823724 | 2.47E-35 | 2.97E-33 | FGFRL1 |
| ENSG00000050405 | 1198.023519 | -1.130403502 | 2.57E-35 | 3.07E-33 | LIMA1 |
| ENSG00000176659 | 141.3892945 | -2.90906729 | 4.36E-35 | 5.17E-33 | LINC02910 |
| ENSG00000137959 | 503.5859266 | -1.691443621 | 4.54E-35 | 5.35E-33 | IFI44L |
| ENSG00000138764 | 493.8945234 | 1.382063439 | 4.81E-35 | 5.64E-33 | CCNG2 |
| ENSG00000196743 | 2067.907499 | 1.966142465 | 7.34E-35 | 8.57E-33 | GM2A |
| ENSG00000184897 | 1035.094455 | 1.460333985 | 7.94E-35 | 9.18E-33 | H1-10 |
| ENSG00000152582 | 231.2989431 | -1.987983863 | 8.78E-35 | 1.01E-32 | SPEF2 |
| ENSG00000005156 | 665.2425831 | 1.374068316 | 9.85E-35 | 1.12E-32 | LIG3 |
| ENSG00000125430 | 490.5192692 | -1.506671294 | 1.06E-34 | 1.20E-32 | HS3ST3B1 |
| ENSG00000104915 | 1084.256906 | 1.159282895 | 1.24E-34 | 1.40E-32 | STX10 |
| ENSG00000147231 | 1346.417484 | -1.190470185 | 1.57E-34 | 1.76E-32 | RADX |
| ENSG00000075426 | 5650.300649 | 1.526785167 | 1.65E-34 | 1.84E-32 | FOSL2 |
| ENSG00000134369 | 242.4341111 | -1.857969591 | 1.91E-34 | 2.13E-32 | NAV1 |
| ENSG00000139410 | 411.0343268 | 1.633794296 | 2.10E-34 | 2.33E-32 | SDSL |
| ENSG00000187231 | 712.1913822 | 1.41907471 | 2.44E-34 | 2.69E-32 | SESTD1 |
| ENSG00000161921 | 463.0130127 | 1.585333076 | 3.06E-34 | 3.35E-32 | CXCL16 |
| ENSG00000175556 | 1324.431807 | 1.209746794 | 3.19E-34 | 3.47E-32 | LONRF3 |
| ENSG00000111801 | 248.3924139 | 2.194031088 | 3.60E-34 | 3.91E-32 | BTN3A3 |
| ENSG00000122547 | 1651.865738 | 1.099551629 | 3.65E-34 | 3.94E-32 | EEPD1 |
| ENSG00000085978 | 1193.650261 | -1.198170955 | 3.82E-34 | 4.11E-32 | ATG16L1 |
| ENSG00000120217 | 112.4757737 | 3.406855995 | 4.71E-34 | 5.05E-32 | CD274 |
| ENSG00000166401 | 1269.940659 | -1.232618701 | 5.57E-34 | 5.94E-32 | SERPINB8 |
| ENSG00000119917 | 949.165982 | -1.628739403 | 6.02E-34 | 6.39E-32 | IFIT3 |
| ENSG00000145390 | 533.7355463 | 1.353559741 | 9.22E-34 | 9.75E-32 | USP53 |
| ENSG00000036672 | 1787.757538 | 1.308964887 | 1.02E-33 | 1.08E-31 | USP2 |
| ENSG00000088325 | 1194.159862 | -1.18085902 | 1.72E-33 | 1.80E-31 | TPX2 |
| ENSG00000145050 | 1146.734149 | -1.173991656 | 1.73E-33 | 1.80E-31 | MANF |
| ENSG00000196911 | 271.4317121 | -1.88660069 | 1.81E-33 | 1.89E-31 | KPNA5 |
| ENSG00000225697 | 1897.154437 | 1.043421299 | 2.06E-33 | 2.13E-31 | SLC26A6 |
| ENSG00000240184 | 2821.892899 | -1.097166878 | 2.41E-33 | 2.48E-31 | PCDHGC3 |
| ENSG00000100167 | 605.296035 | 1.40780782 | 2.42E-33 | 2.48E-31 | SEPTIN3 |
| ENSG00000184060 | 841.8197333 | 1.85205669 | 2.79E-33 | 2.85E-31 | ADAP2 |
| ENSG00000163251 | 416.0883804 | 1.852278608 | 2.88E-33 | 2.93E-31 | FZD5 |
| ENSG00000104805 | 3878.666539 | 1.021485281 | 3.47E-33 | 3.52E-31 | NUCB1 |
| ENSG00000185432 | 1557.41406 | 1.678649398 | 4.47E-33 | 4.48E-31 | METTL7A |
| ENSG00000163362 | 956.068961 | -1.472798229 | 4.83E-33 | 4.80E-31 | INAVA |
| ENSG00000174705 | 2095.829302 | 1.541424881 | 5.70E-33 | 5.63E-31 | SH3PXD2B |
| ENSG00000138623 | 1750.31886 | -1.744014415 | 7.44E-33 | 7.30E-31 | SEMA7A |
| ENSG00000023892 | 830.7277806 | -1.192577716 | 8.10E-33 | 7.92E-31 | DEF6 |
| ENSG00000125534 | 808.8435018 | 2.330114972 | 9.70E-33 | 9.44E-31 | PPDPF |
| ENSG00000184937 | 578.3068865 | -1.260203855 | 1.02E-32 | 9.86E-31 | WT1 |
| ENSG00000183242 | 200.4858863 | -2.07353746 | 1.03E-32 | 1.00E-30 | WT1-AS |
| ENSG00000008294 | 11059.77517 | 1.191294681 | 1.22E-32 | 1.17E-30 | SPAG9 |
| ENSG00000115295 | 744.5523935 | 1.384598426 | 1.24E-32 | 1.19E-30 | CLIP4 |
| ENSG00000111249 | 370.399175 | -1.539979917 | 1.69E-32 | 1.62E-30 | CUX2 |
| ENSG00000070081 | 818.8998005 | -1.57239366 | 1.74E-32 | 1.66E-30 | NUCB2 |
| ENSG00000164983 | 1641.043942 | 1.082274601 | 1.77E-32 | 1.68E-30 | TMEM65 |
| ENSG00000196923 | 1136.35502 | -1.325680129 | 2.83E-32 | 2.66E-30 | PDLIM7 |
| ENSG00000064601 | 4294.612401 | 1.237674925 | 3.67E-32 | 3.44E-30 | CTSA |
| ENSG00000187210 | 1217.238213 | 1.171015506 | 3.93E-32 | 3.67E-30 | GCNT1 |
| ENSG00000125827 | 260.5054297 | -1.749551611 | 5.37E-32 | 4.99E-30 | TMX4 |
| ENSG00000167470 | 1646.978376 | 1.400629877 | 5.81E-32 | 5.38E-30 | MIDN |
| ENSG00000178104 | 513.2285726 | 1.488549078 | 8.73E-32 | 8.06E-30 | PDE4DIP |
| ENSG00000165801 | 430.2023806 | -1.39418855 | 1.10E-31 | 1.01E-29 | ARHGEF40 |
| ENSG00000137462 | 5469.773614 | 1.206176783 | 1.20E-31 | 1.10E-29 | TLR2 |
| ENSG00000075618 | 4036.056664 | -1.278934718 | 1.22E-31 | 1.12E-29 | FSCN1 |
| ENSG00000134815 | 2039.538748 | 1.646412162 | 1.44E-31 | 1.31E-29 | DHX34 |
| ENSG00000159200 | 12893.08055 | 1.762707563 | 1.47E-31 | 1.33E-29 | RCAN1 |
| ENSG00000090889 | 286.7155483 | -1.925794781 | 1.81E-31 | 1.63E-29 | KIF4A |
| ENSG00000107872 | 232.4829038 | 2.096671486 | 1.97E-31 | 1.77E-29 | FBXL15 |
| ENSG00000131370 | 373.3382511 | 1.649976081 | 2.30E-31 | 2.04E-29 | SH3BP5 |
| ENSG00000105429 | 1229.041333 | -1.05674367 | 2.46E-31 | 2.18E-29 | MEGF8 |
| ENSG00000174282 | 857.9745229 | 1.319064176 | 2.57E-31 | 2.27E-29 | ZBTB4 |
| ENSG00000134321 | 385.4538994 | -1.962261496 | 2.88E-31 | 2.53E-29 | RSAD2 |
| ENSG00000076604 | 609.7819695 | 1.536242154 | 3.17E-31 | 2.78E-29 | TRAF4 |
| ENSG00000018280 | 1433.168949 | 3.362145536 | 5.90E-31 | 5.13E-29 | SLC11A1 |
| ENSG00000188483 | 571.3194026 | 1.716907411 | 6.14E-31 | 5.33E-29 | IER5L |
| ENSG00000075275 | 1331.279886 | 1.142806587 | 7.13E-31 | 6.16E-29 | CELSR1 |
| ENSG00000104312 | 1626.391026 | 1.968985011 | 8.13E-31 | 7.00E-29 | RIPK2 |
| ENSG00000277734 | 865.5829292 | -1.720172951 | 8.65E-31 | 7.40E-29 | TRAC |
| ENSG00000120675 | 703.9603462 | -1.21341357 | 8.80E-31 | 7.50E-29 | DNAJC15 |
| ENSG00000180044 | 799.6836054 | -2.007931435 | 9.01E-31 | 7.65E-29 | C3orf80 |
| ENSG00000116133 | 1478.685845 | -1.039834072 | 1.59E-30 | 1.34E-28 | DHCR24 |
| ENSG00000186635 | 4041.843885 | 1.014150074 | 1.60E-30 | 1.35E-28 | ARAP1 |
| ENSG00000114268 | 1196.30491 | 1.108059357 | 1.96E-30 | 1.65E-28 | PFKFB4 |
| ENSG00000197044 | 184.2450071 | 2.030395563 | 3.14E-30 | 2.62E-28 | ZNF441 |
| ENSG00000213672 | 1060.885036 | 1.068130671 | 3.31E-30 | 2.75E-28 | NCKIPSD |
| ENSG00000171603 | 1544.886573 | -1.056975898 | 6.54E-30 | 5.35E-28 | CLSTN1 |
| ENSG00000150347 | 1005.743508 | 1.078625472 | 6.69E-30 | 5.46E-28 | ARID5B |
| ENSG00000165272 | 89.72326163 | -3.708917745 | 7.01E-30 | 5.70E-28 | AQP3 |
| ENSG00000135631 | 667.6969233 | 1.303687726 | 7.30E-30 | 5.92E-28 | RAB11FIP5 |
| ENSG00000159399 | 4641.436324 | 1.424165443 | 7.56E-30 | 6.10E-28 | HK2 |
| ENSG00000173889 | 2350.173141 | 1.0677174 | 8.40E-30 | 6.76E-28 | PHC3 |
| ENSG00000104549 | 23952.91138 | 1.072453344 | 9.52E-30 | 7.64E-28 | SQLE |
| ENSG00000145936 | 429.018178 | -1.72990373 | 9.72E-30 | 7.77E-28 | KCNMB1 |
| ENSG00000169245 | 78.59051662 | -4.915525971 | 1.06E-29 | 8.48E-28 | CXCL10 |
| ENSG00000173198 | 1403.933385 | -1.285376796 | 1.29E-29 | 1.02E-27 | CYSLTR1 |
| ENSG00000104972 | 254.949921 | -1.997728018 | 1.37E-29 | 1.08E-27 | LILRB1 |
| ENSG00000198520 | 274.3495384 | -2.599334671 | 1.39E-29 | 1.10E-27 | ARMH1 |
| ENSG00000108691 | 124.6442577 | -3.002741106 | 1.54E-29 | 1.21E-27 | CCL2 |
| ENSG00000243335 | 424.3796321 | 1.628084948 | 1.61E-29 | 1.26E-27 | KCTD7 |
| ENSG00000184838 | 407.8084713 | -1.325608744 | 1.70E-29 | 1.32E-27 | PRR16 |
| ENSG00000163516 | 730.8052709 | 1.178096001 | 1.98E-29 | 1.54E-27 | ANKZF1 |
| ENSG00000178607 | 1559.645169 | 1.396023722 | 2.35E-29 | 1.81E-27 | ERN1 |
| ENSG00000163563 | 153.6942873 | -2.324125404 | 2.57E-29 | 1.98E-27 | MNDA |
| ENSG00000116678 | 365.5336528 | 1.944998487 | 3.05E-29 | 2.33E-27 | LEPR |
| ENSG00000089127 | 1991.584085 | -1.369478002 | 3.05E-29 | 2.33E-27 | OAS1 |
| ENSG00000175175 | 1161.197553 | 1.492089346 | 3.10E-29 | 2.36E-27 | PPM1E |
| ENSG00000168078 | 467.2512593 | -1.323859657 | 3.28E-29 | 2.49E-27 | PBK |
| ENSG00000126895 | 201.4488015 | -2.318283201 | 3.86E-29 | 2.91E-27 | AVPR2 |
| ENSG00000175857 | 410.3220793 | -2.336808156 | 5.25E-29 | 3.92E-27 | GAPT |
| ENSG00000106665 | 876.6118213 | 1.288930793 | 5.49E-29 | 4.09E-27 | CLIP2 |
| ENSG00000196975 | 1465.016095 | 1.066950647 | 5.85E-29 | 4.33E-27 | ANXA4 |
| ENSG00000003393 | 1592.168242 | -1.135525696 | 6.32E-29 | 4.65E-27 | ALS2 |
| ENSG00000101445 | 269.1346484 | -1.7150196 | 6.70E-29 | 4.91E-27 | PPP1R16B |
| ENSG00000136478 | 6750.643692 | 1.018750097 | 8.71E-29 | 6.35E-27 | TEX2 |
| ENSG00000130529 | 1114.801646 | 1.398511563 | 9.85E-29 | 7.16E-27 | TRPM4 |
| ENSG00000144840 | 903.7606431 | 1.040965923 | 1.02E-28 | 7.39E-27 | RABL3 |
| ENSG00000137968 | 191.576384 | 2.008245028 | 1.05E-28 | 7.56E-27 | SLC44A5 |
| ENSG00000108256 | 13022.23884 | 1.130065024 | 1.07E-28 | 7.68E-27 | NUFIP2 |
| ENSG00000087903 | 1532.100797 | 1.475209577 | 1.37E-28 | 9.82E-27 | RFX2 |
| ENSG00000099875 | 2192.961248 | 1.471703101 | 1.60E-28 | 1.14E-26 | MKNK2 |
| ENSG00000137509 | 595.2631874 | -1.205838135 | 1.60E-28 | 1.14E-26 | PRCP |
| ENSG00000172216 | 2067.910481 | 1.339199662 | 1.80E-28 | 1.27E-26 | CEBPB |
| ENSG00000103066 | 1540.410168 | 1.345505547 | 1.90E-28 | 1.34E-26 | PLA2G15 |
| ENSG00000083844 | 1024.489694 | 1.122747484 | 1.91E-28 | 1.34E-26 | ZNF264 |
| ENSG00000186470 | 476.77413 | 1.411220605 | 1.93E-28 | 1.35E-26 | BTN3A2 |
| ENSG00000214900 | 229.2597404 | -1.741182778 | 2.25E-28 | 1.57E-26 | LINC01588 |
| ENSG00000075223 | 172.8388695 | 3.226723194 | 2.72E-28 | 1.89E-26 | SEMA3C |
| ENSG00000246363 | 145.7362109 | -2.133752099 | 3.10E-28 | 2.14E-26 | LINC02458 |
| ENSG00000130158 | 1180.621028 | 1.403413737 | 3.23E-28 | 2.22E-26 | DOCK6 |
| ENSG00000075218 | 303.3914085 | -1.71930774 | 3.52E-28 | 2.42E-26 | GTSE1 |
| ENSG00000277443 | 1619.358524 | 1.125321448 | 3.75E-28 | 2.57E-26 | MARCKS |
| ENSG00000149294 | 2261.402014 | 1.254472626 | 4.24E-28 | 2.89E-26 | NCAM1 |
| ENSG00000246859 | 1362.75783 | -1.295066341 | 4.65E-28 | 3.16E-26 | STARD4-AS1 |
| ENSG00000031081 | 412.8861846 | -1.441839313 | 4.93E-28 | 3.34E-26 | ARHGAP31 |
| ENSG00000115828 | 146.8285003 | -2.170019278 | 5.28E-28 | 3.55E-26 | QPCT |
| ENSG00000184221 | 214.694046 | 2.558127175 | 6.89E-28 | 4.61E-26 | OLIG1 |
| ENSG00000170571 | 3310.336381 | -1.559667959 | 7.32E-28 | 4.88E-26 | EMB |
| ENSG00000073417 | 965.4791159 | 1.354224434 | 7.94E-28 | 5.27E-26 | PDE8A |
| ENSG00000261087 | 272.5468192 | 1.572516804 | 9.87E-28 | 6.54E-26 | ZNNT1 |
| ENSG00000145555 | 3574.030111 | 1.331702953 | 1.01E-27 | 6.67E-26 | MYO10 |
| ENSG00000102780 | 4236.686718 | 1.014178904 | 1.02E-27 | 6.75E-26 | DGKH |
| ENSG00000154146 | 556.8248003 | -1.490628896 | 1.06E-27 | 6.99E-26 | NRGN |
| ENSG00000178385 | 638.4409066 | 1.258440673 | 1.36E-27 | 8.89E-26 | PLEKHM3 |
| ENSG00000088827 | 2668.325593 | -1.078149803 | 1.62E-27 | 1.06E-25 | SIGLEC1 |
| ENSG00000135253 | 685.2208984 | 1.294243782 | 1.69E-27 | 1.10E-25 | KCP |
| ENSG00000013583 | 672.5119093 | 1.221282913 | 2.00E-27 | 1.29E-25 | HEBP1 |
| ENSG00000166851 | 456.3203285 | -1.592265596 | 2.15E-27 | 1.39E-25 | PLK1 |
| ENSG00000166398 | 604.4828705 | 1.165631038 | 2.43E-27 | 1.57E-25 | GARRE1 |
| ENSG00000149600 | 493.607051 | -1.391980801 | 3.10E-27 | 2.00E-25 | COMMD7 |
| ENSG00000075884 | 616.7846046 | -1.111956 | 3.19E-27 | 2.05E-25 | ARHGAP15 |
| ENSG00000052802 | 914.6812866 | 1.954014405 | 3.93E-27 | 2.51E-25 | MSMO1 |
| ENSG00000163393 | 1115.635336 | 1.016880709 | 4.95E-27 | 3.15E-25 | SLC22A15 |
| ENSG00000239998 | 72.9125013 | -3.422416063 | 5.76E-27 | 3.66E-25 | LILRA2 |
| ENSG00000255112 | 2239.606322 | 1.01292116 | 6.50E-27 | 4.10E-25 | CHMP1B |
| ENSG00000118495 | 632.4251621 | -1.173493811 | 6.83E-27 | 4.30E-25 | PLAGL1 |
| ENSG00000152223 | 1792.65366 | 1.585109933 | 7.81E-27 | 4.90E-25 | EPG5 |
| ENSG00000129951 | 214.5560402 | -1.857940026 | 8.14E-27 | 5.09E-25 | PLPPR3 |
| ENSG00000128011 | 177.4147848 | 2.022625684 | 9.56E-27 | 5.96E-25 | LRFN1 |
| ENSG00000167900 | 315.0328429 | -1.580387503 | 1.01E-26 | 6.27E-25 | TK1 |
| ENSG00000261371 | 9396.590322 | -1.127675299 | 1.12E-26 | 6.96E-25 | PECAM1 |
| ENSG00000005238 | 869.8301017 | 1.095435254 | 1.51E-26 | 9.35E-25 | FAM214B |
| ENSG00000138111 | 335.7535597 | 1.474792754 | 1.80E-26 | 1.11E-24 | MFSD13A |
| ENSG00000139174 | 132.9494036 | -2.218235616 | 1.84E-26 | 1.13E-24 | PRICKLE1 |
| ENSG00000122966 | 452.2976101 | -1.238960828 | 2.00E-26 | 1.23E-24 | CIT |
| ENSG00000114439 | 827.3253137 | 1.130824441 | 2.01E-26 | 1.23E-24 | BBX |
| ENSG00000047597 | 179.2578893 | -2.475094716 | 2.31E-26 | 1.41E-24 | XK |
| ENSG00000136108 | 818.8648108 | -1.08727895 | 2.33E-26 | 1.42E-24 | CKAP2 |
| ENSG00000133056 | 280.3874016 | -1.718569749 | 2.36E-26 | 1.43E-24 | PIK3C2B |
| ENSG00000103811 | 7503.642004 | 1.239507902 | 2.52E-26 | 1.52E-24 | CTSH |
| ENSG00000113328 | 3324.781074 | 1.006177395 | 2.94E-26 | 1.77E-24 | CCNG1 |
| ENSG00000138119 | 521.6712127 | -1.338934126 | 3.32E-26 | 1.98E-24 | MYOF |
| ENSG00000251022 | 1140.902027 | 1.360121249 | 3.32E-26 | 1.98E-24 | THAP9-AS1 |
| ENSG00000132471 | 3136.624898 | 1.085580761 | 3.41E-26 | 2.03E-24 | WBP2 |
| ENSG00000170191 | 478.8818991 | 1.469004361 | 3.43E-26 | 2.03E-24 | NANP |
| ENSG00000136153 | 231.2729563 | -1.62002708 | 4.03E-26 | 2.39E-24 | LMO7 |
| ENSG00000276975 | 88.16640015 | -2.926068627 | 5.17E-26 | 3.06E-24 | HYDIN2 |
| ENSG00000160932 | 2530.853346 | -1.108936383 | 7.20E-26 | 4.23E-24 | LY6E |
| ENSG00000152056 | 357.6874765 | 1.382392173 | 7.43E-26 | 4.36E-24 | AP1S3 |
| ENSG00000114626 | 489.4131056 | 1.612770108 | 9.46E-26 | 5.52E-24 | ABTB1 |
| ENSG00000112096 | 10437.4354 | 1.008217969 | 1.03E-25 | 6.00E-24 | SOD2 |
| ENSG00000286219 | 497.6989021 | 1.473118039 | 1.20E-25 | 6.94E-24 | NOTCH2NLC |
| ENSG00000211584 | 293.3073325 | 1.839221701 | 1.26E-25 | 7.27E-24 | SLC48A1 |
| ENSG00000145730 | 1061.025341 | 1.02651086 | 1.30E-25 | 7.46E-24 | PAM |
| ENSG00000061676 | 191.385975 | 2.270814132 | 1.51E-25 | 8.63E-24 | NCKAP1 |
| ENSG00000182628 | 579.4954063 | -1.351474294 | 1.81E-25 | 1.03E-23 | SKA2 |
| ENSG00000109103 | 750.3256452 | 1.0671295 | 1.97E-25 | 1.12E-23 | UNC119 |
| ENSG00000110013 | 414.5546186 | 1.284467911 | 2.45E-25 | 1.39E-23 | SIAE |
| ENSG00000089041 | 2838.573694 | 1.013201752 | 2.91E-25 | 1.63E-23 | P2RX7 |
| ENSG00000047249 | 2433.905182 | 1.103258435 | 3.37E-25 | 1.88E-23 | ATP6V1H |
| ENSG00000033867 | 10855.97902 | 1.128793716 | 3.43E-25 | 1.91E-23 | SLC4A7 |
| ENSG00000171724 | 128.099597 | -2.609040132 | 3.51E-25 | 1.95E-23 | VAT1L |
| ENSG00000105492 | 69.05423499 | -5.325089241 | 4.11E-25 | 2.28E-23 | SIGLEC6 |
| ENSG00000171503 | 631.2651939 | 1.160149005 | 4.90E-25 | 2.70E-23 | ETFDH |
| ENSG00000104946 | 3894.170786 | -1.134170125 | 5.33E-25 | 2.94E-23 | TBC1D17 |
| ENSG00000121068 | 274.0544442 | 1.900962948 | 5.35E-25 | 2.94E-23 | TBX2 |
| ENSG00000196588 | 1197.259359 | -1.19976311 | 6.04E-25 | 3.31E-23 | MRTFA |
| ENSG00000143382 | 911.9121227 | 1.736047802 | 7.06E-25 | 3.86E-23 | ADAMTSL4 |
| ENSG00000186318 | 622.7474018 | -1.172544427 | 7.65E-25 | 4.18E-23 | BACE1 |
| ENSG00000261609 | 582.3298695 | 1.346214693 | 7.76E-25 | 4.23E-23 | GAN |
| ENSG00000149428 | 4566.243286 | -1.111753659 | 7.90E-25 | 4.29E-23 | HYOU1 |
| ENSG00000101349 | 121.9665949 | -2.693029061 | 8.04E-25 | 4.36E-23 | PAK5 |
| ENSG00000169679 | 850.5962964 | -1.088888337 | 9.08E-25 | 4.90E-23 | BUB1 |
| ENSG00000166250 | 100.8009438 | 2.87464908 | 1.11E-24 | 5.97E-23 | CLMP |
| ENSG00000138443 | 1500.937931 | -1.178450678 | 1.14E-24 | 6.14E-23 | ABI2 |
| ENSG00000127948 | 1499.529458 | 1.050204671 | 1.46E-24 | 7.79E-23 | POR |
| ENSG00000137203 | 240.8991546 | -1.587984141 | 1.49E-24 | 7.94E-23 | TFAP2A |
| ENSG00000188229 | 2975.677221 | -1.05040423 | 1.57E-24 | 8.37E-23 | TUBB4B |
| ENSG00000178573 | 303.4531205 | 1.665802284 | 1.73E-24 | 9.20E-23 | MAF |
| ENSG00000226445 | 265.7681951 | -1.619684288 | 2.05E-24 | 1.09E-22 | THBS2-AS1 |
| ENSG00000089723 | 98.3283967 | 2.875181687 | 2.06E-24 | 1.09E-22 | OTUB2 |
| ENSG00000171236 | 156.8396445 | -1.899523086 | 2.15E-24 | 1.13E-22 | LRG1 |
| ENSG00000117407 | 126.2474876 | -2.380292487 | 2.15E-24 | 1.13E-22 | ARTN |
| ENSG00000187678 | 223.9251778 | -1.881538146 | 2.23E-24 | 1.17E-22 | SPRY4 |
| ENSG00000169122 | 164.2648262 | 1.992062293 | 2.38E-24 | 1.24E-22 | FAM110B |
| ENSG00000153006 | 1338.817298 | -1.132188754 | 2.63E-24 | 1.37E-22 | SREK1IP1 |
| ENSG00000165140 | 144.1254238 | -2.013298901 | 3.16E-24 | 1.64E-22 | FBP1 |
| ENSG00000110660 | 611.1230009 | -1.274665215 | 3.74E-24 | 1.92E-22 | SLC35F2 |
| ENSG00000228486 | 301.7449596 | -1.444800836 | 3.81E-24 | 1.95E-22 | C2orf92 |
| ENSG00000174738 | 355.8249829 | 2.428418564 | 4.63E-24 | 2.36E-22 | NR1D2 |
| ENSG00000174600 | 63.80151655 | -3.491904074 | 5.22E-24 | 2.65E-22 | CMKLR1 |
| ENSG00000225964 | 142.5362808 | -2.261550406 | 5.53E-24 | 2.79E-22 | NRIR |
| ENSG00000183386 | 182.2639053 | -1.830051342 | 5.99E-24 | 3.02E-22 | FHL3 |
| ENSG00000137877 | 291.4051694 | -1.405475159 | 6.45E-24 | 3.23E-22 | SPTBN5 |
| ENSG00000228013 | 112.6722695 | 2.235113549 | 6.53E-24 | 3.27E-22 | IL6R-AS1 |
| ENSG00000110628 | 558.7612812 | 1.167403103 | 7.25E-24 | 3.61E-22 | SLC22A18 |
| ENSG00000090659 | 2522.809567 | -1.122816668 | 8.28E-24 | 4.12E-22 | CD209 |
| ENSG00000137965 | 743.5714021 | -1.134310177 | 9.08E-24 | 4.51E-22 | IFI44 |
| ENSG00000172594 | 314.4523542 | 2.159477801 | 9.69E-24 | 4.80E-22 | SMPDL3A |
| ENSG00000228340 | 88.8882994 | -2.913533411 | 1.03E-23 | 5.08E-22 | MIR646HG |
| ENSG00000118508 | 483.0431193 | 1.236669298 | 1.80E-23 | 8.87E-22 | RAB32 |
| ENSG00000138642 | 529.1078042 | -1.928874479 | 1.86E-23 | 9.14E-22 | HERC6 |
| ENSG00000276070 | 541.7984081 | -1.571982998 | 1.99E-23 | 9.72E-22 | CCL4L2 |
| ENSG00000196839 | 464.4578873 | 1.104512424 | 2.04E-23 | 9.96E-22 | ADA |
| ENSG00000103647 | 305.9246389 | -1.425950992 | 2.29E-23 | 1.11E-21 | CORO2B |
| ENSG00000143228 | 171.5589386 | -1.772297962 | 2.31E-23 | 1.12E-21 | NUF2 |
| ENSG00000188282 | 179.294973 | 1.926048178 | 3.03E-23 | 1.47E-21 | RUFY4 |
| ENSG00000110400 | 174.5452809 | -1.787405536 | 3.03E-23 | 1.47E-21 | NECTIN1 |
| ENSG00000095002 | 647.2376793 | -1.083710414 | 3.05E-23 | 1.47E-21 | MSH2 |
| ENSG00000118762 | 641.5089367 | -1.011435161 | 3.54E-23 | 1.71E-21 | PKD2 |
| ENSG00000068885 | 205.9045337 | -1.960798461 | 3.66E-23 | 1.76E-21 | IFT80 |
| ENSG00000026950 | 282.5551309 | 2.004871041 | 3.67E-23 | 1.76E-21 | BTN3A1 |
| ENSG00000138160 | 464.5003352 | -1.751756702 | 4.96E-23 | 2.36E-21 | KIF11 |
| ENSG00000198176 | 2233.771079 | -1.397617641 | 5.16E-23 | 2.45E-21 | TFDP1 |
| ENSG00000102007 | 862.9399303 | -1.067814207 | 5.62E-23 | 2.66E-21 | PLP2 |
| ENSG00000164070 | 686.5305023 | 1.688797774 | 5.84E-23 | 2.76E-21 | HSPA4L |
| ENSG00000164081 | 1465.604759 | -1.224402865 | 5.93E-23 | 2.80E-21 | TEX264 |
| ENSG00000030582 | 6863.864605 | 1.046664364 | 6.93E-23 | 3.26E-21 | GRN |
| ENSG00000087245 | 2233.79565 | 1.375347751 | 7.21E-23 | 3.38E-21 | MMP2 |
| ENSG00000079616 | 492.9616816 | -1.074605823 | 7.80E-23 | 3.65E-21 | KIF22 |
| ENSG00000182952 | 776.7907467 | 1.013669036 | 7.86E-23 | 3.67E-21 | HMGN4 |
| ENSG00000113368 | 1781.658538 | -1.489199745 | 8.97E-23 | 4.18E-21 | LMNB1 |
| ENSG00000138078 | 1736.699763 | 1.22330629 | 9.11E-23 | 4.23E-21 | PREPL |
| ENSG00000105849 | 793.2190873 | -1.069364729 | 1.07E-22 | 4.98E-21 | POLR1F |
| ENSG00000113916 | 6007.731288 | 1.071593364 | 1.10E-22 | 5.10E-21 | BCL6 |
| ENSG00000234975 | 317.8711564 | 1.755452641 | 1.28E-22 | 5.89E-21 | FTH1P2 |
| ENSG00000172232 | 766.5201188 | -1.346064131 | 1.28E-22 | 5.91E-21 | AZU1 |
| ENSG00000173575 | 5429.739382 | 1.067119359 | 1.36E-22 | 6.24E-21 | CHD2 |
| ENSG00000232187 | 151.6501766 | 1.959271086 | 1.47E-22 | 6.68E-21 | FTH1P7 |
| ENSG00000042493 | 5286.754449 | -1.00125286 | 1.56E-22 | 7.08E-21 | CAPG |
| ENSG00000159733 | 491.5912089 | 1.326454542 | 2.02E-22 | 9.16E-21 | ZFYVE28 |
| ENSG00000135842 | 1882.332912 | -1.445325414 | 2.11E-22 | 9.53E-21 | NIBAN1 |
| ENSG00000138778 | 468.9387229 | -1.737744177 | 2.11E-22 | 9.53E-21 | CENPE |
| ENSG00000109756 | 911.3930484 | 1.220401257 | 2.14E-22 | 9.66E-21 | RAPGEF2 |
| ENSG00000137812 | 784.8762665 | -1.239010383 | 2.35E-22 | 1.06E-20 | KNL1 |
| ENSG00000126814 | 777.5613247 | 1.127221605 | 2.75E-22 | 1.23E-20 | TRMT5 |
| ENSG00000099783 | 2983.35329 | -1.436513923 | 2.83E-22 | 1.26E-20 | HNRNPM |
| ENSG00000177191 | 393.6432563 | 1.368781945 | 3.29E-22 | 1.46E-20 | B3GNT8 |
| ENSG00000131263 | 4397.817025 | 1.101634593 | 3.30E-22 | 1.46E-20 | RLIM |
| ENSG00000138685 | 206.0244305 | -1.973449425 | 3.40E-22 | 1.50E-20 | FGF2 |
| ENSG00000133106 | 1133.634332 | -1.193693254 | 3.69E-22 | 1.63E-20 | EPSTI1 |
| ENSG00000166928 | 1498.065022 | 1.272184541 | 3.76E-22 | 1.66E-20 | MS4A14 |
| ENSG00000086300 | 203.5450489 | 1.541660392 | 3.82E-22 | 1.68E-20 | SNX10 |
| ENSG00000135821 | 5479.783994 | 1.073563857 | 4.03E-22 | 1.76E-20 | GLUL |
| ENSG00000133805 | 2873.060303 | 1.146314998 | 5.23E-22 | 2.28E-20 | AMPD3 |
| ENSG00000050438 | 508.9717488 | 1.10347296 | 6.09E-22 | 2.64E-20 | SLC4A8 |
| ENSG00000211459 | 9823.310251 | 1.247333392 | 6.29E-22 | 2.72E-20 | MT-RNR1 |
| ENSG00000196155 | 224.467811 | -1.622794167 | 8.11E-22 | 3.48E-20 | PLEKHG4 |
| ENSG00000107130 | 1103.323876 | 1.095314153 | 8.34E-22 | 3.56E-20 | NCS1 |
| ENSG00000164611 | 349.2237482 | -1.296894423 | 8.52E-22 | 3.63E-20 | PTTG1 |
| ENSG00000082781 | 3963.574465 | 1.03294132 | 8.65E-22 | 3.68E-20 | ITGB5 |
| ENSG00000114812 | 70.67834015 | -3.418252712 | 8.73E-22 | 3.71E-20 | VIPR1 |
| ENSG00000155660 | 2224.589131 | -1.052699964 | 8.76E-22 | 3.71E-20 | PDIA4 |
| ENSG00000074706 | 732.7748631 | -1.359812033 | 8.80E-22 | 3.72E-20 | IPCEF1 |
| ENSG00000272841 | 190.3925558 | -1.720557583 | 9.01E-22 | 3.81E-20 | MAP3K4-AS1 |
| ENSG00000166130 | 336.1089619 | -1.366397377 | 9.53E-22 | 4.01E-20 | IKBIP |
| ENSG00000178038 | 543.9613084 | 1.189549778 | 9.58E-22 | 4.03E-20 | ALS2CL |
| ENSG00000159618 | 54.0724216 | -4.073340999 | 9.74E-22 | 4.09E-20 | ADGRG5 |
| ENSG00000154734 | 190.5752794 | -1.946944911 | 1.07E-21 | 4.46E-20 | ADAMTS1 |
| ENSG00000115317 | 669.7488587 | 1.201534204 | 1.09E-21 | 4.54E-20 | HTRA2 |
| ENSG00000277462 | 227.4261268 | 1.433933596 | 1.15E-21 | 4.77E-20 | ZNF670 |
| ENSG00000006747 | 451.0023823 | 1.460762508 | 1.31E-21 | 5.44E-20 | SCIN |
| ENSG00000171608 | 698.2199483 | -1.203829467 | 1.44E-21 | 5.95E-20 | PIK3CD |
| ENSG00000225190 | 1804.815671 | 1.180101269 | 1.46E-21 | 6.02E-20 | PLEKHM1 |
| ENSG00000081189 | 5956.495765 | -1.025430407 | 1.62E-21 | 6.68E-20 | MEF2C |
| ENSG00000136636 | 553.355467 | -1.047312497 | 2.16E-21 | 8.88E-20 | KCTD3 |
| ENSG00000196352 | 690.2894018 | 1.270471495 | 2.74E-21 | 1.11E-19 | CD55 |
| ENSG00000170345 | 4861.847545 | -1.153808832 | 2.80E-21 | 1.13E-19 | FOS |
| ENSG00000101605 | 55.4414194 | 3.330187833 | 2.92E-21 | 1.18E-19 | MYOM1 |
| ENSG00000100439 | 503.8330453 | 1.220306061 | 3.40E-21 | 1.37E-19 | ABHD4 |
| ENSG00000114026 | 827.9250328 | -1.020822538 | 3.73E-21 | 1.50E-19 | OGG1 |
| ENSG00000137807 | 401.6625093 | -1.233223164 | 5.26E-21 | 2.11E-19 | KIF23 |
| ENSG00000111886 | 1194.379294 | -1.250614164 | 5.78E-21 | 2.31E-19 | GABRR2 |
| ENSG00000135919 | 341.5124064 | 1.668199239 | 6.01E-21 | 2.40E-19 | SERPINE2 |
| ENSG00000125384 | 149.3506643 | -1.791618151 | 7.42E-21 | 2.94E-19 | PTGER2 |
| ENSG00000173898 | 180.5871102 | 1.682490135 | 7.65E-21 | 3.03E-19 | SPTBN2 |
| ENSG00000168781 | 295.435159 | -1.489779983 | 8.00E-21 | 3.16E-19 | PPIP5K1 |
| ENSG00000141622 | 97.72728537 | -2.132226346 | 8.04E-21 | 3.17E-19 | RNF165 |
| ENSG00000135549 | 522.9602008 | -1.139888892 | 8.50E-21 | 3.34E-19 | PKIB |
| ENSG00000204524 | 635.6435409 | 1.717357668 | 9.07E-21 | 3.56E-19 | ZNF805 |
| ENSG00000143653 | 443.8868378 | -1.257401294 | 9.47E-21 | 3.70E-19 | SCCPDH |
| ENSG00000275342 | 673.5536697 | -1.156062061 | 1.00E-20 | 3.90E-19 | PRAG1 |
| ENSG00000173530 | 792.5118369 | 1.473194773 | 1.03E-20 | 3.99E-19 | TNFRSF10D |
| ENSG00000124406 | 168.2050727 | 1.810466348 | 1.03E-20 | 3.99E-19 | ATP8A1 |
| ENSG00000119242 | 363.3004623 | -1.370140348 | 1.25E-20 | 4.83E-19 | CCDC92 |
| ENSG00000162543 | 54.22276744 | -3.917118531 | 1.61E-20 | 6.21E-19 | UBXN10 |
| ENSG00000013563 | 455.1849429 | 1.193360843 | 1.86E-20 | 7.14E-19 | DNASE1L1 |
| ENSG00000149635 | 143.1225552 | -2.228077478 | 2.34E-20 | 8.98E-19 | OCSTAMP |
| ENSG00000135912 | 1433.390598 | 1.298796414 | 2.56E-20 | 9.78E-19 | TTLL4 |
| ENSG00000033327 | 1795.916142 | 1.159270128 | 2.58E-20 | 9.86E-19 | GAB2 |
| ENSG00000115155 | 63.10095459 | -3.684954838 | 2.72E-20 | 1.04E-18 | OTOF |
| ENSG00000147465 | 181.985836 | -2.126526614 | 2.85E-20 | 1.08E-18 | STAR |
| ENSG00000135094 | 1142.120548 | 2.99056051 | 3.14E-20 | 1.19E-18 | SDS |
| ENSG00000083223 | 9100.911559 | 1.31546456 | 3.82E-20 | 1.45E-18 | TUT7 |
| ENSG00000168710 | 8474.649992 | 1.079768059 | 4.38E-20 | 1.65E-18 | AHCYL1 |
| ENSG00000234608 | 274.670006 | 1.379888153 | 4.64E-20 | 1.75E-18 | MAPKAPK5-AS1 |
| ENSG00000100234 | 449.8883641 | -1.264379583 | 5.13E-20 | 1.93E-18 | TIMP3 |
| ENSG00000228716 | 548.5139765 | -1.784917569 | 5.24E-20 | 1.97E-18 | DHFR |
| ENSG00000112576 | 1508.150003 | -1.270463927 | 5.61E-20 | 2.10E-18 | CCND3 |
| ENSG00000164615 | 615.5267551 | 1.226689653 | 6.00E-20 | 2.24E-18 | CAMLG |
| ENSG00000004399 | 2558.82059 | 1.07449768 | 6.82E-20 | 2.53E-18 | PLXND1 |
| ENSG00000100625 | 72.41634877 | 3.270108381 | 7.00E-20 | 2.59E-18 | SIX4 |
| ENSG00000156345 | 544.8855815 | 1.374608072 | 7.18E-20 | 2.65E-18 | CDK20 |
| ENSG00000138395 | 46.06963944 | -4.214434953 | 8.31E-20 | 3.05E-18 | CDK15 |
| ENSG00000169218 | 50.57260912 | -4.058403009 | 9.13E-20 | 3.34E-18 | RSPO1 |
| ENSG00000023171 | 1165.19316 | 1.050565967 | 9.77E-20 | 3.55E-18 | GRAMD1B |
| ENSG00000171241 | 420.5931648 | -1.345768109 | 9.96E-20 | 3.62E-18 | SHCBP1 |
| ENSG00000214717 | 401.0643347 | -1.248038715 | 1.10E-19 | 3.97E-18 | ZBED1 |
| ENSG00000197632 | 82.2250982 | -3.003454699 | 1.17E-19 | 4.23E-18 | SERPINB2 |
| ENSG00000264538 | 446.9640304 | -1.050648861 | 1.19E-19 | 4.31E-18 | SUZ12P1 |
| ENSG00000135451 | 179.8543677 | -1.739757295 | 1.21E-19 | 4.37E-18 | TROAP |
| ENSG00000170312 | 514.9364803 | -1.257257709 | 1.29E-19 | 4.64E-18 | CDK1 |
| ENSG00000109805 | 399.1658572 | -1.306494846 | 1.32E-19 | 4.74E-18 | NCAPG |
| ENSG00000182983 | 58.02287724 | -3.189644164 | 1.39E-19 | 4.98E-18 | ZNF662 |
| ENSG00000165175 | 7002.966293 | 2.334834827 | 1.42E-19 | 5.06E-18 | MID1IP1 |
| ENSG00000145107 | 629.0002121 | 1.104891907 | 1.51E-19 | 5.36E-18 | TM4SF19 |
| ENSG00000151458 | 1439.72446 | 1.488517227 | 1.51E-19 | 5.37E-18 | ANKRD50 |
| ENSG00000014257 | 313.4226446 | -1.569992901 | 1.78E-19 | 6.31E-18 | ACP3 |
| ENSG00000160961 | 656.3592423 | 1.017568091 | 2.04E-19 | 7.22E-18 | ZNF333 |
| ENSG00000150637 | 71.81359908 | -2.557312656 | 2.17E-19 | 7.68E-18 | CD226 |
| ENSG00000104679 | 400.9940668 | -1.144749707 | 2.18E-19 | 7.69E-18 | R3HCC1 |
| ENSG00000135074 | 53.05771779 | -3.201501518 | 2.21E-19 | 7.80E-18 | ADAM19 |
| ENSG00000186648 | 93.6155008 | 2.766754371 | 2.61E-19 | 9.16E-18 | CARMIL3 |
| ENSG00000073464 | 286.3224809 | -1.827749754 | 2.88E-19 | 1.01E-17 | CLCN4 |
| ENSG00000197965 | 330.5880487 | 1.201436403 | 3.13E-19 | 1.09E-17 | MPZL1 |
| ENSG00000067842 | 59.55692589 | -3.235755263 | 3.99E-19 | 1.38E-17 | ATP2B3 |
| ENSG00000116016 | 865.0369313 | 2.830895548 | 4.08E-19 | 1.41E-17 | EPAS1 |
| ENSG00000100344 | 375.1343478 | 1.144426214 | 4.21E-19 | 1.45E-17 | PNPLA3 |
| ENSG00000184752 | 358.7015446 | -1.133235543 | 4.40E-19 | 1.51E-17 | NDUFA12 |
| ENSG00000135749 | 226.910344 | -1.751772984 | 4.98E-19 | 1.70E-17 | PCNX2 |
| ENSG00000136286 | 221.0367908 | -1.494123333 | 5.22E-19 | 1.78E-17 | MYO1G |
| ENSG00000008083 | 2322.31287 | 1.160829637 | 5.40E-19 | 1.84E-17 | JARID2 |
| ENSG00000198814 | 1431.893275 | 1.239469453 | 6.33E-19 | 2.14E-17 | GK |
| ENSG00000078699 | 922.3588378 | 1.213879903 | 6.58E-19 | 2.22E-17 | CBFA2T2 |
| ENSG00000060982 | 5802.353437 | 1.291591309 | 7.89E-19 | 2.66E-17 | BCAT1 |
| ENSG00000173083 | 185.4526526 | -1.61694694 | 8.02E-19 | 2.70E-17 | HPSE |
| ENSG00000179300 | 45.56685672 | -3.574943837 | 8.11E-19 | 2.72E-17 | RTL3 |
| ENSG00000113296 | 56.16967533 | -3.240214301 | 8.39E-19 | 2.82E-17 | THBS4 |
| ENSG00000145349 | 2521.618388 | 1.000383893 | 8.85E-19 | 2.96E-17 | CAMK2D |
| ENSG00000133597 | 423.715518 | -1.412645716 | 1.11E-18 | 3.70E-17 | ADCK2 |
| ENSG00000066279 | 557.956656 | -1.309815035 | 1.12E-18 | 3.74E-17 | ASPM |
| ENSG00000118922 | 89.64091926 | -2.733835283 | 1.28E-18 | 4.27E-17 | KLF12 |
| ENSG00000166762 | 169.4369651 | -1.605926269 | 1.39E-18 | 4.61E-17 | CATSPER2 |
| ENSG00000185278 | 1419.588725 | 1.043922615 | 1.66E-18 | 5.48E-17 | ZBTB37 |
| ENSG00000158296 | 50.9466515 | -3.299735921 | 1.71E-18 | 5.65E-17 | SLC13A3 |
| ENSG00000288558 | 94.72988638 | -2.026647457 | 1.92E-18 | 6.31E-17 | DUS4L-BCAP29 |
| ENSG00000101695 | 117.9230868 | -2.01206571 | 1.98E-18 | 6.48E-17 | RNF125 |
| ENSG00000135587 | 415.682299 | 1.123126522 | 2.05E-18 | 6.71E-17 | SMPD2 |
| ENSG00000173848 | 305.295506 | -1.284603516 | 2.10E-18 | 6.87E-17 | NET1 |
| ENSG00000168300 | 981.0594561 | 1.018342835 | 2.63E-18 | 8.52E-17 | PCMTD1 |
| ENSG00000114054 | 782.2511774 | 1.011053682 | 2.63E-18 | 8.52E-17 | PCCB |
| ENSG00000185650 | 3931.96134 | 1.185688281 | 2.72E-18 | 8.79E-17 | ZFP36L1 |
| ENSG00000184232 | 702.6278728 | -1.001669878 | 2.86E-18 | 9.24E-17 | OAF |
| ENSG00000167680 | 192.0520821 | 1.41427502 | 3.17E-18 | 1.02E-16 | SEMA6B |
| ENSG00000058668 | 867.9238689 | -1.041495679 | 3.58E-18 | 1.15E-16 | ATP2B4 |
| ENSG00000132688 | 152.5012771 | -1.989613101 | 3.88E-18 | 1.24E-16 | NES |
| ENSG00000119922 | 316.4669122 | -1.459098387 | 3.91E-18 | 1.25E-16 | IFIT2 |
| ENSG00000135241 | 631.2723711 | 1.090626463 | 4.36E-18 | 1.39E-16 | PNPLA8 |
| ENSG00000136490 | 340.0288513 | -1.623778295 | 5.02E-18 | 1.60E-16 | LIMD2 |
| ENSG00000151692 | 175.2855557 | -1.554915679 | 5.38E-18 | 1.70E-16 | RNF144A |
| ENSG00000108950 | 175.2385187 | 2.20780427 | 5.94E-18 | 1.87E-16 | FAM20A |
| ENSG00000152684 | 353.5498031 | 1.120225209 | 6.07E-18 | 1.91E-16 | PELO |
| ENSG00000164284 | 478.5192684 | 1.02762817 | 6.24E-18 | 1.96E-16 | GRPEL2 |
| ENSG00000119280 | 361.7889582 | 1.063953638 | 6.57E-18 | 2.06E-16 | C1orf198 |
| ENSG00000175567 | 637.6122927 | 1.169728574 | 6.92E-18 | 2.16E-16 | UCP2 |
| ENSG00000153485 | 431.2728052 | 1.086723024 | 7.12E-18 | 2.22E-16 | TMEM251 |
| ENSG00000113369 | 3483.959886 | 3.012770572 | 8.58E-18 | 2.66E-16 | ARRDC3 |
| ENSG00000186577 | 422.3744607 | 1.142027435 | 9.22E-18 | 2.85E-16 | SMIM29 |
| ENSG00000225855 | 448.1031966 | 1.554850443 | 9.37E-18 | 2.90E-16 | RUSC1-AS1 |
| ENSG00000171425 | 280.6099063 | 1.281966827 | 9.73E-18 | 3.00E-16 | ZNF581 |
| ENSG00000103245 | 407.0139784 | 1.070944311 | 1.00E-17 | 3.09E-16 | CIAO3 |
| ENSG00000166949 | 918.6123809 | -1.007225779 | 1.03E-17 | 3.17E-16 | SMAD3 |
| ENSG00000106546 | 485.894252 | 1.114183414 | 1.16E-17 | 3.57E-16 | AHR |
| ENSG00000174804 | 104.7973936 | 1.978888403 | 1.43E-17 | 4.38E-16 | FZD4 |
| ENSG00000105011 | 260.2743148 | -1.962407272 | 1.82E-17 | 5.51E-16 | ASF1B |
| ENSG00000024526 | 115.247604 | -2.091504852 | 1.88E-17 | 5.69E-16 | DEPDC1 |
| ENSG00000198838 | 63.83705726 | -2.654752971 | 1.91E-17 | 5.76E-16 | RYR3 |
| ENSG00000150977 | 1744.192626 | 1.026601263 | 1.95E-17 | 5.89E-16 | RILPL2 |
| ENSG00000205045 | 140.183074 | -1.708894393 | 2.06E-17 | 6.20E-16 | SLFN12L |
| ENSG00000140961 | 121.8104134 | 1.680402786 | 2.19E-17 | 6.59E-16 | OSGIN1 |
| ENSG00000121858 | 59.77812634 | -2.985542078 | 2.45E-17 | 7.32E-16 | TNFSF10 |
| ENSG00000247982 | 694.3179432 | -1.377104077 | 2.47E-17 | 7.38E-16 | LINC00926 |
| ENSG00000163482 | 363.8702947 | -1.176172966 | 2.52E-17 | 7.50E-16 | STK36 |
| ENSG00000163389 | 245.7193046 | -1.721045674 | 2.53E-17 | 7.51E-16 | POGLUT1 |
| ENSG00000224043 | 137.6796348 | 1.740046195 | 2.64E-17 | 7.83E-16 | CCNT2-AS1 |
| ENSG00000090621 | 27083.05477 | 1.018782202 | 2.66E-17 | 7.88E-16 | PABPC4 |
| ENSG00000113249 | 402.5817548 | 1.584795833 | 2.70E-17 | 7.99E-16 | HAVCR1 |
| ENSG00000105835 | 2151.565633 | 1.095352189 | 2.71E-17 | 8.03E-16 | NAMPT |
| ENSG00000187498 | 120.1862378 | -1.768898324 | 2.79E-17 | 8.24E-16 | COL4A1 |
| ENSG00000250479 | 251.0040704 | 1.612795113 | 2.85E-17 | 8.40E-16 | CHCHD10 |
| ENSG00000100523 | 1224.53518 | 1.167578572 | 3.13E-17 | 9.22E-16 | DDHD1 |
| ENSG00000187608 | 394.0859707 | -1.492021441 | 3.28E-17 | 9.63E-16 | ISG15 |
| ENSG00000187764 | 511.8271884 | -1.032640157 | 3.73E-17 | 1.09E-15 | SEMA4D |
| ENSG00000143061 | 93.42519403 | -2.151231609 | 3.75E-17 | 1.10E-15 | IGSF3 |
| ENSG00000039560 | 2601.060376 | 1.111070615 | 3.93E-17 | 1.15E-15 | RAI14 |
| ENSG00000149547 | 1755.241735 | -1.172704304 | 5.19E-17 | 1.50E-15 | EI24 |
| ENSG00000101966 | 2970.133819 | 1.166152801 | 5.48E-17 | 1.58E-15 | XIAP |
| ENSG00000173706 | 1747.329764 | -1.29722776 | 5.73E-17 | 1.65E-15 | HEG1 |
| ENSG00000092969 | 110.31715 | 1.942434817 | 5.99E-17 | 1.72E-15 | TGFB2 |
| ENSG00000166394 | 156.0042302 | -1.574765996 | 6.22E-17 | 1.78E-15 | CYB5R2 |
| ENSG00000163536 | 108.0078003 | 1.939449742 | 6.87E-17 | 1.96E-15 | SERPINI1 |
| ENSG00000065675 | 73.62370654 | -2.320691538 | 8.11E-17 | 2.30E-15 | PRKCQ |
| ENSG00000152217 | 289.5844679 | -1.256092295 | 8.42E-17 | 2.39E-15 | SETBP1 |
| ENSG00000188177 | 465.8812616 | 1.585476999 | 8.84E-17 | 2.50E-15 | ZC3H6 |
| ENSG00000142945 | 403.5158258 | -1.298977325 | 9.68E-17 | 2.73E-15 | KIF2C |
| ENSG00000141458 | 2617.862914 | 1.005845809 | 9.85E-17 | 2.78E-15 | NPC1 |
| ENSG00000278129 | 455.9802647 | 1.033225345 | 1.17E-16 | 3.27E-15 | ZNF8 |
| ENSG00000152778 | 262.01663 | -1.623685109 | 1.31E-16 | 3.67E-15 | IFIT5 |
| ENSG00000085563 | 221.699918 | -1.246046304 | 1.32E-16 | 3.68E-15 | ABCB1 |
| ENSG00000089060 | 787.7632797 | 1.022320422 | 1.38E-16 | 3.86E-15 | SLC8B1 |
| ENSG00000184979 | 305.3885572 | -1.990750138 | 1.44E-16 | 4.02E-15 | USP18 |
| ENSG00000151575 | 65.79645074 | -2.988652279 | 1.55E-16 | 4.31E-15 | TEX9 |
| ENSG00000087076 | 123.5518118 | 1.976947446 | 1.63E-16 | 4.53E-15 | HSD17B14 |
| ENSG00000019102 | 368.4024671 | -1.402681956 | 1.66E-16 | 4.60E-15 | VSIG2 |
| ENSG00000134057 | 626.6012977 | -1.383317947 | 1.68E-16 | 4.64E-15 | CCNB1 |
| ENSG00000107438 | 96.2114371 | -2.217760585 | 1.73E-16 | 4.78E-15 | PDLIM1 |
| ENSG00000182963 | 351.5905116 | 1.222827435 | 1.77E-16 | 4.90E-15 | GJC1 |
| ENSG00000234771 | 290.7381187 | -1.18380821 | 1.86E-16 | 5.12E-15 | SLC25A25-AS1 |
| ENSG00000157240 | 117.1157698 | -1.814601811 | 2.17E-16 | 5.93E-15 | FZD1 |
| ENSG00000164938 | 245.6081946 | 1.323018364 | 2.24E-16 | 6.10E-15 | TP53INP1 |
| ENSG00000088305 | 171.6724814 | -1.561488224 | 2.59E-16 | 7.04E-15 | DNMT3B |
| ENSG00000197119 | 456.7845307 | 1.711143204 | 2.62E-16 | 7.12E-15 | SLC25A29 |
| ENSG00000111206 | 313.0638653 | -1.370555877 | 2.74E-16 | 7.43E-15 | FOXM1 |
| ENSG00000259207 | 1606.561528 | 1.281275731 | 3.03E-16 | 8.19E-15 | ITGB3 |
| ENSG00000232810 | 667.3121379 | -1.573552116 | 3.09E-16 | 8.33E-15 | TNF |
| ENSG00000136720 | 334.8613181 | -1.523237857 | 3.43E-16 | 9.22E-15 | HS6ST1 |
| ENSG00000129116 | 513.0999161 | 1.405694644 | 3.81E-16 | 1.02E-14 | PALLD |
| ENSG00000136315 | 49.08425498 | -3.01857986 | 4.70E-16 | 1.26E-14 | RNASE2CP |
| ENSG00000003436 | 250.3217357 | 1.579728709 | 4.73E-16 | 1.26E-14 | TFPI |
| ENSG00000158769 | 617.6461359 | 1.391256883 | 4.76E-16 | 1.27E-14 | F11R |
| ENSG00000156711 | 928.740097 | 1.208226146 | 4.77E-16 | 1.27E-14 | MAPK13 |
| ENSG00000131668 | 353.2716575 | -1.112422975 | 5.13E-16 | 1.36E-14 | BARX1 |
| ENSG00000103966 | 1121.183688 | -1.296902805 | 5.30E-16 | 1.40E-14 | EHD4 |
| ENSG00000179222 | 387.5148706 | -1.298143468 | 5.55E-16 | 1.46E-14 | MAGED1 |
| ENSG00000100075 | 913.068519 | 1.147942573 | 5.75E-16 | 1.51E-14 | SLC25A1 |
| ENSG00000007384 | 851.2386685 | 1.361400829 | 6.30E-16 | 1.65E-14 | RHBDF1 |
| ENSG00000229644 | 182.6620408 | 1.294980463 | 7.07E-16 | 1.85E-14 | NAMPTP1 |
| ENSG00000133247 | 315.4810877 | 1.086905246 | 7.23E-16 | 1.89E-14 | KMT5C |
| ENSG00000256268 | 59.35592862 | -2.565999786 | 8.10E-16 | 2.11E-14 | LINC02454 |
| ENSG00000224116 | 144.0848589 | -1.679268518 | 8.79E-16 | 2.29E-14 | INHBA-AS1 |
| ENSG00000173041 | 139.6225173 | 1.682079712 | 9.51E-16 | 2.47E-14 | ZNF680 |
| ENSG00000229754 | 85.95159073 | 1.937023023 | 9.51E-16 | 2.47E-14 | CXCR2P1 |
| ENSG00000111331 | 6157.68745 | -1.143706439 | 1.03E-15 | 2.66E-14 | OAS3 |
| ENSG00000163960 | 3633.035057 | 1.199584372 | 1.03E-15 | 2.68E-14 | UBXN7 |
| ENSG00000108846 | 350.3595836 | 1.857550924 | 1.08E-15 | 2.78E-14 | ABCC3 |
| ENSG00000000938 | 1328.762506 | 1.022352066 | 1.24E-15 | 3.19E-14 | FGR |
| ENSG00000121005 | 133.8196823 | -1.704809213 | 1.32E-15 | 3.39E-14 | CRISPLD1 |
| ENSG00000164124 | 203.6788386 | 1.800660567 | 1.45E-15 | 3.70E-14 | TMEM144 |
| ENSG00000184164 | 411.5620585 | -1.203006055 | 1.45E-15 | 3.70E-14 | CRELD2 |
| ENSG00000182253 | 143.8609546 | -1.570308908 | 1.47E-15 | 3.74E-14 | SYNM |
| ENSG00000196357 | 144.1031802 | 1.647358898 | 1.52E-15 | 3.86E-14 | ZNF565 |
| ENSG00000116761 | 52.04930395 | 2.700018654 | 1.53E-15 | 3.90E-14 | CTH |
| ENSG00000071539 | 331.9680546 | -1.36110036 | 1.55E-15 | 3.93E-14 | TRIP13 |
| ENSG00000168944 | 326.9040065 | -1.065361533 | 1.56E-15 | 3.94E-14 | CEP120 |
| ENSG00000153162 | 47.75342469 | 4.131997825 | 1.58E-15 | 4.01E-14 | BMP6 |
| ENSG00000177000 | 2040.330644 | 1.18654203 | 1.61E-15 | 4.08E-14 | MTHFR |
| ENSG00000172889 | 282.7423648 | 1.243195669 | 1.63E-15 | 4.12E-14 | EGFL7 |
| ENSG00000154309 | 554.9139868 | 1.147670923 | 1.67E-15 | 4.21E-14 | DISP1 |
| ENSG00000198556 | 525.5974459 | -1.228103242 | 1.93E-15 | 4.83E-14 | ZNF789 |
| ENSG00000176890 | 514.262399 | -1.144222957 | 2.25E-15 | 5.60E-14 | TYMS |
| ENSG00000126787 | 244.9687292 | -1.315215004 | 2.31E-15 | 5.75E-14 | DLGAP5 |
| ENSG00000158859 | 43.49889069 | -3.426606232 | 2.31E-15 | 5.75E-14 | ADAMTS4 |
| ENSG00000109743 | 175.737598 | -1.613039722 | 2.51E-15 | 6.22E-14 | BST1 |
| ENSG00000276043 | 636.8332273 | -1.288952694 | 2.51E-15 | 6.23E-14 | UHRF1 |
| ENSG00000172379 | 643.7648686 | -1.358910971 | 2.68E-15 | 6.63E-14 | ARNT2 |
| ENSG00000162976 | 326.6370593 | 1.171908604 | 2.72E-15 | 6.73E-14 | SLC66A3 |
| ENSG00000128512 | 1060.473194 | 1.016846712 | 2.76E-15 | 6.80E-14 | DOCK4 |
| ENSG00000011132 | 474.1710758 | 1.026047263 | 2.76E-15 | 6.80E-14 | APBA3 |
| ENSG00000151611 | 114.194629 | 1.774433076 | 2.81E-15 | 6.90E-14 | MMAA |
| ENSG00000265148 | 272.1396101 | -1.158011903 | 2.82E-15 | 6.94E-14 | TSPOAP1-AS1 |
| ENSG00000099308 | 1289.642031 | 1.065391638 | 3.06E-15 | 7.51E-14 | MAST3 |
| ENSG00000165102 | 3026.036276 | -1.062766214 | 3.11E-15 | 7.62E-14 | HGSNAT |
| ENSG00000093009 | 209.7317587 | -1.512820825 | 3.25E-15 | 7.95E-14 | CDC45 |
| ENSG00000214174 | 135.2492234 | 1.487108715 | 3.32E-15 | 8.10E-14 | AMZ2P1 |
| ENSG00000099725 | 150.8465014 | -1.56738974 | 3.35E-15 | 8.17E-14 | PRKY |
| ENSG00000034693 | 160.1910094 | -1.400454724 | 3.82E-15 | 9.28E-14 | PEX3 |
| ENSG00000175352 | 1719.664091 | 1.01587844 | 4.02E-15 | 9.75E-14 | NRIP3 |
| ENSG00000163634 | 444.7296091 | -1.05029648 | 5.16E-15 | 1.24E-13 | THOC7 |
| ENSG00000197555 | 250.9626238 | 1.397273973 | 5.44E-15 | 1.31E-13 | SIPA1L1 |
| ENSG00000128917 | 137.9979497 | -1.951051338 | 6.10E-15 | 1.46E-13 | DLL4 |
| ENSG00000126005 | 257.3348691 | 1.104568249 | 6.24E-15 | 1.49E-13 | MMP24OS |
| ENSG00000101057 | 877.080255 | -1.34091137 | 6.24E-15 | 1.49E-13 | MYBL2 |
| ENSG00000142156 | 934.6800916 | 1.308383785 | 6.39E-15 | 1.52E-13 | COL6A1 |
| ENSG00000132434 | 236.1686148 | -1.326298727 | 6.47E-15 | 1.54E-13 | LANCL2 |
| ENSG00000118096 | 87.87597496 | -1.944231232 | 6.79E-15 | 1.61E-13 | IFT46 |
| ENSG00000197714 | 227.5577052 | 1.158944406 | 8.66E-15 | 2.05E-13 | ZNF460 |
| ENSG00000187837 | 156.7583167 | 1.587347463 | 9.60E-15 | 2.27E-13 | H1-2 |
| ENSG00000173210 | 118.5780666 | 1.569544813 | 9.70E-15 | 2.29E-13 | ABLIM3 |
| ENSG00000166451 | 264.3846886 | -1.205594654 | 1.24E-14 | 2.90E-13 | CENPN |
| ENSG00000164308 | 394.0739473 | 1.005975776 | 1.24E-14 | 2.90E-13 | ERAP2 |
| ENSG00000008277 | 82.57190993 | -1.98790423 | 1.41E-14 | 3.27E-13 | ADAM22 |
| ENSG00000106692 | 464.7236487 | -1.25782264 | 1.43E-14 | 3.31E-13 | FKTN |
| ENSG00000118473 | 851.5502799 | -1.134659859 | 1.47E-14 | 3.40E-13 | SGIP1 |
| ENSG00000034677 | 3505.98888 | 1.00341305 | 1.50E-14 | 3.47E-13 | RNF19A |
| ENSG00000196684 | 263.2971755 | -1.65749282 | 1.60E-14 | 3.69E-13 | HSH2D |
| ENSG00000273033 | 192.2043 | 1.44036536 | 1.87E-14 | 4.29E-13 | LINC02035 |
| ENSG00000186628 | 78.02233942 | 3.109746199 | 1.94E-14 | 4.44E-13 | FSD2 |
| ENSG00000119986 | 129.9404404 | 1.463344814 | 2.01E-14 | 4.59E-13 | AVPI1 |
| ENSG00000117298 | 130.3321597 | -1.815919061 | 2.10E-14 | 4.80E-13 | ECE1 |
| ENSG00000151498 | 465.1809434 | -1.034822817 | 2.14E-14 | 4.88E-13 | ACAD8 |
| ENSG00000172009 | 739.3492858 | -1.166209964 | 2.20E-14 | 4.99E-13 | THOP1 |
| ENSG00000128228 | 293.4462167 | -1.158156027 | 2.24E-14 | 5.08E-13 | SDF2L1 |
| ENSG00000147364 | 289.9814892 | -1.015271521 | 2.25E-14 | 5.09E-13 | FBXO25 |
| ENSG00000102524 | 257.1917317 | -1.349768011 | 2.31E-14 | 5.22E-13 | TNFSF13B |
| ENSG00000196428 | 784.4443487 | 1.107548441 | 2.36E-14 | 5.34E-13 | TSC22D2 |
| ENSG00000108405 | 121.72952 | -1.65036559 | 2.40E-14 | 5.43E-13 | P2RX1 |
| ENSG00000205534 | 82.13181894 | 2.085560924 | 2.49E-14 | 5.61E-13 | SMG1P2 |
| ENSG00000117594 | 52.4282736 | -2.388772036 | 2.60E-14 | 5.86E-13 | HSD11B1 |
| ENSG00000081870 | 234.9109892 | -1.329016512 | 2.78E-14 | 6.25E-13 | HSPB11 |
| ENSG00000147614 | 39.43590939 | 3.403066398 | 2.90E-14 | 6.51E-13 | ATP6V0D2 |
| ENSG00000138463 | 163.2875503 | 1.486280688 | 3.32E-14 | 7.39E-13 | SLC49A4 |
| ENSG00000145623 | 77.8616603 | -1.918270567 | 3.33E-14 | 7.42E-13 | OSMR |
| ENSG00000148700 | 295.9907157 | -1.132577967 | 3.39E-14 | 7.54E-13 | ADD3 |
| ENSG00000169220 | 133.8516818 | -1.444153549 | 3.57E-14 | 7.93E-13 | RGS14 |
| ENSG00000219507 | 65.79061011 | 2.294628961 | 3.61E-14 | 8.01E-13 | FTH1P8 |
| ENSG00000111266 | 170.230005 | 1.334658481 | 3.78E-14 | 8.37E-13 | DUSP16 |
| ENSG00000056277 | 289.427605 | 1.14243885 | 4.06E-14 | 8.97E-13 | ZNF280C |
| ENSG00000184156 | 2103.764518 | 1.251631127 | 4.14E-14 | 9.13E-13 | KCNQ3 |
| ENSG00000189362 | 247.0149282 | -1.080354051 | 4.50E-14 | 9.89E-13 | NEMP2 |
| ENSG00000130956 | 197.3076948 | 1.298288016 | 4.52E-14 | 9.92E-13 | HABP4 |
| ENSG00000112511 | 1018.011439 | 1.008418587 | 4.54E-14 | 9.96E-13 | PHF1 |
| ENSG00000188191 | 154.6404476 | -1.461937926 | 4.65E-14 | 1.02E-12 | PRKAR1B |
| ENSG00000135525 | 88.61986087 | -1.7159637 | 5.15E-14 | 1.12E-12 | MAP7 |
| ENSG00000010818 | 290.3104822 | 1.325787141 | 5.44E-14 | 1.18E-12 | HIVEP2 |
| ENSG00000120697 | 690.5354093 | -1.155092729 | 5.63E-14 | 1.22E-12 | ALG5 |
| ENSG00000020577 | 156.0067374 | 1.326636095 | 6.71E-14 | 1.45E-12 | SAMD4A |
| ENSG00000100292 | 7350.243024 | 2.720233514 | 6.88E-14 | 1.48E-12 | HMOX1 |
| ENSG00000136319 | 129.8346627 | -1.489400314 | 7.30E-14 | 1.57E-12 | TTC5 |
| ENSG00000141956 | 464.7682183 | 1.038345835 | 9.62E-14 | 2.05E-12 | PRDM15 |
| ENSG00000059728 | 2167.861605 | 1.192122963 | 9.84E-14 | 2.10E-12 | MXD1 |
| ENSG00000183486 | 2788.324532 | -1.042142931 | 1.05E-13 | 2.24E-12 | MX2 |
| ENSG00000166068 | 403.5812274 | -1.519345798 | 1.18E-13 | 2.48E-12 | SPRED1 |
| ENSG00000162413 | 403.0276033 | 1.078917638 | 1.39E-13 | 2.92E-12 | KLHL21 |
| ENSG00000137710 | 1093.278641 | 1.010703018 | 1.41E-13 | 2.96E-12 | RDX |
| ENSG00000110047 | 754.4106489 | -1.134333554 | 1.47E-13 | 3.08E-12 | EHD1 |
| ENSG00000100321 | 363.6092612 | -1.198513378 | 1.48E-13 | 3.10E-12 | SYNGR1 |
| ENSG00000224916 | 322.3254977 | 1.129517787 | 1.49E-13 | 3.12E-12 | APOC4-APOC2 |
| ENSG00000120306 | 291.1119787 | 1.635972893 | 1.52E-13 | 3.17E-12 | CYSTM1 |
| ENSG00000186812 | 544.3622266 | 1.045918436 | 1.58E-13 | 3.29E-12 | ZNF397 |
| ENSG00000178568 | 212.4572289 | 1.433000098 | 1.67E-13 | 3.47E-12 | ERBB4 |
| ENSG00000175832 | 211.659053 | -1.84061072 | 1.71E-13 | 3.56E-12 | ETV4 |
| ENSG00000248092 | 292.4964353 | 1.032456502 | 2.19E-13 | 4.50E-12 | NNT-AS1 |
| ENSG00000136717 | 2327.531594 | 1.068793906 | 2.49E-13 | 5.09E-12 | BIN1 |
| ENSG00000078687 | 281.7578029 | -1.01678898 | 2.56E-13 | 5.23E-12 | TNRC6C |
| ENSG00000172086 | 592.4952173 | -1.077685666 | 2.87E-13 | 5.82E-12 | KRCC1 |
| ENSG00000152154 | 47.99159208 | 2.816771254 | 2.95E-13 | 5.98E-12 | TMEM178A |
| ENSG00000275302 | 961.6814097 | -1.019473392 | 3.47E-13 | 7.00E-12 | CCL4 |
| ENSG00000142731 | 282.6756632 | -1.093220101 | 3.90E-13 | 7.83E-12 | PLK4 |
| ENSG00000112276 | 296.4898276 | -1.149346491 | 4.06E-13 | 8.14E-12 | BVES |
| ENSG00000182676 | 1081.149682 | 1.126366397 | 4.09E-13 | 8.18E-12 | PPP1R27 |
| ENSG00000090238 | 379.9484735 | 1.083652136 | 4.41E-13 | 8.81E-12 | YPEL3 |
| ENSG00000196074 | 117.323533 | -1.440660355 | 4.41E-13 | 8.81E-12 | SYCP2 |
| ENSG00000253210 | 368.4539897 | -1.256033564 | 4.50E-13 | 8.99E-12 | DENND3-AS1 |
| ENSG00000260852 | 592.0936932 | 1.1147824 | 5.08E-13 | 1.01E-11 | FBXL19-AS1 |
| ENSG00000141232 | 928.6043934 | 1.160987694 | 5.09E-13 | 1.01E-11 | TOB1 |
| ENSG00000133739 | 150.3792289 | -1.405289927 | 5.14E-13 | 1.02E-11 | LRRCC1 |
| ENSG00000166428 | 293.830673 | -1.027042365 | 5.28E-13 | 1.05E-11 | PLD4 |
| ENSG00000115840 | 211.261156 | -1.237624838 | 5.29E-13 | 1.05E-11 | SLC25A12 |
| ENSG00000188747 | 111.5774728 | 1.691248945 | 5.55E-13 | 1.10E-11 | NOXA1 |
| ENSG00000149289 | 8321.016241 | 1.023141538 | 5.88E-13 | 1.16E-11 | ZC3H12C |
| ENSG00000103253 | 273.8592089 | 1.177044783 | 5.89E-13 | 1.16E-11 | HAGHL |
| ENSG00000103187 | 3859.648814 | -1.06491621 | 5.92E-13 | 1.16E-11 | COTL1 |
| ENSG00000072952 | 30.12287512 | -3.559803479 | 6.39E-13 | 1.25E-11 | IRAG1 |
| ENSG00000170892 | 298.2632798 | 1.03006208 | 6.51E-13 | 1.28E-11 | TSEN34 |
| ENSG00000157873 | 219.7184011 | 1.58292594 | 7.07E-13 | 1.38E-11 | TNFRSF14 |
| ENSG00000163082 | 91.70662681 | -2.446775116 | 7.07E-13 | 1.38E-11 | SGPP2 |
| ENSG00000121741 | 2818.966115 | 1.057539538 | 7.38E-13 | 1.44E-11 | ZMYM2 |
| ENSG00000101216 | 571.5189848 | 1.243755796 | 7.44E-13 | 1.45E-11 | GMEB2 |
| ENSG00000094804 | 386.0242885 | -1.005806233 | 9.02E-13 | 1.74E-11 | CDC6 |
| ENSG00000151632 | 3458.600801 | -1.84114141 | 9.05E-13 | 1.75E-11 | AKR1C2 |
| ENSG00000198754 | 295.9597167 | -1.051239438 | 9.18E-13 | 1.77E-11 | OXCT2 |
| ENSG00000099194 | 12953.06519 | 1.673623733 | 9.25E-13 | 1.78E-11 | SCD |
| ENSG00000112759 | 1398.607074 | 1.037722175 | 1.04E-12 | 2.00E-11 | SLC29A1 |
| ENSG00000130590 | 84.17825434 | -1.874427956 | 1.06E-12 | 2.03E-11 | SAMD10 |
| ENSG00000196083 | 284.349224 | -1.16385408 | 1.06E-12 | 2.03E-11 | IL1RAP |
| ENSG00000115841 | 74.5109374 | -2.20022359 | 1.07E-12 | 2.05E-11 | RMDN2 |
| ENSG00000118257 | 2142.109474 | 1.050789563 | 1.11E-12 | 2.12E-11 | NRP2 |
| ENSG00000203791 | 187.3112823 | -1.208354098 | 1.24E-12 | 2.37E-11 | EEF1AKMT2 |
| ENSG00000141965 | 238.5538532 | -1.090881233 | 1.27E-12 | 2.42E-11 | FEM1A |
| ENSG00000038945 | 4686.539472 | 1.047671369 | 1.30E-12 | 2.47E-11 | MSR1 |
| ENSG00000088256 | 200.30559 | -1.128275691 | 1.30E-12 | 2.48E-11 | GNA11 |
| ENSG00000106789 | 485.0307079 | 1.1524652 | 1.33E-12 | 2.52E-11 | CORO2A |
| ENSG00000204397 | 83.4463441 | 1.659113444 | 1.34E-12 | 2.54E-11 | CARD16 |
| ENSG00000111907 | 51.70504231 | 2.882038209 | 1.46E-12 | 2.77E-11 | TPD52L1 |
| ENSG00000105856 | 727.1075284 | 1.009083632 | 1.56E-12 | 2.95E-11 | HBP1 |
| ENSG00000172578 | 3212.933438 | 1.853874744 | 1.69E-12 | 3.18E-11 | KLHL6 |
| ENSG00000172031 | 258.6799769 | -1.027543342 | 1.74E-12 | 3.25E-11 | EPHX4 |
| ENSG00000167600 | 405.848574 | -1.100860075 | 1.74E-12 | 3.25E-11 | CYP2S1 |
| ENSG00000112599 | 266.0443399 | 1.678949364 | 1.76E-12 | 3.29E-11 | GUCA1B |
| ENSG00000157017 | 59.13594935 | -2.1144217 | 1.85E-12 | 3.45E-11 | GHRL |
| ENSG00000169231 | 443.3650818 | 1.20262426 | 1.97E-12 | 3.67E-11 | THBS3 |
| ENSG00000132718 | 206.4216324 | 1.995155657 | 1.98E-12 | 3.68E-11 | SYT11 |
| ENSG00000122122 | 694.9872322 | -1.329962903 | 2.04E-12 | 3.79E-11 | SASH3 |
| ENSG00000112742 | 387.5037377 | -1.059961399 | 2.08E-12 | 3.85E-11 | TTK |
| ENSG00000183049 | 255.9737628 | -1.037767678 | 2.16E-12 | 3.99E-11 | CAMK1D |
| ENSG00000111846 | 152.0251658 | -1.353031457 | 2.17E-12 | 4.01E-11 | GCNT2 |
| ENSG00000188070 | 241.5598633 | -1.075338071 | 2.46E-12 | 4.53E-11 | ZFTA |
| ENSG00000171307 | 452.2168453 | -1.153921821 | 2.61E-12 | 4.80E-11 | ZDHHC16 |
| ENSG00000139160 | 67.6710921 | 2.470854729 | 2.66E-12 | 4.87E-11 | ETFBKMT |
| ENSG00000166780 | 269.0351579 | -1.006728311 | 2.74E-12 | 5.02E-11 | BMERB1 |
| ENSG00000155011 | 121.1168747 | -1.554058973 | 2.75E-12 | 5.03E-11 | DKK2 |
| ENSG00000090020 | 1139.618158 | 1.028045502 | 2.98E-12 | 5.43E-11 | SLC9A1 |
| ENSG00000141504 | 215.3968636 | 1.227520491 | 3.22E-12 | 5.85E-11 | SAT2 |
| ENSG00000138311 | 42.18009233 | 2.837097188 | 3.32E-12 | 6.03E-11 | ZNF365 |
| ENSG00000171813 | 192.8288057 | 1.092165661 | 3.46E-12 | 6.27E-11 | PWWP2B |
| ENSG00000173207 | 300.9824299 | -1.033796541 | 3.58E-12 | 6.46E-11 | CKS1B |
| ENSG00000169738 | 561.3503358 | 1.120667357 | 4.16E-12 | 7.50E-11 | DCXR |
| ENSG00000169291 | 207.8682531 | 1.656187292 | 4.56E-12 | 8.20E-11 | SHE |
| ENSG00000197647 | 107.0491024 | 1.746859243 | 4.89E-12 | 8.77E-11 | ZNF433 |
| ENSG00000163291 | 538.3586348 | 1.113783586 | 4.92E-12 | 8.80E-11 | PAQR3 |
| ENSG00000165409 | 74.64330956 | -1.993294621 | 4.92E-12 | 8.80E-11 | TSHR |
| ENSG00000172348 | 87.22904115 | -1.72123996 | 5.28E-12 | 9.43E-11 | RCAN2 |
| ENSG00000198551 | 340.8850976 | 1.29538042 | 5.98E-12 | 1.06E-10 | ZNF627 |
| ENSG00000114805 | 33.07171658 | 3.770893143 | 6.01E-12 | 1.07E-10 | PLCH1 |
| ENSG00000185507 | 803.711676 | -1.135108976 | 6.23E-12 | 1.10E-10 | IRF7 |
| ENSG00000149212 | 159.9474458 | -1.377841259 | 6.55E-12 | 1.16E-10 | SESN3 |
| ENSG00000130204 | 1431.375636 | -1.0325918 | 7.85E-12 | 1.38E-10 | TOMM40 |
| ENSG00000099954 | 255.3722152 | 1.210295347 | 8.02E-12 | 1.41E-10 | CECR2 |
| ENSG00000145569 | 428.759461 | -1.029096369 | 8.09E-12 | 1.42E-10 | OTULINL |
| ENSG00000133812 | 770.2103712 | 1.014185021 | 8.22E-12 | 1.44E-10 | SBF2 |
| ENSG00000137558 | 39.04673674 | -2.49313378 | 8.35E-12 | 1.46E-10 | PI15 |
| ENSG00000139631 | 341.8101021 | 1.122473292 | 8.59E-12 | 1.50E-10 | CSAD |
| ENSG00000168496 | 425.2433482 | -1.009181679 | 8.83E-12 | 1.54E-10 | FEN1 |
| ENSG00000114126 | 181.9526336 | -1.089927867 | 9.21E-12 | 1.60E-10 | TFDP2 |
| ENSG00000198417 | 35.63747931 | -2.842721071 | 9.36E-12 | 1.63E-10 | MT1F |
| ENSG00000136492 | 211.008313 | -1.146192418 | 1.00E-11 | 1.73E-10 | BRIP1 |
| ENSG00000185634 | 47.95443729 | -2.421888461 | 1.09E-11 | 1.88E-10 | SHC4 |
| ENSG00000144821 | 29.13783498 | -3.295257886 | 1.12E-11 | 1.92E-10 | MYH15 |
| ENSG00000186409 | 264.0588244 | 1.017323855 | 1.25E-11 | 2.14E-10 | CCDC30 |
| ENSG00000268758 | 410.9755905 | -1.099565624 | 1.36E-11 | 2.32E-10 | ADGRE4P |
| ENSG00000184371 | 2215.388806 | 2.964475928 | 1.38E-11 | 2.35E-10 | CSF1 |
| ENSG00000164087 | 336.070135 | -1.052957918 | 1.41E-11 | 2.40E-10 | POC1A |
| ENSG00000229852 | 163.9558743 | -1.207659168 | 1.41E-11 | 2.41E-10 | KHDC1-AS1 |
| ENSG00000204482 | 389.495261 | -1.205927953 | 1.42E-11 | 2.42E-10 | LST1 |
| ENSG00000117479 | 323.8738415 | 1.170630072 | 1.44E-11 | 2.45E-10 | SLC19A2 |
| ENSG00000168672 | 768.4876784 | 1.048612928 | 1.61E-11 | 2.72E-10 | LRATD2 |
| ENSG00000146670 | 255.334824 | -1.015868903 | 1.75E-11 | 2.96E-10 | CDCA5 |
| ENSG00000096092 | 138.7882173 | -1.331502106 | 1.81E-11 | 3.05E-10 | TMEM14A |
| ENSG00000197646 | 38.77453198 | 3.03030041 | 1.82E-11 | 3.07E-10 | PDCD1LG2 |
| ENSG00000100147 | 907.8310523 | 1.030355579 | 1.86E-11 | 3.14E-10 | CCDC134 |
| ENSG00000143028 | 53.72070894 | -1.970935764 | 2.00E-11 | 3.35E-10 | SYPL2 |
| ENSG00000263961 | 49.18938233 | -8.083431638 | 2.03E-11 | 3.40E-10 | RHEX |
| ENSG00000101447 | 128.4969803 | -1.317591344 | 2.06E-11 | 3.45E-10 | FAM83D |
| ENSG00000133466 | 233.5177814 | 1.069354211 | 2.52E-11 | 4.18E-10 | C1QTNF6 |
| ENSG00000099822 | 77.1223967 | 2.123733148 | 2.61E-11 | 4.33E-10 | HCN2 |
| ENSG00000128039 | 96.60006127 | -1.511847971 | 2.70E-11 | 4.46E-10 | SRD5A3 |
| ENSG00000011638 | 195.2960277 | 1.82314555 | 2.70E-11 | 4.47E-10 | LDAF1 |
| ENSG00000134256 | 36.19202752 | -2.62708364 | 2.75E-11 | 4.54E-10 | CD101 |
| ENSG00000185697 | 78.02112488 | -1.636238611 | 2.81E-11 | 4.62E-10 | MYBL1 |
| ENSG00000139734 | 251.6648333 | -1.070902983 | 2.88E-11 | 4.74E-10 | DIAPH3 |
| ENSG00000144677 | 128.8834098 | -1.477212217 | 2.93E-11 | 4.81E-10 | CTDSPL |
| ENSG00000129993 | 134.991722 | -1.323787114 | 3.39E-11 | 5.55E-10 | CBFA2T3 |
| ENSG00000156687 | 65.7635342 | -1.7212669 | 3.41E-11 | 5.57E-10 | UNC5D |
| ENSG00000167552 | 469.1158226 | -1.036082116 | 3.48E-11 | 5.69E-10 | TUBA1A |
| ENSG00000099785 | 206.8012667 | 1.30925318 | 3.62E-11 | 5.91E-10 | MARCHF2 |
| ENSG00000149929 | 164.5627839 | -1.181931143 | 3.65E-11 | 5.95E-10 | HIRIP3 |
| ENSG00000103226 | 1106.595232 | 1.032078777 | 3.77E-11 | 6.12E-10 | NOMO3 |
| ENSG00000160957 | 677.8243741 | -1.080575957 | 3.83E-11 | 6.19E-10 | RECQL4 |
| ENSG00000138180 | 240.8963943 | -1.069495631 | 3.85E-11 | 6.23E-10 | CEP55 |
| ENSG00000223361 | 66.24882016 | 1.848781098 | 3.88E-11 | 6.27E-10 | FTH1P10 |
| ENSG00000021762 | 262.8309593 | -1.068668254 | 3.93E-11 | 6.35E-10 | OSBPL5 |
| ENSG00000105926 | 278.4797406 | 1.02192981 | 4.06E-11 | 6.56E-10 | PALS2 |
| ENSG00000234664 | 290.3449849 | -1.166254952 | 4.58E-11 | 7.35E-10 | HMGN2P5 |
| ENSG00000167207 | 70.23765994 | -1.905689153 | 4.81E-11 | 7.68E-10 | NOD2 |
| ENSG00000139998 | 94.27597627 | -1.570107146 | 4.97E-11 | 7.94E-10 | RAB15 |
| ENSG00000204291 | 35.21727238 | -2.752808748 | 5.13E-11 | 8.18E-10 | COL15A1 |
| ENSG00000248508 | 173.6223156 | 1.092071497 | 5.17E-11 | 8.25E-10 | SRP14-DT |
| ENSG00000163472 | 175.052262 | 1.230949272 | 5.20E-11 | 8.28E-10 | TMEM79 |
| ENSG00000148356 | 439.8274953 | 1.232391381 | 5.41E-11 | 8.58E-10 | LRSAM1 |
| ENSG00000166317 | 34.75863132 | -3.003191165 | 5.56E-11 | 8.80E-10 | SYNPO2L |
| ENSG00000113749 | 340.6545987 | -1.019511941 | 5.85E-11 | 9.25E-10 | HRH2 |
| ENSG00000064300 | 113.232487 | 4.693470714 | 6.00E-11 | 9.48E-10 | NGFR |
| ENSG00000188985 | 153.6601051 | -1.36619489 | 6.02E-11 | 9.50E-10 | DHFRP1 |
| ENSG00000254726 | 247.7924166 | 1.61223763 | 6.14E-11 | 9.68E-10 | MEX3A |
| ENSG00000248008 | 88.005613 | -1.612822851 | 6.91E-11 | 1.08E-09 | NRAV |
| ENSG00000134901 | 49.91840018 | -2.044665893 | 7.08E-11 | 1.11E-09 | POGLUT2 |
| ENSG00000198719 | 277.2326148 | 1.303200164 | 7.23E-11 | 1.13E-09 | DLL1 |
| ENSG00000120051 | 155.8303847 | 1.304417716 | 7.58E-11 | 1.18E-09 | CFAP58 |
| ENSG00000149218 | 161.7523689 | -1.236420711 | 7.82E-11 | 1.21E-09 | ENDOD1 |
| ENSG00000170153 | 140.7370371 | 4.308735032 | 8.57E-11 | 1.32E-09 | RNF150 |
| ENSG00000155893 | 79.95385331 | -1.553575175 | 8.87E-11 | 1.37E-09 | PXYLP1 |
| ENSG00000091409 | 398.0027938 | -1.128817631 | 8.90E-11 | 1.37E-09 | ITGA6 |
| ENSG00000215190 | 63.75246315 | -1.70573511 | 9.11E-11 | 1.40E-09 | LINC00680 |
| ENSG00000167604 | 393.9739913 | 1.163202703 | 9.17E-11 | 1.41E-09 | NFKBID |
| ENSG00000149564 | 188.5243557 | -1.021717323 | 9.72E-11 | 1.49E-09 | ESAM |
| ENSG00000104055 | 27.9869234 | -5.770260569 | 9.95E-11 | 1.52E-09 | TGM5 |
| ENSG00000107719 | 241.4677916 | -1.014928548 | 1.03E-10 | 1.57E-09 | PALD1 |
| ENSG00000174206 | 189.7578689 | 1.055478044 | 1.07E-10 | 1.62E-09 | KICS2 |
| ENSG00000172738 | 37.06704463 | -2.360603499 | 1.10E-10 | 1.67E-09 | TMEM217 |
| ENSG00000162694 | 193.9620909 | -1.139146606 | 1.13E-10 | 1.72E-09 | EXTL2 |
| ENSG00000115109 | 207.1658501 | 1.254168901 | 1.26E-10 | 1.90E-09 | EPB41L5 |
| ENSG00000163032 | 150.857218 | -1.354317301 | 1.29E-10 | 1.94E-09 | VSNL1 |
| ENSG00000142046 | 110.8038832 | 1.308694281 | 1.38E-10 | 2.07E-09 | TMEM91 |
| ENSG00000140534 | 212.7932246 | -1.026063934 | 1.40E-10 | 2.10E-09 | TICRR |
| ENSG00000164674 | 194.6913443 | 1.106460075 | 1.43E-10 | 2.13E-09 | SYTL3 |
| ENSG00000134824 | 609.5896703 | -1.245952749 | 1.44E-10 | 2.15E-09 | FADS2 |
| ENSG00000132965 | 2118.683665 | -2.281523485 | 1.50E-10 | 2.23E-09 | ALOX5AP |
| ENSG00000115163 | 135.5147733 | -1.374118844 | 1.51E-10 | 2.24E-09 | CENPA |
| ENSG00000140450 | 469.6804627 | 1.342986913 | 1.51E-10 | 2.24E-09 | ARRDC4 |
| ENSG00000163818 | 146.5291233 | -1.15538407 | 1.57E-10 | 2.33E-09 | LZTFL1 |
| ENSG00000197182 | 107.2252899 | 1.700932247 | 1.72E-10 | 2.55E-09 | MIRLET7BHG |
| ENSG00000186417 | 5492.042351 | -1.802001164 | 1.76E-10 | 2.60E-09 | GLDN |
| ENSG00000270344 | 123.0797902 | -1.352870089 | 1.76E-10 | 2.60E-09 | POC1B-AS1 |
| ENSG00000226318 | 43.21400196 | 2.540195644 | 1.79E-10 | 2.65E-09 | RPS3AP38 |
| ENSG00000181754 | 124.17932 | -1.208137244 | 2.11E-10 | 3.10E-09 | AMIGO1 |
| ENSG00000100290 | 81.95542858 | -1.981095384 | 2.12E-10 | 3.12E-09 | BIK |
| ENSG00000112137 | 129.1641098 | -1.43586332 | 2.19E-10 | 3.21E-09 | PHACTR1 |
| ENSG00000115687 | 205.9858862 | -1.046958849 | 2.22E-10 | 3.26E-09 | PASK |
| ENSG00000164867 | 41.99056986 | -2.310855251 | 2.34E-10 | 3.43E-09 | NOS3 |
| ENSG00000169715 | 21.83527968 | -4.132635794 | 2.41E-10 | 3.52E-09 | MT1E |
| ENSG00000110844 | 68.1420313 | -1.689613478 | 2.47E-10 | 3.60E-09 | PRPF40B |
| ENSG00000173275 | 134.6854202 | 1.43643767 | 2.51E-10 | 3.65E-09 | ZNF449 |
| ENSG00000065328 | 235.4405298 | -1.145917625 | 2.52E-10 | 3.66E-09 | MCM10 |
| ENSG00000170325 | 653.3230529 | 1.047049278 | 2.53E-10 | 3.68E-09 | PRDM10 |
| ENSG00000185112 | 163.3186001 | -1.698398047 | 2.54E-10 | 3.69E-09 | FAM43A |
| ENSG00000163453 | 151.3324996 | -1.109837343 | 2.66E-10 | 3.85E-09 | IGFBP7 |
| ENSG00000277494 | 72.28897458 | 1.561596788 | 2.74E-10 | 3.96E-09 | GPIHBP1 |
| ENSG00000006740 | 167.4270534 | -1.411828588 | 2.82E-10 | 4.06E-09 | ARHGAP44 |
| ENSG00000092094 | 533.4755994 | -1.104740638 | 2.91E-10 | 4.19E-09 | OSGEP |
| ENSG00000152104 | 470.142756 | -1.069972054 | 2.96E-10 | 4.26E-09 | PTPN14 |
| ENSG00000077984 | 42.03299133 | 2.727793584 | 3.09E-10 | 4.43E-09 | CST7 |
| ENSG00000170396 | 522.4553246 | -1.305868956 | 3.16E-10 | 4.52E-09 | ZNF804A |
| ENSG00000113389 | 33.72719675 | 4.314364943 | 3.16E-10 | 4.53E-09 | NPR3 |
| ENSG00000134690 | 175.5830561 | -1.202343047 | 3.27E-10 | 4.68E-09 | CDCA8 |
| ENSG00000172794 | 36.58448305 | -2.385805623 | 3.61E-10 | 5.14E-09 | RAB37 |
| ENSG00000213071 | 43.22173104 | -2.434120197 | 3.66E-10 | 5.21E-09 | LPAL2 |
| ENSG00000125355 | 301.7116041 | 1.441881865 | 3.74E-10 | 5.31E-09 | TMEM255A |
| ENSG00000124549 | 149.9726189 | 1.092777198 | 3.77E-10 | 5.36E-09 | BTN2A3P |
| ENSG00000167397 | 197.2839999 | 1.063675225 | 3.79E-10 | 5.38E-09 | VKORC1 |
| ENSG00000271643 | 78.51671622 | -1.678390405 | 3.80E-10 | 5.39E-09 | PDCD6IP-DT |
| ENSG00000184319 | 468.0646742 | 1.061417215 | 3.85E-10 | 5.45E-09 | RPL23AP82 |
| ENSG00000140280 | 178.7105219 | -1.179105424 | 3.90E-10 | 5.52E-09 | LYSMD2 |
| ENSG00000163430 | 35.9313127 | -2.454062184 | 4.04E-10 | 5.72E-09 | FSTL1 |
| ENSG00000005882 | 133.1805367 | -1.276957343 | 4.09E-10 | 5.77E-09 | PDK2 |
| ENSG00000180626 | 99.73361476 | -1.314933919 | 4.09E-10 | 5.77E-09 | ZNF594 |
| ENSG00000169860 | 181.1124811 | 1.363251938 | 4.11E-10 | 5.80E-09 | P2RY1 |
| ENSG00000175573 | 163.2113416 | 1.212417137 | 4.31E-10 | 6.05E-09 | C11orf68 |
| ENSG00000215375 | 236.6783673 | 1.059580302 | 4.33E-10 | 6.08E-09 | MYL5 |
| ENSG00000146950 | 28.82840594 | -2.919492736 | 4.35E-10 | 6.10E-09 | SHROOM2 |
| ENSG00000120837 | 204.1119638 | -1.002457122 | 4.53E-10 | 6.34E-09 | NFYB |
| ENSG00000164626 | 117.8541986 | -1.649963376 | 4.68E-10 | 6.55E-09 | KCNK5 |
| ENSG00000165795 | 233.8647548 | -1.105673946 | 4.71E-10 | 6.59E-09 | NDRG2 |
| ENSG00000080986 | 255.3323464 | -1.121561927 | 4.93E-10 | 6.88E-09 | NDC80 |
| ENSG00000198125 | 22.04520918 | -4.989727613 | 5.02E-10 | 6.99E-09 | MB |
| ENSG00000187775 | 208.0887152 | 1.06466318 | 5.13E-10 | 7.14E-09 | DNAH17 |
| ENSG00000175182 | 105.9242011 | 1.513825301 | 5.36E-10 | 7.45E-09 | FAM131A |
| ENSG00000164125 | 83.95357334 | 1.858041677 | 5.58E-10 | 7.74E-09 | GASK1B |
| ENSG00000168803 | 157.0148414 | -1.227135187 | 5.63E-10 | 7.81E-09 | ADAL |
| ENSG00000137460 | 159.1062265 | 1.154273874 | 5.78E-10 | 8.01E-09 | FHDC1 |
| ENSG00000267060 | 149.6249453 | -1.245495971 | 6.59E-10 | 9.07E-09 | PTGES3L |
| ENSG00000186162 | 267.986283 | 1.023062673 | 7.08E-10 | 9.70E-09 | CIDECP1 |
| ENSG00000122025 | 952.5285808 | -2.35971818 | 7.20E-10 | 9.86E-09 | FLT3 |
| ENSG00000120659 | 54.45942017 | -1.888380368 | 7.50E-10 | 1.02E-08 | TNFSF11 |
| ENSG00000154864 | 51.04950368 | -2.216947603 | 7.76E-10 | 1.06E-08 | PIEZO2 |
| ENSG00000225101 | 23.70138806 | -3.184582766 | 7.95E-10 | 1.08E-08 | OR52K3P |
| ENSG00000145386 | 394.9389595 | -1.134384375 | 8.06E-10 | 1.09E-08 | CCNA2 |
| ENSG00000243055 | 51.74115067 | 2.173945292 | 8.37E-10 | 1.13E-08 | GK-AS1 |
| ENSG00000121210 | 387.8220557 | -1.157609311 | 8.93E-10 | 1.20E-08 | TMEM131L |
| ENSG00000097096 | 46.55010463 | 2.470417757 | 9.08E-10 | 1.22E-08 | SYDE2 |
| ENSG00000125148 | 37.31381304 | -2.793575149 | 9.58E-10 | 1.29E-08 | MT2A |
| ENSG00000174501 | 188.3434796 | 1.061162339 | 1.06E-09 | 1.42E-08 | ANKRD36C |
| ENSG00000101849 | 55.75840396 | -1.80085842 | 1.15E-09 | 1.53E-08 | TBL1X |
| ENSG00000237672 | 71.5796824 | 1.998386851 | 1.22E-09 | 1.61E-08 | KRR1P1 |
| ENSG00000145022 | 220.8134794 | -1.150583066 | 1.36E-09 | 1.79E-08 | TCTA |
| ENSG00000168502 | 157.5401323 | 1.077798635 | 1.36E-09 | 1.79E-08 | MTCL1 |
| ENSG00000196220 | 72.48062237 | 1.484997563 | 1.40E-09 | 1.84E-08 | SRGAP3 |
| ENSG00000157601 | 1050.526627 | -1.932023658 | 1.44E-09 | 1.88E-08 | MX1 |
| ENSG00000086570 | 183.337006 | 1.822760609 | 1.54E-09 | 2.00E-08 | FAT2 |
| ENSG00000198901 | 153.4574225 | -1.121878958 | 1.56E-09 | 2.03E-08 | PRC1 |
| ENSG00000237943 | 54.4786176 | -1.706828205 | 1.60E-09 | 2.07E-08 | PRKCQ-AS1 |
| ENSG00000196569 | 120.4710668 | -1.484267816 | 1.64E-09 | 2.12E-08 | LAMA2 |
| ENSG00000088367 | 265.6158781 | 1.08545642 | 1.65E-09 | 2.13E-08 | EPB41L1 |
| ENSG00000149599 | 40.22748101 | 2.559813289 | 1.68E-09 | 2.17E-08 | DUSP15 |
| ENSG00000150687 | 82.24107022 | 1.908071978 | 1.84E-09 | 2.37E-08 | PRSS23 |
| ENSG00000232082 | 84.95518508 | 1.405804207 | 2.13E-09 | 2.73E-08 | RPS6KA2-IT1 |
| ENSG00000148690 | 288.6004241 | 1.024124031 | 2.19E-09 | 2.80E-08 | FRA10AC1 |
| ENSG00000151612 | 170.9584035 | 1.015110717 | 2.27E-09 | 2.90E-08 | ZNF827 |
| ENSG00000181634 | 803.5896359 | -2.27936768 | 2.35E-09 | 3.00E-08 | TNFSF15 |
| ENSG00000132423 | 129.0082929 | -1.263662632 | 2.36E-09 | 3.01E-08 | COQ3 |
| ENSG00000151012 | 2280.676099 | 1.85552532 | 2.38E-09 | 3.03E-08 | SLC7A11 |
| ENSG00000146674 | 814.6221341 | -1.111583741 | 2.56E-09 | 3.25E-08 | IGFBP3 |
| ENSG00000115137 | 237.6352636 | 1.299532461 | 2.65E-09 | 3.35E-08 | DNAJC27 |
| ENSG00000172572 | 28.69683908 | -6.399390053 | 2.73E-09 | 3.45E-08 | PDE3A |
| ENSG00000137135 | 150.1595375 | -1.266187487 | 2.77E-09 | 3.50E-08 | ARHGEF39 |
| ENSG00000171224 | 44.62667123 | 1.909054679 | 2.86E-09 | 3.61E-08 | FAM241B |
| ENSG00000237190 | 182.3246756 | -1.041588693 | 3.11E-09 | 3.90E-08 | CDKN2AIPNL |
| ENSG00000005448 | 72.19308147 | -1.57226416 | 3.21E-09 | 4.02E-08 | WDR54 |
| ENSG00000166803 | 150.0736644 | -1.285679111 | 3.21E-09 | 4.02E-08 | PCLAF |
| ENSG00000168386 | 123.5384704 | -1.369201172 | 3.24E-09 | 4.05E-08 | FILIP1L |
| ENSG00000154027 | 26.46763188 | -2.871344763 | 3.26E-09 | 4.07E-08 | AK5 |
| ENSG00000144395 | 73.02226598 | -1.562461805 | 3.60E-09 | 4.48E-08 | CCDC150 |
| ENSG00000128165 | 165.1439171 | 1.549117075 | 3.78E-09 | 4.70E-08 | ADM2 |
| ENSG00000184828 | 68.04228436 | -1.63201158 | 4.44E-09 | 5.48E-08 | ZBTB7C |
| ENSG00000187980 | 73.85160374 | -1.770299905 | 4.48E-09 | 5.52E-08 | PLA2G2C |
| ENSG00000078900 | 47.27081011 | -1.880051275 | 4.51E-09 | 5.55E-08 | TP73 |
| ENSG00000117155 | 123.4374085 | -1.105421182 | 4.76E-09 | 5.85E-08 | SSX2IP |
| ENSG00000182667 | 39.73866971 | -2.166588586 | 5.51E-09 | 6.73E-08 | NTM |
| ENSG00000184381 | 135.6359986 | 1.236403371 | 5.51E-09 | 6.73E-08 | PLA2G6 |
| ENSG00000128641 | 390.3620889 | 1.076237395 | 5.51E-09 | 6.73E-08 | MYO1B |
| ENSG00000147894 | 246.8123601 | 1.311085694 | 5.56E-09 | 6.78E-08 | C9orf72 |
| ENSG00000140030 | 246.8572207 | 1.349878826 | 5.71E-09 | 6.94E-08 | GPR65 |
| ENSG00000238005 | 54.63954714 | -1.791561501 | 5.72E-09 | 6.95E-08 | LNCATV |
| ENSG00000008311 | 186.146067 | 1.125913913 | 5.77E-09 | 7.01E-08 | AASS |
| ENSG00000249937 | 52.19895766 | -1.728247901 | 5.98E-09 | 7.24E-08 | LINC02223 |
| ENSG00000213160 | 88.74203138 | -1.41046084 | 6.20E-09 | 7.50E-08 | KLHL23 |
| ENSG00000054277 | 98.19778483 | 1.57420924 | 6.70E-09 | 8.08E-08 | OPN3 |
| ENSG00000111186 | 29.24683343 | -2.649822656 | 6.71E-09 | 8.08E-08 | WNT5B |
| ENSG00000121897 | 158.3174622 | 1.16220706 | 6.90E-09 | 8.29E-08 | LIAS |
| ENSG00000130054 | 85.62157423 | 1.771059724 | 6.92E-09 | 8.31E-08 | NALF2 |
| ENSG00000188906 | 58.77853755 | -1.770655287 | 6.96E-09 | 8.36E-08 | LRRK2 |
| ENSG00000151491 | 689.8454314 | 1.003726474 | 6.96E-09 | 8.36E-08 | EPS8 |
| ENSG00000041988 | 102.1716224 | 1.205105535 | 7.06E-09 | 8.47E-08 | THAP3 |
| ENSG00000088986 | 2147.304735 | -1.682660388 | 7.29E-09 | 8.73E-08 | DYNLL1 |
| ENSG00000168404 | 170.4564488 | -1.199786926 | 7.49E-09 | 8.94E-08 | MLKL |
| ENSG00000177483 | 43.90303213 | 2.449421897 | 7.64E-09 | 9.12E-08 | RBM44 |
| ENSG00000113966 | 76.26192434 | -1.604279458 | 8.16E-09 | 9.70E-08 | ARL6 |
| ENSG00000196646 | 170.5010604 | 1.13834404 | 8.25E-09 | 9.79E-08 | ZNF136 |
| ENSG00000241721 | 64.6580548 | 1.490843693 | 8.28E-09 | 9.83E-08 | SUMO1P1 |
| ENSG00000168952 | 32.70882803 | -2.189167046 | 8.36E-09 | 9.91E-08 | STXBP6 |
| ENSG00000131067 | 201.2458748 | -1.628453654 | 8.53E-09 | 1.01E-07 | GGT7 |
| ENSG00000101307 | 117.0346653 | -1.589352107 | 8.75E-09 | 1.03E-07 | SIRPB1 |
| ENSG00000240024 | 94.50243162 | -1.306260909 | 9.30E-09 | 1.10E-07 | LINC00888 |
| ENSG00000226453 | 64.45163153 | 1.499908007 | 9.30E-09 | 1.10E-07 | LINC02542 |
| ENSG00000002587 | 22.90362631 | -2.810148071 | 9.40E-09 | 1.11E-07 | HS3ST1 |
| ENSG00000256746 | 22.41693117 | 3.041951067 | 9.44E-09 | 1.11E-07 | MADD-AS1 |
| ENSG00000099985 | 232.7214885 | -1.460612108 | 9.70E-09 | 1.14E-07 | OSM |
| ENSG00000156804 | 1413.055042 | 1.752535421 | 1.00E-08 | 1.17E-07 | FBXO32 |
| ENSG00000115008 | 49.50563788 | -1.849855617 | 1.03E-08 | 1.20E-07 | IL1A |
| ENSG00000138459 | 184.6368296 | -1.139577791 | 1.11E-08 | 1.29E-07 | SLC35A5 |
| ENSG00000197859 | 36.89939787 | -2.170757275 | 1.12E-08 | 1.30E-07 | ADAMTSL2 |
| ENSG00000086544 | 188.4065256 | 1.16008553 | 1.13E-08 | 1.31E-07 | ITPKC |
| ENSG00000105948 | 19.01051462 | -3.363432747 | 1.15E-08 | 1.33E-07 | TTC26 |
| ENSG00000154930 | 300.0358698 | 1.009081433 | 1.15E-08 | 1.34E-07 | ACSS1 |
| ENSG00000050767 | 511.0716252 | 1.030777648 | 1.16E-08 | 1.34E-07 | COL23A1 |
| ENSG00000082512 | 86.66627709 | 1.349908333 | 1.22E-08 | 1.41E-07 | TRAF5 |
| ENSG00000177791 | 202.2319639 | -1.206229029 | 1.24E-08 | 1.43E-07 | MYOZ1 |
| ENSG00000246705 | 156.762196 | 1.237770131 | 1.28E-08 | 1.47E-07 | H2AJ |
| ENSG00000021826 | 141.5435166 | -1.081061701 | 1.32E-08 | 1.52E-07 | CPS1 |
| ENSG00000101868 | 448.0382838 | -1.050011015 | 1.34E-08 | 1.54E-07 | POLA1 |
| ENSG00000244040 | 147.3175552 | -1.064755662 | 1.35E-08 | 1.55E-07 | IL12A-AS1 |
| ENSG00000172197 | 197.7928013 | -1.085918702 | 1.39E-08 | 1.59E-07 | MBOAT1 |
| ENSG00000106804 | 167.1687903 | -1.033307864 | 1.40E-08 | 1.60E-07 | C5 |
| ENSG00000118276 | 95.3464425 | -1.296321429 | 1.41E-08 | 1.61E-07 | B4GALT6 |
| ENSG00000117519 | 29.86428744 | -2.360679395 | 1.51E-08 | 1.72E-07 | CNN3 |
| ENSG00000035664 | 106.8478421 | -1.343309545 | 1.58E-08 | 1.79E-07 | DAPK2 |
| ENSG00000142173 | 548.9954226 | 1.051816383 | 1.59E-08 | 1.80E-07 | COL6A2 |
| ENSG00000181016 | 47.57169365 | 1.87570372 | 1.61E-08 | 1.81E-07 | LSMEM1 |
| ENSG00000177272 | 162.8333707 | -1.014900046 | 1.61E-08 | 1.82E-07 | KCNA3 |
| ENSG00000164953 | 32.90643296 | -2.151370485 | 1.66E-08 | 1.88E-07 | TMEM67 |
| ENSG00000130368 | 37.95977451 | 1.924066919 | 1.74E-08 | 1.96E-07 | MAS1 |
| ENSG00000204611 | 107.1486662 | 1.206637346 | 1.78E-08 | 2.00E-07 | ZNF616 |
| ENSG00000187240 | 126.5217145 | -1.260251557 | 1.85E-08 | 2.07E-07 | DYNC2H1 |
| ENSG00000250091 | 114.3650509 | -1.185248433 | 1.85E-08 | 2.07E-07 | DNAH10OS |
| ENSG00000247774 | 75.04081409 | -1.411354924 | 1.88E-08 | 2.10E-07 | PCED1B-AS1 |
| ENSG00000102359 | 108.7046058 | 1.312036071 | 1.93E-08 | 2.16E-07 | SRPX2 |
| ENSG00000119508 | 1140.675549 | 2.759776361 | 2.01E-08 | 2.24E-07 | NR4A3 |
| ENSG00000198590 | 125.5140153 | 1.675696904 | 2.01E-08 | 2.24E-07 | APRG1 |
| ENSG00000111665 | 89.99365993 | -1.281868593 | 2.08E-08 | 2.32E-07 | CDCA3 |
| ENSG00000122952 | 592.077873 | -1.9645854 | 2.15E-08 | 2.39E-07 | ZWINT |
| ENSG00000236345 | 72.60892362 | -1.44558246 | 2.15E-08 | 2.39E-07 | SCAT8 |
| ENSG00000100276 | 24.3598013 | -2.646285054 | 2.21E-08 | 2.45E-07 | RASL10A |
| ENSG00000139132 | 67.86223325 | 1.552142348 | 2.25E-08 | 2.49E-07 | FGD4 |
| ENSG00000152784 | 80.93409172 | -1.769208703 | 2.26E-08 | 2.49E-07 | PRDM8 |
| ENSG00000102384 | 122.0705165 | -1.090663351 | 2.42E-08 | 2.66E-07 | CENPI |
| ENSG00000150907 | 273.7757943 | 1.218375296 | 2.47E-08 | 2.71E-07 | FOXO1 |
| ENSG00000197496 | 83.43432918 | -1.333918844 | 2.55E-08 | 2.80E-07 | SLC2A10 |
| ENSG00000264343 | 177.934698 | 1.352828688 | 2.56E-08 | 2.80E-07 | NOTCH2NLA |
| ENSG00000120458 | 163.2294993 | -1.048310808 | 2.66E-08 | 2.91E-07 | MSANTD2 |
| ENSG00000154451 | 56.04911753 | -2.315810159 | 2.78E-08 | 3.04E-07 | GBP5 |
| ENSG00000161999 | 192.3509153 | -1.134652397 | 2.88E-08 | 3.14E-07 | JMJD8 |
| ENSG00000184470 | 380.8467151 | -1.005975735 | 2.97E-08 | 3.23E-07 | TXNRD2 |
| ENSG00000166548 | 287.8557476 | -1.134324841 | 3.00E-08 | 3.27E-07 | TK2 |
| ENSG00000100385 | 27.89404453 | -2.789968847 | 3.19E-08 | 3.46E-07 | IL2RB |
| ENSG00000204764 | 70.41896135 | -1.434568445 | 3.22E-08 | 3.48E-07 | RANBP17 |
| ENSG00000087085 | 21.52671194 | 2.774228105 | 3.30E-08 | 3.57E-07 | ACHE |
| ENSG00000100379 | 127.2224114 | -1.173802815 | 3.40E-08 | 3.67E-07 | KCTD17 |
| ENSG00000102547 | 1448.239905 | 1.624415341 | 3.47E-08 | 3.75E-07 | CAB39L |
| ENSG00000236915 | 97.91548318 | -1.183016667 | 3.48E-08 | 3.76E-07 | CLCA4-AS1 |
| ENSG00000123570 | 41.0663258 | -1.816824776 | 3.55E-08 | 3.82E-07 | RAB9B |
| ENSG00000118113 | 39.09513645 | -2.053506365 | 3.57E-08 | 3.84E-07 | MMP8 |
| ENSG00000112139 | 518.7654771 | -2.401842026 | 3.73E-08 | 4.00E-07 | MDGA1 |
| ENSG00000120063 | 7995.116147 | 1.767843618 | 3.78E-08 | 4.05E-07 | GNA13 |
| ENSG00000128872 | 17.22341127 | -3.763080681 | 3.87E-08 | 4.14E-07 | TMOD2 |
| ENSG00000113494 | 98.19843899 | -1.328161789 | 3.92E-08 | 4.19E-07 | PRLR |
| ENSG00000175106 | 98.48379439 | 1.175332255 | 4.18E-08 | 4.45E-07 | TVP23C |
| ENSG00000117090 | 26.96088324 | -2.505935463 | 4.36E-08 | 4.63E-07 | SLAMF1 |
| ENSG00000170558 | 38.4833267 | -2.154080309 | 4.47E-08 | 4.74E-07 | CDH2 |
| ENSG00000004777 | 47.4611229 | -1.801279893 | 4.56E-08 | 4.83E-07 | ARHGAP33 |
| ENSG00000175602 | 216.9457274 | 1.379185073 | 4.56E-08 | 4.83E-07 | CCDC85B |
| ENSG00000122970 | 109.8905351 | -1.088216635 | 4.59E-08 | 4.86E-07 | IFT81 |
| ENSG00000115648 | 31.74406383 | -2.576233754 | 5.30E-08 | 5.54E-07 | MLPH |
| ENSG00000067141 | 133.6837813 | 1.003997388 | 5.34E-08 | 5.58E-07 | NEO1 |
| ENSG00000129810 | 131.7245051 | -1.150368487 | 5.35E-08 | 5.59E-07 | SGO1 |
| ENSG00000176046 | 66.28529745 | 1.591824252 | 5.39E-08 | 5.63E-07 | NUPR1 |
| ENSG00000127920 | 49.7499395 | -1.609660047 | 5.71E-08 | 5.93E-07 | GNG11 |
| ENSG00000180340 | 147.60035 | -1.0751431 | 5.88E-08 | 6.08E-07 | FZD2 |
| ENSG00000165912 | 97.6096952 | 1.16815759 | 5.91E-08 | 6.12E-07 | PACSIN3 |
| ENSG00000182782 | 24.46757734 | 3.417152521 | 5.93E-08 | 6.14E-07 | HCAR2 |
| ENSG00000140043 | 112.614978 | 1.220934883 | 6.22E-08 | 6.42E-07 | PTGR2 |
| ENSG00000143333 | 2020.523722 | -1.889530571 | 6.46E-08 | 6.66E-07 | RGS16 |
| ENSG00000198342 | 38.51461995 | 2.100826207 | 6.51E-08 | 6.71E-07 | ZNF442 |
| ENSG00000074219 | 53.47407996 | -1.576596681 | 6.52E-08 | 6.71E-07 | TEAD2 |
| ENSG00000181027 | 91.28876455 | -1.319368419 | 6.58E-08 | 6.77E-07 | FKRP |
| ENSG00000178700 | 161.7546738 | 1.114050622 | 6.60E-08 | 6.78E-07 | DHFR2 |
| ENSG00000169248 | 16.56484994 | -3.951020449 | 7.51E-08 | 7.66E-07 | CXCL11 |
| ENSG00000144043 | 1573.054267 | -1.682111133 | 7.59E-08 | 7.73E-07 | TEX261 |
| ENSG00000141505 | 55.13438618 | -1.761901001 | 7.79E-08 | 7.92E-07 | ASGR1 |
| ENSG00000131979 | 64.36075276 | -1.441561363 | 8.30E-08 | 8.40E-07 | GCH1 |
| ENSG00000234232 | 450.9762226 | 1.16329924 | 8.46E-08 | 8.55E-07 | PDE4DIPP7 |
| ENSG00000169239 | 149.5471303 | 1.330213931 | 8.53E-08 | 8.62E-07 | CA5B |
| ENSG00000070444 | 447.6461999 | 1.051820556 | 9.09E-08 | 9.15E-07 | MNT |
| ENSG00000234076 | 36.47044458 | -1.932938127 | 9.25E-08 | 9.30E-07 | TPRG1-AS1 |
| ENSG00000106351 | 72.0476843 | -1.485180967 | 9.39E-08 | 9.43E-07 | AGFG2 |
| ENSG00000185551 | 76.19831544 | -1.376760636 | 9.43E-08 | 9.46E-07 | NR2F2 |
| ENSG00000178409 | 157.173435 | 1.124252728 | 9.51E-08 | 9.55E-07 | BEND3 |
| ENSG00000248671 | 44.64814436 | 1.641976433 | 9.62E-08 | 9.65E-07 | ALG1L9P |
| ENSG00000240522 | 34.45342842 | 1.903656198 | 9.63E-08 | 9.65E-07 | RPL7AP10 |
| ENSG00000131153 | 93.5094623 | -1.562521923 | 1.02E-07 | 1.02E-06 | GINS2 |
| ENSG00000010319 | 53.4004744 | -1.699064494 | 1.03E-07 | 1.03E-06 | SEMA3G |
| ENSG00000125945 | 139.4969436 | 1.321300552 | 1.03E-07 | 1.03E-06 | ZNF436 |
| ENSG00000196466 | 85.30001855 | 1.212972077 | 1.07E-07 | 1.07E-06 | ZNF799 |
| ENSG00000143147 | 32.72832882 | -2.14028202 | 1.08E-07 | 1.07E-06 | GPR161 |
| ENSG00000286122 | 19.83859632 | -2.896894777 | 1.08E-07 | 1.08E-06 | LINC02964 |
| ENSG00000158402 | 50.82440347 | -1.570796599 | 1.11E-07 | 1.10E-06 | CDC25C |
| ENSG00000273372 | 21.46231584 | -2.699827186 | 1.17E-07 | 1.16E-06 | SFTPD-AS1 |
| ENSG00000109881 | 42.92397169 | -1.831665465 | 1.17E-07 | 1.16E-06 | CCDC34 |
| ENSG00000133863 | 85.20899105 | -1.316252943 | 1.22E-07 | 1.20E-06 | TEX15 |
| ENSG00000147180 | 121.8411883 | -1.18070192 | 1.23E-07 | 1.21E-06 | ZNF711 |
| ENSG00000286019 | 102.7327408 | 1.133711578 | 1.28E-07 | 1.25E-06 | NOTCH2NLB |
| ENSG00000072571 | 336.6593761 | -1.187232751 | 1.32E-07 | 1.30E-06 | HMMR |
| ENSG00000237499 | 108.9657268 | -1.233392497 | 1.35E-07 | 1.32E-06 | WAKMAR2 |
| ENSG00000059915 | 56.62366571 | 1.444129538 | 1.38E-07 | 1.34E-06 | PSD |
| ENSG00000196391 | 57.33094463 | 1.450077393 | 1.39E-07 | 1.35E-06 | ZNF774 |
| ENSG00000145850 | 93.27418199 | 1.68856725 | 1.39E-07 | 1.36E-06 | TIMD4 |
| ENSG00000137673 | 380.585143 | 2.210224194 | 1.39E-07 | 1.36E-06 | MMP7 |
| ENSG00000162374 | 15.5595412 | -3.403221728 | 1.40E-07 | 1.36E-06 | ELAVL4 |
| ENSG00000146540 | 402.4439046 | 1.09382823 | 1.42E-07 | 1.39E-06 | C7orf50 |
| ENSG00000163803 | 125.558974 | -1.006647585 | 1.51E-07 | 1.46E-06 | PLB1 |
| ENSG00000170364 | 149.3125436 | -1.012058695 | 1.53E-07 | 1.48E-06 | SETMAR |
| ENSG00000139055 | 75.9428493 | -1.50494331 | 1.54E-07 | 1.49E-06 | ERP27 |
| ENSG00000117650 | 118.3212418 | -1.189497209 | 1.57E-07 | 1.52E-06 | NEK2 |
| ENSG00000146054 | 36.70136836 | 2.227674875 | 1.58E-07 | 1.53E-06 | TRIM7 |
| ENSG00000267272 | 41.25964273 | -2.650950873 | 1.58E-07 | 1.53E-06 | LINC01140 |
| ENSG00000140451 | 51.96556839 | -1.764425532 | 1.59E-07 | 1.54E-06 | PIF1 |
| ENSG00000073605 | 175.5893159 | 1.030264627 | 1.64E-07 | 1.58E-06 | GSDMB |
| ENSG00000105516 | 119.3577663 | 1.707253136 | 1.70E-07 | 1.63E-06 | DBP |
| ENSG00000112984 | 166.5376011 | -1.080993384 | 1.76E-07 | 1.68E-06 | KIF20A |
| ENSG00000244556 | 25.31090712 | 2.938175187 | 1.78E-07 | 1.70E-06 | ODCP |
| ENSG00000117242 | 82.26912149 | -1.218084168 | 1.78E-07 | 1.71E-06 | PINK1-AS |
| ENSG00000167074 | 256.3855855 | 1.375469039 | 1.81E-07 | 1.73E-06 | TEF |
| ENSG00000099377 | 172.7124982 | 1.059208811 | 1.87E-07 | 1.79E-06 | HSD3B7 |
| ENSG00000235760 | 32.38073386 | -1.981849223 | 2.05E-07 | 1.94E-06 | MSH2-OT1 |
| ENSG00000243650 | 52.12602266 | -1.520495898 | 2.11E-07 | 2.00E-06 | RN7SL834P |
| ENSG00000111452 | 88.42610609 | -1.428104397 | 2.13E-07 | 2.02E-06 | ADGRD1 |
| ENSG00000000460 | 81.81537042 | -1.192439069 | 2.15E-07 | 2.04E-06 | C1orf112 |
| ENSG00000092421 | 60.88397896 | -1.510674746 | 2.19E-07 | 2.07E-06 | SEMA6A |
| ENSG00000104147 | 38.5113198 | -1.987776289 | 2.31E-07 | 2.18E-06 | OIP5 |
| ENSG00000253320 | 184.6493729 | -1.25336808 | 2.32E-07 | 2.19E-06 | MAILR |
| ENSG00000159450 | 67.41839541 | -1.417122288 | 2.41E-07 | 2.26E-06 | TCHH |
| ENSG00000160298 | 103.8799102 | -1.298309601 | 2.50E-07 | 2.35E-06 | C21orf58 |
| ENSG00000130940 | 88.53244683 | 1.2484387 | 2.65E-07 | 2.48E-06 | CASZ1 |
| ENSG00000213949 | 72.47847705 | -1.371772277 | 2.70E-07 | 2.52E-06 | ITGA1 |
| ENSG00000139597 | 323.6807966 | 1.135428516 | 2.79E-07 | 2.60E-06 | N4BP2L1 |
| ENSG00000166348 | 145.5062294 | -1.18906149 | 2.93E-07 | 2.72E-06 | USP54 |
| ENSG00000196664 | 14.0973616 | -4.321709787 | 2.97E-07 | 2.76E-06 | TLR7 |
| ENSG00000159167 | 22.13864437 | -2.481701817 | 3.00E-07 | 2.78E-06 | STC1 |
| ENSG00000139263 | 18.85331777 | 2.7623495 | 3.04E-07 | 2.81E-06 | LRIG3 |
| ENSG00000277561 | 161.1670579 | 1.061032157 | 3.04E-07 | 2.81E-06 | GOLGA8IP |
| ENSG00000135426 | 27.33304032 | -2.145919292 | 3.11E-07 | 2.87E-06 | TESPA1 |
| ENSG00000151006 | 121.5495797 | 1.333243541 | 3.12E-07 | 2.88E-06 | PRSS53 |
| ENSG00000180479 | 51.9314459 | 1.669344942 | 3.12E-07 | 2.88E-06 | ZNF571 |
| ENSG00000174576 | 14.10219071 | -3.977325212 | 3.17E-07 | 2.92E-06 | NPAS4 |
| ENSG00000196208 | 168.0461045 | 1.057405357 | 3.22E-07 | 2.97E-06 | GREB1 |
| ENSG00000265743 | 54.28531606 | 1.97827753 | 3.23E-07 | 2.97E-06 | LINC02978 |
| ENSG00000263843 | 31.46193134 | -2.335958016 | 3.30E-07 | 3.04E-06 | MIF4GD-DT |
| ENSG00000126460 | 18.31067712 | 2.948484756 | 3.34E-07 | 3.07E-06 | PRRG2 |
| ENSG00000185666 | 22.49838579 | -2.342784479 | 3.44E-07 | 3.16E-06 | SYN3 |
| ENSG00000198848 | 204.7949663 | -1.031434959 | 3.44E-07 | 3.16E-06 | CES1 |
| ENSG00000100413 | 438.038146 | -1.044129652 | 3.52E-07 | 3.22E-06 | POLR3H |
| ENSG00000133063 | 38.06474407 | 2.089835989 | 3.53E-07 | 3.24E-06 | CHIT1 |
| ENSG00000115884 | 30.592775 | 2.779176654 | 3.58E-07 | 3.27E-06 | SDC1 |
| ENSG00000171246 | 579.0920655 | 1.986876584 | 3.77E-07 | 3.44E-06 | NPTX1 |
| ENSG00000150967 | 98.31585045 | -1.181884829 | 3.78E-07 | 3.45E-06 | ABCB9 |
| ENSG00000128918 | 30.46114581 | -1.960180125 | 3.83E-07 | 3.49E-06 | ALDH1A2 |
| ENSG00000155016 | 71.52724416 | -1.262295996 | 3.83E-07 | 3.49E-06 | CYP2U1 |
| ENSG00000104213 | 39.44691228 | 2.191023045 | 3.89E-07 | 3.55E-06 | PDGFRL |
| ENSG00000253522 | 66.224917 | -1.350549509 | 4.04E-07 | 3.68E-06 | MIR3142HG |
| ENSG00000171130 | 76.37142885 | -1.239174506 | 4.06E-07 | 3.69E-06 | ATP6V0E2 |
| ENSG00000227487 | 123.2850923 | 1.003762002 | 4.10E-07 | 3.73E-06 | NCAM1-AS1 |
| ENSG00000236901 | 212.7132408 | 1.206310933 | 4.20E-07 | 3.81E-06 | MIR600HG |
| ENSG00000212743 | 10.01008488 | 6.811218134 | 4.33E-07 | 3.92E-06 | LINC02656 |
| ENSG00000105327 | 127.9545229 | 1.62032059 | 4.49E-07 | 4.05E-06 | BBC3 |
| ENSG00000112039 | 211.9399871 | -1.15686267 | 4.64E-07 | 4.18E-06 | FANCE |
| ENSG00000172244 | 208.122303 | 1.121762892 | 4.68E-07 | 4.21E-06 | C5orf34 |
| ENSG00000233217 | 17.87936372 | -2.843685684 | 4.79E-07 | 4.30E-06 | MROH3P |
| ENSG00000172819 | 506.8404669 | -1.05402042 | 5.11E-07 | 4.57E-06 | RARG |
| ENSG00000204792 | 125.2408838 | -1.256658996 | 5.11E-07 | 4.57E-06 | LINC01291 |
| ENSG00000108700 | 8.932108836 | -6.587845701 | 5.16E-07 | 4.61E-06 | CCL8 |
| ENSG00000147647 | 42.36910733 | -1.675309136 | 5.16E-07 | 4.61E-06 | DPYS |
| ENSG00000117602 | 68.56903396 | -1.314137991 | 5.37E-07 | 4.79E-06 | RCAN3 |
| ENSG00000133392 | 60.66807227 | -1.324487359 | 5.50E-07 | 4.89E-06 | MYH11 |
| ENSG00000143156 | 67.92771625 | -1.294629193 | 5.88E-07 | 5.20E-06 | NME7 |
| ENSG00000237036 | 74.88402651 | 1.191236676 | 5.94E-07 | 5.25E-06 | ZEB1-AS1 |
| ENSG00000166106 | 52.41092538 | 2.082884679 | 6.44E-07 | 5.66E-06 | ADAMTS15 |
| ENSG00000127530 | 8.78677691 | -6.563684377 | 6.49E-07 | 5.70E-06 | OR7C1 |
| ENSG00000166426 | 59.88888355 | -1.400670754 | 6.53E-07 | 5.74E-06 | CRABP1 |
| ENSG00000166793 | 17.18441762 | 2.977759996 | 6.54E-07 | 5.74E-06 | YPEL4 |
| ENSG00000173930 | 25.5697493 | -2.478340378 | 6.60E-07 | 5.78E-06 | SLCO4C1 |
| ENSG00000143226 | 143.5279854 | -1.077493647 | 6.79E-07 | 5.93E-06 | FCGR2A |
| ENSG00000074966 | 92.41491974 | -1.608341492 | 6.79E-07 | 5.93E-06 | TXK |
| ENSG00000182108 | 90.44051018 | 1.210598947 | 6.88E-07 | 6.01E-06 | DEXI |
| ENSG00000162849 | 91.2098999 | -1.196747986 | 6.97E-07 | 6.08E-06 | KIF26B |
| ENSG00000108932 | 103.9382503 | 1.629582895 | 6.98E-07 | 6.08E-06 | SLC16A6 |
| ENSG00000100031 | 85.50877064 | 1.434754355 | 7.01E-07 | 6.11E-06 | GGT1 |
| ENSG00000236008 | 84.45886509 | -1.110298362 | 7.04E-07 | 6.13E-06 | LINC01814 |
| ENSG00000133110 | 8.251805296 | -6.474612889 | 7.20E-07 | 6.26E-06 | POSTN |
| ENSG00000154040 | 52.41233211 | -1.791783046 | 7.21E-07 | 6.28E-06 | CABYR |
| ENSG00000185442 | 62.70590521 | -1.313909143 | 7.22E-07 | 6.28E-06 | FAM174B |
| ENSG00000147570 | 87.11613096 | 1.331437919 | 7.44E-07 | 6.46E-06 | DNAJC5B |
| ENSG00000006638 | 122.5619613 | -1.176698008 | 7.53E-07 | 6.53E-06 | TBXA2R |
| ENSG00000267796 | 98.33669564 | 1.03977212 | 8.02E-07 | 6.92E-06 | LIN37 |
| ENSG00000243742 | 67.84618268 | 1.406810929 | 8.09E-07 | 6.97E-06 | RPLP0P2 |
| ENSG00000168542 | 13.92703093 | -6.250579336 | 8.25E-07 | 7.10E-06 | COL3A1 |
| ENSG00000145358 | 2142.356528 | 2.057220237 | 8.42E-07 | 7.23E-06 | DDIT4L |
| ENSG00000129226 | 53.5971265 | 1.492218497 | 8.74E-07 | 7.48E-06 | CD68 |
| ENSG00000006652 | 402.6879013 | 1.135420037 | 8.76E-07 | 7.49E-06 | IFRD1 |
| ENSG00000238164 | 44.48873704 | 1.912977823 | 8.89E-07 | 7.60E-06 | TNFRSF14-AS1 |
| ENSG00000224957 | 31.27698189 | -1.966293075 | 8.95E-07 | 7.65E-06 | LINC01266 |
| ENSG00000188026 | 29.09321997 | 2.763099613 | 9.29E-07 | 7.93E-06 | RILPL1 |
| ENSG00000073670 | 96.93323681 | -1.097554896 | 9.44E-07 | 8.04E-06 | ADAM11 |
| ENSG00000205978 | 49.14559873 | -1.436178175 | 9.58E-07 | 8.16E-06 | NYNRIN |
| ENSG00000197594 | 13.4362612 | -6.198041106 | 9.75E-07 | 8.30E-06 | ENPP1 |
| ENSG00000105855 | 29.64190957 | -2.165108725 | 9.97E-07 | 8.47E-06 | ITGB8 |
| ENSG00000077152 | 154.4800888 | -1.017524538 | 1.01E-06 | 8.56E-06 | UBE2T |
| ENSG00000172349 | 375.4102977 | -1.877740483 | 1.01E-06 | 8.59E-06 | IL16 |
| ENSG00000145476 | 207.8836279 | -1.024590011 | 1.02E-06 | 8.62E-06 | CYP4V2 |
| ENSG00000162148 | 91.57281121 | 1.032706168 | 1.07E-06 | 9.01E-06 | PPP1R32 |
| ENSG00000249096 | 29.2281495 | 2.219264022 | 1.08E-06 | 9.12E-06 | LINC02362 |
| ENSG00000089847 | 116.7548313 | 1.067703186 | 1.10E-06 | 9.22E-06 | ANKRD24 |
| ENSG00000107201 | 90.45600728 | -1.102656286 | 1.11E-06 | 9.35E-06 | DDX58 |
| ENSG00000163273 | 13.86168915 | 3.284991176 | 1.14E-06 | 9.57E-06 | NPPC |
| ENSG00000126822 | 78.32593257 | -1.148180855 | 1.14E-06 | 9.60E-06 | PLEKHG3 |
| ENSG00000141497 | 22.10831986 | 2.302169395 | 1.15E-06 | 9.64E-06 | ZMYND15 |
| ENSG00000204131 | 13.86181544 | -3.948651835 | 1.17E-06 | 9.79E-06 | NHSL2 |
| ENSG00000139438 | 28.14510256 | -2.135835227 | 1.20E-06 | 1.00E-05 | FAM222A |
| ENSG00000149927 | 91.98174711 | 1.363782643 | 1.23E-06 | 1.03E-05 | DOC2A |
| ENSG00000061337 | 52.91960202 | -1.413896975 | 1.24E-06 | 1.03E-05 | LZTS1 |
| ENSG00000108813 | 77.33949771 | -1.175415687 | 1.28E-06 | 1.07E-05 | DLX4 |
| ENSG00000165124 | 45.70486 | -1.607979718 | 1.28E-06 | 1.07E-05 | SVEP1 |
| ENSG00000163596 | 71.08003026 | -1.330051831 | 1.33E-06 | 1.10E-05 | ICA1L |
| ENSG00000125657 | 250.8586867 | 1.238456809 | 1.34E-06 | 1.11E-05 | TNFSF9 |
| ENSG00000187860 | 22.4900495 | -2.192143567 | 1.34E-06 | 1.11E-05 | CCDC157 |
| ENSG00000138587 | 22.534723 | -2.265786963 | 1.42E-06 | 1.17E-05 | MNS1 |
| ENSG00000180871 | 100.3460138 | -1.28480483 | 1.42E-06 | 1.17E-05 | CXCR2 |
| ENSG00000008394 | 1432.293457 | 1.641655231 | 1.44E-06 | 1.19E-05 | MGST1 |
| ENSG00000204682 | 19.18618382 | -2.421017907 | 1.49E-06 | 1.22E-05 | MIR1915HG |
| ENSG00000108924 | 12.30025784 | -6.06924532 | 1.53E-06 | 1.25E-05 | HLF |
| ENSG00000255666 | 113.6761207 | -1.519544479 | 1.54E-06 | 1.26E-05 | LINC02700 |
| ENSG00000156509 | 33.97598792 | -1.762808081 | 1.54E-06 | 1.26E-05 | FBXO43 |
| ENSG00000164687 | 2315.139851 | 1.454937647 | 1.60E-06 | 1.31E-05 | FABP5 |
| ENSG00000112246 | 48.73991171 | 1.443923268 | 1.62E-06 | 1.32E-05 | SIM1 |
| ENSG00000238365 | 27.15497357 | 1.977634579 | 1.64E-06 | 1.34E-05 | RNU7-57P |
| ENSG00000102554 | 146.229751 | -1.050827521 | 1.66E-06 | 1.35E-05 | KLF5 |
| ENSG00000167653 | 21.55184248 | 2.34142461 | 1.66E-06 | 1.35E-05 | PSCA |
| ENSG00000073282 | 51.47583394 | -1.425007571 | 1.69E-06 | 1.37E-05 | TP63 |
| ENSG00000257354 | 160.4375632 | -1.1233263 | 1.74E-06 | 1.41E-05 | MIRLET7IHG |
| ENSG00000166349 | 29.84341532 | -2.026044451 | 1.76E-06 | 1.42E-05 | RAG1 |
| ENSG00000135898 | 18.31177053 | -2.764911368 | 1.80E-06 | 1.45E-05 | GPR55 |
| ENSG00000240891 | 32.83801935 | -1.779062992 | 1.83E-06 | 1.48E-05 | PLCXD2 |
| ENSG00000259205 | 63.35636531 | -1.302006489 | 1.94E-06 | 1.56E-05 | PRKXP1 |
| ENSG00000115009 | 1718.341464 | 1.592800454 | 1.97E-06 | 1.58E-05 | CCL20 |
| ENSG00000184786 | 97.85956067 | -1.071518441 | 2.02E-06 | 1.62E-05 | DYNLT2 |
| ENSG00000204653 | 120.7305314 | 1.216249027 | 2.10E-06 | 1.68E-05 | ASPDH |
| ENSG00000162174 | 23.14627472 | -2.310613671 | 2.11E-06 | 1.68E-05 | ASRGL1 |
| ENSG00000166788 | 141.3869406 | -1.068062574 | 2.12E-06 | 1.69E-05 | SAAL1 |
| ENSG00000126351 | 609.4610375 | 2.033610783 | 2.25E-06 | 1.79E-05 | THRA |
| ENSG00000164331 | 184.4301525 | 1.004814118 | 2.27E-06 | 1.80E-05 | ANKRA2 |
| ENSG00000133111 | 94.06963507 | -1.315597033 | 2.30E-06 | 1.82E-05 | RFXAP |
| ENSG00000179111 | 20.04290311 | 2.749652325 | 2.37E-06 | 1.87E-05 | HES7 |
| ENSG00000266274 | 37.77536236 | 1.764353557 | 2.41E-06 | 1.90E-05 | RN7SL138P |
| ENSG00000135929 | 25.15963609 | 1.949079902 | 2.42E-06 | 1.91E-05 | CYP27A1 |
| ENSG00000160323 | 95.74121601 | 1.057565781 | 2.42E-06 | 1.91E-05 | ADAMTS13 |
| ENSG00000125869 | 33.94502437 | -1.87898746 | 2.47E-06 | 1.94E-05 | LAMP5 |
| ENSG00000168477 | 158.326175 | 1.486488955 | 2.52E-06 | 1.98E-05 | TNXB |
| ENSG00000226806 | 27.01389592 | -2.496966816 | 2.56E-06 | 2.00E-05 | LCT-AS1 |
| ENSG00000115641 | 138.2907462 | -1.097717289 | 2.56E-06 | 2.01E-05 | FHL2 |
| ENSG00000224137 | 60.70793196 | 1.494278095 | 2.57E-06 | 2.02E-05 | LINC01857 |
| ENSG00000138646 | 121.1811627 | -1.050569843 | 2.63E-06 | 2.06E-05 | HERC5 |
| ENSG00000168939 | 96.63669808 | -1.625663499 | 2.65E-06 | 2.07E-05 | SPRY3 |
| ENSG00000170323 | 9386.681811 | 3.330682814 | 2.67E-06 | 2.08E-05 | FABP4 |
| ENSG00000135636 | 27.57047867 | 2.470340018 | 2.68E-06 | 2.09E-05 | DYSF |
| ENSG00000088538 | 125.7902926 | 1.153468721 | 2.83E-06 | 2.20E-05 | DOCK3 |
| ENSG00000147166 | 35.60443141 | -1.581888379 | 3.03E-06 | 2.35E-05 | ITGB1BP2 |
| ENSG00000168899 | 34.72727258 | 1.721228249 | 3.11E-06 | 2.40E-05 | VAMP5 |
| ENSG00000163069 | 34.71881064 | -1.649187435 | 3.12E-06 | 2.41E-05 | SGCB |
| ENSG00000105963 | 90.09312139 | -1.291442551 | 3.14E-06 | 2.42E-05 | ADAP1 |
| ENSG00000185905 | 123.6879196 | -1.194844121 | 3.17E-06 | 2.44E-05 | C16orf54 |
| ENSG00000183831 | 15.16338727 | -2.846581631 | 3.17E-06 | 2.45E-05 | ANKRD45 |
| ENSG00000092345 | 24.26644027 | 2.192065634 | 3.17E-06 | 2.45E-05 | DAZL |
| ENSG00000235602 | 20.62650843 | 3.014581169 | 3.26E-06 | 2.51E-05 | POU5F1P3 |
| ENSG00000163607 | 122.3756484 | -1.036315668 | 3.26E-06 | 2.51E-05 | GTPBP8 |
| ENSG00000226752 | 59.98147691 | -1.245559792 | 3.48E-06 | 2.66E-05 | CUTALP |
| ENSG00000118308 | 1754.764807 | -1.352785887 | 3.52E-06 | 2.69E-05 | IRAG2 |
| ENSG00000229331 | 13.81734261 | 3.490866385 | 3.58E-06 | 2.73E-05 | GK-IT1 |
| ENSG00000145864 | 84.14426927 | 1.084605298 | 3.58E-06 | 2.73E-05 | GABRB2 |
| ENSG00000129173 | 92.53046816 | -1.016544549 | 3.67E-06 | 2.79E-05 | E2F8 |
| ENSG00000280987 | 108.3336719 | 1.085162592 | 3.69E-06 | 2.80E-05 | MATR3 |
| ENSG00000164061 | 89.73211862 | 1.12490246 | 3.69E-06 | 2.80E-05 | BSN |
| ENSG00000100302 | 37.0763177 | -2.185739626 | 3.74E-06 | 2.84E-05 | RASD2 |
| ENSG00000105792 | 20.38746351 | 2.324623013 | 3.77E-06 | 2.85E-05 | CFAP69 |
| ENSG00000253649 | 62.79797883 | -1.28309352 | 3.77E-06 | 2.85E-05 | PRSS51 |
| ENSG00000162975 | 17.33574339 | 2.511773255 | 3.80E-06 | 2.88E-05 | KCNF1 |
| ENSG00000107242 | 30.49481982 | -1.918995534 | 3.89E-06 | 2.94E-05 | PIP5K1B |
| ENSG00000285793 | 86.10994886 | 1.223506489 | 4.10E-06 | 3.09E-05 | ANAPC1P2 |
| ENSG00000113657 | 503.2237857 | 1.868710936 | 4.11E-06 | 3.09E-05 | DPYSL3 |
| ENSG00000104332 | 18.09352099 | -2.411860877 | 4.11E-06 | 3.09E-05 | SFRP1 |
| ENSG00000145335 | 34.54453564 | -1.881026758 | 4.12E-06 | 3.10E-05 | SNCA |
| ENSG00000006459 | 904.3283951 | 1.437736423 | 4.14E-06 | 3.11E-05 | KDM7A |
| ENSG00000197816 | 11.83157916 | -4.045789595 | 4.22E-06 | 3.18E-05 | CCDC180 |
| ENSG00000166046 | 44.91487974 | 1.437149756 | 4.31E-06 | 3.23E-05 | TCP11L2 |
| ENSG00000197506 | 7.170752879 | 6.330325703 | 4.37E-06 | 3.27E-05 | SLC28A3 |
| ENSG00000003096 | 13.59742289 | -3.194840336 | 4.40E-06 | 3.29E-05 | KLHL13 |
| ENSG00000101977 | 18.77341937 | -2.581692818 | 4.40E-06 | 3.29E-05 | MCF2 |
| ENSG00000082126 | 19.85229926 | -2.291759332 | 4.43E-06 | 3.31E-05 | MPP4 |
| ENSG00000180573 | 84.0495727 | 1.130456497 | 4.60E-06 | 3.42E-05 | H2AC6 |
| ENSG00000210082 | 124872.906 | 1.278823358 | 4.60E-06 | 3.42E-05 | MT-RNR2 |
| ENSG00000100490 | 125.9214884 | -1.148892743 | 4.64E-06 | 3.45E-05 | CDKL1 |
| ENSG00000106689 | 42.84385845 | -1.489247308 | 4.66E-06 | 3.46E-05 | LHX2 |
| ENSG00000121621 | 105.0710756 | -1.157391157 | 4.73E-06 | 3.51E-05 | KIF18A |
| ENSG00000254102 | 45.09395728 | -1.443075321 | 4.84E-06 | 3.59E-05 | BHLHE22-AS1 |
| ENSG00000124839 | 66.75098088 | -1.542535888 | 4.91E-06 | 3.64E-05 | RAB17 |
| ENSG00000171827 | 180.0480286 | 1.140852017 | 4.94E-06 | 3.66E-05 | ZNF570 |
| ENSG00000179627 | 37.23524043 | -1.844745958 | 4.95E-06 | 3.66E-05 | ZBTB42 |
| ENSG00000064195 | 12.15930363 | -3.221414873 | 4.95E-06 | 3.66E-05 | DLX3 |
| ENSG00000153132 | 45.94029023 | 1.426875418 | 4.96E-06 | 3.66E-05 | CLGN |
| ENSG00000124772 | 67.95177703 | -1.159299767 | 5.17E-06 | 3.81E-05 | CPNE5 |
| ENSG00000111834 | 17.10458242 | -2.534069253 | 5.20E-06 | 3.83E-05 | RSPH4A |
| ENSG00000149743 | 173.2925222 | 1.093868507 | 5.26E-06 | 3.87E-05 | TRPT1 |
| ENSG00000142765 | 49.73214644 | -1.334650266 | 5.29E-06 | 3.89E-05 | SYTL1 |
| ENSG00000172508 | 40.86968671 | 1.532434278 | 5.52E-06 | 4.04E-05 | CARNS1 |
| ENSG00000170500 | 18.54982115 | -2.358251779 | 5.52E-06 | 4.05E-05 | LONRF2 |
| ENSG00000112195 | 10.84305851 | -3.920904228 | 5.54E-06 | 4.06E-05 | TREML2 |
| ENSG00000245904 | 39.3531278 | -1.637536462 | 5.60E-06 | 4.10E-05 | BTG1-DT |
| ENSG00000153495 | 15.13357951 | 3.231274311 | 5.65E-06 | 4.13E-05 | TEX29 |
| ENSG00000177374 | 50.18027731 | -1.430076117 | 5.65E-06 | 4.13E-05 | HIC1 |
| ENSG00000246922 | 81.1774574 | -1.093086339 | 5.74E-06 | 4.19E-05 | UBAP1L |
| ENSG00000104974 | 12.15181389 | -3.223441606 | 6.01E-06 | 4.37E-05 | LILRA1 |
| ENSG00000130592 | 30.17328506 | 1.737872447 | 6.09E-06 | 4.42E-05 | LSP1 |
| ENSG00000179240 | 80.33432412 | -1.219976977 | 6.31E-06 | 4.56E-05 | GVQW3 |
| ENSG00000163827 | 92.83584867 | 1.039412285 | 6.41E-06 | 4.63E-05 | LRRC2 |
| ENSG00000137103 | 27.23544863 | 2.148817011 | 6.66E-06 | 4.79E-05 | TMEM8B |
| ENSG00000153814 | 87.19313498 | -1.154974344 | 6.70E-06 | 4.82E-05 | JAZF1 |
| ENSG00000109436 | 88.26619488 | 1.079001676 | 6.77E-06 | 4.87E-05 | TBC1D9 |
| ENSG00000129195 | 19.52758507 | -2.18170412 | 6.79E-06 | 4.88E-05 | PIMREG |
| ENSG00000237423 | 21.29660145 | -2.60658318 | 7.20E-06 | 5.15E-05 | LINC01522 |
| ENSG00000185133 | 40.15726942 | -1.420210285 | 7.22E-06 | 5.16E-05 | INPP5J |
| ENSG00000171462 | 71.99447843 | -1.102101661 | 7.35E-06 | 5.25E-05 | DLK2 |
| ENSG00000092068 | 494.4873293 | 2.439060511 | 7.36E-06 | 5.26E-05 | SLC7A8 |
| ENSG00000067177 | 108.2674648 | 1.000227995 | 7.71E-06 | 5.48E-05 | PHKA1 |
| ENSG00000239887 | 68.39557539 | 1.340460115 | 7.75E-06 | 5.51E-05 | C1orf226 |
| ENSG00000133069 | 12.49597522 | -3.267176291 | 7.87E-06 | 5.59E-05 | TMCC2 |
| ENSG00000114107 | 131.2257142 | -1.0335259 | 8.05E-06 | 5.70E-05 | CEP70 |
| ENSG00000214050 | 27.02548642 | -1.84997884 | 8.13E-06 | 5.76E-05 | FBXO16 |
| ENSG00000105737 | 35.64968993 | 1.518691147 | 8.20E-06 | 5.80E-05 | GRIK5 |
| ENSG00000116991 | 1020.354998 | 1.465879801 | 8.30E-06 | 5.87E-05 | SIPA1L2 |
| ENSG00000271383 | 70.88521872 | 1.341041174 | 8.33E-06 | 5.89E-05 | NBPF19 |
| ENSG00000187791 | 133.401109 | 1.051860579 | 8.37E-06 | 5.91E-05 | FAM205C |
| ENSG00000163792 | 66.15598605 | -1.117674093 | 8.47E-06 | 5.98E-05 | TCF23 |
| ENSG00000081818 | 46.60369848 | 1.434405814 | 8.51E-06 | 6.01E-05 | PCDHB4 |
| ENSG00000100162 | 70.61698719 | -1.573107112 | 8.51E-06 | 6.01E-05 | CENPM |
| ENSG00000124507 | 14.15933578 | -3.070338622 | 8.53E-06 | 6.02E-05 | PACSIN1 |
| ENSG00000080493 | 36.61858977 | 1.504844864 | 8.86E-06 | 6.23E-05 | SLC4A4 |
| ENSG00000164463 | 727.4663963 | 1.4341212 | 8.94E-06 | 6.29E-05 | CREBRF |
| ENSG00000011052 | 36.00809177 | 1.537012581 | 9.03E-06 | 6.34E-05 | NME1-NME2 |
| ENSG00000144115 | 20.19573454 | -2.081140101 | 9.14E-06 | 6.41E-05 | THNSL2 |
| ENSG00000122574 | 45.43121708 | 1.486652202 | 9.14E-06 | 6.41E-05 | WIPF3 |
| ENSG00000245864 | 20.88851648 | -2.393037271 | 9.37E-06 | 6.55E-05 | MEF2C-AS2 |
| ENSG00000164683 | 68.27157509 | -1.475060041 | 9.49E-06 | 6.63E-05 | HEY1 |
| ENSG00000188133 | 5.976758533 | -6.009790165 | 9.54E-06 | 6.66E-05 | TMEM215 |
| ENSG00000198889 | 50.01540236 | -1.370016638 | 9.69E-06 | 6.75E-05 | DCAF12L1 |
| ENSG00000187068 | 27.61919146 | -1.78049034 | 9.83E-06 | 6.84E-05 | C3orf70 |
| ENSG00000154262 | 15.12025163 | -2.56121615 | 1.00E-05 | 6.96E-05 | ABCA6 |
| ENSG00000104881 | 106.564381 | 1.158809486 | 1.00E-05 | 6.97E-05 | PPP1R13L |
| ENSG00000152253 | 89.59374323 | -1.028922393 | 1.06E-05 | 7.32E-05 | SPC25 |
| ENSG00000130762 | 27.18738467 | 1.878199533 | 1.06E-05 | 7.35E-05 | ARHGEF16 |
| ENSG00000162373 | 16.24538087 | -2.334884045 | 1.08E-05 | 7.45E-05 | BEND5 |
| ENSG00000055732 | 81.14463864 | 1.327794421 | 1.08E-05 | 7.46E-05 | MCOLN3 |
| ENSG00000177707 | 109.8765381 | 1.183819339 | 1.08E-05 | 7.46E-05 | NECTIN3 |
| ENSG00000263293 | 47.97088478 | 1.943175436 | 1.09E-05 | 7.52E-05 | EFCAB13-DT |
| ENSG00000176720 | 24.11109131 | -1.914553563 | 1.09E-05 | 7.52E-05 | BOK |
| ENSG00000204475 | 26.27678884 | -1.906257677 | 1.11E-05 | 7.66E-05 | NCR3 |
| ENSG00000237781 | 53.23104598 | 1.323908698 | 1.13E-05 | 7.80E-05 | ADAMTSL4-AS2 |
| ENSG00000164776 | 61.81908527 | 1.326506166 | 1.14E-05 | 7.83E-05 | PHKG1 |
| ENSG00000106688 | 13.00801964 | -2.753008021 | 1.16E-05 | 7.94E-05 | SLC1A1 |
| ENSG00000128833 | 17.05850872 | -2.318750469 | 1.17E-05 | 8.05E-05 | MYO5C |
| ENSG00000183763 | 85.3915321 | -1.115039377 | 1.19E-05 | 8.13E-05 | TRAIP |
| ENSG00000069702 | 42.34867376 | -1.375995379 | 1.22E-05 | 8.33E-05 | TGFBR3 |
| ENSG00000177854 | 55.72381748 | 1.255105927 | 1.25E-05 | 8.53E-05 | TMEM187 |
| ENSG00000141540 | 79.30632894 | 1.270675142 | 1.27E-05 | 8.63E-05 | TTYH2 |
| ENSG00000104808 | 40.32008947 | 1.854232442 | 1.28E-05 | 8.75E-05 | DHDH |
| ENSG00000146648 | 21.2511108 | -2.488936049 | 1.29E-05 | 8.78E-05 | EGFR |
| ENSG00000236682 | 59.85259097 | -1.260832584 | 1.31E-05 | 8.88E-05 | MAP3K2-DT |
| ENSG00000226791 | 11.40809758 | -3.64913467 | 1.32E-05 | 8.98E-05 | LINC02611 |
| ENSG00000153790 | 84.91065173 | -1.005741581 | 1.34E-05 | 9.07E-05 | C7orf31 |
| ENSG00000242193 | 111.9231875 | -1.124092534 | 1.34E-05 | 9.07E-05 | CRYZL2P |
| ENSG00000117013 | 502.6418592 | 2.104455397 | 1.35E-05 | 9.13E-05 | KCNQ4 |
| ENSG00000096654 | 108.774991 | 1.081489775 | 1.36E-05 | 9.18E-05 | ZNF184 |
| ENSG00000145491 | 9.225368552 | -5.647356201 | 1.38E-05 | 9.32E-05 | ROPN1L |
| ENSG00000091986 | 12.82513053 | -2.908793786 | 1.40E-05 | 9.46E-05 | CCDC80 |
| ENSG00000258704 | 49.24308689 | 1.325614929 | 1.46E-05 | 9.82E-05 | SRP54-AS1 |
| ENSG00000144648 | 17.69004511 | -2.276338203 | 1.46E-05 | 9.82E-05 | ACKR2 |
| ENSG00000184261 | 37.87301112 | -1.428451331 | 1.46E-05 | 9.86E-05 | KCNK12 |
| ENSG00000153885 | 93.01282919 | -1.004352663 | 1.46E-05 | 9.86E-05 | KCTD15 |
| ENSG00000236591 | 26.90690133 | -1.736736182 | 1.51E-05 | 0.000101723 | UST-AS2 |
| ENSG00000227212 | 143.0373725 | 1.027315089 | 1.57E-05 | 0.000104953 | PFN1P6 |
| ENSG00000087253 | 2899.215297 | -1.324124643 | 1.57E-05 | 0.000104953 | LPCAT2 |
| ENSG00000159640 | 33.83862029 | 1.519207605 | 1.61E-05 | 0.000107557 | ACE |
| ENSG00000150681 | 9.722945549 | -4.193636293 | 1.66E-05 | 0.000110829 | RGS18 |
| ENSG00000103569 | 52.24270047 | 1.771454273 | 1.69E-05 | 0.000112127 | AQP9 |
| ENSG00000151882 | 61.5731918 | -1.283318371 | 1.70E-05 | 0.000112906 | CCL28 |
| ENSG00000235072 | 21.61846717 | -1.986833911 | 1.71E-05 | 0.000113236 | ARNILA |
| ENSG00000165474 | 14.52384443 | 2.43559927 | 1.73E-05 | 0.000114704 | GJB2 |
| ENSG00000002726 | 53.22757825 | -1.54929862 | 1.75E-05 | 0.000115771 | AOC1 |
| ENSG00000128284 | 26.19488352 | 1.755470942 | 1.76E-05 | 0.000116213 | APOL3 |
| ENSG00000148926 | 451.8670455 | 1.639167275 | 1.77E-05 | 0.000116999 | ADM |
| ENSG00000160683 | 10.36868646 | -4.899094108 | 1.81E-05 | 0.00011957 | CXCR5 |
| ENSG00000140398 | 213.6982524 | -1.0698448 | 1.81E-05 | 0.000119623 | NEIL1 |
| ENSG00000152503 | 97.25888015 | -1.005439922 | 1.81E-05 | 0.000119623 | TRIM36 |
| ENSG00000197181 | 34.85197362 | 2.0851166 | 1.82E-05 | 0.000120099 | PIWIL2 |
| ENSG00000179213 | 10.21269855 | -4.879581919 | 1.83E-05 | 0.000120416 | SIGLECL1 |
| ENSG00000173638 | 333.4593922 | -1.023570421 | 1.85E-05 | 0.000121541 | SLC19A1 |
| ENSG00000178202 | 224.5634773 | -2.042670482 | 1.87E-05 | 0.000122938 | POGLUT3 |
| ENSG00000186439 | 23.67543357 | 2.207475439 | 1.87E-05 | 0.000123009 | TRDN |
| ENSG00000186765 | 75.95726814 | 1.035007467 | 1.87E-05 | 0.000123059 | FSCN2 |
| ENSG00000137642 | 2861.440617 | -1.264156525 | 1.88E-05 | 0.000123285 | SORL1 |
| ENSG00000135617 | 80.08643044 | -1.102147691 | 1.89E-05 | 0.000123886 | PRADC1 |
| ENSG00000197629 | 18.04121256 | -2.216418495 | 1.91E-05 | 0.000124874 | MPEG1 |
| ENSG00000118402 | 9.546726425 | -4.166397701 | 2.00E-05 | 0.000130801 | ELOVL4 |
| ENSG00000127074 | 227.4298785 | -1.929735619 | 2.25E-05 | 0.000145967 | RGS13 |
| ENSG00000007968 | 46.48668511 | -1.317664867 | 2.28E-05 | 0.000147789 | E2F2 |
| ENSG00000255624 | 26.79997661 | -1.727951291 | 2.32E-05 | 0.000150022 | C10orf88B |
| ENSG00000144857 | 18.99854705 | -2.722014457 | 2.32E-05 | 0.000150386 | BOC |
| ENSG00000103044 | 49.15394371 | -1.283850206 | 2.37E-05 | 0.000153314 | HAS3 |
| ENSG00000156232 | 1936.449661 | 1.462059906 | 2.39E-05 | 0.000153991 | WHAMM |
| ENSG00000108852 | 67.17068245 | -1.034686979 | 2.47E-05 | 0.000159055 | MPP2 |
| ENSG00000153086 | 20.95337106 | 1.929290895 | 2.54E-05 | 0.000163403 | ACMSD |
| ENSG00000196372 | 100.3798383 | -1.058243728 | 2.60E-05 | 0.000166778 | ASB13 |
| ENSG00000257718 | 23.12524617 | -2.031274893 | 2.63E-05 | 0.000168706 | CPNE8-AS1 |
| ENSG00000269404 | 10.64911031 | -3.548396117 | 2.66E-05 | 0.00017003 | SPIB |
| ENSG00000254122 | 37.88608779 | -1.365444137 | 2.74E-05 | 0.000175202 | PCDHGB7 |
| ENSG00000104970 | 5.777943348 | -5.962487687 | 2.77E-05 | 0.00017658 | KIR3DX1 |
| ENSG00000154957 | 60.98683781 | 1.172602758 | 2.78E-05 | 0.000177239 | ZNF18 |
| ENSG00000221817 | 135.4166946 | -1.294759114 | 2.84E-05 | 0.000180795 | PPP3CB-AS1 |
| ENSG00000105767 | 83.46339704 | 1.197984366 | 2.92E-05 | 0.000185396 | CADM4 |
| ENSG00000079739 | 52.99266251 | -1.274212937 | 2.95E-05 | 0.000187054 | PGM1 |
| ENSG00000070759 | 493.637083 | 1.543546069 | 3.00E-05 | 0.000189691 | TESK2 |
| ENSG00000222047 | 52.6829791 | -1.137217412 | 3.04E-05 | 0.000192163 | C10orf55 |
| ENSG00000173762 | 9.193003242 | 4.167250014 | 3.09E-05 | 0.000194698 | CD7 |
| ENSG00000263528 | 772.8325524 | -1.298056308 | 3.19E-05 | 0.000200551 | IKBKE |
| ENSG00000214922 | 52.16533636 | 1.753900647 | 3.22E-05 | 0.000202344 | HLA-F-AS1 |
| ENSG00000225783 | 24.59284696 | 2.036736339 | 3.22E-05 | 0.000202515 | MIAT |
| ENSG00000186300 | 258.084603 | 1.059564852 | 3.25E-05 | 0.000203841 | ZNF555 |
| ENSG00000242600 | 28.38767554 | -1.728711448 | 3.35E-05 | 0.000209825 | MBL1P |
| ENSG00000164176 | 32.04928687 | -1.701741831 | 3.43E-05 | 0.000214465 | EDIL3 |
| ENSG00000158813 | 14.0074964 | 2.501644697 | 3.46E-05 | 0.000215779 | EDA |
| ENSG00000166450 | 23.92463733 | 2.22474298 | 3.48E-05 | 0.000217287 | PRTG |
| ENSG00000196139 | 727.2023512 | -1.644269005 | 3.57E-05 | 0.000221963 | AKR1C3 |
| ENSG00000087842 | 168.683878 | 1.025475111 | 3.57E-05 | 0.000222333 | PIR |
| ENSG00000085449 | 3365.506573 | 1.141916302 | 3.63E-05 | 0.000225972 | WDFY1 |
| ENSG00000104888 | 652.651616 | 1.448650809 | 3.78E-05 | 0.000234299 | SLC17A7 |
| ENSG00000281005 | 78.1055526 | -1.023463827 | 3.80E-05 | 0.000235415 | LINC00921 |
| ENSG00000236778 | 85.88754559 | 1.012960382 | 3.89E-05 | 0.000240554 | INTS6-AS1 |
| ENSG00000197077 | 52.94868028 | 1.163501773 | 3.91E-05 | 0.000241682 | KIAA1671 |
| ENSG00000162600 | 83.66419237 | 1.067051688 | 3.94E-05 | 0.000242973 | OMA1 |
| ENSG00000261126 | 34.91686703 | -1.5049026 | 3.97E-05 | 0.000245249 | RBFADN |
| ENSG00000170214 | 4.864112555 | -5.711414499 | 4.06E-05 | 0.000250113 | ADRA1B |
| ENSG00000205592 | 10.38714059 | -3.495654777 | 4.10E-05 | 0.000252266 | MUC19 |
| ENSG00000234964 | 1135.961828 | 1.686956023 | 4.10E-05 | 0.000252266 | FABP5P7 |
| ENSG00000175147 | 32.72964416 | 1.459414339 | 4.15E-05 | 0.000255119 | TMEM51-AS1 |
| ENSG00000242960 | 24.04906336 | 1.75304576 | 4.25E-05 | 0.000260805 | FTH1P23 |
| ENSG00000253368 | 8.916177691 | -4.061632924 | 4.25E-05 | 0.000261071 | TRNP1 |
| ENSG00000164440 | 89.34882448 | -1.106352567 | 4.26E-05 | 0.000261233 | TXLNB |
| ENSG00000134013 | 30.3540143 | -1.722925071 | 4.26E-05 | 0.000261375 | LOXL2 |
| ENSG00000253641 | 76.2461967 | -1.003036741 | 4.27E-05 | 0.000261764 | LINC03022 |
| ENSG00000186638 | 85.81173184 | -1.010061685 | 4.30E-05 | 0.000263534 | KIF24 |
| ENSG00000049769 | 52.23433294 | -1.301982592 | 4.31E-05 | 0.000263803 | PPP1R3F |
| ENSG00000162897 | 11.31905424 | -2.893903145 | 4.36E-05 | 0.000266638 | FCAMR |
| ENSG00000235888 | 24.35223768 | -1.823174493 | 4.39E-05 | 0.000268178 | LINC02940 |
| ENSG00000114737 | 61.43227301 | 1.203663336 | 4.39E-05 | 0.000268178 | CISH |
| ENSG00000100311 | 80.51607309 | 1.105465522 | 4.40E-05 | 0.000268909 | PDGFB |
| ENSG00000165996 | 13.9850781 | -2.431498273 | 4.42E-05 | 0.000269816 | HACD1 |
| ENSG00000237522 | 113.2417238 | 1.154241081 | 4.43E-05 | 0.00027007 | NONOP2 |
| ENSG00000227359 | 21.24232922 | -1.810085147 | 4.63E-05 | 0.000281179 | LINC02936 |
| ENSG00000254521 | 7.626212872 | -5.366283292 | 4.66E-05 | 0.000282889 | SIGLEC12 |
| ENSG00000183853 | 11.10014311 | -3.070909887 | 4.77E-05 | 0.000289476 | KIRREL1 |
| ENSG00000245148 | 39.59604805 | 1.317239388 | 4.82E-05 | 0.000292068 | ARAP1-AS2 |
| ENSG00000138100 | 66.27560853 | 1.170868891 | 4.86E-05 | 0.000294499 | TRIM54 |
| ENSG00000134853 | 26.71101483 | -1.976826561 | 4.92E-05 | 0.000297605 | PDGFRA |
| ENSG00000137338 | 40.93731159 | -1.461213998 | 4.97E-05 | 0.00030039 | PGBD1 |
| ENSG00000231064 | 42.26331245 | 1.256474715 | 4.98E-05 | 0.000300869 | THBS3-AS1 |
| ENSG00000113924 | 104.9187671 | 1.41419933 | 5.01E-05 | 0.000302351 | HGD |
| ENSG00000275183 | 34.01983297 | 1.572075377 | 5.07E-05 | 0.00030535 | LENG9 |
| ENSG00000187601 | 83.57618077 | 1.083990992 | 5.11E-05 | 0.000307374 | MAGEH1 |
| ENSG00000119608 | 20.39542059 | -1.804806885 | 5.12E-05 | 0.000307891 | PROX2 |
| ENSG00000154133 | 28.20841416 | 1.660588511 | 5.12E-05 | 0.000308066 | ROBO4 |
| ENSG00000163217 | 8.714898009 | 3.636638219 | 5.15E-05 | 0.00030962 | BMP10 |
| ENSG00000126778 | 33.45151866 | 1.463163067 | 5.17E-05 | 0.00031099 | SIX1 |
| ENSG00000232354 | 13.95290241 | -2.426478588 | 5.21E-05 | 0.000312878 | VIPR1-AS1 |
| ENSG00000140545 | 128.2908852 | -1.003253488 | 5.22E-05 | 0.00031316 | MFGE8 |
| ENSG00000124602 | 42.96278083 | 1.739392469 | 5.25E-05 | 0.000314926 | UNC5CL |
| ENSG00000175003 | 17.83106355 | 2.005035124 | 5.33E-05 | 0.000319354 | SLC22A1 |
| ENSG00000139364 | 66.46447037 | -1.207996292 | 5.34E-05 | 0.000319474 | TMEM132B |
| ENSG00000162913 | 20.02106655 | -1.91249421 | 5.41E-05 | 0.000323041 | OBSCN-AS1 |
| ENSG00000233038 | 18.25545194 | -2.140614754 | 5.45E-05 | 0.000325802 | PTPRN2-AS1 |
| ENSG00000110077 | 68.69002624 | -1.341866887 | 5.60E-05 | 0.000333722 | MS4A6A |
| ENSG00000204086 | 9.231282225 | -3.323427157 | 5.69E-05 | 0.000338942 | RPA4 |
| ENSG00000253159 | 38.22710112 | -1.419567303 | 5.80E-05 | 0.000344841 | PCDHGA12 |
| ENSG00000230587 | 16.92211726 | -2.193827137 | 5.81E-05 | 0.000345503 | LINC02580 |
| ENSG00000042980 | 63.20858417 | 1.195575383 | 5.87E-05 | 0.000348801 | ADAM28 |
| ENSG00000237638 | 48.29463577 | -1.22169468 | 6.14E-05 | 0.000363409 | LINC02245 |
| ENSG00000198929 | 38.29746197 | 1.657019805 | 6.18E-05 | 0.000365914 | NOS1AP |
| ENSG00000090382 | 72.44991516 | -1.050282571 | 6.20E-05 | 0.00036653 | LYZ |
| ENSG00000223485 | 50.13218185 | -1.166660263 | 6.21E-05 | 0.000367333 | LINC01615 |
| ENSG00000170421 | 10.75577854 | -2.80409671 | 6.38E-05 | 0.00037636 | KRT8 |
| ENSG00000197933 | 80.7876046 | 1.003461415 | 6.39E-05 | 0.000376681 | ZNF823 |
| ENSG00000082458 | 16.92429298 | -2.109608292 | 6.40E-05 | 0.000377066 | DLG3 |
| ENSG00000103942 | 90.25672121 | -1.028727906 | 6.42E-05 | 0.000378163 | HOMER2 |
| ENSG00000188385 | 10.34870657 | 3.030773905 | 6.44E-05 | 0.000378952 | JAKMIP3 |
| ENSG00000267046 | 4.67282939 | -5.655480423 | 6.50E-05 | 0.000382489 | E2F3P1 |
| ENSG00000212541 | 12.4970806 | -2.691180871 | 6.61E-05 | 0.000388474 | RNU6-510P |
| ENSG00000172322 | 4.867232118 | -5.712293164 | 6.65E-05 | 0.000390822 | CLEC12A |
| ENSG00000285533 | 45.52674242 | 1.208376941 | 6.82E-05 | 0.000399615 | RELA-DT |
| ENSG00000183166 | 35.84096281 | -1.31746428 | 6.90E-05 | 0.000404259 | CALN1 |
| ENSG00000015520 | 9.636887024 | 4.240011407 | 6.99E-05 | 0.000408411 | NPC1L1 |
| ENSG00000237149 | 43.67538579 | 1.249988359 | 7.02E-05 | 0.000410385 | ZNF503-AS2 |
| ENSG00000258667 | 49.11730961 | 1.386053652 | 7.03E-05 | 0.000410655 | HIF1A-AS3 |
| ENSG00000106123 | 14.9646213 | -2.557496097 | 7.22E-05 | 0.000420232 | EPHB6 |
| ENSG00000188687 | 55.58230846 | -1.294877448 | 7.22E-05 | 0.000420299 | SLC4A5 |
| ENSG00000135298 | 12.72817887 | 2.775412881 | 7.39E-05 | 0.000429036 | ADGRB3 |
| ENSG00000100479 | 86.44614109 | -1.036682365 | 7.63E-05 | 0.000442329 | POLE2 |
| ENSG00000249855 | 35.76031804 | 1.392065159 | 7.74E-05 | 0.00044803 | EEF1A1P19 |
| ENSG00000162687 | 46.37095699 | -1.194702485 | 7.76E-05 | 0.000448634 | KCNT2 |
| ENSG00000203721 | 23.12639107 | 1.791706245 | 7.86E-05 | 0.000453285 | LINC00862 |
| ENSG00000158470 | 3300.335207 | 1.112504081 | 7.87E-05 | 0.000453749 | B4GALT5 |
| ENSG00000130513 | 5588.263772 | 1.511937436 | 7.95E-05 | 0.000457928 | GDF15 |
| ENSG00000135625 | 10.40088611 | -2.752692765 | 7.99E-05 | 0.000460174 | EGR4 |
| ENSG00000154917 | 159.7953265 | -1.022697032 | 8.22E-05 | 0.000472695 | RAB6B |
| ENSG00000228393 | 131.4371825 | -1.064822613 | 8.36E-05 | 0.000480103 | LINC01004 |
| ENSG00000263327 | 27.59287711 | -1.721517206 | 8.41E-05 | 0.000482465 | TAPT1-AS1 |
| ENSG00000205837 | 16.54148839 | -2.159795432 | 8.41E-05 | 0.000482465 | LINC00487 |
| ENSG00000179813 | 84.08851364 | -1.070003267 | 8.43E-05 | 0.00048339 | FAM216B |
| ENSG00000188766 | 92.29600394 | 1.415339949 | 8.54E-05 | 0.000489053 | SPRED3 |
| ENSG00000171843 | 60.83512255 | -1.10288073 | 8.56E-05 | 0.00048989 | MLLT3 |
| ENSG00000154188 | 6.989274784 | -5.236949339 | 8.62E-05 | 0.00049284 | ANGPT1 |
| ENSG00000137177 | 54.83287792 | 1.30059682 | 8.84E-05 | 0.00050496 | KIF13A |
| ENSG00000185674 | 11.56427362 | -2.552047661 | 8.87E-05 | 0.000506683 | LYG2 |
| ENSG00000242265 | 8.401492516 | -4.587378833 | 9.01E-05 | 0.00051373 | PEG10 |
| ENSG00000103494 | 38.31039492 | -1.289990419 | 9.20E-05 | 0.000523552 | RPGRIP1L |
| ENSG00000204388 | 1023.864581 | 1.302976376 | 9.20E-05 | 0.000523639 | HSPA1B |
| ENSG00000206530 | 154.664366 | -1.1172053 | 9.43E-05 | 0.000536027 | CFAP44 |
| ENSG00000064201 | 70.74379031 | -1.003179001 | 9.46E-05 | 0.000537327 | TSPAN32 |
| ENSG00000201183 | 15.14201342 | -2.205462726 | 9.50E-05 | 0.000539296 | RNVU1-3 |
| ENSG00000185022 | 1486.846332 | 1.408080634 | 9.52E-05 | 0.000540244 | MAFF |
| ENSG00000251493 | 58.31202303 | 1.228900457 | 9.70E-05 | 0.000549462 | FOXD1 |
| ENSG00000279141 | 40.46566104 | 1.277667573 | 9.79E-05 | 0.000554297 | LINC01451 |
| ENSG00000197603 | 167.3441355 | -1.021995491 | 9.80E-05 | 0.000554738 | CPLANE1 |
| ENSG00000176894 | 77.63793828 | -1.091437647 | 9.86E-05 | 0.000557982 | PXMP2 |
| ENSG00000102886 | 40.11716932 | 1.876462165 | 0.000100409 | 0.000566615 | GDPD3 |
| ENSG00000109265 | 30.0478685 | 1.861734534 | 0.000100672 | 0.000567839 | CRACD |
| ENSG00000171777 | 28.79703891 | 1.51732088 | 0.000101504 | 0.000572138 | RASGRP4 |
| ENSG00000081059 | 30.43752468 | -1.50874627 | 0.000103353 | 0.000581365 | TCF7 |
| ENSG00000112118 | 1559.214168 | -1.095442255 | 0.000103729 | 0.00058308 | MCM3 |
| ENSG00000253309 | 53.10061151 | -1.478534398 | 0.00010474 | 0.000587955 | SERPINE3 |
| ENSG00000170537 | 52.45129379 | 1.106919957 | 0.000105737 | 0.000593011 | TMC7 |
| ENSG00000163961 | 886.6427534 | 1.331560207 | 0.000107091 | 0.000600197 | RNF168 |
| ENSG00000179085 | 99.64774589 | 1.110075544 | 0.000108153 | 0.000605322 | DPM3 |
| ENSG00000133135 | 13.85086016 | -2.167113776 | 0.000109525 | 0.000612305 | RNF128 |
| ENSG00000231999 | 53.1486895 | -1.130282136 | 0.000110119 | 0.000615488 | LRRC8C-DT |
| ENSG00000182600 | 65.46144329 | 1.015979362 | 0.000113213 | 0.000631346 | SNORC |
| ENSG00000185250 | 18.61376652 | 2.01017229 | 0.000113325 | 0.000631686 | PPIL6 |
| ENSG00000169752 | 26.99626881 | -1.586589453 | 0.000117148 | 0.000651668 | NRG4 |
| ENSG00000105352 | 8.78245565 | -3.235714985 | 0.000119596 | 0.000664084 | CEACAM4 |
| ENSG00000283813 | 15.735245 | 2.985571497 | 0.000121293 | 0.000672601 | MIR4485 |
| ENSG00000109944 | 66.40210664 | -1.104337851 | 0.000121415 | 0.000673126 | JHY |
| ENSG00000109063 | 20.10841008 | -1.777579436 | 0.000123029 | 0.000681317 | MYH3 |
| ENSG00000036565 | 8.784966323 | -3.236033738 | 0.000123773 | 0.000685272 | SLC18A1 |
| ENSG00000083290 | 73.41982483 | -1.003360005 | 0.000124703 | 0.000690109 | ULK2 |
| ENSG00000131849 | 13.35309852 | -2.222125918 | 0.000125173 | 0.000692403 | ZNF132 |
| ENSG00000221598 | 11.53630496 | 2.458134968 | 0.000125642 | 0.000694685 | MIR1249 |
| ENSG00000078098 | 43.13408898 | -1.352974362 | 0.000128976 | 0.000711523 | FAP |
| ENSG00000171444 | 46.95462983 | 1.415602321 | 0.000129119 | 0.000712147 | MCC |
| ENSG00000008086 | 56.1382594 | 1.452054061 | 0.000129638 | 0.000714692 | CDKL5 |
| ENSG00000128606 | 8.807777648 | -3.590540928 | 0.000130945 | 0.000721412 | LRRC17 |
| ENSG00000239264 | 60.58920734 | 1.107596367 | 0.000131185 | 0.000722572 | TXNDC5 |
| ENSG00000171291 | 10.97305025 | 2.909437347 | 0.000131652 | 0.000724741 | ZNF439 |
| ENSG00000159761 | 24.29723634 | 1.610451316 | 0.000132182 | 0.000727092 | C16orf86 |
| ENSG00000118946 | 17.71484501 | -1.92177916 | 0.000132773 | 0.000730012 | PCDH17 |
| ENSG00000134207 | 11.21039448 | 2.570752085 | 0.000133327 | 0.000732732 | SYT6 |
| ENSG00000127951 | 36.52081732 | -1.607113337 | 0.000135577 | 0.000743603 | FGL2 |
| ENSG00000170390 | 24.97651623 | -1.690743872 | 0.00013616 | 0.000746637 | DCLK2 |
| ENSG00000007866 | 17.20394198 | -2.33558153 | 0.000136953 | 0.000750653 | TEAD3 |
| ENSG00000154065 | 24.34403251 | -1.70734671 | 0.000137203 | 0.000751852 | ANKRD29 |
| ENSG00000101353 | 15.46152694 | -2.13399345 | 0.000139331 | 0.000762669 | MROH8 |
| ENSG00000142910 | 4.225956687 | -5.507884421 | 0.000143313 | 0.00078203 | TINAGL1 |
| ENSG00000212123 | 26.09110721 | -1.824208413 | 0.000143585 | 0.00078334 | PRR22 |
| ENSG00000130829 | 31.04568557 | -1.550983698 | 0.000144628 | 0.000788333 | DUSP9 |
| ENSG00000121895 | 26.20741703 | 1.610347881 | 0.00014524 | 0.000791493 | TMEM156 |
| ENSG00000211445 | 24.75349614 | -1.79012109 | 0.000145383 | 0.000791992 | GPX3 |
| ENSG00000177181 | 11.91478733 | -2.442990846 | 0.000146173 | 0.000795524 | RIMKLA |
| ENSG00000091129 | 30.21500516 | 2.279383007 | 0.000146564 | 0.000797471 | NRCAM |
| ENSG00000183250 | 952.6580398 | -1.733965639 | 0.000147267 | 0.000801122 | LINC01547 |
| ENSG00000248449 | 17.05282105 | -2.222362981 | 0.000147914 | 0.000803753 | PCDHGB8P |
| ENSG00000115339 | 20.8897335 | -1.775864591 | 0.0001488 | 0.000807132 | GALNT3 |
| ENSG00000181790 | 47.36078094 | 1.340673884 | 0.000149445 | 0.000809927 | ADGRB1 |
| ENSG00000162924 | 1978.123378 | 1.350773795 | 0.000149927 | 0.000812365 | REL |
| ENSG00000205643 | 54.82090771 | 1.079555585 | 0.000150778 | 0.000816435 | CDPF1 |
| ENSG00000179604 | 126.5811991 | -1.105883546 | 0.000151447 | 0.000819876 | CDC42EP4 |
| ENSG00000161888 | 66.35953829 | -1.151890395 | 0.000151558 | 0.000820299 | SPC24 |
| ENSG00000157510 | 11.66363384 | 2.479289276 | 0.000152496 | 0.000824471 | AFAP1L1 |
| ENSG00000238045 | 48.34781481 | 1.174408655 | 0.000154194 | 0.000833101 | MVP-DT |
| ENSG00000180667 | 1341.008429 | 1.052017467 | 0.000155856 | 0.000840605 | YOD1 |
| ENSG00000215186 | 11.04134722 | 2.537401973 | 0.000163262 | 0.00087671 | GOLGA6B |
| ENSG00000271848 | 8.957328854 | -2.975149767 | 0.000166009 | 0.000889908 | SYNPO2L-AS1 |
| ENSG00000174007 | 81.472568 | -1.044368448 | 0.000167508 | 0.000897751 | CEP19 |
| ENSG00000186666 | 81.92334353 | 1.196360878 | 0.000170274 | 0.00091059 | BCDIN3D |
| ENSG00000261480 | 63.63745797 | 1.002400894 | 0.000171001 | 0.000914081 | GOLGA8M |
| ENSG00000213569 | 38.01285529 | -1.334625026 | 0.000174159 | 0.000928949 | GTF3C6P2 |
| ENSG00000141642 | 91.78577186 | -1.051293549 | 0.000175315 | 0.00093491 | ELAC1 |
| ENSG00000147408 | 27.50672429 | -1.533972579 | 0.000175991 | 0.000938312 | CSGALNACT1 |
| ENSG00000187902 | 63.31576363 | -1.111707035 | 0.000176339 | 0.000939966 | SHISA7 |
| ENSG00000134569 | 42.40973805 | 1.429421638 | 0.00017703 | 0.000943037 | LRP4 |
| ENSG00000160791 | 35.40926937 | -1.355938865 | 0.000178618 | 0.000951086 | CCR5 |
| ENSG00000283646 | 15.52550273 | 2.014372804 | 0.000181332 | 0.000964079 | LINC02009 |
| ENSG00000105649 | 60.70952657 | 1.16639053 | 0.000182085 | 0.000967869 | RAB3A |
| ENSG00000118971 | 61.12219631 | 1.45400416 | 0.000182429 | 0.00096949 | CCND2 |
| ENSG00000158292 | 49.38056905 | -1.356165004 | 0.00018325 | 0.000973348 | GPR153 |
| ENSG00000108641 | 35.27929793 | 1.264995362 | 0.000183273 | 0.000973348 | B9D1 |
| ENSG00000247498 | 19.6764799 | 1.816923215 | 0.000183599 | 0.000974866 | GPRC5D-AS1 |
| ENSG00000185561 | 27.48003896 | -1.439827656 | 0.000185487 | 0.000984469 | TLCD2 |
| ENSG00000237753 | 55.35410286 | -1.078627901 | 0.000187285 | 0.000992728 | SLC20A1-DT |
| ENSG00000099251 | 60.18232149 | 1.160908078 | 0.000187544 | 0.000993887 | HSD17B7P2 |
| ENSG00000168152 | 53.17876266 | 1.195415736 | 0.000191522 | 0.001013443 | THAP9 |
| ENSG00000197429 | 41.69681886 | 1.181052237 | 0.000191671 | 0.00101401 | IPP |
| ENSG00000166578 | 37.69750141 | -1.257807832 | 0.000193663 | 0.00102367 | IQCD |
| ENSG00000016602 | 20.53511121 | -1.883172104 | 0.00019437 | 0.001027189 | CLCA4 |
| ENSG00000081803 | 31.491158 | 1.378670433 | 0.00019738 | 0.001042646 | CADPS2 |
| ENSG00000116833 | 26.1479893 | -1.48134974 | 0.000197662 | 0.001043466 | NR5A2 |
| ENSG00000142632 | 68.59537512 | -1.041615359 | 0.000199539 | 0.001052245 | ARHGEF19 |
| ENSG00000165810 | 31.60061448 | -1.418614001 | 0.000199867 | 0.00105375 | BTNL9 |
| ENSG00000161911 | 589.2568038 | -1.325579521 | 0.000201504 | 0.001062147 | TREML1 |
| ENSG00000079337 | 42.47179796 | 1.115825886 | 0.000201546 | 0.001062147 | RAPGEF3 |
| ENSG00000227082 | 24.29182263 | -1.693470456 | 0.000202754 | 0.001068059 | LINC02798 |
| ENSG00000140044 | 101.581732 | 1.120037582 | 0.000203347 | 0.001070726 | JDP2 |
| ENSG00000110723 | 34.51810577 | 1.319923404 | 0.000204583 | 0.001077002 | EXPH5 |
| ENSG00000267280 | 17.52649279 | 1.891306443 | 0.000205204 | 0.001080037 | TBX2-AS1 |
| ENSG00000154240 | 7.561876977 | -4.42769606 | 0.000208114 | 0.001094652 | CEP112 |
| ENSG00000130783 | 45.87294855 | 1.37509572 | 0.000208784 | 0.001097711 | CCDC62 |
| ENSG00000186831 | 7.264823771 | -4.36620598 | 0.000210261 | 0.001104768 | KRT17P2 |
| ENSG00000109846 | 5.883507402 | 5.039908185 | 0.000218319 | 0.001144422 | CRYAB |
| ENSG00000240429 | 33.73422059 | -1.330245019 | 0.000218481 | 0.00114478 | LRRFIP1P1 |
| ENSG00000155530 | 35.96613099 | -1.430002909 | 0.000221612 | 0.001160447 | LRGUK |
| ENSG00000215267 | 29.03734594 | -1.597203998 | 0.000221894 | 0.001161184 | AKR1C7P |
| ENSG00000285077 | 47.70489223 | -1.105177698 | 0.000222048 | 0.001161746 | ARHGAP11B |
| ENSG00000129167 | 24.36600643 | -1.49065423 | 0.000222334 | 0.001162745 | TPH1 |
| ENSG00000108370 | 21.98382065 | 2.380297615 | 0.000224299 | 0.001172214 | RGS9 |
| ENSG00000267296 | 108.4241157 | 1.318576136 | 0.000224335 | 0.001172214 | CEBPA-DT |
| ENSG00000104883 | 19.27192292 | -1.695249655 | 0.000226088 | 0.00117987 | PEX11G |
| ENSG00000147872 | 20273.02665 | 1.285167253 | 0.000228119 | 0.001189461 | PLIN2 |
| ENSG00000246263 | 64.24306361 | 1.107346936 | 0.000228585 | 0.001191643 | UBR5-DT |
| ENSG00000111305 | 21.18110595 | -1.868198448 | 0.000231552 | 0.001205321 | GSG1 |
| ENSG00000149516 | 7.403973066 | -4.400948447 | 0.000234347 | 0.001218586 | MS4A3 |
| ENSG00000133636 | 7.810982281 | -3.411360609 | 0.000241128 | 0.001251203 | NTS |
| ENSG00000188643 | 13.23422145 | -2.331482471 | 0.000243193 | 0.001259796 | S100A16 |
| ENSG00000262772 | 17.36257405 | -1.891612963 | 0.000243854 | 0.001262953 | LINC01977 |
| ENSG00000245571 | 42.90765159 | 1.243187064 | 0.000244374 | 0.001265118 | FAM111A-DT |
| ENSG00000182022 | 301.2492834 | 1.808291035 | 0.000246985 | 0.001277024 | CHST15 |
| ENSG00000258056 | 53.79794871 | 1.308382145 | 0.000247046 | 0.001277068 | CD63-AS1 |
| ENSG00000142920 | 10.25976779 | -2.530571534 | 0.000247974 | 0.001280523 | AZIN2 |
| ENSG00000163072 | 15.30438846 | -2.025601785 | 0.000248758 | 0.001284003 | NOSTRIN |
| ENSG00000214145 | 7.491333682 | -4.40713685 | 0.000252543 | 0.001300574 | LINC00887 |
| ENSG00000196689 | 42.15311874 | -1.225485104 | 0.000253574 | 0.001305336 | TRPV1 |
| ENSG00000124233 | 7.264158386 | -4.360776744 | 0.000255852 | 0.001316236 | SEMG1 |
| ENSG00000198816 | 75.08406964 | -1.004077804 | 0.000256659 | 0.001320114 | ZNF358 |
| ENSG00000186056 | 48.43283024 | 1.192854068 | 0.000256946 | 0.001321316 | MATN1-AS1 |
| ENSG00000162592 | 24.20000169 | -1.480134445 | 0.000257017 | 0.001321401 | CCDC27 |
| ENSG00000095370 | 168.6579294 | -1.002402611 | 0.00026253 | 0.00134862 | SH2D3C |
| ENSG00000120256 | 44.42676265 | 1.087077996 | 0.000265821 | 0.001362402 | LRP11 |
| ENSG00000183604 | 91.00203456 | 1.171002085 | 0.000265935 | 0.001362703 | SMG1P5 |
| ENSG00000125675 | 34.65699785 | -1.248003267 | 0.000268738 | 0.001374776 | GRIA3 |
| ENSG00000164291 | 109.5856104 | 1.020594354 | 0.000270631 | 0.001383597 | ARSK |
| ENSG00000162888 | 49.79366924 | -1.081021836 | 0.000276395 | 0.001411562 | C1orf147 |
| ENSG00000257529 | 3.686512059 | 5.369420743 | 0.000277532 | 0.001416531 | RPL36A-HNRNPH2 |
| ENSG00000135436 | 40.1252445 | -1.267238459 | 0.000279804 | 0.00142724 | FAM186B |
| ENSG00000272142 | 43.11217252 | -1.194108737 | 0.000282334 | 0.001439249 | LYRM4-AS1 |
| ENSG00000179859 | 30.72323118 | 1.491457644 | 0.000283467 | 0.00144413 | RNF227 |
| ENSG00000196611 | 62.30783248 | -1.024114608 | 0.00028398 | 0.001446445 | MMP1 |
| ENSG00000154319 | 9.250612234 | -2.784154292 | 0.000284203 | 0.001447281 | FAM167A |
| ENSG00000282608 | 84.71652067 | -2.351336875 | 0.000285292 | 0.001452523 | ADORA3 |
| ENSG00000133661 | 21.86123644 | -1.558954386 | 0.000292874 | 0.001488049 | SFTPD |
| ENSG00000219986 | 10.54305248 | 2.46339441 | 0.000294917 | 0.001496578 | BTF3P7 |
| ENSG00000163121 | 32.46741882 | 1.516967941 | 0.000295199 | 0.0014977 | NEURL3 |
| ENSG00000232977 | 11.86930703 | -2.287349281 | 0.000299747 | 0.001518275 | LINC00327 |
| ENSG00000196735 | 13.13660574 | 2.523650287 | 0.000300292 | 0.001520096 | HLA-DQA1 |
| ENSG00000125170 | 20.56953401 | -1.625162881 | 0.000301776 | 0.001526042 | DOK4 |
| ENSG00000137269 | 62.66539088 | -1.173644417 | 0.000305806 | 0.001544518 | LRRC1 |
| ENSG00000248445 | 29.03794878 | -1.459657452 | 0.000306869 | 0.001549571 | SEMA6A-AS1 |
| ENSG00000268941 | 7.775144443 | -3.410285532 | 0.000309525 | 0.0015617 | LINC01711 |
| ENSG00000204025 | 21.8143225 | -1.67865401 | 0.00031018 | 0.001564683 | TRPC5OS |
| ENSG00000147394 | 19.72843547 | 1.821539007 | 0.000313319 | 0.001579845 | ZNF185 |
| ENSG00000137857 | 35.28483506 | 1.298330397 | 0.000313378 | 0.001579845 | DUOX1 |
| ENSG00000137501 | 34.22678758 | -1.22379642 | 0.000314116 | 0.001582917 | SYTL2 |
| ENSG00000221866 | 10.14412016 | -2.921352415 | 0.000315522 | 0.001589678 | PLXNA4 |
| ENSG00000064989 | 38.62798928 | 4.666994654 | 0.000316014 | 0.001591507 | CALCRL |
| ENSG00000104765 | 1244.471026 | 1.075285881 | 0.000321024 | 0.001613111 | BNIP3L |
| ENSG00000259024 | 21.65658187 | 1.732682484 | 0.000322576 | 0.001619915 | TVP23C-CDRT4 |
| ENSG00000189212 | 46.91027349 | -1.073828977 | 0.00032291 | 0.001621261 | DPY19L2P1 |
| ENSG00000139985 | 17.30682715 | 1.713893656 | 0.000326729 | 0.001638765 | ADAM21 |
| ENSG00000231822 | 24.77702973 | 1.505229493 | 0.000332825 | 0.001667181 | SMC3P1 |
| ENSG00000166482 | 19.42191854 | -2.019690617 | 0.000332935 | 0.001667181 | MFAP4 |
| ENSG00000049283 | 5.540141537 | 4.951001025 | 0.000334096 | 0.001672652 | EPN3 |
| ENSG00000143355 | 6.964513055 | -3.677375082 | 0.000336725 | 0.001685131 | LHX9 |
| ENSG00000279873 | 18.37930925 | -1.906683051 | 0.000342276 | 0.001710478 | LINC01126 |
| ENSG00000101298 | 26.42381575 | -1.659629156 | 0.00034478 | 0.00172229 | SNPH |
| ENSG00000196110 | 125.9765769 | 1.025582626 | 0.000347181 | 0.001731828 | ZNF699 |
| ENSG00000172123 | 43.23407098 | -1.067762948 | 0.000350621 | 0.001746509 | SLFN12 |
| ENSG00000129480 | 43.6143834 | -1.139310619 | 0.000351383 | 0.001749599 | DTD2 |
| ENSG00000157680 | 33.38402825 | 1.223502091 | 0.000367074 | 0.001820002 | DGKI |
| ENSG00000172000 | 111.9613223 | 1.034614939 | 0.000367232 | 0.001820419 | ZNF556 |
| ENSG00000137474 | 8.784332477 | -2.948274765 | 0.000367807 | 0.0018229 | MYO7A |
| ENSG00000180279 | 12.54927287 | 2.302944903 | 0.000371587 | 0.001839048 | LINC01869 |
| ENSG00000109625 | 3.573345669 | -5.266027134 | 0.00037211 | 0.001840897 | CPZ |
| ENSG00000099834 | 17.80800194 | 1.922883028 | 0.000380987 | 0.001881407 | CDHR5 |
| ENSG00000135312 | 21.76106097 | -1.545492464 | 0.000389087 | 0.001917949 | HTR1B |
| ENSG00000171345 | 3.538121127 | -5.254750384 | 0.000389659 | 0.001920383 | KRT19 |
| ENSG00000175104 | 1222.887054 | 1.168463962 | 0.000392179 | 0.001932031 | TRAF6 |
| ENSG00000119782 | 27.76064213 | -1.41523274 | 0.000395669 | 0.001948054 | FKBP1B |
| ENSG00000077264 | 7.235969965 | -4.367137508 | 0.000396269 | 0.001950229 | PAK3 |
| ENSG00000084110 | 30.23769023 | -1.335163438 | 0.000396794 | 0.001952035 | HAL |
| ENSG00000130487 | 21.39858002 | -1.513860346 | 0.000403511 | 0.001983097 | KLHDC7B |
| ENSG00000107807 | 8.270713276 | -2.844996247 | 0.000404364 | 0.001986102 | TLX1 |
| ENSG00000255471 | 3.509838053 | 5.299347975 | 0.00040634 | 0.001995009 | PRSS23-AS1 |
| ENSG00000213225 | 13.37424008 | -2.096878842 | 0.000412931 | 0.002025754 | NOC2LP1 |
| ENSG00000271425 | 477.8247458 | 1.42118551 | 0.000414324 | 0.002031376 | NBPF10 |
| ENSG00000252516 | 34.63093122 | 1.234071693 | 0.000415047 | 0.002034112 | RNA5SP82 |
| ENSG00000176641 | 54.15753905 | 1.130516929 | 0.000415481 | 0.002035831 | RNF152 |
| ENSG00000108342 | 11.20544037 | 2.408844762 | 0.00041693 | 0.002040901 | CSF3 |
| ENSG00000230204 | 12.69136472 | 2.086182501 | 0.000424157 | 0.002074631 | FTH1P5 |
| ENSG00000128283 | 59.10198585 | 1.242548894 | 0.000434198 | 0.002118945 | CDC42EP1 |
| ENSG00000250585 | 43.67181433 | 1.099830309 | 0.000435583 | 0.002124191 | LINC00604 |
| ENSG00000280055 | 11.83922664 | -2.291399947 | 0.000436281 | 0.00212675 | LINC02912 |
| ENSG00000160460 | 19.02957714 | 2.470940304 | 0.00044303 | 0.002156662 | SPTBN4 |
| ENSG00000174502 | 8.611769756 | 4.677024905 | 0.000443593 | 0.002158975 | SLC26A9 |
| ENSG00000115194 | 55.5662713 | 1.497716058 | 0.000447663 | 0.002176634 | SLC30A3 |
| ENSG00000122507 | 33.28048324 | -1.417532391 | 0.000454642 | 0.002207954 | BBS9 |
| ENSG00000253651 | 5.168632745 | -4.791287716 | 0.000463925 | 0.002248864 | SOD1P3 |
| ENSG00000164330 | 33.59603853 | 1.197618955 | 0.000464683 | 0.002251832 | EBF1 |
| ENSG00000171124 | 7.508697503 | 3.054126021 | 0.00046582 | 0.002256455 | FUT3 |
| ENSG00000239911 | 11.35166807 | 2.276255388 | 0.000475661 | 0.002302316 | PRKAG2-AS1 |
| ENSG00000052126 | 22.0078963 | -1.570305441 | 0.000477635 | 0.002310962 | PLEKHA5 |
| ENSG00000213997 | 13.91287291 | -2.051976973 | 0.00047938 | 0.002318948 | PGAM1P7 |
| ENSG00000111276 | 533.7024256 | 1.258362649 | 0.000489775 | 0.002365517 | CDKN1B |
| ENSG00000144366 | 60.85337377 | 1.232960015 | 0.000491983 | 0.002375718 | GULP1 |
| ENSG00000122257 | 2065.471043 | 1.002966592 | 0.00049225 | 0.002376541 | RBBP6 |
| ENSG00000088899 | 21.06975578 | 1.56325426 | 0.000492425 | 0.00237692 | LZTS3 |
| ENSG00000176884 | 16.45531697 | -1.969011811 | 0.000494243 | 0.002383996 | GRIN1 |
| ENSG00000163734 | 222.8142566 | 2.417743673 | 0.000494252 | 0.002383996 | CXCL3 |
| ENSG00000163884 | 16.81743555 | 1.733877629 | 0.000494998 | 0.002386064 | KLF15 |
| ENSG00000235831 | 72.19650923 | -2.557146404 | 0.000497353 | 0.002396008 | BHLHE40-AS1 |
| ENSG00000250295 | 24.90253379 | 1.515603097 | 0.000503648 | 0.002423494 | RDH10-AS1 |
| ENSG00000169989 | 11.52458749 | 2.163335079 | 0.00050394 | 0.002424422 | TIGD4 |
| ENSG00000138795 | 48.9584061 | -1.069917698 | 0.000507952 | 0.002441819 | LEF1 |
| ENSG00000265190 | 9.558251987 | -2.617189408 | 0.000512013 | 0.002457998 | ANXA8 |
| ENSG00000136842 | 8.817559925 | -2.69667727 | 0.000529797 | 0.002536928 | TMOD1 |
| ENSG00000256340 | 18.57167065 | 1.846619357 | 0.000536431 | 0.002566201 | ABCC6P1 |
| ENSG00000210140 | 8.063706163 | 3.166779824 | 0.000543492 | 0.002596952 | MT-TC |
| ENSG00000196403 | 3.1983067 | 5.163051606 | 0.000546546 | 0.002610532 | OR10D1P |
| ENSG00000180257 | 48.89692126 | 1.064453244 | 0.000550571 | 0.002627721 | ZNF816 |
| ENSG00000249709 | 21.3436629 | 1.707770957 | 0.00055281 | 0.002637382 | ZNF564 |
| ENSG00000069399 | 505.1614482 | -1.480417473 | 0.00055328 | 0.002639114 | BCL3 |
| ENSG00000233828 | 5.026420382 | -4.748303946 | 0.000567317 | 0.00270241 | MIR4280HG |
| ENSG00000235374 | 30.42601569 | -1.276093917 | 0.000569088 | 0.002709796 | SSR4P1 |
| ENSG00000179583 | 36.21145541 | 1.376872694 | 0.000575148 | 0.002735377 | CIITA |
| ENSG00000215472 | 23.47992027 | 1.394678425 | 0.000575238 | 0.002735377 | RPL17-C18orf32 |
| ENSG00000177575 | 687.5419385 | 2.121318399 | 0.000583485 | 0.002771385 | CD163 |
| ENSG00000230457 | 31.52286883 | -1.231029802 | 0.000588304 | 0.002792656 | PA2G4P4 |
| ENSG00000113504 | 5.034561293 | -4.750158518 | 0.000600401 | 0.002845694 | SLC12A7 |
| ENSG00000237264 | 17.09513557 | 1.922401676 | 0.000600897 | 0.002846951 | FTH1P11 |
| ENSG00000011332 | 24.66964597 | -1.413672205 | 0.000607065 | 0.002874516 | DPF1 |
| ENSG00000141527 | 12.94936794 | 2.237297669 | 0.000610343 | 0.002887263 | CARD14 |
| ENSG00000232713 | 50.78350685 | 1.27542531 | 0.000616357 | 0.002912913 | RPS12P3 |
| ENSG00000262097 | 37.49324926 | -1.527385619 | 0.000616497 | 0.002913017 | LINC02185 |
| ENSG00000231233 | 26.92538574 | 1.384684713 | 0.000618419 | 0.002920419 | CFAP58-DT |
| ENSG00000232098 | 84.33942205 | 1.093369398 | 0.000618741 | 0.002921377 | ZNF584-DT |
| ENSG00000189283 | 38.2547308 | 1.290494674 | 0.000626043 | 0.002953023 | FHIT |
| ENSG00000198771 | 1255.298229 | -1.192785698 | 0.000636588 | 0.002998746 | RCSD1 |
| ENSG00000232075 | 6.485001598 | -3.56561058 | 0.000643501 | 0.003028457 | MRPL35P2 |
| ENSG00000180139 | 4.868401277 | -4.699407557 | 0.000648007 | 0.00304759 | ACTA2-AS1 |
| ENSG00000226983 | 7.320505198 | -2.941619376 | 0.000654154 | 0.003072093 | LINC01692 |
| ENSG00000125398 | 3.250501725 | -5.129232616 | 0.00066408 | 0.003113956 | SOX9 |
| ENSG00000223510 | 6.138388028 | -4.115738953 | 0.000665151 | 0.003118388 | CDRT15 |
| ENSG00000141542 | 95.89967903 | -1.024157907 | 0.000667975 | 0.003129243 | RAB40B |
| ENSG00000238105 | 34.76205983 | -1.230539062 | 0.000671723 | 0.003145008 | GOLGA2P5 |
| ENSG00000182580 | 53.12147441 | 1.25443223 | 0.000679016 | 0.003174927 | EPHB3 |
| ENSG00000137878 | 17.48986716 | 1.585758752 | 0.000687239 | 0.003208505 | GCOM1 |
| ENSG00000237310 | 32.06392271 | 1.271244781 | 0.000694809 | 0.003242005 | LINC03011 |
| ENSG00000177570 | 5.995493865 | -4.075941897 | 0.000696571 | 0.003249607 | SAMD12 |
| ENSG00000175161 | 4.709798251 | 4.708763979 | 0.000703047 | 0.003278581 | CADM2 |
| ENSG00000230397 | 15.76958739 | 1.867914665 | 0.0007042 | 0.003282094 | SPTLC1P1 |
| ENSG00000105255 | 3.218396746 | -5.117899574 | 0.000705362 | 0.003286265 | FSD1 |
| ENSG00000105613 | 104.4467531 | 1.15238622 | 0.000713181 | 0.003320178 | MAST1 |
| ENSG00000104738 | 1964.054118 | -1.057403784 | 0.00071846 | 0.003342859 | MCM4 |
| ENSG00000240970 | 26.41849892 | -1.322706703 | 0.00072833 | 0.003383031 | RPL23AP64 |
| ENSG00000274736 | 30.95651053 | -1.227231789 | 0.000731748 | 0.003396348 | CCL23 |
| ENSG00000253030 | 18.09357617 | 1.652538967 | 0.000743727 | 0.0034487 | MIR2116 |
| ENSG00000114019 | 13.11064978 | -2.1805626 | 0.000746342 | 0.003459521 | AMOTL2 |
| ENSG00000204789 | 3.810128546 | 5.419053257 | 0.000749795 | 0.003473566 | ZNF204P |
| ENSG00000227953 | 18.32474794 | -1.905688622 | 0.000751621 | 0.003480718 | LINC01341 |
| ENSG00000184792 | 39.39802915 | -1.11589894 | 0.000757835 | 0.003506857 | OSBP2 |
| ENSG00000159231 | 44.2502078 | -1.185978073 | 0.000761088 | 0.003517944 | CBR3 |
| ENSG00000249825 | 9.249144781 | -2.358458627 | 0.000765677 | 0.003536499 | THBS4-AS1 |
| ENSG00000096696 | 3.224102097 | -5.119913565 | 0.000782957 | 0.003609545 | DSP |
| ENSG00000196081 | 41.18364146 | 1.197158025 | 0.000789988 | 0.003639234 | ZNF724 |
| ENSG00000113721 | 18.09011414 | -1.577982624 | 0.000805259 | 0.003701968 | PDGFRB |
| ENSG00000169548 | 14.81452814 | -1.770644649 | 0.000806843 | 0.003708556 | ZNF280A |
| ENSG00000130766 | 308.9620124 | 1.31973576 | 0.000809041 | 0.003717965 | SESN2 |
| ENSG00000230091 | 19.87963382 | -1.835498997 | 0.000812077 | 0.003730228 | TMEM254-AS1 |
| ENSG00000273079 | 6.33620511 | 3.592049505 | 0.000816268 | 0.003746985 | GRIN2B |
| ENSG00000102174 | 3.083107514 | -5.053395569 | 0.000816668 | 0.00374812 | PHEX |
| ENSG00000250748 | 8.010842891 | -2.792842259 | 0.000834461 | 0.003822661 | MSRB3-AS1 |
| ENSG00000178803 | 20.19616245 | 1.794577507 | 0.000845767 | 0.003872293 | ADORA2A-AS1 |
| ENSG00000232729 | 19.47499981 | -1.795701653 | 0.000856889 | 0.003917388 | GTF2I-AS1 |
| ENSG00000121807 | 796.7731857 | -1.836301215 | 0.000857305 | 0.003918564 | CCR2 |
| ENSG00000238097 | 6.681433312 | -3.621560071 | 0.000864394 | 0.003947303 | LINC02037 |
| ENSG00000138678 | 835.2758448 | 1.987960474 | 0.000865949 | 0.003953673 | GPAT3 |
| ENSG00000129009 | 4.894859604 | -4.706820491 | 0.000870934 | 0.003973487 | ISLR |
| ENSG00000198064 | 39.86359794 | 1.029110063 | 0.000872455 | 0.003979687 | NPIPB13 |
| ENSG00000233621 | 43.71889669 | -1.068412423 | 0.000886221 | 0.004032773 | ZC3H12A-DT |
| ENSG00000179168 | 39.70938872 | 1.385564999 | 0.000887113 | 0.004035341 | GGN |
| ENSG00000254535 | 38.14601603 | -1.20049813 | 0.000890089 | 0.004047381 | PABPC4L |
| ENSG00000144362 | 15.06867848 | -1.794742752 | 0.000895895 | 0.004072278 | PHOSPHO2 |
| ENSG00000154175 | 18.59118506 | 1.70257784 | 0.000899995 | 0.004088655 | ABI3BP |
| ENSG00000138755 | 7.655952667 | -2.721315861 | 0.000906807 | 0.004112771 | CXCL9 |
| ENSG00000007171 | 15.35577482 | -1.747753889 | 0.000910251 | 0.004126112 | NOS2 |
| ENSG00000234409 | 23.80069368 | -1.394468691 | 0.000911928 | 0.004130677 | CCDC188 |
| ENSG00000133216 | 20.25325274 | -1.466241814 | 0.000913461 | 0.004135336 | EPHB2 |
| ENSG00000138080 | 31.88710634 | -1.148250291 | 0.000914738 | 0.00414036 | EMILIN1 |
| ENSG00000203709 | 20.02699557 | 1.585560123 | 0.000919044 | 0.004157556 | MIR29B2CHG |
| ENSG00000068831 | 45.39951538 | 1.062874484 | 0.000923638 | 0.004176806 | RASGRP2 |
| ENSG00000181773 | 56.49391552 | -1.514244846 | 0.00092398 | 0.004177583 | GPR3 |
| ENSG00000155974 | 39.79126252 | 1.111281762 | 0.000930477 | 0.00420233 | GRIP1 |
| ENSG00000257167 | 41.44900679 | -1.211346822 | 0.000932536 | 0.004210084 | TMPO-AS1 |
| ENSG00000198286 | 7.918272824 | -2.776004873 | 0.000940684 | 0.004243003 | CARD11 |
| ENSG00000179965 | 36.84006334 | -1.149090874 | 0.000946893 | 0.004268642 | ZNF771 |
| ENSG00000225724 | 4.527355111 | -4.593753524 | 0.000960564 | 0.004326315 | ASH2LP3 |
| ENSG00000130643 | 7.135272971 | 3.336331126 | 0.000967505 | 0.004355186 | CALY |
| ENSG00000121691 | 1191.512363 | 1.036274129 | 0.000971985 | 0.004372957 | CAT |
| ENSG00000271303 | 63.56021589 | 1.422766949 | 0.000977809 | 0.004394343 | SRXN1 |
| ENSG00000146038 | 30.51723218 | 1.328967716 | 0.000979901 | 0.00440294 | DCDC2 |
| ENSG00000164619 | 7.808094733 | -2.746532791 | 0.000982353 | 0.004411543 | BMPER |
| ENSG00000157782 | 8.613623322 | -2.439583234 | 0.00101093 | 0.004527495 | CABP1 |
| ENSG00000129596 | 34.52784193 | -1.080067767 | 0.001016369 | 0.004548547 | CDO1 |
| ENSG00000239732 | 14.29062322 | 1.865865347 | 0.001018729 | 0.004556626 | TLR9 |
| ENSG00000258227 | 10.28850794 | -2.184514489 | 0.00102598 | 0.004585727 | CLEC5A |
| ENSG00000104067 | 7.923338657 | -2.779307306 | 0.001031363 | 0.004605607 | TJP1 |
| ENSG00000167771 | 22.58612511 | -1.506451648 | 0.001031986 | 0.004607556 | RCOR2 |
| ENSG00000148950 | 38.16902288 | -1.143974072 | 0.001036898 | 0.004628647 | IMMP1L |
| ENSG00000118004 | 4.378931461 | 4.598624702 | 0.001047639 | 0.004670667 | COLEC11 |
| ENSG00000227258 | 28.01311196 | -1.299264727 | 0.001048537 | 0.004673825 | SMIM2-AS1 |
| ENSG00000204618 | 31.70060406 | 1.24763965 | 0.001049011 | 0.004675093 | RNF39 |
| ENSG00000166407 | 35.72057988 | 1.109347694 | 0.001049604 | 0.004676888 | LMO1 |
| ENSG00000154153 | 31.63379419 | -1.239053571 | 0.001066671 | 0.004746068 | RETREG1 |
| ENSG00000100628 | 11.56699922 | -1.965472696 | 0.001070013 | 0.004759552 | ASB2 |
| ENSG00000088992 | 28.84050745 | -1.198981329 | 0.00108165 | 0.004805773 | TESC |
| ENSG00000111057 | 2.911300847 | -4.971699803 | 0.001082062 | 0.004806735 | KRT18 |
| ENSG00000004848 | 35.40555571 | -1.160532643 | 0.001089074 | 0.004834397 | ARX |
| ENSG00000135736 | 4.974836703 | -4.736542165 | 0.001101734 | 0.004885327 | CCDC102A |
| ENSG00000170049 | 17.66555466 | -1.606645369 | 0.001109557 | 0.004914691 | KCNAB3 |
| ENSG00000087884 | 38.65653308 | -1.12799739 | 0.001119143 | 0.00495092 | AAMDC |
| ENSG00000158786 | 161.142557 | -2.061754869 | 0.001124648 | 0.004972596 | PLA2G2F |
| ENSG00000218459 | 10.62297175 | 2.652682211 | 0.001125916 | 0.00497731 | RPS27P15 |
| ENSG00000070087 | 12.71368917 | -1.902291015 | 0.001130554 | 0.004995124 | PFN2 |
| ENSG00000004799 | 189.3399611 | 2.418390133 | 0.001137176 | 0.005021679 | PDK4 |
| ENSG00000157765 | 3.269369333 | -5.136465369 | 0.001153406 | 0.005086055 | SLC34A2 |
| ENSG00000164344 | 4.376261339 | -4.540242385 | 0.001162241 | 0.00512318 | KLKB1 |
| ENSG00000147044 | 5.992918155 | -3.447531555 | 0.001170035 | 0.005153847 | CASK |
| ENSG00000163568 | 14.47407885 | -1.72896341 | 0.001171624 | 0.005158079 | AIM2 |
| ENSG00000174123 | 28.77602938 | 1.567701397 | 0.001180552 | 0.005193671 | TLR10 |
| ENSG00000168077 | 2.901867043 | -4.968032372 | 0.00118603 | 0.005215906 | SCARA3 |
| ENSG00000231249 | 11.98478119 | 1.979187253 | 0.00119789 | 0.005261486 | ITPR1-DT |
| ENSG00000132141 | 17.32237092 | -1.563444925 | 0.001200651 | 0.005271734 | CCT6B |
| ENSG00000102962 | 26.31443725 | -1.35420939 | 0.001205252 | 0.00529005 | CCL22 |
| ENSG00000141665 | 28.14721841 | -1.230498354 | 0.001214808 | 0.005329146 | FBXO15 |
| ENSG00000102349 | 25.59739428 | -1.50145505 | 0.001256238 | 0.005498161 | KLF8 |
| ENSG00000237080 | 10.69035563 | 2.178162047 | 0.001256503 | 0.005498344 | EHMT2-AS1 |
| ENSG00000055813 | 13.45699494 | 1.761610257 | 0.001263207 | 0.005524734 | CCDC85A |
| ENSG00000074410 | 7.842216688 | -2.758446649 | 0.001280307 | 0.005591583 | CA12 |
| ENSG00000251602 | 9.625352657 | -2.238071337 | 0.001286333 | 0.005614794 | MTA1-DT |
| ENSG00000111404 | 3.103876905 | -5.061519385 | 0.001286534 | 0.005614794 | RERGL |
| ENSG00000186205 | 53.67608745 | 1.168437187 | 0.001306733 | 0.005692864 | MTARC1 |
| ENSG00000215244 | 7.80769604 | 2.82900443 | 0.001316718 | 0.005731296 | LINC02649 |
| ENSG00000180758 | 26.68045079 | 1.457396171 | 0.001327857 | 0.005774677 | GPR157 |
| ENSG00000214534 | 9.153270313 | 2.226814234 | 0.001333682 | 0.005796939 | ZNF705EP |
| ENSG00000155754 | 13.34956071 | -1.87360227 | 0.001374649 | 0.005959231 | C2CD6 |
| ENSG00000185518 | 16.17594923 | -1.588126041 | 0.001408484 | 0.006094049 | SV2B |
| ENSG00000035720 | 10.79949053 | -2.120149695 | 0.001409448 | 0.006096139 | STAP1 |
| ENSG00000182747 | 15.2593469 | -1.735407452 | 0.001410081 | 0.006097393 | SLC35D3 |
| ENSG00000215483 | 8.834325246 | 2.340442258 | 0.001410233 | 0.006097393 | LINC00598 |
| ENSG00000224177 | 5.199946413 | 3.910835921 | 0.001418686 | 0.006130713 | LINC00570 |
| ENSG00000183850 | 47.48531787 | 1.058340276 | 0.001424613 | 0.006152012 | ZNF730 |
| ENSG00000105825 | 16.83676774 | -1.588111967 | 0.001426244 | 0.006156896 | TFPI2 |
| ENSG00000167642 | 4.21204539 | -4.483999169 | 0.00142945 | 0.006168571 | SPINT2 |
| ENSG00000168405 | 20.35108135 | -1.545437105 | 0.001437565 | 0.006194908 | CMAHP |
| ENSG00000226564 | 12.39751567 | 2.036131462 | 0.001462263 | 0.006292534 | FTH1P20 |
| ENSG00000110025 | 25.95772746 | 1.266745284 | 0.001470513 | 0.00632472 | SNX15 |
| ENSG00000013619 | 274.2438004 | 1.454712423 | 0.001472895 | 0.006333859 | MAMLD1 |
| ENSG00000104863 | 27.80097141 | 1.357606494 | 0.001534541 | 0.006573707 | LIN7B |
| ENSG00000253955 | 26.69688657 | -1.392908591 | 0.001541071 | 0.006598239 | LINC02995 |
| ENSG00000214433 | 5.700064712 | 3.422045864 | 0.001543139 | 0.006604796 | GOLGA2P8 |
| ENSG00000171680 | 11.08275523 | -2.032922073 | 0.001562952 | 0.00668263 | PLEKHG5 |
| ENSG00000204839 | 547.6482551 | 1.123472099 | 0.001566724 | 0.006695271 | MROH6 |
| ENSG00000251485 | 11.39836641 | -1.94248907 | 0.001569451 | 0.006704597 | NRBF2P6 |
| ENSG00000174885 | 18.65667282 | 1.576644945 | 0.001591852 | 0.006789698 | NLRP6 |
| ENSG00000176177 | 4.035565595 | 4.476584758 | 0.001593185 | 0.006793922 | ENTHD1 |
| ENSG00000130635 | 7.939370469 | -3.086542962 | 0.001593394 | 0.006793922 | COL5A1 |
| ENSG00000203872 | 27.52412659 | -1.183668424 | 0.001611401 | 0.006863572 | C6orf163 |
| ENSG00000126353 | 10.99155044 | 2.074730518 | 0.001626034 | 0.006922306 | CCR7 |
| ENSG00000130943 | 21.44674487 | 1.658749411 | 0.001627015 | 0.006925288 | PKDREJ |
| ENSG00000177427 | 43.89083525 | 1.01048011 | 0.001629366 | 0.006934097 | MIEF2 |
| ENSG00000184867 | 8.612536817 | 2.980132356 | 0.001646287 | 0.007001268 | ARMCX2 |
| ENSG00000233469 | 5.187987723 | 3.919259881 | 0.001660982 | 0.007056451 | ST6GALNAC4P1 |
| ENSG00000143994 | 10.32653982 | 1.969130321 | 0.001671903 | 0.007099173 | ABHD1 |
| ENSG00000221867 | 2.755242223 | -4.891607005 | 0.00167382 | 0.007104865 | MAGEA3 |
| ENSG00000270948 | 22.1709234 | 1.609832642 | 0.001686912 | 0.007159204 | MTDHP1 |
| ENSG00000241859 | 9.648034576 | -2.417798548 | 0.001695648 | 0.007191321 | ANOS2P |
| ENSG00000196632 | 6.993092842 | 2.63593881 | 0.001702776 | 0.007216582 | WNK3 |
| ENSG00000229647 | 29.51549863 | -1.203336978 | 0.001703497 | 0.007216952 | MYOSLID |
| ENSG00000124019 | 31.11681143 | -1.095474956 | 0.001718358 | 0.007271362 | FAM124B |
| ENSG00000141519 | 42.36552258 | -1.047171521 | 0.001738238 | 0.007345391 | CCDC40 |
| ENSG00000248202 | 4.04655076 | -4.424931894 | 0.001760862 | 0.007429523 | LINC02234 |
| ENSG00000173404 | 7.823541597 | 2.559066818 | 0.001784181 | 0.007517611 | INSM1 |
| ENSG00000131401 | 20.07090581 | 1.582723356 | 0.001863016 | 0.007812361 | NAPSB |
| ENSG00000239247 | 7.661098965 | 2.533370894 | 0.001869679 | 0.007836299 | RN7SL589P |
| ENSG00000223878 | 17.14269646 | 1.478609626 | 0.001911203 | 0.007995371 | PPIAP53 |
| ENSG00000135373 | 11.91555592 | -1.778655063 | 0.00192054 | 0.008028978 | EHF |
| ENSG00000186868 | 31.53252748 | 1.102478392 | 0.00192886 | 0.008058292 | MAPT |
| ENSG00000161649 | 7.180972995 | -2.923726376 | 0.001932074 | 0.008068979 | CD300LG |
| ENSG00000120903 | 24.10860456 | -1.535512965 | 0.001940803 | 0.008102205 | CHRNA2 |
| ENSG00000170955 | 4.181842195 | -4.475475126 | 0.002032928 | 0.008441518 | CAVIN3 |
| ENSG00000246695 | 35.10324948 | -1.3374707 | 0.002037351 | 0.008454183 | RASSF8-AS1 |
| ENSG00000142512 | 21.51031963 | -1.531803793 | 0.002051847 | 0.008504308 | SIGLEC10 |
| ENSG00000172403 | 62.3864575 | -1.188691446 | 0.002078793 | 0.008602962 | SYNPO2 |
| ENSG00000235385 | 19.30366247 | 1.447929873 | 0.002087781 | 0.008637255 | LINC02154 |
| ENSG00000260193 | 5.53031162 | -3.318672374 | 0.00210331 | 0.00869274 | LINC02846 |
| ENSG00000130720 | 9.810256545 | -2.102910737 | 0.00213392 | 0.008810377 | FIBCD1 |
| ENSG00000183775 | 4.024312725 | 4.474072234 | 0.002134518 | 0.008811368 | KCTD16 |
| ENSG00000197380 | 16.67524601 | 2.247157947 | 0.002138468 | 0.008824719 | DACT3 |
| ENSG00000165449 | 38.92641352 | 1.522548597 | 0.002147956 | 0.008859055 | SLC16A9 |
| ENSG00000204262 | 26.16086587 | -1.210990743 | 0.002148227 | 0.008859055 | COL5A2 |
| ENSG00000230778 | 49.60508517 | -1.066027937 | 0.002155185 | 0.008880316 | ANKRD63 |
| ENSG00000138083 | 32.39864504 | 1.476225108 | 0.002175338 | 0.00894857 | SIX3 |
| ENSG00000233013 | 34.44996604 | 1.096933806 | 0.002178209 | 0.008958699 | FAM157B |
| ENSG00000205683 | 592.0289336 | -1.110591443 | 0.00218952 | 0.009003714 | DPF3 |
| ENSG00000136541 | 43.53534384 | 1.114771259 | 0.002190718 | 0.009007137 | ERMN |
| ENSG00000171097 | 17.81905341 | 1.479915925 | 0.002195987 | 0.009027295 | KYAT1 |
| ENSG00000157554 | 10.44570636 | -1.918650873 | 0.002211121 | 0.009084507 | ERG |
| ENSG00000224747 | 2.824975629 | 4.987393403 | 0.002211748 | 0.009084507 | MTCYBP21 |
| ENSG00000173269 | 23.81356841 | 1.791688224 | 0.00221423 | 0.009091669 | MMRN2 |
| ENSG00000005187 | 15.11157568 | 1.618828459 | 0.002217003 | 0.009101275 | ACSM3 |
| ENSG00000117228 | 37.13549011 | 1.018204527 | 0.002217309 | 0.009101275 | GBP1 |
| ENSG00000139200 | 5.236778253 | 3.918590354 | 0.00222352 | 0.009123729 | PIANP |
| ENSG00000274286 | 12.10131306 | -2.045333959 | 0.002238493 | 0.009175995 | ADRA2B |
| ENSG00000229444 | 21.92414593 | -1.280435797 | 0.002248903 | 0.009212535 | ST3GAL3-AS1 |
| ENSG00000226200 | 30.24724706 | -1.378413421 | 0.002252039 | 0.009223434 | SGMS1-AS1 |
| ENSG00000164116 | 2.59286936 | -4.804002774 | 0.002266801 | 0.009279681 | GUCY1A1 |
| ENSG00000182508 | 5.016235335 | -3.803141079 | 0.002267455 | 0.009280817 | LHFPL1 |
| ENSG00000129450 | 14.19456595 | -1.596739487 | 0.002268672 | 0.009284254 | SIGLEC9 |
| ENSG00000142871 | 10.74852361 | -1.973151239 | 0.002272685 | 0.009297587 | CCN1 |
| ENSG00000244219 | 7.488798684 | 2.493586605 | 0.00228043 | 0.009324627 | TMEM225B |
| ENSG00000273003 | 4.149760287 | 4.524431317 | 0.002284093 | 0.009336504 | ARL2-SNX15 |
| ENSG00000256612 | 31.65752142 | 1.109586806 | 0.00229342 | 0.009373075 | CYP2B7P |
| ENSG00000255274 | 28.65674041 | 1.344104558 | 0.002296605 | 0.009382844 | SMIM35 |
| ENSG00000232368 | 5.354149942 | 3.329988728 | 0.002303252 | 0.009403896 | FTLP2 |
| ENSG00000181722 | 33.95698483 | 1.284182114 | 0.00230633 | 0.009414901 | ZBTB20 |
| ENSG00000108001 | 21.43498885 | -1.404340236 | 0.00231413 | 0.009445176 | EBF3 |
| ENSG00000175591 | 556.7380641 | -1.097128598 | 0.002338546 | 0.009538427 | P2RY2 |
| ENSG00000267871 | 10.46078202 | 1.994191235 | 0.002342638 | 0.009550456 | ZNF460-AS1 |
| ENSG00000164197 | 11.89685874 | -1.897372638 | 0.002356352 | 0.009603189 | RNF180 |
| ENSG00000164778 | 32.34462262 | -1.208316801 | 0.002370019 | 0.009654095 | EN2 |
| ENSG00000132470 | 16.99839282 | -1.460228535 | 0.002370565 | 0.00965472 | ITGB4 |
| ENSG00000179165 | 8.646410379 | 2.303945281 | 0.002387706 | 0.009718106 | PXT1 |
| ENSG00000274840 | 14.62429327 | -1.741062193 | 0.002415818 | 0.009821163 | BALR6 |
| ENSG00000100314 | 12.27124486 | 2.279317115 | 0.002422383 | 0.009842978 | CABP7 |
| ENSG00000174099 | 6.274097379 | -3.064271946 | 0.002432524 | 0.009879294 | MSRB3 |
| ENSG00000135119 | 39.41966967 | 1.00447833 | 0.0024378 | 0.009899092 | RNFT2 |
| ENSG00000231494 | 5.545988957 | -3.316593907 | 0.002440424 | 0.009908112 | RPL21P35 |
| ENSG00000277893 | 11.89231467 | -1.894320645 | 0.002444208 | 0.00992184 | SRD5A2 |
| ENSG00000164362 | 3.89802636 | -4.366561152 | 0.002445898 | 0.009925432 | TERT |
| ENSG00000182057 | 33.52390035 | -1.14166418 | 0.002462083 | 0.009987816 | OGFRP1 |
| ENSG00000288568 | 14.16493293 | 1.671274517 | 0.002467636 | 0.010008697 | ZCCHC14-DT |
| ENSG00000175643 | 30.99380603 | -1.116245457 | 0.002477686 | 0.010042844 | RMI2 |
| ENSG00000231752 | 31.8723589 | -1.142237575 | 0.00250249 | 0.010136708 | EMBP1 |
| ENSG00000278175 | 11.12520657 | -1.893665772 | 0.002508898 | 0.010155987 | GLIDR |
| ENSG00000273129 | 4.329017206 | -4.526735439 | 0.002543052 | 0.010282411 | PACERR |
| ENSG00000169071 | 6.991689161 | 2.648087927 | 0.002548199 | 0.010296465 | ROR2 |
| ENSG00000162545 | 44.27831494 | 1.076660429 | 0.002549835 | 0.010301384 | CAMK2N1 |
| ENSG00000117525 | 23.85775823 | -1.20297917 | 0.00255238 | 0.010309974 | F3 |
| ENSG00000158023 | 32.61458016 | -1.160811015 | 0.002553027 | 0.010310895 | CFAP251 |
| ENSG00000237732 | 21.1786201 | 1.398088512 | 0.002562548 | 0.010344262 | CT75 |
| ENSG00000198435 | 183.0853577 | -1.754455841 | 0.002563681 | 0.010347137 | NRARP |
| ENSG00000140285 | 5.057165228 | -3.813055204 | 0.002564548 | 0.010348942 | FGF7 |
| ENSG00000155749 | 15.60248201 | -1.695663408 | 0.00256971 | 0.010366376 | FLACC1 |
| ENSG00000225808 | 19.41484921 | 1.581150414 | 0.002623736 | 0.010570472 | DNAJC19P5 |
| ENSG00000161381 | 16.30477251 | 1.600484216 | 0.002625858 | 0.010574101 | PLXDC1 |
| ENSG00000136895 | 32.85092022 | 1.115674747 | 0.002631587 | 0.010593437 | GARNL3 |
| ENSG00000266983 | 48.35776516 | 1.16859662 | 0.00264148 | 0.010628051 | RANBP3-DT |
| ENSG00000137941 | 15.21179392 | -1.548241244 | 0.002645043 | 0.010637174 | TTLL7 |
| ENSG00000079156 | 16.33956417 | -1.528765754 | 0.002647609 | 0.010644726 | OSBPL6 |
| ENSG00000102996 | 7.326668544 | -2.387128513 | 0.00264924 | 0.010648836 | MMP15 |
| ENSG00000154721 | 18.52157037 | -1.415473925 | 0.002651395 | 0.010655761 | JAM2 |
| ENSG00000237803 | 7.072640671 | -2.901718069 | 0.00265647 | 0.010674417 | LINC00211 |
| ENSG00000176029 | 10.43369338 | -1.913604042 | 0.002665423 | 0.010705351 | C11orf16 |
| ENSG00000160606 | 5.379085342 | 3.337628256 | 0.002665472 | 0.010705351 | TLCD1 |
| ENSG00000165091 | 30.15972067 | -1.281627591 | 0.002671363 | 0.01072551 | TMC1 |
| ENSG00000233654 | 7.761518188 | -2.73985771 | 0.002682144 | 0.010761618 | NEMP2-DT |
| ENSG00000122786 | 8.783802168 | -2.701904546 | 0.002702166 | 0.010835057 | CALD1 |
| ENSG00000133116 | 27.65841998 | -1.443883986 | 0.00270955 | 0.01086113 | KL |
| ENSG00000169155 | 2471.83022 | 1.351643499 | 0.002712304 | 0.010868629 | ZBTB43 |
| ENSG00000130055 | 20.12350342 | -1.448866584 | 0.002729811 | 0.01093167 | GDPD2 |
| ENSG00000250116 | 54.99184224 | 1.135170449 | 0.002733173 | 0.010943355 | RHOQ-AS1 |
| ENSG00000140403 | 5504.998009 | 1.050003786 | 0.002760065 | 0.011040261 | DNAJA4 |
| ENSG00000168269 | 45.64271597 | -3.387973057 | 0.002770076 | 0.011071314 | FOXI1 |
| ENSG00000259172 | 17.29715219 | 1.422155985 | 0.002795187 | 0.011164432 | SNRPA1-DT |
| ENSG00000182492 | 3.728728163 | -4.301133383 | 0.002805482 | 0.011197568 | BGN |
| ENSG00000145685 | 786.4786936 | 1.126303479 | 0.002828613 | 0.011279648 | LHFPL2 |
| ENSG00000139725 | 36.23253887 | 1.026001007 | 0.002854245 | 0.011372656 | RHOF |
| ENSG00000272767 | 29.47637174 | -1.198987722 | 0.002874754 | 0.011446968 | JMJD1C-AS1 |
| ENSG00000175785 | 2.668901528 | 4.904481404 | 0.002877168 | 0.011454726 | PRIMA1 |
| ENSG00000183379 | 2.526554443 | 4.822768605 | 0.002881667 | 0.011468932 | SYNDIG1L |
| ENSG00000118322 | 7.677834721 | -2.459933509 | 0.002895638 | 0.011520813 | ATP10B |
| ENSG00000236668 | 9.467195604 | -2.037206553 | 0.002924894 | 0.0116297 | LHX2-AS1 |
| ENSG00000130675 | 35.89005229 | -1.166029924 | 0.002934012 | 0.011660766 | MNX1 |
| ENSG00000111729 | 5.329206083 | 3.319482733 | 0.002956775 | 0.011741301 | CLEC4A |
| ENSG00000270177 | 5.206179189 | -3.216968865 | 0.002968295 | 0.011783245 | PPP2CA-DT |
| ENSG00000238160 | 43.21675131 | -1.01298419 | 0.002970649 | 0.011786893 | LINC02863 |
| ENSG00000187686 | 2.567687511 | -4.79271143 | 0.002972726 | 0.011793233 | KRT18P59 |
| ENSG00000260034 | 14.48863568 | 1.617033937 | 0.00297903 | 0.011814438 | LCMT1-AS2 |
| ENSG00000145721 | 4.704061593 | -3.69595952 | 0.002997065 | 0.011878312 | LIX1 |
| ENSG00000179403 | 2.601694273 | -4.80806845 | 0.003000296 | 0.01188499 | VWA1 |
| ENSG00000033030 | 816.1234334 | 1.032273326 | 0.003026318 | 0.011982681 | ZCCHC8 |
| ENSG00000277610 | 19.68441817 | -1.531860332 | 0.003059546 | 0.012106463 | RNVU1-4 |
| ENSG00000169981 | 36.89013578 | 1.089924309 | 0.003063525 | 0.012118316 | ZNF35 |
| ENSG00000162771 | 2.501586567 | 4.811347432 | 0.003070819 | 0.012145218 | GARIN4 |
| ENSG00000116157 | 18.1020785 | -1.509774412 | 0.003082423 | 0.012187198 | GPX7 |
| ENSG00000255769 | 13.29037158 | 1.735248722 | 0.003094265 | 0.012230093 | GOLGA2P10 |
| ENSG00000167183 | 32.66732319 | 1.244985975 | 0.003129288 | 0.012352663 | PRR15L |
| ENSG00000198019 | 270.4598729 | -1.772280814 | 0.003134907 | 0.012366918 | FCGR1BP |
| ENSG00000227038 | 8.297978681 | 2.238209707 | 0.003142869 | 0.01239634 | GTF2IP7 |
| ENSG00000121900 | 23.30624756 | -1.259901899 | 0.003150149 | 0.012421078 | TMEM54 |
| ENSG00000154358 | 354.6724777 | -1.14670201 | 0.003181287 | 0.012531819 | OBSCN |
| ENSG00000250444 | 12.00491869 | 1.747887605 | 0.003210267 | 0.012633853 | CCT5P1 |
| ENSG00000171346 | 4.903979406 | 3.822109342 | 0.003233808 | 0.012708227 | KRT15 |
| ENSG00000235354 | 5.021969707 | 3.86526672 | 0.003241511 | 0.012728345 | RPS29P16 |
| ENSG00000131724 | 2513.745627 | 1.021308778 | 0.003252253 | 0.012766452 | IL13RA1 |
| ENSG00000224892 | 21.01031172 | 1.321146237 | 0.003291692 | 0.012896605 | RPS4XP16 |
| ENSG00000169247 | 31.28579572 | 1.119402207 | 0.003300506 | 0.012927024 | SH3TC2 |
| ENSG00000176716 | 2.427985822 | -4.70957744 | 0.003302596 | 0.012933155 | OR10AB1P |
| ENSG00000166086 | 17.57622502 | -1.530155348 | 0.003419252 | 0.013343318 | JAM3 |
| ENSG00000114013 | 23.14519053 | -1.391026267 | 0.003438311 | 0.013411319 | CD86 |
| ENSG00000230701 | 13.76522327 | -1.841915843 | 0.003460854 | 0.01348217 | FBXW4P1 |
| ENSG00000231638 | 2.74519953 | -4.887368632 | 0.003466567 | 0.013500153 | LUARIS |
| ENSG00000214274 | 2.357993277 | 4.723144065 | 0.003525011 | 0.013708253 | ANG |
| ENSG00000186310 | 5.162795118 | -3.213118805 | 0.00353265 | 0.013729288 | NAP1L3 |
| ENSG00000251791 | 13.21315941 | -1.656710586 | 0.003542482 | 0.013765329 | SCARNA6 |
| ENSG00000102904 | 5.049509473 | -3.170385412 | 0.003543467 | 0.013766986 | TSNAXIP1 |
| ENSG00000278558 | 6.052270288 | 3.049613937 | 0.003554028 | 0.013805838 | TMEM191B |
| ENSG00000175279 | 19.58541257 | -1.281106845 | 0.003590354 | 0.013929376 | CENPS |
| ENSG00000163288 | 3.566355299 | -4.233903948 | 0.003617331 | 0.014027515 | GABRB1 |
| ENSG00000197872 | 219.2207669 | -1.361089949 | 0.003617358 | 0.014027515 | CYRIA |
| ENSG00000154269 | 3.903654394 | -4.369459717 | 0.003623411 | 0.014048772 | ENPP3 |
| ENSG00000274963 | 14.92986054 | 1.768003377 | 0.003691582 | 0.014292842 | RN7SL600P |
| ENSG00000256582 | 12.72612227 | -1.686948729 | 0.003713141 | 0.014369534 | LINC02390 |
| ENSG00000182798 | 6.849692717 | -2.527496303 | 0.003761421 | 0.014528985 | MAGEB17 |
| ENSG00000088881 | 5.206745796 | -3.209876389 | 0.003762164 | 0.014529573 | EBF4 |
| ENSG00000070182 | 5.529578996 | -2.851781385 | 0.003768492 | 0.01454945 | SPTB |
| ENSG00000140511 | 34.65162163 | -1.229802537 | 0.003795495 | 0.014646816 | HAPLN3 |
| ENSG00000261645 | 4.659885252 | 3.748467864 | 0.003798498 | 0.014653814 | DISC1FP1 |
| ENSG00000251880 | 10.41549336 | -1.912594281 | 0.003821209 | 0.014732199 | RNU7-75P |
| ENSG00000106178 | 5.997080939 | -2.61815643 | 0.003909792 | 0.015050164 | CCL24 |
| ENSG00000235776 | 19.24687904 | -1.424891558 | 0.003911502 | 0.015054394 | RPL7AP70 |
| ENSG00000273340 | 15.13095213 | 1.538748373 | 0.003927321 | 0.0151003 | MICE |
| ENSG00000228653 | 22.30656683 | 1.455716049 | 0.003950098 | 0.015176854 | HNRNPCP7 |
| ENSG00000213055 | 2.414139562 | -4.702965436 | 0.003972195 | 0.015254616 | EEF1B2P7 |
| ENSG00000284240 | 7.661902041 | -2.225098945 | 0.003977407 | 0.01526987 | LINC02801 |
| ENSG00000257488 | 6.676012337 | -2.48996778 | 0.003992539 | 0.015316027 | LINC02354 |
| ENSG00000151364 | 3.575789103 | -4.237044696 | 0.004038941 | 0.015474754 | KCTD14 |
| ENSG00000198046 | 6.509936315 | 2.807335211 | 0.004042987 | 0.015487847 | ZNF667 |
| ENSG00000112706 | 31.2476652 | 1.14260641 | 0.004089726 | 0.015647426 | IMPG1 |
| ENSG00000110200 | 61.94919386 | -1.024835963 | 0.004090994 | 0.015649847 | ANAPC15 |
| ENSG00000253328 | 19.11691261 | 1.31139662 | 0.004091772 | 0.015650393 | SUMO2P19 |
| ENSG00000186086 | 36.26638849 | -1.029784546 | 0.004126336 | 0.015772799 | NBPF6 |
| ENSG00000074660 | 18.2292098 | 1.462929549 | 0.004172612 | 0.015917575 | SCARF1 |
| ENSG00000244218 | 11.24865477 | 2.124852679 | 0.004250773 | 0.016175661 | RN7SL81P |
| ENSG00000148677 | 13.66579414 | 1.503365901 | 0.004268577 | 0.016235884 | ANKRD1 |
| ENSG00000143248 | 23.68391691 | -1.233046202 | 0.004283265 | 0.016283894 | RGS5 |
| ENSG00000109943 | 10.62823399 | 2.013728105 | 0.004284462 | 0.016283894 | CRTAM |
| ENSG00000092621 | 706.8969474 | 1.020782884 | 0.004284504 | 0.016283894 | PHGDH |
| ENSG00000198945 | 840.4256071 | -1.04107376 | 0.004302328 | 0.016336511 | L3MBTL3 |
| ENSG00000164023 | 20.32792011 | -1.348351684 | 0.004320023 | 0.016391686 | SGMS2 |
| ENSG00000185261 | 12.89071259 | -1.600889091 | 0.004332577 | 0.016431108 | KIAA0825 |
| ENSG00000122824 | 10.96651353 | -1.738154937 | 0.004362449 | 0.016534211 | NUDT10 |
| ENSG00000257242 | 12.80364366 | -1.921599929 | 0.004430071 | 0.016754416 | LINC01619 |
| ENSG00000166813 | 11.26608088 | -1.672273785 | 0.004462647 | 0.016862083 | KIF7 |
| ENSG00000201207 | 4.569393968 | 3.706686049 | 0.00449675 | 0.016964917 | Y_RNA |
| ENSG00000183734 | 2.270634306 | -4.612288499 | 0.004555777 | 0.017153453 | ASCL2 |
| ENSG00000130147 | 3.558825482 | -4.230437929 | 0.00458496 | 0.017247516 | SH3BP4 |
| ENSG00000164123 | 7.126723951 | 2.666867369 | 0.004606944 | 0.017311709 | C4orf45 |
| ENSG00000242829 | 4.882113059 | -3.119645594 | 0.00462719 | 0.017374535 | RPS26P21 |
| ENSG00000074181 | 15.88432237 | -1.466328333 | 0.004631308 | 0.017384698 | NOTCH3 |
| ENSG00000153902 | 25.73354389 | 1.208063524 | 0.004662203 | 0.017490006 | LGI4 |
| ENSG00000166689 | 22.12531998 | -1.243027352 | 0.004724816 | 0.017698366 | PLEKHA7 |
| ENSG00000188452 | 18.96807786 | -1.334508851 | 0.004769473 | 0.017840799 | CERKL |
| ENSG00000143479 | 327.7218792 | 1.658877921 | 0.004788781 | 0.017899431 | DYRK3 |
| ENSG00000236570 | 5.357772328 | -2.800625833 | 0.004805826 | 0.017957694 | RAD23BP1 |
| ENSG00000230330 | 31.37432702 | -1.217842522 | 0.004834393 | 0.01804801 | HMGN2P3 |
| ENSG00000237886 | 20.83675815 | 1.603449419 | 0.004846888 | 0.018089175 | NALT1 |
| ENSG00000257135 | 32.42017932 | 1.184666687 | 0.004885834 | 0.018220722 | ODC1-DT |
| ENSG00000185015 | 11.46003762 | -1.946705994 | 0.004912764 | 0.018315606 | CA13 |
| ENSG00000242866 | 24.06953459 | -1.48010648 | 0.004936414 | 0.018380764 | STRC |
| ENSG00000156886 | 9.290641559 | 1.932079406 | 0.004939712 | 0.018388239 | ITGAD |
| ENSG00000187045 | 4.875798818 | -3.11817076 | 0.004954786 | 0.018441566 | TMPRSS6 |
| ENSG00000117215 | 2.488476398 | 4.804650364 | 0.004976569 | 0.018517045 | PLA2G2D |
| ENSG00000217325 | 10.94070638 | -1.736291685 | 0.004983792 | 0.01853832 | PRELID1P1 |
| ENSG00000121318 | 5.538274504 | -2.845084785 | 0.004997282 | 0.018577279 | TAS2R10 |
| ENSG00000213013 | 11.95998696 | 1.636977434 | 0.005000292 | 0.018582859 | RPS15AP36 |
| ENSG00000183638 | 11.21218916 | -1.919508655 | 0.005054345 | 0.018755442 | RP1L1 |
| ENSG00000104728 | 23.99054061 | 1.208475477 | 0.005075988 | 0.018830082 | ARHGEF10 |
| ENSG00000211699 | 14.83751523 | -1.425593363 | 0.005164586 | 0.019109821 | TRGV3 |
| ENSG00000136160 | 251.5332391 | 2.15942713 | 0.005187681 | 0.019184268 | EDNRB |
| ENSG00000119630 | 44.99729822 | 1.038147815 | 0.005187821 | 0.019184268 | PGF |
| ENSG00000251323 | 3.392646848 | -4.159477047 | 0.005217287 | 0.019278759 | LINC02728 |
| ENSG00000187955 | 25.05395111 | -1.122007227 | 0.005236807 | 0.019336387 | COL14A1 |
| ENSG00000231952 | 5.024212431 | -3.153548527 | 0.005251351 | 0.019384276 | DPY19L1P2 |
| ENSG00000204104 | 181.9338877 | -1.707153047 | 0.005268428 | 0.019438574 | TRAF3IP1 |
| ENSG00000172893 | 241.6991217 | 1.317877821 | 0.005274521 | 0.019458141 | DHCR7 |
| ENSG00000285532 | 2.17569884 | 4.609038391 | 0.005284908 | 0.01949354 | LINC02788 |
| ENSG00000125144 | 3.824989286 | -4.343960269 | 0.005318088 | 0.019604184 | MT1G |
| ENSG00000124134 | 18.21252899 | -1.603816709 | 0.005421962 | 0.019927449 | KCNS1 |
| ENSG00000131620 | 2.267514742 | -4.610786099 | 0.005443432 | 0.019988573 | ANO1 |
| ENSG00000011105 | 14.71585828 | -1.401344222 | 0.005469052 | 0.020073564 | TSPAN9 |
| ENSG00000054654 | 4.857601002 | -3.114291246 | 0.005480987 | 0.020114374 | SYNE2 |
| ENSG00000156206 | 4.216943002 | -3.525184217 | 0.005485076 | 0.020125755 | CFAP161 |
| ENSG00000185271 | 14.65270039 | -1.573281035 | 0.005538757 | 0.020299161 | KLHL33 |
| ENSG00000227908 | 7.32207806 | -2.146616023 | 0.005597043 | 0.020484694 | IL6ST-DT |
| ENSG00000147724 | 5.873665477 | -2.57553702 | 0.005597179 | 0.020484694 | FAM135B |
| ENSG00000228519 | 6.041626294 | -2.616627398 | 0.005662245 | 0.020684032 | RANBP1P1 |
| ENSG00000233791 | 6.674860858 | -2.498997171 | 0.005670608 | 0.020708437 | BTG2-DT |
| ENSG00000164512 | 3.227154421 | -4.08408172 | 0.005718928 | 0.020863246 | ANKRD55 |
| ENSG00000111728 | 6.160313643 | 2.417803697 | 0.005721922 | 0.020871077 | ST8SIA1 |
| ENSG00000106714 | 13.59901231 | -1.594655181 | 0.005732245 | 0.020893263 | CNTNAP3 |
| ENSG00000167889 | 31.1215379 | -1.067209361 | 0.005744117 | 0.020927243 | MGAT5B |
| ENSG00000133985 | 7.668327069 | -2.235819871 | 0.005756854 | 0.020970546 | TTC9 |
| ENSG00000198513 | 21.59809243 | -1.145905319 | 0.005799357 | 0.021112883 | ATL1 |
| ENSG00000027869 | 7.022649925 | -2.308332544 | 0.005862173 | 0.021300641 | SH2D2A |
| ENSG00000162733 | 29.41890115 | -1.095064993 | 0.005982281 | 0.021682689 | DDR2 |
| ENSG00000102755 | 17.30559263 | -1.411953658 | 0.005991681 | 0.021710368 | FLT1 |
| ENSG00000156140 | 90.57257207 | -1.832823044 | 0.005994217 | 0.021716362 | ADAMTS3 |
| ENSG00000152952 | 24.21348626 | -1.517852269 | 0.006004731 | 0.021751254 | PLOD2 |
| ENSG00000233143 | 3.204617472 | 4.126817708 | 0.006026903 | 0.02181553 | DIRC3-AS1 |
| ENSG00000066248 | 24.47009466 | -1.361541888 | 0.006046397 | 0.021876449 | NGEF |
| ENSG00000257838 | 21.21743903 | 1.243146234 | 0.006064656 | 0.021932844 | OTOAP1 |
| ENSG00000258479 | 3.489928489 | 4.260768669 | 0.006070149 | 0.021949488 | LINC00640 |
| ENSG00000254554 | 6.680396735 | 2.291442929 | 0.006071714 | 0.021951926 | ADM-DT |
| ENSG00000260233 | 8.170466104 | 2.220277176 | 0.006219188 | 0.022416015 | ZNRD2-DT |
| ENSG00000264743 | 25.95056255 | 1.146944986 | 0.006220786 | 0.022418493 | DPRXP4 |
| ENSG00000134363 | 5.336986521 | -2.798943504 | 0.006224781 | 0.022429608 | FST |
| ENSG00000088387 | 14.68398149 | -1.404531662 | 0.006285631 | 0.02260588 | DOCK9 |
| ENSG00000151917 | 21.92323814 | -1.274172879 | 0.006310956 | 0.022688345 | BEND6 |
| ENSG00000189157 | 3.602872734 | -4.246834953 | 0.006325822 | 0.022733825 | FAM47E |
| ENSG00000234118 | 22.49835348 | 1.262993431 | 0.006353564 | 0.022804045 | RPL13AP6 |
| ENSG00000105696 | 3.171530501 | 4.114675345 | 0.006408977 | 0.022965655 | TMEM59L |
| ENSG00000204287 | 26.32997277 | -1.218897732 | 0.006438582 | 0.023058325 | HLA-DRA |
| ENSG00000148848 | 4.705823227 | -3.055445724 | 0.0064703 | 0.023144993 | ADAM12 |
| ENSG00000235863 | 25.8591821 | 1.052091511 | 0.006491727 | 0.023211527 | B3GALT4 |
| ENSG00000281491 | 2.103240095 | -4.5025664 | 0.006517947 | 0.023268126 | DNAJB5-DT |
| ENSG00000149609 | 10.68507487 | 1.64234896 | 0.006534142 | 0.023315802 | C20orf144 |
| ENSG00000170629 | 17.66880679 | -1.32146178 | 0.006611404 | 0.023564187 | DPY19L2P2 |
| ENSG00000030419 | 5.693828686 | -2.537191448 | 0.006619543 | 0.023586371 | IKZF2 |
| ENSG00000259706 | 8.515769747 | -2.01598018 | 0.006634202 | 0.023631767 | HSP90B2P |
| ENSG00000179938 | 44.5285777 | 1.192180183 | 0.006640273 | 0.023649972 | GOLGA8J |
| ENSG00000205572 | 7.983551869 | -2.305133851 | 0.006657091 | 0.023694074 | SERF1B |
| ENSG00000280039 | 15.28604019 | 1.550242366 | 0.006665035 | 0.023707311 | RN7SKP23 |
| ENSG00000121454 | 25.36103225 | -1.136331837 | 0.006748565 | 0.023980187 | LHX4 |
| ENSG00000258609 | 3.21330816 | -4.078906634 | 0.006773897 | 0.024045919 | LINC-ROR |
| ENSG00000205097 | 4.909140195 | -3.119838724 | 0.006793233 | 0.024111083 | FRG2 |
| ENSG00000257599 | 27.64982133 | 1.076312301 | 0.006828337 | 0.024225205 | OVCH1-AS1 |
| ENSG00000228903 | 32.51935064 | -1.106000525 | 0.006858954 | 0.024316313 | RASA4CP |
| ENSG00000258057 | 10.8937061 | 1.79148198 | 0.006891875 | 0.024415456 | BCDIN3D-AS1 |
| ENSG00000172818 | 4.160483516 | 3.566142309 | 0.006897034 | 0.024426706 | OVOL1 |
| ENSG00000198597 | 17.70023598 | -1.522298095 | 0.00691191 | 0.024469412 | ZNF536 |
| ENSG00000242498 | 10.72743037 | -1.826821938 | 0.00692603 | 0.024508256 | ARPIN |
| ENSG00000106336 | 20.22112952 | 1.318232448 | 0.006944458 | 0.024562878 | FBXO24 |
| ENSG00000168671 | 5.669866821 | -2.529563943 | 0.007017349 | 0.024792219 | UGT3A2 |
| ENSG00000198910 | 17.19524686 | -1.341998112 | 0.007103647 | 0.025074884 | L1CAM |
| ENSG00000112964 | 10.27820042 | -1.752227688 | 0.007133574 | 0.025163137 | GHR |
| ENSG00000215866 | 5.530108637 | -2.480195591 | 0.007138505 | 0.025176926 | LINC01356 |
| ENSG00000266017 | 20.77929839 | -1.167080861 | 0.007145675 | 0.025194999 | MIR4477B |
| ENSG00000018408 | 17.01275451 | -1.390400927 | 0.007182895 | 0.025310919 | WWTR1 |
| ENSG00000118523 | 4.047038118 | -3.461169145 | 0.007196666 | 0.025349387 | CCN2 |
| ENSG00000144712 | 19.42566904 | -1.208910922 | 0.007248929 | 0.025511591 | CAND2 |
| ENSG00000227256 | 10.79402853 | -1.58874307 | 0.007262768 | 0.025556645 | MIS18A-AS1 |
| ENSG00000237575 | 13.42310189 | -1.581636392 | 0.007274276 | 0.025589825 | PYY2 |
| ENSG00000255303 | 4.565512648 | -3.00453644 | 0.007301492 | 0.025674568 | OR5BA1P |
| ENSG00000231665 | 12.94587817 | 1.781856508 | 0.007308392 | 0.025695162 | OGFOD1P1 |
| ENSG00000232878 | 7.982088823 | -1.903206376 | 0.007487639 | 0.026253926 | DPYD-AS1 |
| ENSG00000137821 | 8.453448626 | -1.839601365 | 0.007511329 | 0.026329735 | LRRC49 |
| ENSG00000232403 | 2.014004309 | 4.496767115 | 0.007557074 | 0.026471252 | RPL27P5 |
| ENSG00000152465 | 159.3551072 | 1.428504955 | 0.00755911 | 0.026474619 | NMT2 |
| ENSG00000119888 | 2.103848985 | -4.502890308 | 0.007565381 | 0.026488684 | EPCAM |
| ENSG00000250159 | 25.74863659 | -1.041236851 | 0.007565728 | 0.026488684 | PKD2L2-DT |
| ENSG00000197565 | 2.23411687 | -4.593119491 | 0.007665548 | 0.02681309 | COL4A6 |
| ENSG00000215277 | 9.655864245 | -1.768357675 | 0.007682909 | 0.026870001 | RNF212B |
| ENSG00000229927 | 26.26664533 | 1.047644654 | 0.00773262 | 0.027024674 | RHEBP1 |
| ENSG00000226851 | 14.67009168 | -1.48131634 | 0.007734196 | 0.027026347 | ZNF277-AS1 |
| ENSG00000151276 | 6.190696751 | -2.350752822 | 0.007748575 | 0.027068911 | MAGI1 |
| ENSG00000188859 | 5.992174786 | -2.62575657 | 0.007774498 | 0.027145799 | FAM78B |
| ENSG00000039068 | 2.110772115 | -4.506717055 | 0.007787661 | 0.027174625 | CDH1 |
| ENSG00000070031 | 2.173200176 | 4.607457538 | 0.007792592 | 0.027184127 | SCT |
| ENSG00000237624 | 13.71430105 | -1.441122329 | 0.007832009 | 0.02730966 | OXCT2P1 |
| ENSG00000213830 | 17.87147555 | 1.424738816 | 0.007833012 | 0.02730966 | CFL1P5 |
| ENSG00000064886 | 13.03968518 | -1.431872095 | 0.007871324 | 0.027420012 | CHI3L2 |
| ENSG00000215788 | 5.728274853 | -2.528811226 | 0.007888667 | 0.027468708 | TNFRSF25 |
| ENSG00000088882 | 11.40706886 | -1.700247316 | 0.007921347 | 0.027563018 | CPXM1 |
| ENSG00000235034 | 19.5404279 | 1.238434215 | 0.007936871 | 0.027609236 | C19orf81 |
| ENSG00000249619 | 5.632033471 | 3.399882133 | 0.008043544 | 0.027913296 | HMGN1P13 |
| ENSG00000234062 | 13.88719196 | -1.374806083 | 0.008098205 | 0.028075299 | TM9SF5P |
| ENSG00000243674 | 2.02773758 | 4.504535005 | 0.008107773 | 0.028100557 | OR7E66P |
| ENSG00000070388 | 3.094984752 | -4.017150628 | 0.008164395 | 0.028272935 | FGF22 |
| ENSG00000092607 | 63.42578292 | 1.090167384 | 0.008179736 | 0.028314117 | TBX15 |
| ENSG00000260615 | 5.625762451 | 2.949057122 | 0.008184821 | 0.028323757 | RPL23AP97 |
| ENSG00000179094 | 1156.246554 | 1.181204089 | 0.008195733 | 0.028357535 | PER1 |
| ENSG00000137270 | 12.46543977 | -1.743800722 | 0.008227057 | 0.028454405 | GCM1 |
| ENSG00000237766 | 4.012510247 | 3.510910728 | 0.008264639 | 0.028547833 | GGTA2P |
| ENSG00000165309 | 10.8806475 | -1.870982375 | 0.008310607 | 0.028696956 | ARMC3 |
| ENSG00000270433 | 5.814954735 | 2.63771659 | 0.008311306 | 0.028696956 | H3P37 |
| ENSG00000013588 | 28.4154046 | 2.887483954 | 0.008345068 | 0.028805454 | GPRC5A |
| ENSG00000270587 | 13.67392796 | -1.433228271 | 0.008368599 | 0.028866453 | FAM136DP |
| ENSG00000230417 | 8.275546434 | 2.048607178 | 0.008377995 | 0.028890771 | LINC00595 |
| ENSG00000269012 | 29.17226936 | 1.035972815 | 0.008410509 | 0.028990715 | KRT18P40 |
| ENSG00000130294 | 2.109554335 | -4.506124208 | 0.008460052 | 0.029141099 | KIF1A |
| ENSG00000114646 | 12.03540801 | -1.812726553 | 0.008477482 | 0.029192972 | CSPG5 |
| ENSG00000163220 | 9.931900425 | 2.358669234 | 0.008488538 | 0.029218791 | S100A9 |
| ENSG00000153904 | 16.48224342 | -1.468100922 | 0.008517266 | 0.029297207 | DDAH1 |
| ENSG00000226397 | 5.039397336 | -2.704846895 | 0.008525208 | 0.029312245 | LINC02909 |
| ENSG00000137507 | 9.288972692 | 2.239090294 | 0.008530664 | 0.02932691 | LRRC32 |
| ENSG00000138379 | 17.38602283 | -1.283542488 | 0.008578898 | 0.029468053 | MSTN |
| ENSG00000008226 | 19.28448394 | 1.15252442 | 0.00861388 | 0.029567601 | DLEC1 |
| ENSG00000104611 | 12.0945114 | -1.474896173 | 0.00870195 | 0.029844949 | SH2D4A |
| ENSG00000229563 | 6.198228771 | -2.351978448 | 0.00880704 | 0.030159184 | LINC01204 |
| ENSG00000163632 | 4.70473265 | -3.069611327 | 0.00881732 | 0.030190189 | C3orf49 |
| ENSG00000203688 | 8.009337562 | -2.295509269 | 0.008835092 | 0.03023843 | LINC02487 |
| ENSG00000171877 | 7.798527714 | -2.061039263 | 0.008850782 | 0.030283715 | FRMD5 |
| ENSG00000185585 | 11.30000619 | -1.562024425 | 0.008863528 | 0.030318904 | OLFML2A |
| ENSG00000142408 | 4.880757332 | -2.645464581 | 0.008873567 | 0.030343242 | CACNG8 |
| ENSG00000147381 | 5.025551075 | -2.701463972 | 0.008874339 | 0.030343242 | MAGEA4 |
| ENSG00000089199 | 7.02213761 | -2.320547586 | 0.009008878 | 0.030734999 | CHGB |
| ENSG00000223592 | 8.429706742 | 2.084639609 | 0.009014402 | 0.030749585 | FNDC3CP |
| ENSG00000134640 | 2.296425044 | -4.62632514 | 0.00901922 | 0.030757502 | MTNR1B |
| ENSG00000223117 | 2.918156737 | -3.928933349 | 0.00902581 | 0.030775714 | RN7SKP296 |
| ENSG00000160207 | 14.25012018 | -1.335233983 | 0.009125042 | 0.031058178 | HSF2BP |
| ENSG00000138769 | 5.040064924 | -2.708602175 | 0.009129966 | 0.031070644 | CDKL2 |
| ENSG00000100473 | 14.97151503 | -1.365988164 | 0.009151792 | 0.031140619 | COCH |
| ENSG00000163395 | 49.24903104 | 1.116265663 | 0.009201926 | 0.031268014 | IGFN1 |
| ENSG00000152661 | 7.664349881 | -2.015268265 | 0.009248126 | 0.031377388 | GJA1 |
| ENSG00000259343 | 12.26162277 | -1.611381435 | 0.00930482 | 0.03154425 | TMC3-AS1 |
| ENSG00000253669 | 48.37909745 | -2.696437644 | 0.00930499 | 0.03154425 | GASAL1 |
| ENSG00000099204 | 3.22464595 | -4.081680147 | 0.009342254 | 0.031640095 | ABLIM1 |
| ENSG00000101306 | 3.886567038 | -3.397016685 | 0.00940083 | 0.031807863 | MYLK2 |
| ENSG00000169314 | 4.690577765 | 2.64267136 | 0.009405849 | 0.031820475 | C22orf15 |
| ENSG00000169129 | 2.106359658 | -4.504322994 | 0.009478976 | 0.032015101 | AFAP1L2 |
| ENSG00000212237 | 8.56100862 | 1.923348643 | 0.009527452 | 0.032161189 | RNA5SP18 |
| ENSG00000278416 | 10.27047616 | -1.616153267 | 0.009620194 | 0.032443131 | PMS2P2 |
| ENSG00000258701 | 12.42730146 | -1.521541902 | 0.009627961 | 0.03246488 | LINC00638 |
| ENSG00000167646 | 2.909275328 | -3.92262329 | 0.009688396 | 0.032632923 | DNAAF3 |
| ENSG00000186652 | 150.1957185 | -2.046312437 | 0.00978499 | 0.032922262 | PRG2 |
| ENSG00000284713 | 8.905899446 | 2.572597703 | 0.009865282 | 0.033151656 | SMIM38 |
| ENSG00000238018 | 39.7574824 | 1.061028837 | 0.009916192 | 0.033300019 | SPTBN1-AS2 |
| ENSG00000261739 | 28.78687174 | 1.059576108 | 0.009935096 | 0.033345317 | GOLGA8S |
| ENSG00000183463 | 1.933944101 | -4.382654525 | 0.009941355 | 0.03336178 | URAD |
| ENSG00000226539 | 3.679767894 | 3.37129764 | 0.00996861 | 0.033444131 | MLXP1 |
| ENSG00000101049 | 17.07784418 | 1.206519832 | 0.0100163 | 0.033581258 | SGK2 |
| ENSG00000125508 | 5.819362202 | 2.994890224 | 0.010100384 | 0.033826331 | SRMS |
| ENSG00000257122 | 38.31202182 | -1.071735432 | 0.010162364 | 0.03401078 | RRN3P3 |
| ENSG00000168453 | 9.581722381 | -1.765066475 | 0.010195278 | 0.034107035 | HR |
| ENSG00000100146 | 3.676646128 | 3.370238097 | 0.010218278 | 0.034174693 | SOX10 |
| ENSG00000164591 | 6.984392601 | 2.143980971 | 0.010286258 | 0.034355406 | MYOZ3 |
| ENSG00000182240 | 26.15399728 | -1.088285819 | 0.010313428 | 0.034432143 | BACE2 |
| ENSG00000206754 | 20.61426773 | -1.162072887 | 0.010410538 | 0.034718708 | SNORD101 |
| ENSG00000168314 | 2.913746484 | -3.925934907 | 0.010470543 | 0.034904644 | MOBP |
| ENSG00000185052 | 11.41634416 | -1.475535383 | 0.010525076 | 0.035057967 | SLC24A3 |
| ENSG00000184613 | 5.363379811 | -2.433921809 | 0.010563998 | 0.03516859 | NELL2 |
| ENSG00000184709 | 8.647746238 | -1.713490427 | 0.010628712 | 0.035341042 | LRRC26 |
| ENSG00000203804 | 18.69438003 | 1.266826945 | 0.010631737 | 0.035346328 | ADAMTSL4-AS1 |
| ENSG00000151715 | 3.986868527 | 3.509207181 | 0.010641355 | 0.035365011 | TMEM45B |
| ENSG00000225400 | 4.182969729 | 2.930340388 | 0.010649393 | 0.035385922 | RAB28P5 |
| ENSG00000262655 | 7.001912072 | -2.080900814 | 0.010654432 | 0.035393117 | SPON1 |
| ENSG00000221493 | 2.850640103 | 3.950235935 | 0.010666651 | 0.035428929 | MIR320C1 |
| ENSG00000235330 | 2.100120531 | -4.500847855 | 0.010807171 | 0.035832056 | RPL12P25 |
| ENSG00000207561 | 14.58074636 | 1.55674037 | 0.010808386 | 0.035832056 | MIR635 |
| ENSG00000279720 | 4.694907505 | 2.648110223 | 0.010819208 | 0.035858279 | SOWAHCP2 |
| ENSG00000196456 | 32.3014547 | 1.017259183 | 0.010850482 | 0.035942585 | ZNF775 |
| ENSG00000183378 | 2.940146112 | -3.936725023 | 0.010886007 | 0.036041007 | OVCH2 |
| ENSG00000165168 | 4141.581845 | -1.686385183 | 0.010899055 | 0.036079227 | CYBB |
| ENSG00000239282 | 11.11456727 | 1.499671038 | 0.010912227 | 0.036103991 | CASTOR1 |
| ENSG00000237977 | 10.23919405 | -1.621452289 | 0.010912289 | 0.036103991 | EIF4HP2 |
| ENSG00000185988 | 6.645406716 | 2.054104846 | 0.010991729 | 0.036337174 | PLK5 |
| ENSG00000189001 | 3.678420709 | 3.38590196 | 0.01100144 | 0.036364395 | SBSN |
| ENSG00000148468 | 3.863341265 | -3.402675891 | 0.01100542 | 0.03637267 | FAM171A1 |
| ENSG00000105976 | 20.42901862 | 1.268852178 | 0.011123136 | 0.03671738 | MET |
| ENSG00000231715 | 2.023369609 | 4.502200117 | 0.01117721 | 0.036866234 | COX6CP2 |
| ENSG00000224614 | 16.35372194 | -1.237956682 | 0.011236541 | 0.03703713 | TNK2-AS1 |
| ENSG00000140905 | 24.35718704 | 1.173259003 | 0.011283061 | 0.037160631 | GCSH |
| ENSG00000268598 | 5.817502375 | 2.326695321 | 0.011318051 | 0.037261769 | VN1R80P |
| ENSG00000278048 | 5.67741085 | -2.534157307 | 0.011353205 | 0.037356685 | U2 |
| ENSG00000175315 | 7.352286926 | -1.944960638 | 0.01148401 | 0.037716551 | CST6 |
| ENSG00000226416 | 1.842944479 | 4.369223625 | 0.011612277 | 0.038066757 | MRPL23-AS1 |
| ENSG00000235258 | 1.842944479 | 4.369223625 | 0.011612277 | 0.038066757 | NDUFB4P6 |
| ENSG00000268535 | 7.998926873 | 1.987058567 | 0.011662676 | 0.038221797 | MTDHP4 |
| ENSG00000142182 | 5.32177733 | 2.848782561 | 0.011763153 | 0.038484412 | DNMT3L |
| ENSG00000253819 | 4.208107344 | -2.885360518 | 0.011765038 | 0.038484412 | LINC01151 |
| ENSG00000197935 | 6.324868246 | -2.13991245 | 0.011767806 | 0.038484412 | ZNF311 |
| ENSG00000226415 | 32.01522292 | -1.006138128 | 0.011859719 | 0.038754126 | TPI1P1 |
| ENSG00000101916 | 7.139643473 | -2.111261403 | 0.01186653 | 0.038766095 | TLR8 |
| ENSG00000164484 | 23.12346027 | -1.034634865 | 0.01192276 | 0.038934301 | TMEM200A |
| ENSG00000171951 | 748.460156 | 1.036800914 | 0.011950026 | 0.039007823 | SCG2 |
| ENSG00000197291 | 2.769573639 | -3.84423562 | 0.011970315 | 0.039063699 | RAMP2-AS1 |
| ENSG00000224680 | 15.71462198 | -1.304365014 | 0.012036332 | 0.039268732 | PLA2G12AP1 |
| ENSG00000108821 | 36.68966584 | -1.144377157 | 0.012120273 | 0.039516421 | COL1A1 |
| ENSG00000263515 | 11.10027662 | -1.521849193 | 0.0122414 | 0.039869121 | MIR548AN |
| ENSG00000163659 | 1432.946733 | 1.079759634 | 0.012308199 | 0.040060194 | TIPARP |
| ENSG00000065618 | 3.665933643 | 3.382295575 | 0.012388309 | 0.040278357 | COL17A1 |
| ENSG00000184293 | 24.41799439 | -1.164069219 | 0.012414709 | 0.040353539 | CLECL1 |
| ENSG00000144550 | 27.26997437 | -1.091469589 | 0.012431749 | 0.040392936 | CPNE9 |
| ENSG00000200204 | 16.47608574 | 1.327239986 | 0.012465294 | 0.040480569 | RNU1-22P |
| ENSG00000265485 | 9.584703997 | -1.624534459 | 0.012482524 | 0.040519625 | LINC01915 |
| ENSG00000266969 | 26.13301428 | -1.032605437 | 0.012546467 | 0.04069053 | CEP192-DT |
| ENSG00000137727 | 21.10416184 | -1.098912381 | 0.012609614 | 0.040884562 | ARHGAP20 |
| ENSG00000166866 | 14.42524432 | -1.36463971 | 0.012675963 | 0.041083463 | MYO1A |
| ENSG00000245213 | 27.5794318 | 1.077745032 | 0.012717125 | 0.041178945 | GALNT7-DT |
| ENSG00000100985 | 17216.32717 | 1.250121538 | 0.01276321 | 0.041311879 | MMP9 |
| ENSG00000231426 | 24.85012935 | -1.135444353 | 0.012814547 | 0.041461699 | FILNC1 |
| ENSG00000174225 | 15.57332175 | 1.357759722 | 0.012821958 | 0.04148023 | ARL13A |
| ENSG00000247373 | 28.29896739 | 1.049743407 | 0.012841786 | 0.041538919 | TMED2-DT |
| ENSG00000171219 | 11.43542703 | -1.589094924 | 0.012846832 | 0.041549784 | CDC42BPG |
| ENSG00000233613 | 18.8252628 | 1.407599156 | 0.013010485 | 0.042007375 | DCUN1D2-AS |
| ENSG00000234753 | 15.10800548 | 1.228506741 | 0.013044921 | 0.042102005 | FOXP4-AS1 |
| ENSG00000138152 | 4.393663697 | -2.933890957 | 0.013061689 | 0.042150598 | BTBD16 |
| ENSG00000157343 | 4.694434612 | -2.581875423 | 0.013083116 | 0.042208685 | ARMC12 |
| ENSG00000227344 | 6.513462711 | -1.942546815 | 0.013101277 | 0.042256208 | HAUS6P1 |
| ENSG00000118492 | 3.566231564 | -3.264235719 | 0.013108298 | 0.042262252 | ADGB |
| ENSG00000218676 | 8.486151762 | 1.748911717 | 0.013111095 | 0.042265738 | BRD7P4 |
| ENSG00000249467 | 1.961636622 | -4.399395197 | 0.013113069 | 0.042266569 | H2AL1Q |
| ENSG00000182950 | 26.93845032 | 1.264110996 | 0.013124401 | 0.042296856 | ODF3L1 |
| ENSG00000270011 | 13.92741699 | 1.372847305 | 0.013176591 | 0.042381585 | ZNF559-ZNF177 |
| ENSG00000239959 | 5.138250901 | 2.792705238 | 0.013180662 | 0.042384603 | ENPP7P2 |
| ENSG00000244286 | 14.39367746 | 1.443665469 | 0.01318514 | 0.042393471 | ITGB5-AS1 |
| ENSG00000203808 | 9.664664202 | -1.63466694 | 0.01319889 | 0.042427214 | BVES-AS1 |
| ENSG00000169885 | 8.15181349 | 1.854412379 | 0.013238913 | 0.042533051 | CALML6 |
| ENSG00000137868 | 1.954104602 | -4.394843501 | 0.013250318 | 0.042556267 | STRA6 |
| ENSG00000197444 | 9.131127564 | -1.820884273 | 0.013339377 | 0.042794417 | OGDHL |
| ENSG00000250420 | 4.211299818 | -2.890220057 | 0.013408493 | 0.042982561 | AACSP1 |
| ENSG00000201616 | 3.308880257 | 4.18021344 | 0.013416234 | 0.042996186 | RNU1-91P |
| ENSG00000120055 | 12.97896017 | 1.317966284 | 0.013434962 | 0.043036724 | C10orf95 |
| ENSG00000103067 | 13.91301773 | -1.37449723 | 0.013435381 | 0.043036724 | ESRP2 |
| ENSG00000103599 | 17.49922289 | -1.168256821 | 0.013468793 | 0.043130956 | IQCH |
| ENSG00000200677 | 2.836906832 | 3.944277361 | 0.013591429 | 0.043484099 | SNORD18 |
| ENSG00000184923 | 15.65333494 | -1.222618489 | 0.013705485 | 0.043763741 | NUTM2A |
| ENSG00000213250 | 6.538644559 | -1.947433796 | 0.013812656 | 0.04404315 | RBMS2P1 |
| ENSG00000167880 | 7.485237174 | -1.989638459 | 0.013843648 | 0.044130549 | EVPL |
| ENSG00000104938 | 4.5572458 | -2.526644891 | 0.013902794 | 0.044308572 | CLEC4M |
| ENSG00000203797 | 24.39184886 | -1.022249896 | 0.013908896 | 0.044315603 | DDO |
| ENSG00000022556 | 8.002846205 | -1.904732316 | 0.013914881 | 0.044325179 | NLRP2 |
| ENSG00000260526 | 11.67168417 | 1.479264211 | 0.0139155 | 0.044325179 | AP1AR-DT |
| ENSG00000243811 | 5.493558715 | 2.218906632 | 0.014063274 | 0.044766939 | APOBEC3D |
| ENSG00000173825 | 10.44165303 | 1.844916543 | 0.014084534 | 0.044811451 | TIGD3 |
| ENSG00000260325 | 10.96816344 | -1.499442859 | 0.014087217 | 0.044814197 | HSPB9 |
| ENSG00000260799 | 2.756392764 | -3.841596913 | 0.01410106 | 0.04484665 | KRT8P50 |
| ENSG00000207962 | 8.777757152 | -1.918100577 | 0.014150692 | 0.044978036 | MIR30C1 |
| ENSG00000228624 | 35.27836615 | 1.087003383 | 0.014326438 | 0.045446081 | HDAC2-AS2 |
| ENSG00000227759 | 1.866666151 | 4.384118356 | 0.014328557 | 0.045446951 | VTI1BP4 |
| ENSG00000222069 | 5.546398331 | -2.171142907 | 0.014362825 | 0.04553219 | RN7SKP285 |
| ENSG00000138134 | 58.73604129 | -2.085914148 | 0.014466687 | 0.045826058 | STAMBPL1 |
| ENSG00000205930 | 13.91344418 | -1.287138784 | 0.014478749 | 0.045849894 | C21orf62-AS1 |
| ENSG00000240370 | 21.83045187 | 1.056320617 | 0.014578556 | 0.046150751 | RPL13P5 |
| ENSG00000101333 | 6.867076457 | -2.031119652 | 0.014624114 | 0.046253362 | PLCB4 |
| ENSG00000218208 | 7.320793041 | -1.937236893 | 0.014632553 | 0.04627411 | RPS27AP11 |
| ENSG00000262484 | 10.78719032 | -1.590115306 | 0.014681639 | 0.046411466 | CCER2 |
| ENSG00000164185 | 12.9409831 | 1.310722509 | 0.01469206 | 0.046432489 | ZNF474 |
| ENSG00000170382 | 19.44925844 | -1.090739095 | 0.014730383 | 0.046547634 | LRRN2 |
| ENSG00000109321 | 23.06912252 | -1.192744381 | 0.014836872 | 0.04684207 | AREG |
| ENSG00000165066 | 3.58633557 | -3.2616235 | 0.014886187 | 0.046980693 | NKX6-3 |
| ENSG00000060718 | 3.564329781 | -3.263522445 | 0.014911988 | 0.047050904 | COL11A1 |
| ENSG00000171970 | 23.19658688 | 1.042179671 | 0.014944213 | 0.047123073 | ZNF57 |
| ENSG00000178947 | 3.55618887 | -3.260798363 | 0.014984607 | 0.047211671 | SMIM10L2A |
| ENSG00000204956 | 4.538418272 | -2.531375527 | 0.015000062 | 0.047248276 | PCDHGA1 |
| ENSG00000108448 | 42.60833842 | 1.02537533 | 0.015049085 | 0.047348199 | TRIM16L |
| ENSG00000255760 | 24.66691049 | -1.048660876 | 0.015081221 | 0.047443248 | LINC02422 |
| ENSG00000144681 | 12.75785384 | -1.389432654 | 0.015155057 | 0.047645102 | STAC |
| ENSG00000243431 | 2.763999898 | -3.845033735 | 0.015229568 | 0.047830514 | RPL5P30 |
| ENSG00000251513 | 5.331885926 | -2.423907353 | 0.015416069 | 0.048313067 | LIX1-AS1 |
| ENSG00000126231 | 6.669693023 | -1.996852168 | 0.015438238 | 0.048356392 | PROZ |
| ENSG00000251768 | 4.362832741 | 2.517258848 | 0.015444625 | 0.048370248 | RNA5SP217 |
| ENSG00000204960 | 5.843467953 | -2.262027996 | 0.015467136 | 0.048416127 | BLACE |
| ENSG00000256280 | 6.855164456 | -2.029838227 | 0.015510265 | 0.048532627 | ATP5MGP1 |
| ENSG00000205277 | 2.583977206 | -3.743738446 | 0.015521305 | 0.048561003 | MUC12 |
| ENSG00000232485 | 4.484505161 | 2.57794966 | 0.0155402 | 0.048607772 | RPL37A-DT |
| ENSG00000162757 | 4.043177803 | 2.863458447 | 0.015552274 | 0.048623769 | C1orf74 |
| ENSG00000235472 | 5.02768462 | 2.386647832 | 0.015559372 | 0.04863686 | EIF4A1P7 |
| ENSG00000067601 | 23.82792084 | -1.017816856 | 0.015607408 | 0.048756078 | PMS2P4 |
| ENSG00000151240 | 1.77408191 | -4.257916187 | 0.015631312 | 0.048818368 | DIP2C |
| ENSG00000179978 | 24.82162253 | 1.041740913 | 0.015719795 | 0.049045218 | NAIPP2 |
| ENSG00000238269 | 1.841698274 | 4.368325461 | 0.015719877 | 0.049045218 | PAGE2B |
| ENSG00000139269 | 11.25516726 | 1.627458735 | 0.01572828 | 0.049065223 | INHBE |
| ENSG00000234557 | 2.607200776 | -3.75113081 | 0.015743346 | 0.049106003 | PPIAP90 |
| ENSG00000237840 | 8.984355003 | 1.569140922 | 0.015748627 | 0.049116254 | FAM21FP |
| ENSG00000178732 | 17.05782289 | 1.205948378 | 0.015750952 | 0.049117158 | GP5 |
| ENSG00000236992 | 9.316466733 | 1.509032998 | 0.015787495 | 0.04921255 | RPL12P12 |
| ENSG00000079385 | 12.77026296 | 1.3816746 | 0.015792727 | 0.049222631 | CEACAM1 |
| ENSG00000253199 | 3.599556526 | -3.271092544 | 0.015805174 | 0.049236506 | LINC02153 |
| ENSG00000213413 | 19.21063787 | -1.115827557 | 0.015860254 | 0.049370628 | PVRIG |
| ENSG00000277449 | 14.19482096 | -1.254367917 | 0.01587679 | 0.049403372 | CEBPB-AS1 |
| ENSG00000170128 | 17.38644295 | -1.15037164 | 0.015922541 | 0.049539475 | GPR25 |
| ENSG00000189184 | 17.17931337 | 1.205600025 | 0.015962401 | 0.049650948 | PCDH18 |
| ENSG00000203910 | 16.71173648 | -1.276573666 | 0.016000017 | 0.049736551 | C1orf146 |
| ENSG00000099810 | 1.787928171 | -4.266944566 | 0.016022043 | 0.049786168 | MTAP |
| ENSG00000248712 | 8.803070033 | -1.595942215 | 0.016050905 | 0.049863272 | CCDC153 |
| ENSG00000267757 | 3.709109997 | 3.379875806 | 0.016079086 | 0.04994452 | EML2-AS1 |
| ENSG00000234534 | 1.784808608 | -4.26494567 | 0.01609631 | 0.049988237 | CSNK1G2P1 |
| ENSG00000187260 | 7.485156756 | 1.865455027 | 0.016097219 | 0.049988237 | WDR86 |
