# Supplementary material for: Circulating mitochondrial DNA promotes M2 polarization of tumor associated macrophages and HCC resistance to sorafenib
Source: Cell Death Dis. 2025 Mar 4;16(1):153. doi: 10.1038/s41419-025-07473-8 (PMC11880550; doi:10.1038/s41419-025-07473-8)

Fig.1F

β-actin

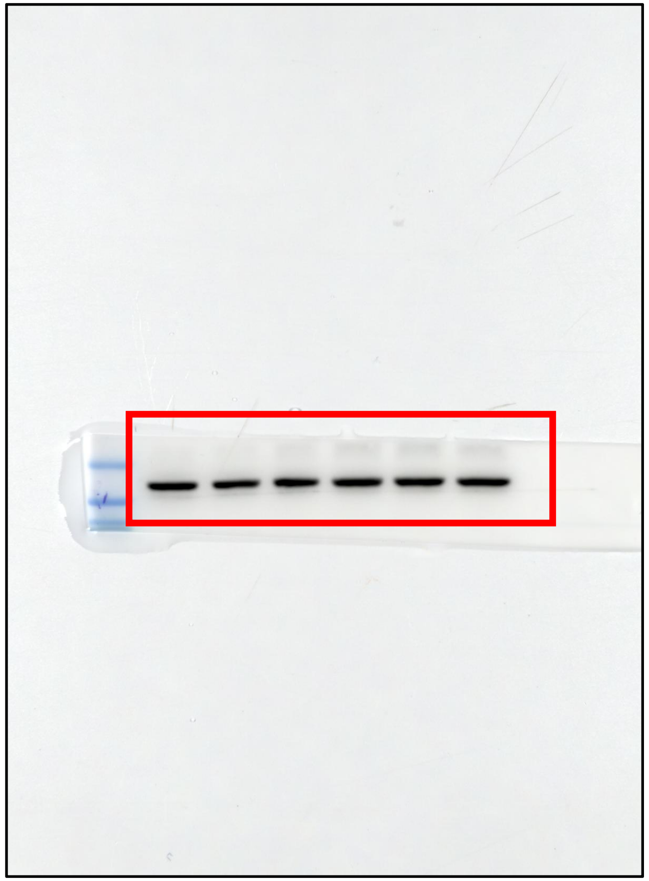

CD206

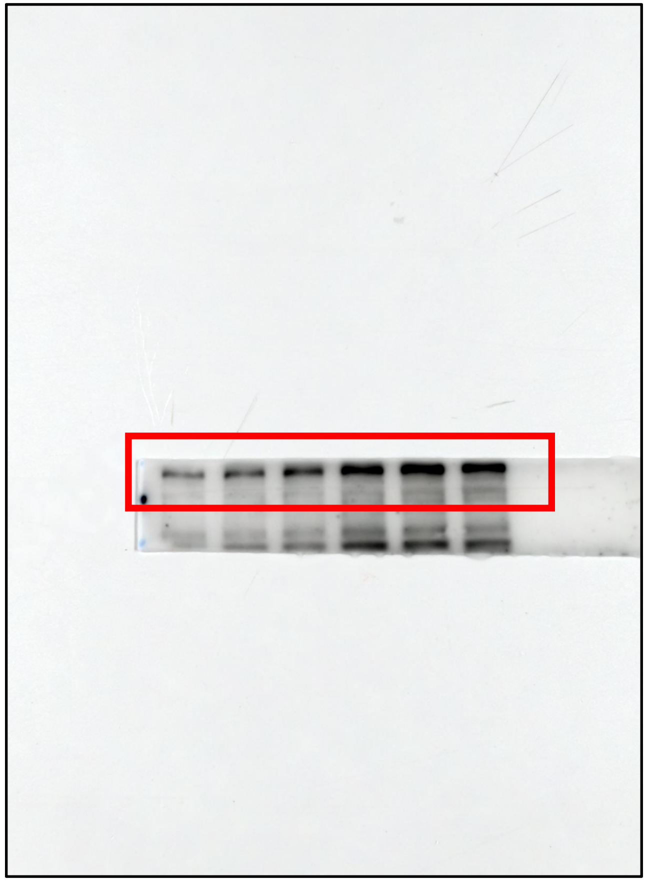

Fig.4B

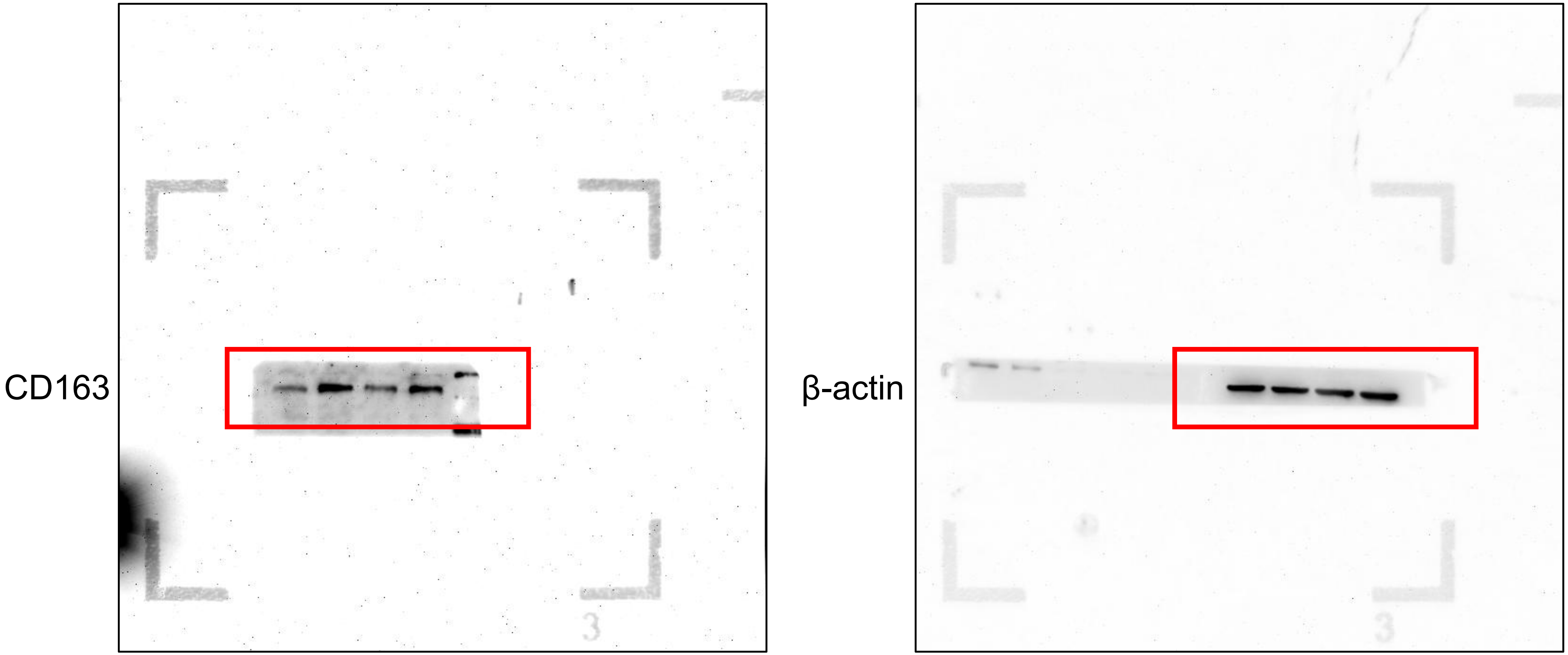

Fig.4B

CD206

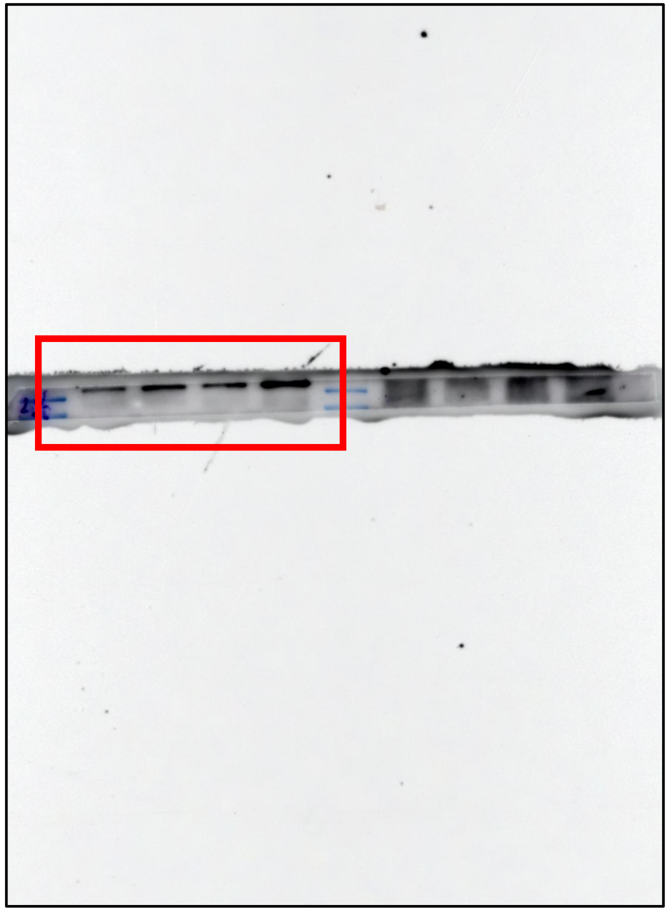

β-actin

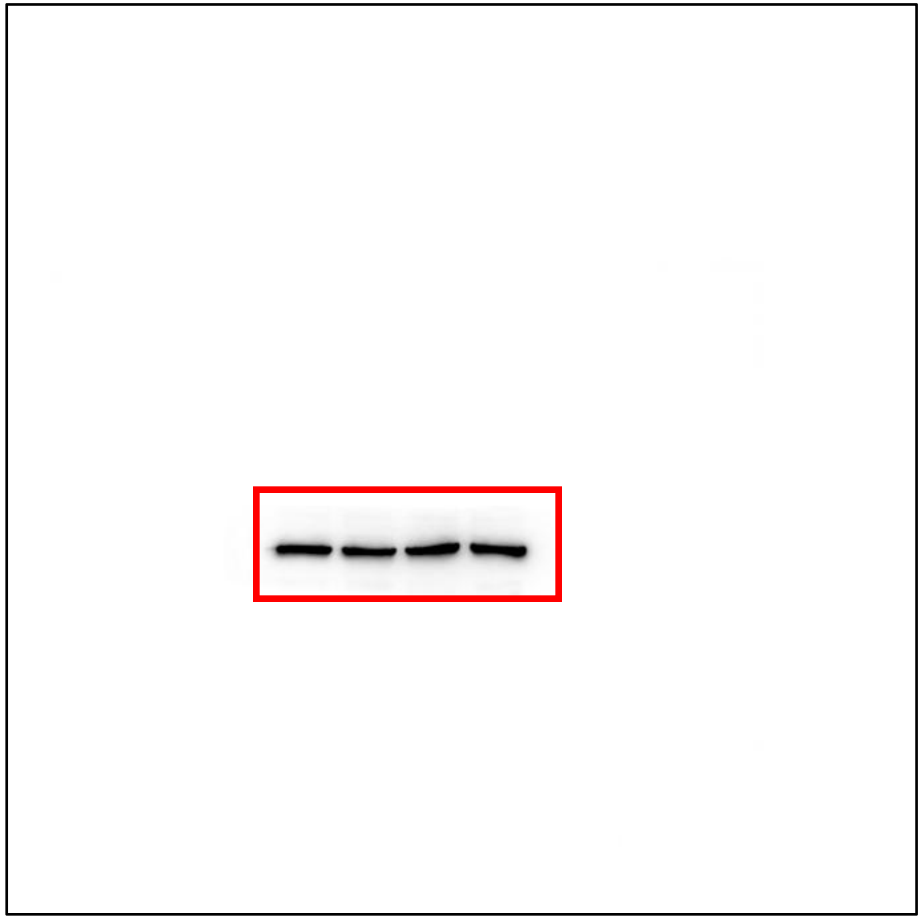

Fig.5F

CD206

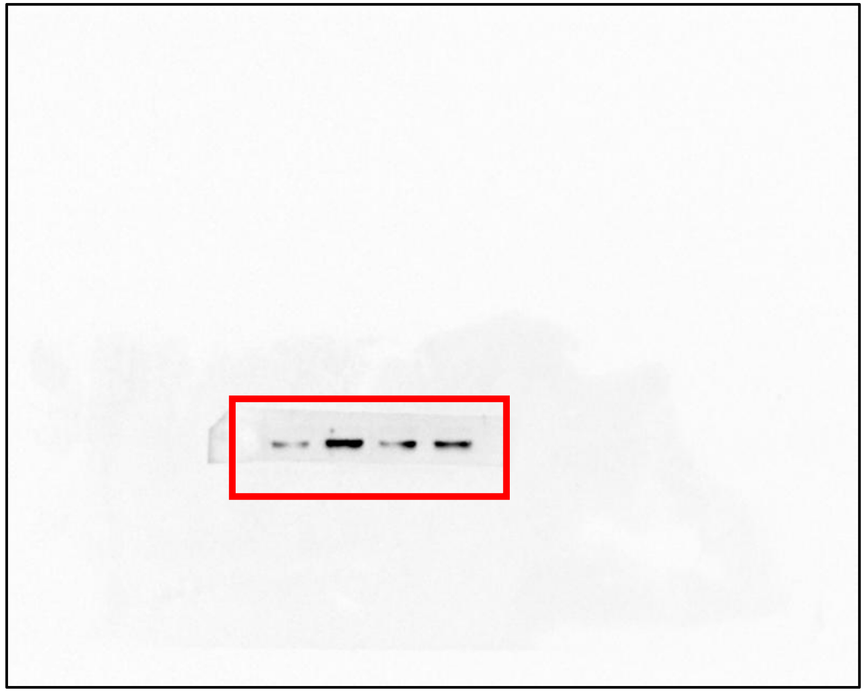

β-actin

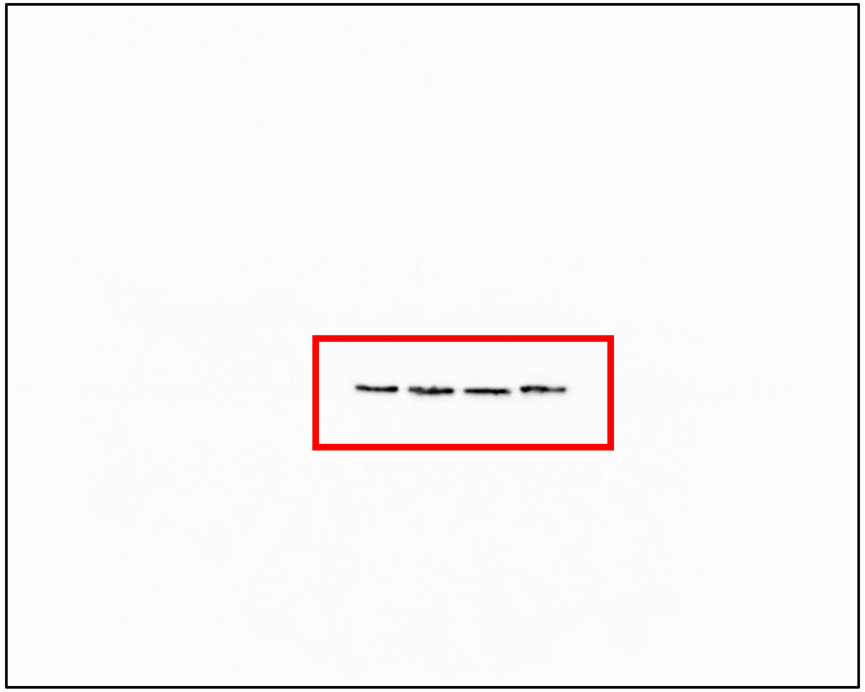

Fig.6D

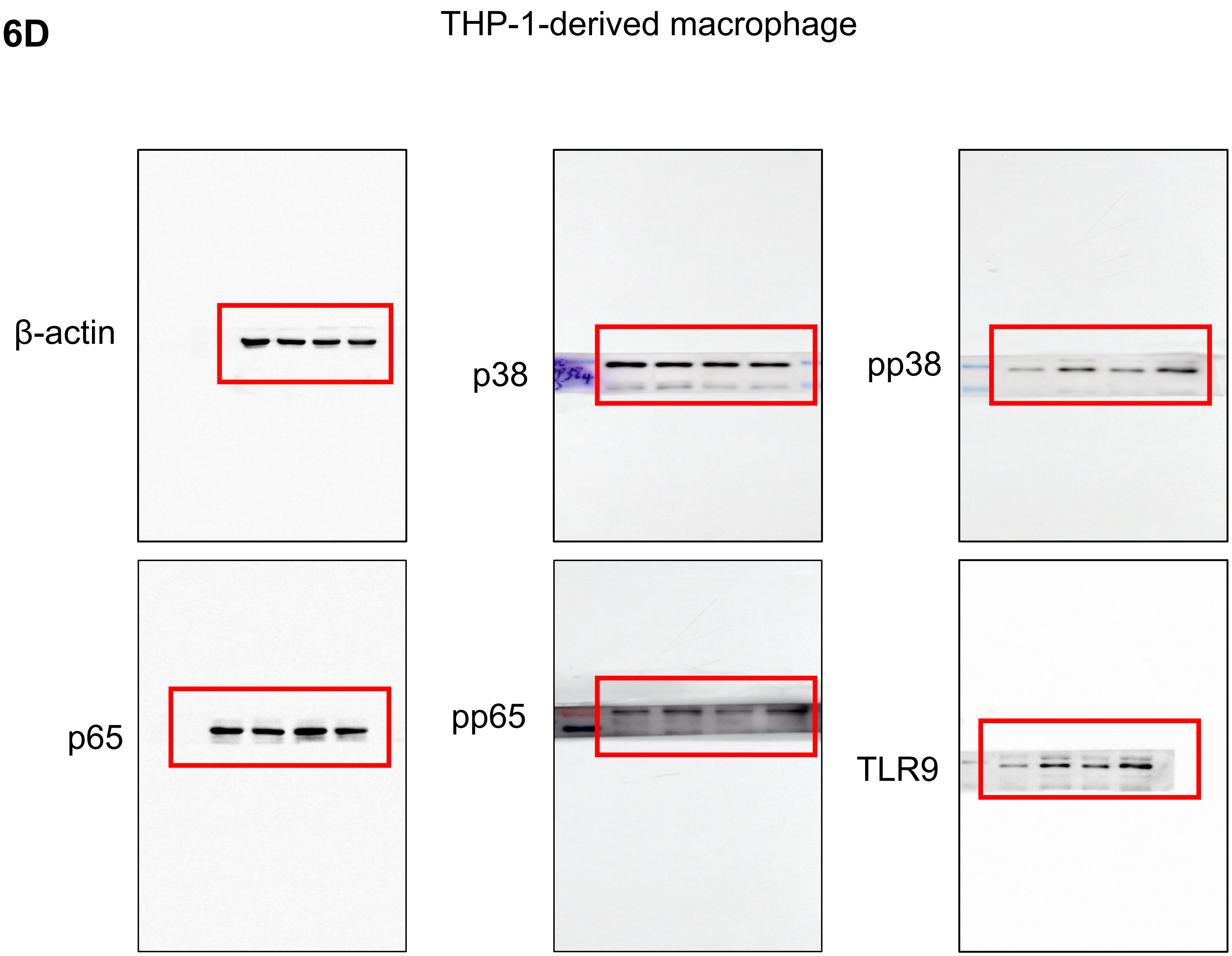

Fig.6D

RAW264.7

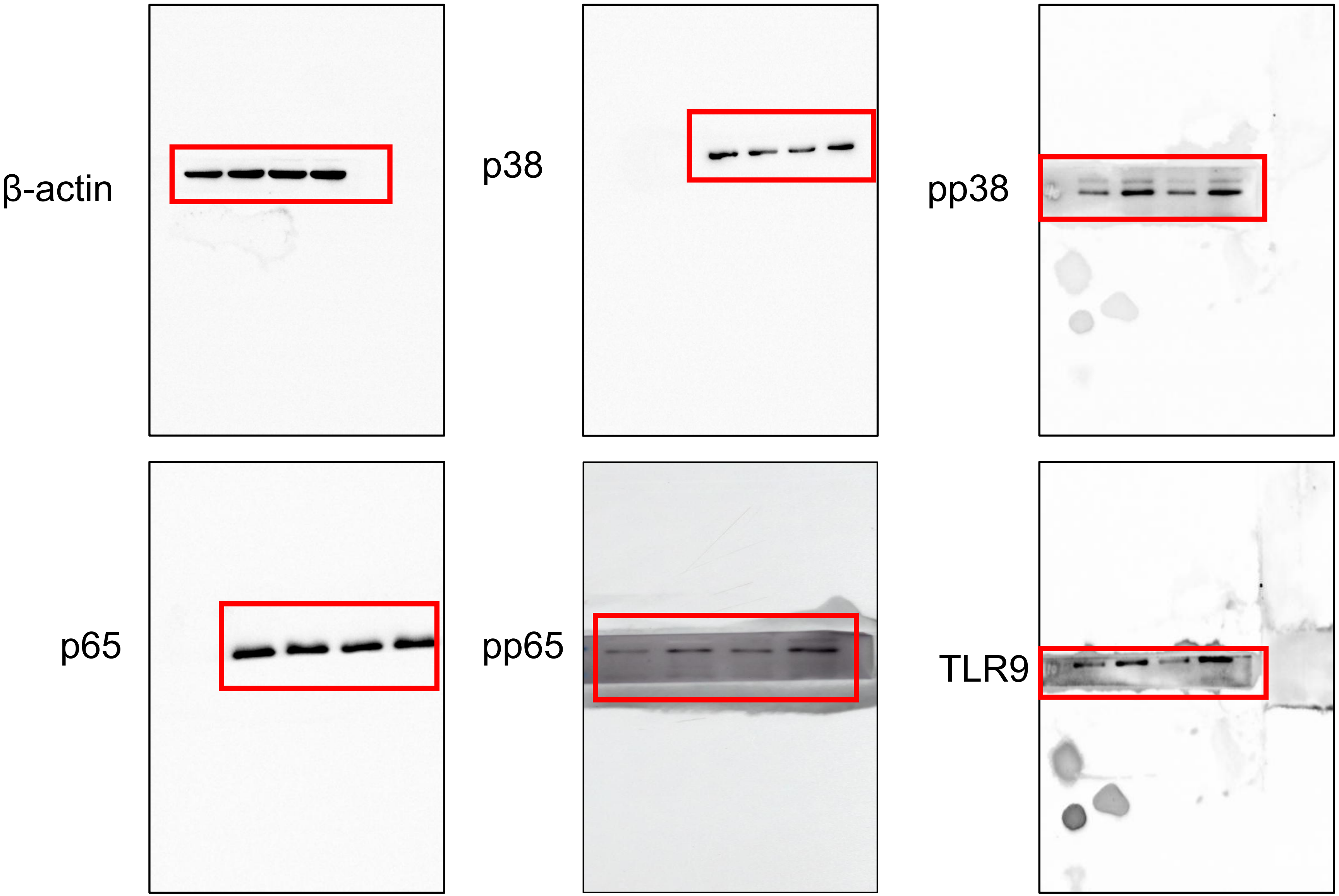

Fig.7B

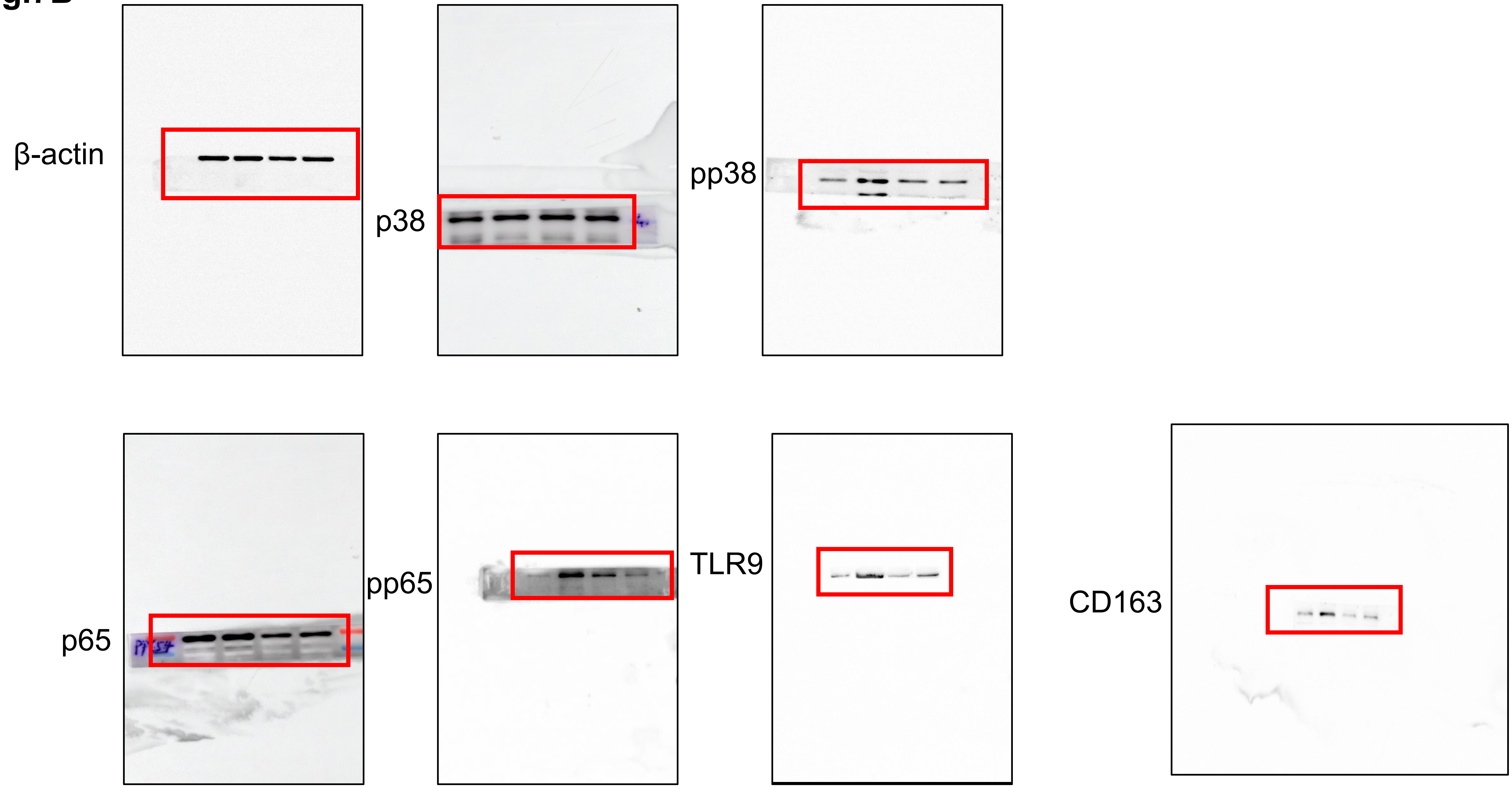

Fig. S5B

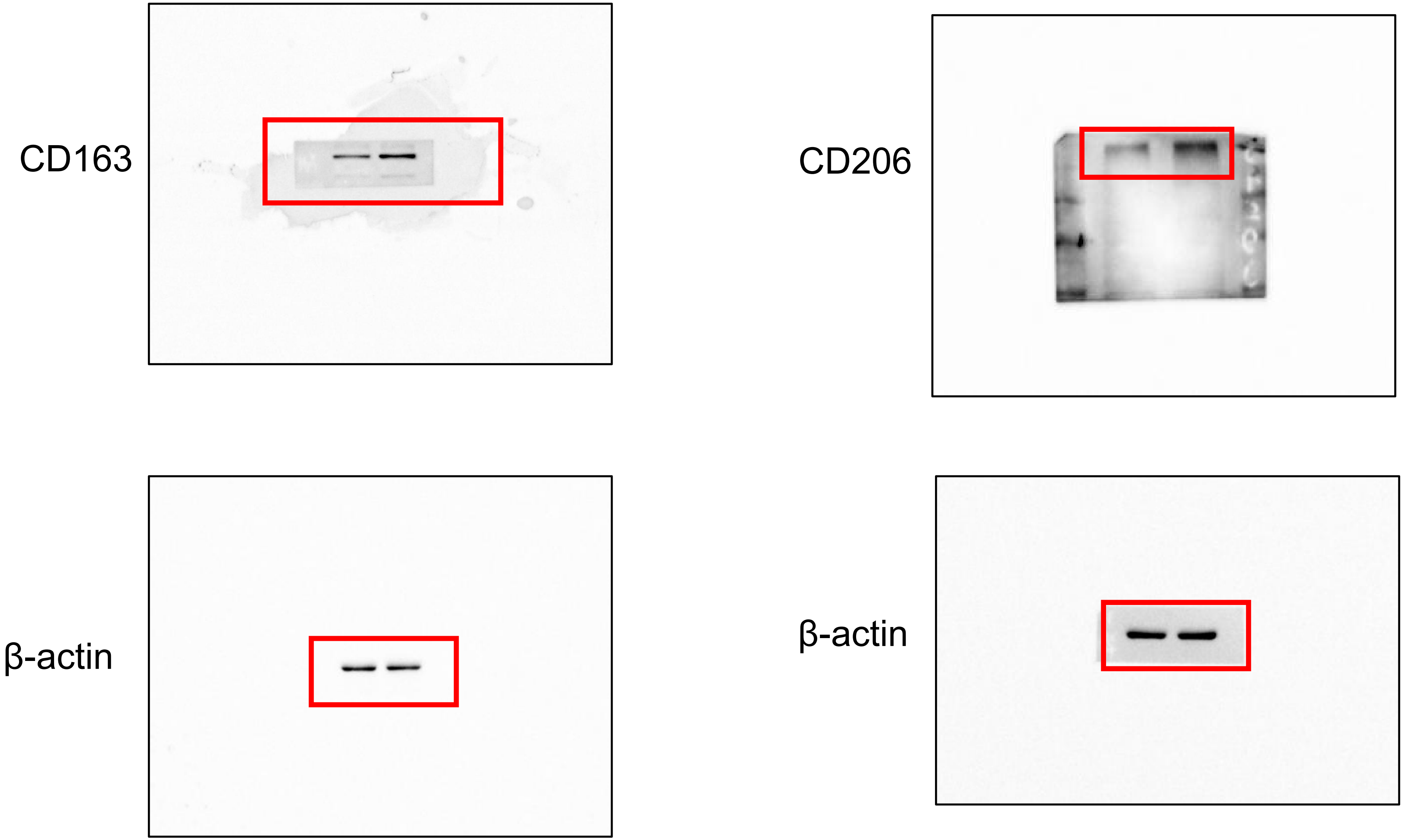

Fig. S6B

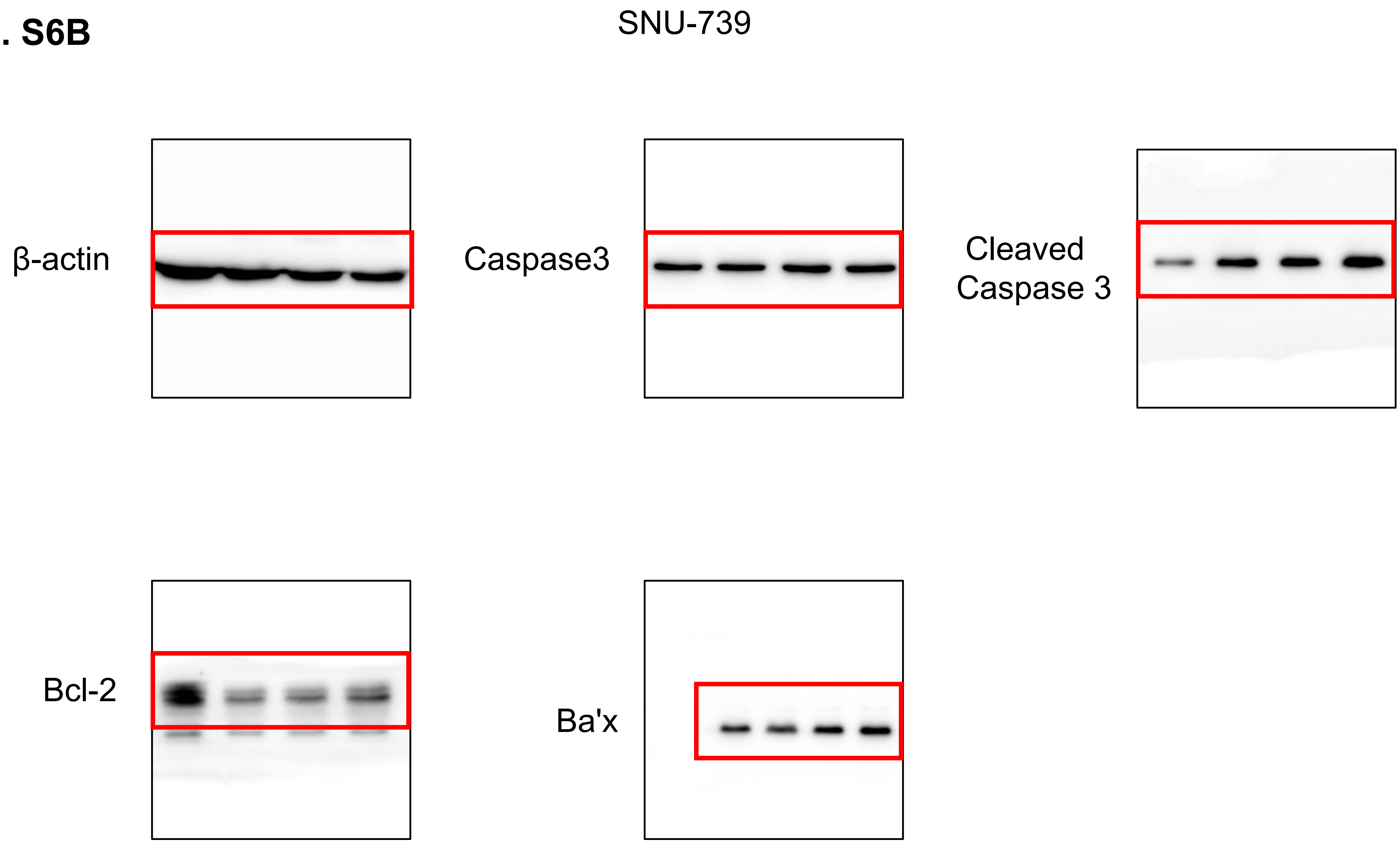

Fig. S6B

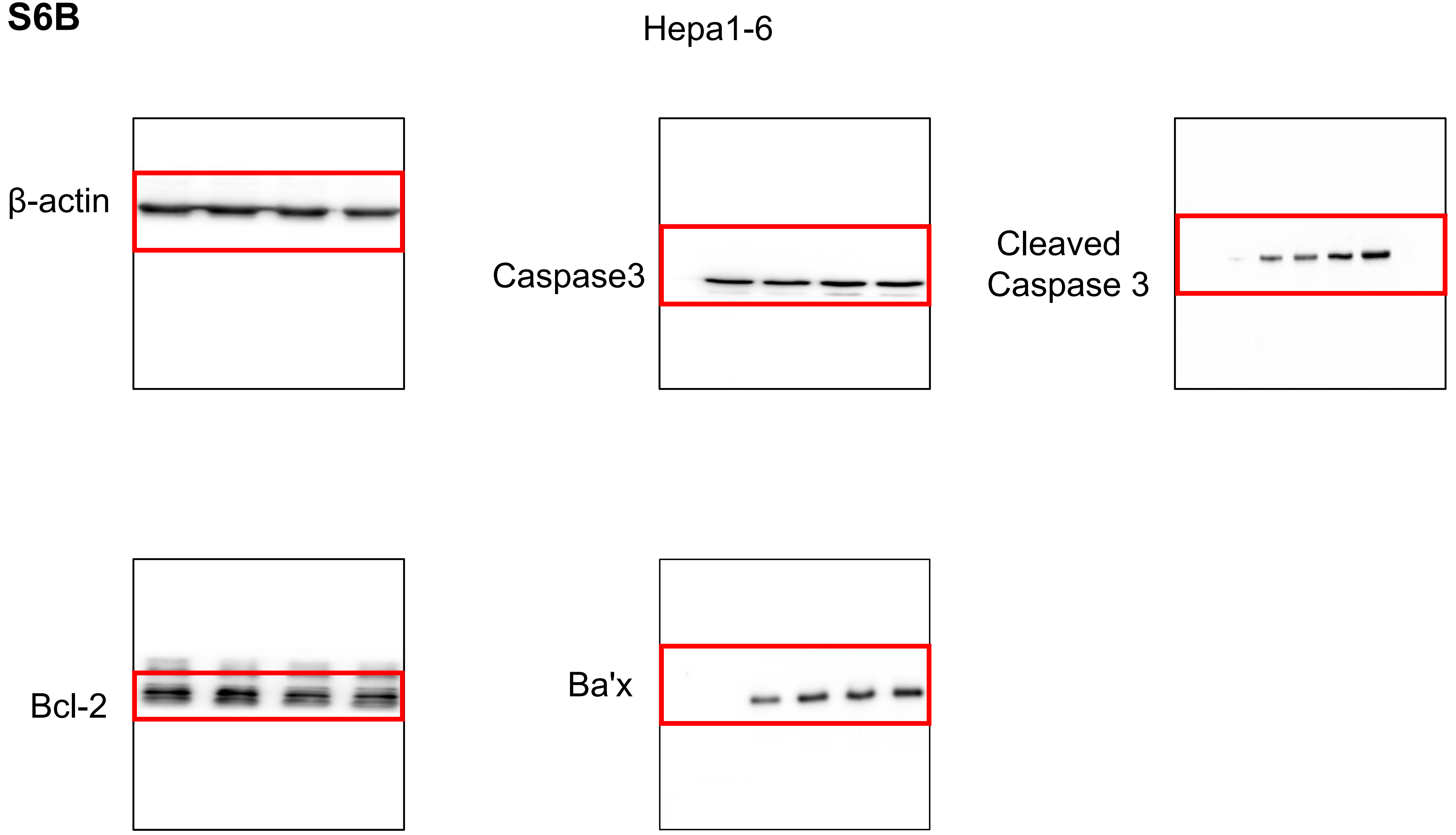

Fig. S7B

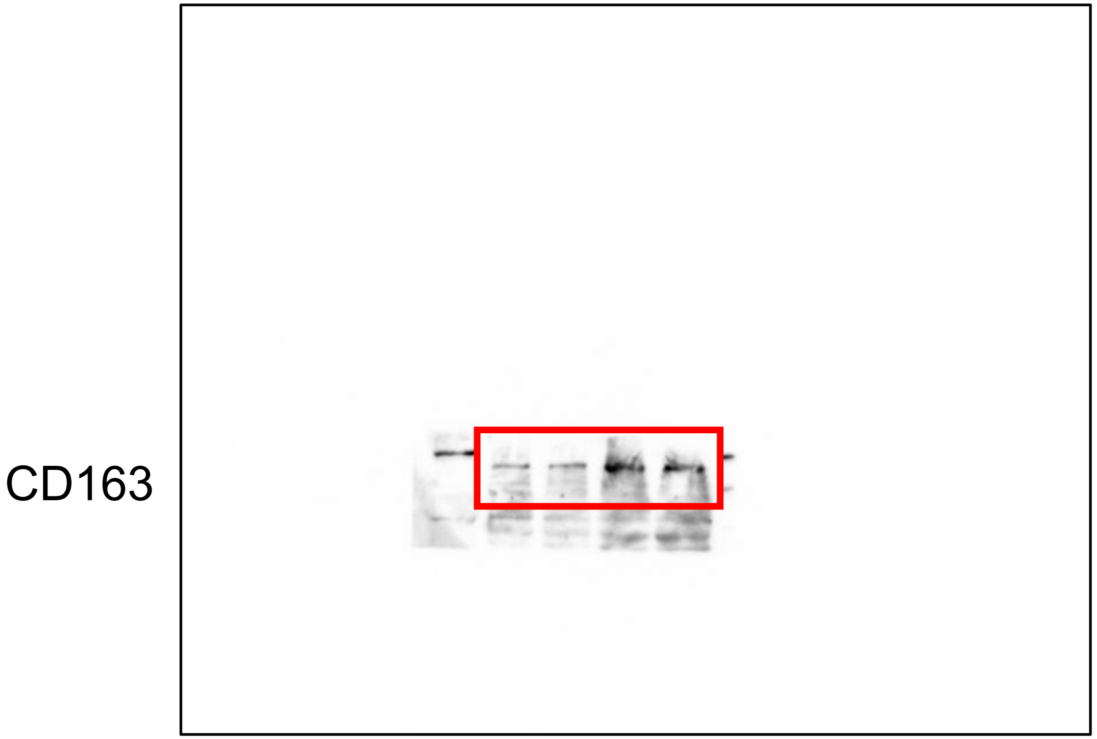

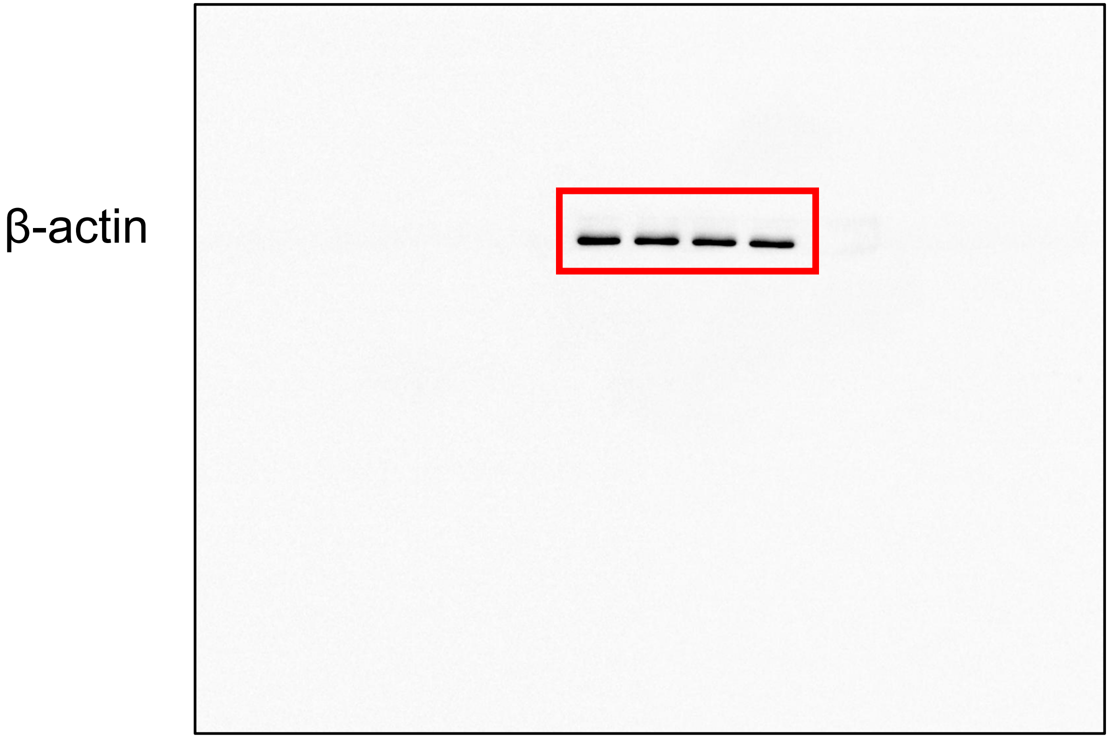

Supplement: Supplementary file 2 — Supplementary for WB [file 41419_2025_7473_MOESM2_ESM.pdf]
